# Genetic or pharmacologic inhibition of EGFR ameliorates sepsis-induced AKI

**DOI:** 10.18632/oncotarget.21244

**Published:** 2017-09-23

**Authors:** Xuan Xu, Juan Wang, Ruhao Yang, Zheng Dong, Dongshan Zhang

**Affiliations:** ^1^ Department of Emergency Medicine, Second Xiangya Hospital, Central South University, Changsha, Hunan, People's Republic of China; ^2^ Emergency Medicine and Difficult Diseases Institute, Central South University, Changsha, Hunan, People's Republic of China; ^3^ Department of Nephrology, Second Xiangya Hospital, Central South University, Changsha, Hunan, People’s Republic of China; ^4^ Department of Cellular Biology and Anatomy, Medical College of Georgia and Charlie Norwood VA Medical Center, Augusta, Georgia, USA; ^5^ Department of Emergency Medicine, Second Affiliated Hospital of Sun Yat-Sen University, Guangzhou, Guangdong, People's Republic of China

**Keywords:** CLP, EGFR, inflammation

## Abstract

Despite recent studies have demonstrated that the EGF receptor (EGFR) activation provided a renoprotective role during ischemic and folic acid-induced AKI, the role and regulation mechanism of EGFR in septic AKI remains unclear. Here, gefitinib, a highly selective EGFR inhibitor, abrogated LPS-induced phosphorylation of EGFR, ERK1/2, and STAT3 as well as expression of COX, eNOS, and proinflammatory cytokines in HK-2 cells. In addition, c-Src is an upstream of EGFR signaling pathway and mediates LPS-induced EGFR transactivation. *In vivo*, either gefitinib or genetic approaches (Wave-2 mutant mice, which have reduced EGFR tyrosine kinase activity) protected against LPS or cecal ligation and puncture (CLP) induced AKI respectively. Interestingly, the beneficial effects of gefitinib or genetic approaches were accompanied by the dephosphorylation of EGFR, ERK1/2, and STAT3, the down regulation of expression of COX, eNOS, macrophage infiltration, proinflammatory cytokines production and the renal cell apoptosis. Furthermore, mRNA array results indicated that gene families involved in cell death, inflammation, proliferation and signal transduction were down regulated in Wave-2 (Wa-2) mice. Take together, these data suggest that EGFR may mediate renal injury by promoting production of inflammatory factors and cell apoptosis. Inhibition of EGFR may have therapeutic potential for AKI during endotoxemia.

## INTRODUCTION

Acute kidney injury (AKI) would be induced by sepsis, ischemia-reperfusion (I/R) injury, trauma, and nephrotoxic agents. Among them, sepsis is considered as the most common cause (50% of all cases) in the intensive care unit (ICU) setting [1]. The mortality in septic AKI patients increases almost twofold compare with those in non-septic AKI group [2, 3]. Unfortunately, only ways of supportive care and renal replacement therapy are currently available for treatment of sepsis-induced AKI. Thus the understanding of the molecular events leading to renal damage in septic AKI may develop new therapeutic strategies to improve outcomes in this disease.

Numerous studies have demonstrated that inhibition of epidermal growth factor receptor (EGFR) alleviated renal fibrosis in various types of kidney injury models [4–7]. A few studies began to focus on the role of EGFR in AKI [8–10]. In 2013, S Zhuang and colleagues reported that inhibition of EGFR aggravated renal damage during I/R and folic acid-induced AKI [8, 11], which suggested that EGFR provided a renoprotective role in AKI. However, the subsequent studies verified that inhibition of EGFR ameliorated LPS-induced acute lung injury (ALI) by erlotinib [12]. The opposite results suggest that the role of EGFR in acute organ injury is dependent on the injury organs and stimulation factors. Although EGFR was activated in endotoxin-induced AKI [13], little is known about the role and regulation mechanism of EGFR in sepsis AKI. In view of these findings, this study was initiated to investigate whether pharmacology inhibitor or genetic approaches may ameliorate septic AKI.

In current study, we used the specific EGFR inhibitor gefitinib and Wa-2 mice, and identified EGFR as a critical factor of inflammation and apoptosis in both *in vitro* and *in vivo* experimental models of LPS and CLP induced AKI. Mechanistically, we show that LPS activated EGFR via Src, thus resulting in the activation of ERK1/2 and STAT3 signaling pathways to promote inflammation and renal cell injury. Additional global gene expression analysis showed the induction of 4831 genes by CLP induced AKI in wild-type kidneys, of which the induction of 995 genes was abrogated in Wa-2 tissues. These 995 genes included regulators of cell death, inflammation, proliferation, and signal transduction. Together, the results suggest that EGFR contributes critically to AKI by regulating multiple genes involved in kidney tissue injury.

## RESULTS

### The activation of EGFR, ERK1/2, and STAT3 pathways was induced by LPS in HK-2 cells

**Figure 1:**
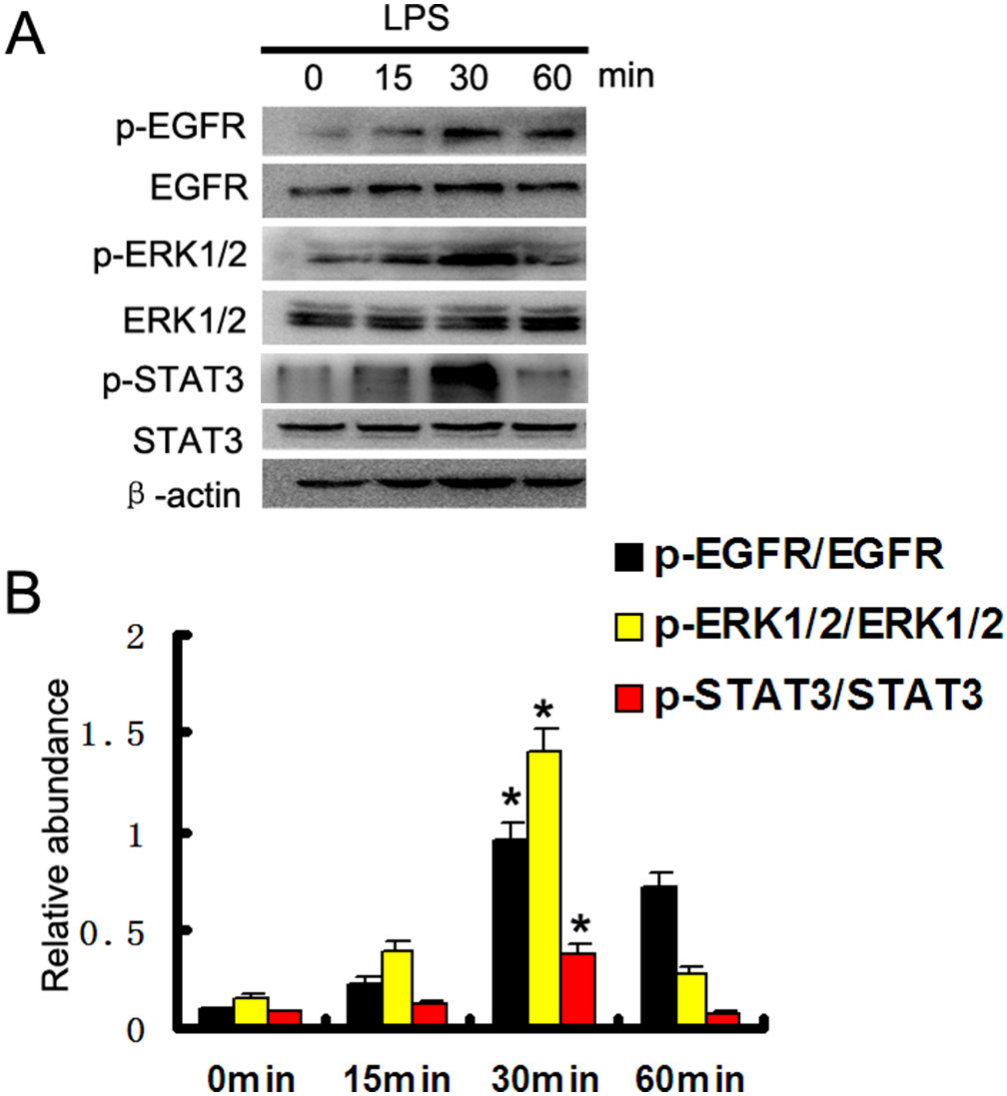
LPS induced activation of EGFR, ERK1/2, and STAT3 pathways in HK-2 cells **(A)** HK-2 cells were treated with 10μg/ml LPS for 0, 15, 30, or 60 min to collect lysate for immunoblot analysis of expression and activation of EGFR, STAT3, and ERK1/2. **(B)** Densitometric analysis of the p-EGFR/EGFR, p-ERK1/2/ERK1/2, and p-STAT3/STAT3 ratio. * *P<0.05* versus the other group. Data are representative of at least four separate experiments.

LPS is also known to induce EGFR transactivation in a variety of cell types, including epithelial cells and renal medullary collecting duct cells [14–16]. Here, we first verified whether LPS could induce the activation of EGFR pathways in HK-2 cells. Western blot assay showed that tyrosine 845 residue of EGFR started within 15 min of LPS treatment, reached peak levels at 30 minutes, and then significantly reduced at 60 minutes. We focused on ERK1/2 and STAT3 signaling, because both ERK1/2 and STAT3 were classic downstream signaling pathways of EGFR [17], and activated during LPS treatment in kidney tubular cells and tissues [10]. The results also indicated that LPS significantly activated ERK1/2 and STAT3 signaling pathways, and expression trend of them was consistent with direction of EGFR in HK2 cells (Figure 1A & 1B).

### Gefitinib suppressed LPS induced activation of ERK1/2 and STAT3 by blocking EGFR activation in HK-2 cells

**Figure 2:**
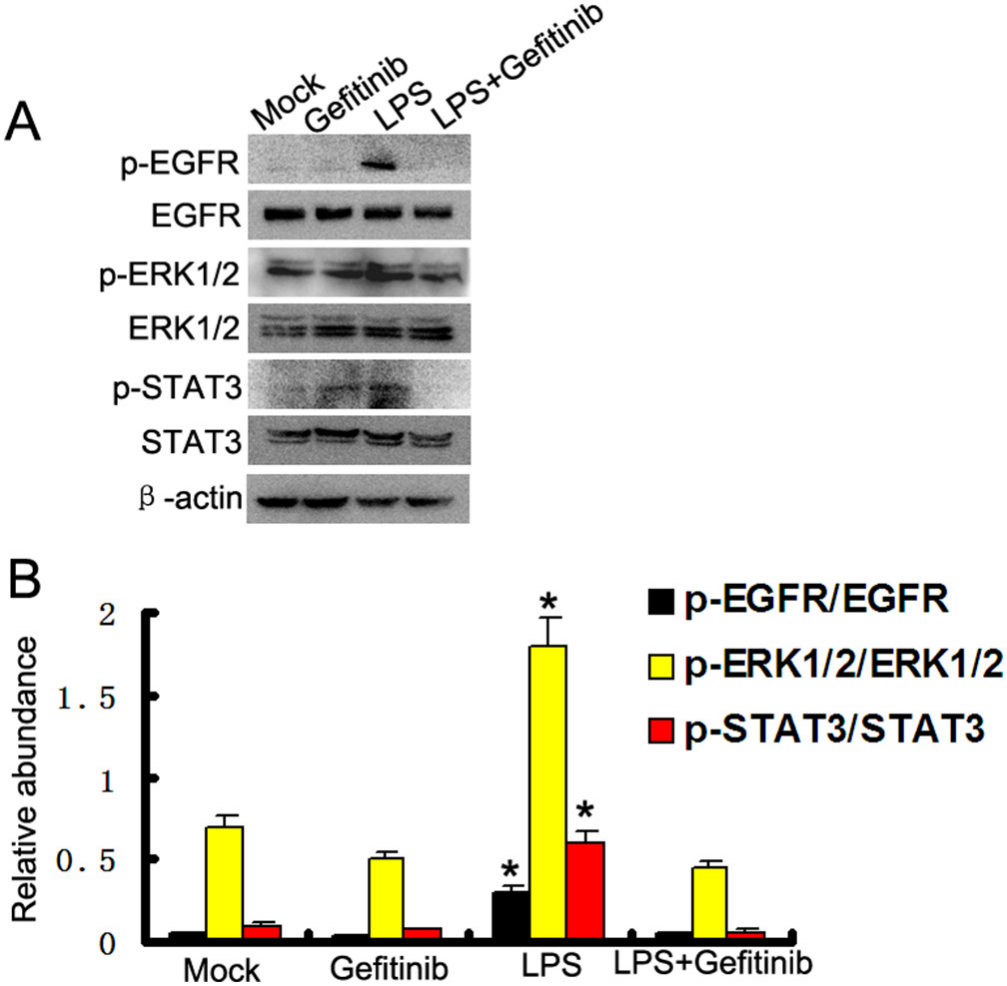
Gefitinib suppressed LPS induced ERK1/2 and STAT3 phosphorylation by blocking EGFR activation in HK-2 cells **(A)** HK-2 cells were treated with 10μg/ml LPS in presence or absence of 5 nM gefitinib for 30 min to collect lysate for immunoblot analysis of expression and activation of EGFR, STAT3, and ERK1/2. **(B)** Densitometric analysis of the p-EGFR/EGFR, p-ERK1/2/ERK1/2, and p-STAT3/STAT3 ratio. * *P<0.05* versus the other group. Data are representative of at least four separate experiments.

To further confirm the finding that LPS activated ERK1/2 and STAT3 signaling pathways through activation of EGFR, we used gefitinib, a specific EGFR inhibitor, to block EGFR activation. Western blot assay demonstrated that gefitinib markedly inhibited LPS induced EGFR phosphorylation in HK-2 cells, whereas expression levels of total EGFR were not affected. We also examined the effect of gefitinib on activation of ERK1/2 and STAT3 signaling pathways in HK-2 cells (Figure 2A & 2B). Gefitinib administration also inhibited LPS-induced activation of all these signaling pathways (Figure 2A & 2B). These data provide additional evidence for the importance of EGFR in mediating activation of ERK1/2 and STAT3 signaling pathways after LPS treatment.

### LPS induced EGFR activation depends on Src in HK-2 cells

**Figure 3:**
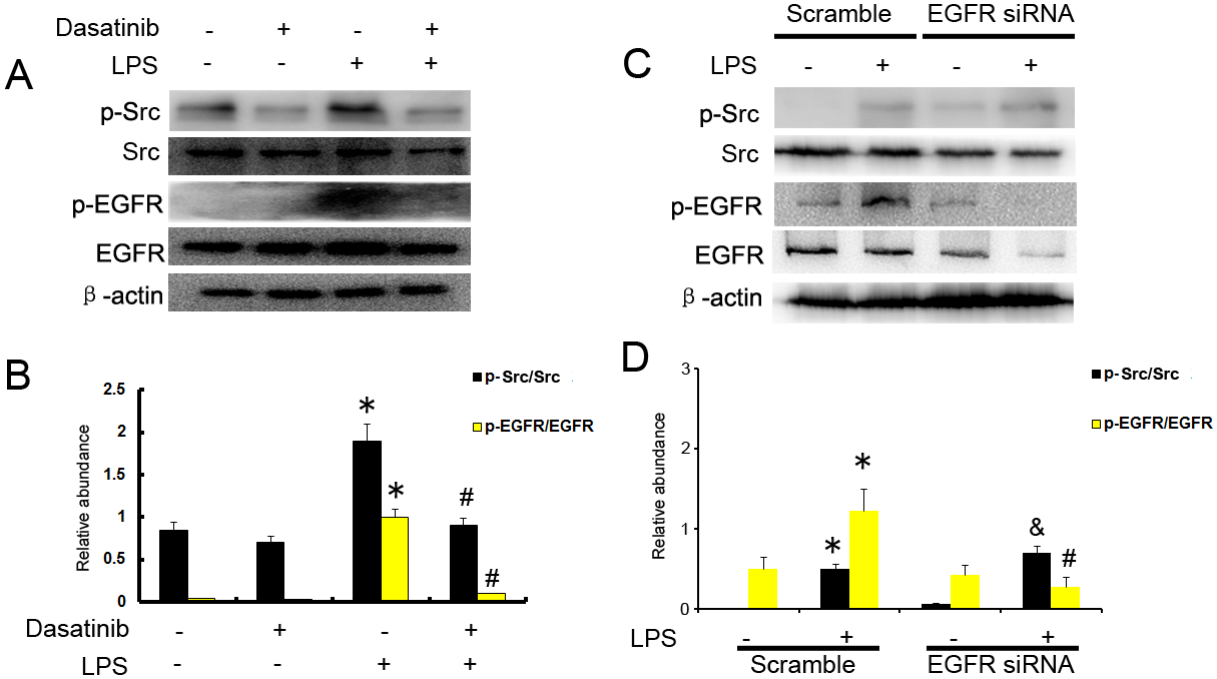
c-Src mediates the LPS induced EGFR activation in HK-2 cells **(A)** HK-2 cells were treated with 10μg/ml LPS in presence or absence of 20 nmol dasatinib for 30 min to collect lysate for immunoblot analysis of expression and activation of Src and EGFR. **(B)** Densitometric analysis of the p-EGFR/EGFR and p-Src/Src ratio. **(C)** HK-2 cells were treated with 10μg/ml LPS after the transfection of EGFR siRNA or siRNA-NC for 30 min to collect lysate for immunoblot analysis of expression and activation of Src and EGFR, densitometry **(D)** of proteins signals on immunoblots. * *P<0.05* versus mock group; # *P<0.05* versus LPS group; & *P>0.05* versus LPS group. Data are representative of at least four separate experiments.

Our results have demonstrated that LPS induced EGFR activation in HK2 cells, however, the mechanism remains unclear. Previous studies suggested that Ang-II induced EGFR activation via c-Src [4]. We presumed that c-Src might play an important role in LPS induced EGFR transactivation. HK-2 cells were pretreated with the c-Src inhibitor dasatinib (20 nmol) for 30 mins before LPS (10μg/ml ) stimulation. Dasatinib was shown to inhibit the phosphorylation of c-Src and EGFR stimulated by LPS (Figure 3A & 3B). However, EGFR siRNA had no effect on LPS–induced Src phosphorylation (Figure 3C & 3D). These data suggest that c-Src is an upstream of EGFR signaling pathway and mediates LPS–induced EGFR transactivation.

### ERK1/2 and STAT3 mediated different inflammation gene expression induced by LPS

**Figure 4:**
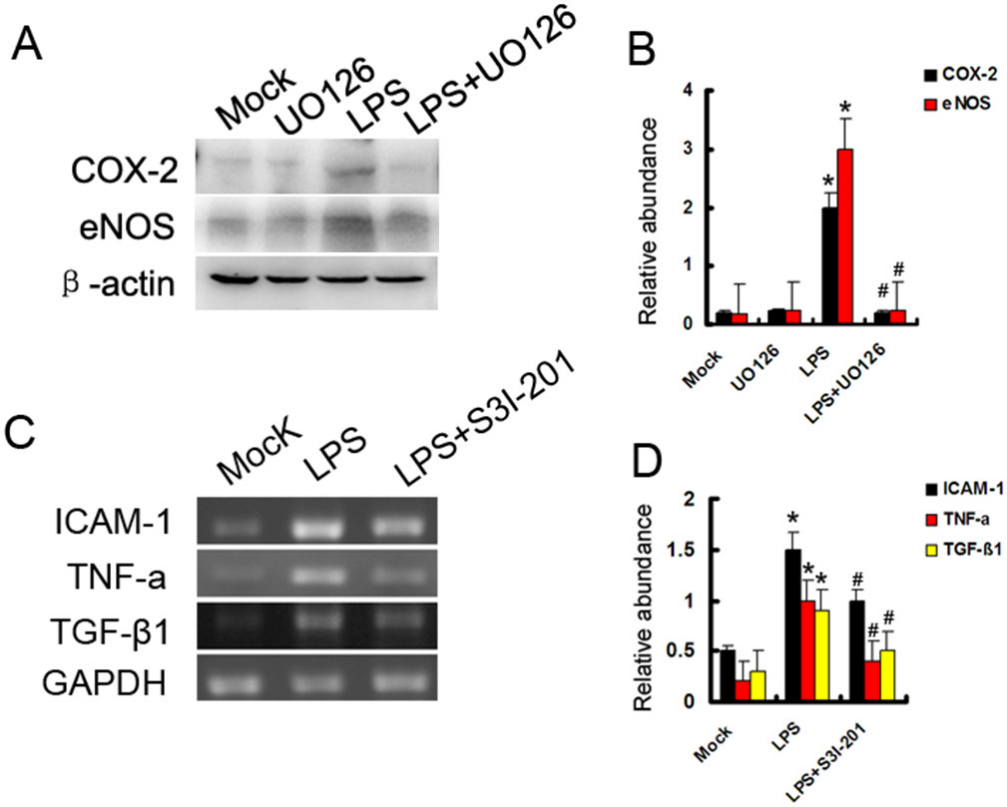
Both ERK1/2 and STAT3 play different role in LPS induced gene expression **(A)** HK-2 cells were treated with 10μg/ml LPS in presence or absence of 400 nmol U0126 for 24 h to collect lysate for immunoblot analysis of expression of COX-2 and eNOS. **(B)** Densitometric analysis of the COX-2/β-actin and eNOS/β-actin. **(C)** HK-2 cells were also treated with 10μg/ml LPS in presence or absence of 50μM S3I-201 for 24 h to collect lysate for RT-PCR analysis of expression of ICAM-1, TNF-α, and TGF-β1. **(D)** Densitometric analysis of the ICAM-1/GAPDH, TNF-α/GAPDH, and TGF-β1/GAPDH ratio. * *P<0.05* versus mock group; #*P<0.05* versus LPS group. Data are representative of at least four separate experiments.

ERK is activated in response to pro-inflammatory cytokines [18]. Both eNOS and COX-2 may mediate AKI [19, 20]. In this study, ERK inhibitor U0126 prior to treatment with LPS in HK-2 cells. As shown in Figure 4A and 4B, compared with mock or U0126 group, both eNOS and COX-2 were significantly increased by LPS, which was suppressed by U0126. STAT3 was also activated in LPS-induced AKI [21]. We further demonstrated whether STAT3 signaling played an important role in LPS-induced inflammation cytokines. STAT3 inhibitor S3I-201 prior to treatment with LPS in HK-2 cells. As shown in Figure 4C and 4D, compared with mock or S3I-201 group, the expression levels of ICAM-1, TNF-α, and TGF-β were markedly increased by LPS, which was blocked by S3I-201. The data suggest that ERK1/2 and STAT3 played different role in LPS- induced inflammation gene expression respectively.

### Gefitinib reduces kidney injury and renal dysfunction in LPS-induced AKI mice model

**Figure 5:**
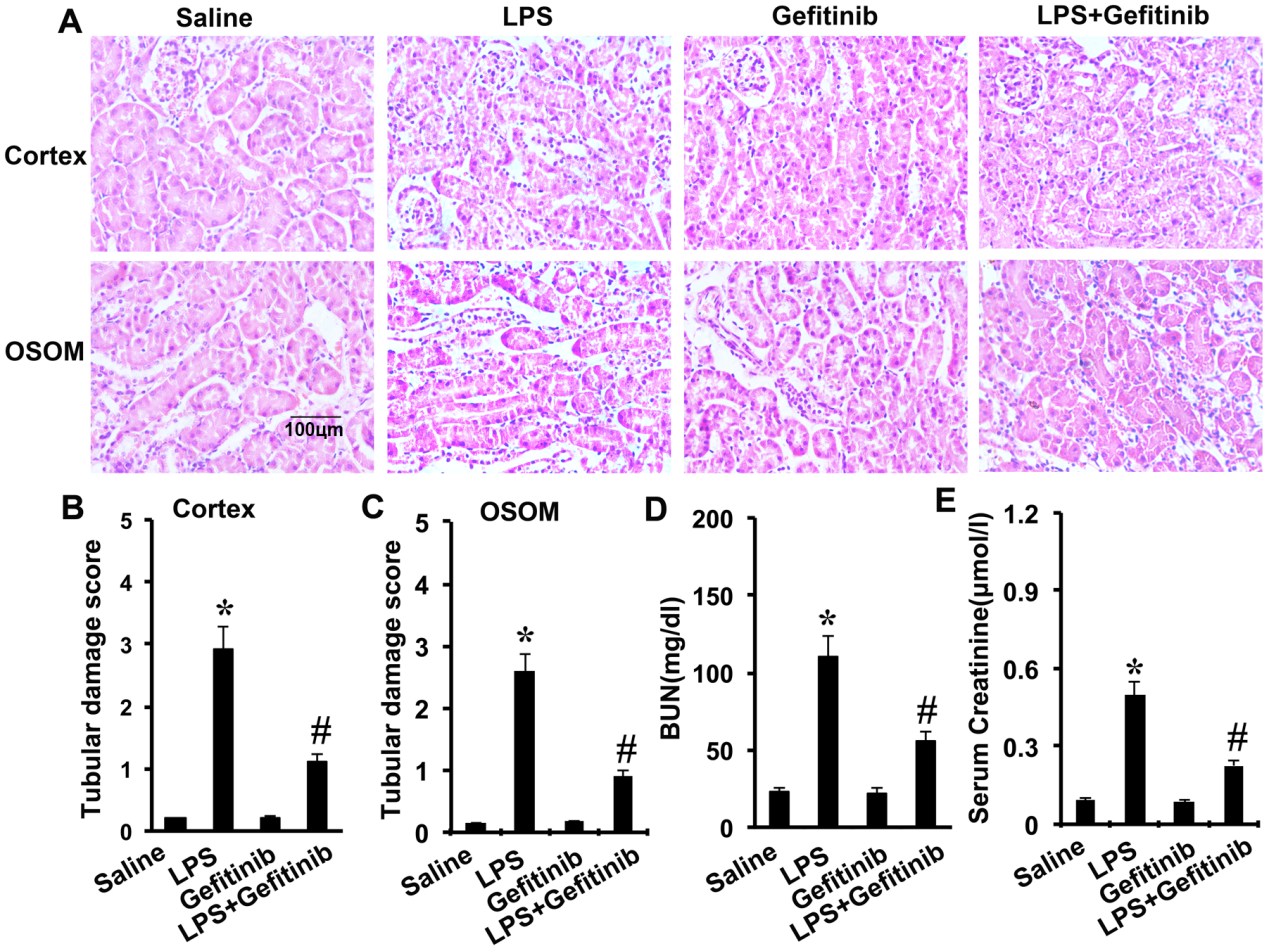
Effect of gefitinib on LPS-induced renal tissue damage, BUN, and serum creatinine in mice Male C57 mice were injected with 10 mg/kg LPS with or without 100 mg/kg gefitinib. As a control, the mice were injected with saline. Kidney tissues and blood samples were collected at 24 h after treatment. **(A)** Kidney sections were stained with hematoxylin and eosin to assess tubular damage. Tubular damage scores of kidney cortex **(B)**, tubular damage scores of OSOM **(C)**, BUN **(D)**, and serum creatinine **(E)**. Data are shown as mean ± S.E.M. (n=10). Data are presented as mean ± SEM; * *P<0.05* versus saline group; #*P<0.05* versus LPS group. Data are represe ntative of at least four separate experiments. Original magnification, x200 in A.

LPS-induced kidney injury was shown in the tubules in both renal cortex and OSOM. The tubular damage was markedly attenuated by gefitinib (Figure 5A-5C). To analyze kidney function, BUN and serum creatinine were determined. As shown in Figure 5D and 5E, LPS induced significant increases in both BUN and serum creatinine levels, which were significantly attenuated by cotreatment with gefitinib.

### Gefitinib inhibited macrophages infiltration and expression of ICAM-1, TNF-α, and TGF-β in LPS-induced AKI mice

**Figure 6:**
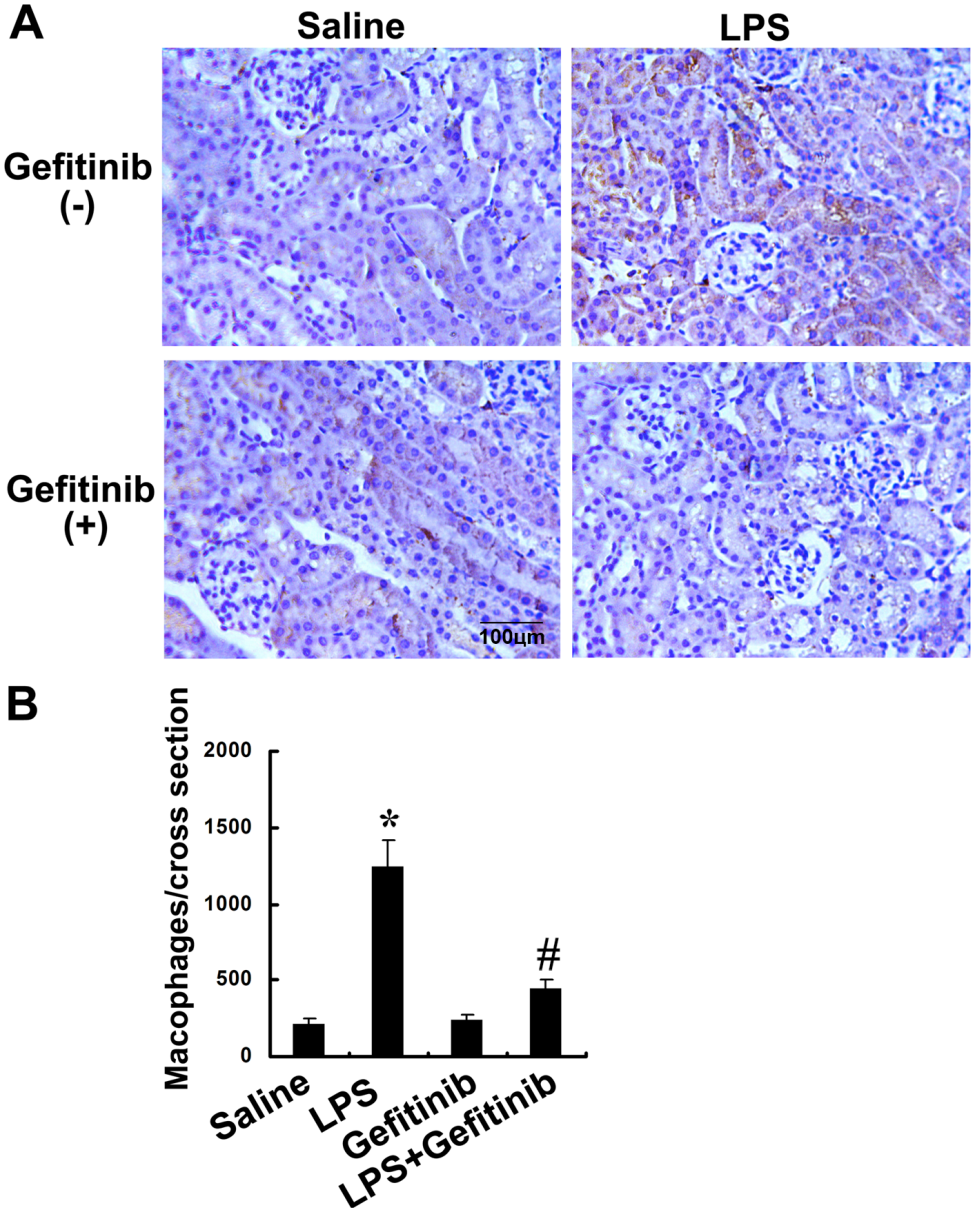
Gefitinib inhibited infiltration of macrophages in a LPS induced AKI model Male C57 mice were injected with 10 mg/kg LPS with or without 100 mg/kg gefitinib. As a control, the mice were injected with saline. Kidney tissues were collected at 24 h after treatment. **(A)** Representative macrophage immunohistochemistry from kidney cortical tissues of different groups at 24h (original magnification, ×400). **(B)** Quantitation of macrophage-positive cells per total cross-sectional area was performed from each group (n=10). Data are presented as mean ± SEM; * *P<0.05* versus saline group; #*P<0.05* versus LPS group. Data are representative of at least four separate experiments. Original magnification, x200 in A.

**Figure 7:**
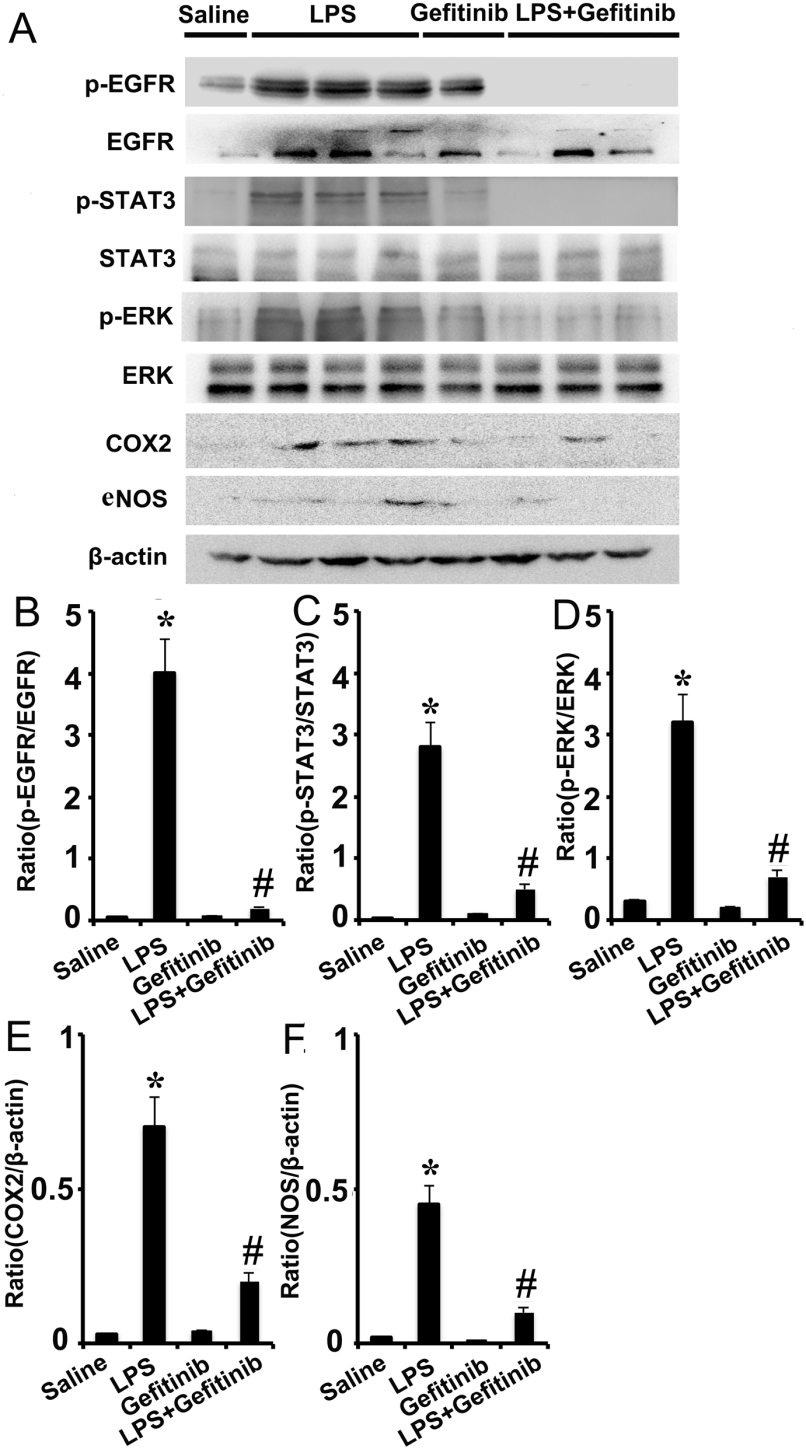
Gefitinib suppressed phosphorylation of ERK1/2 and STAT3, and expression of COX-2 and eNOS by blocking EGFR activation in a LPS induced AKI model Male C57 mice were injected with 10 mg/kg LPS with or without 100 mg/kg gefitinib. As a control, the mice were injected with saline. Kidney tissues were collected at 24 h after treatment. **(A)** Immunoblot analysis of expression and activation of EGFR, STAT3, and ERK1/2, and expression of COX-2 and eNOS. **(B-F)** Densitometric analysis of the p-EGFR/EGFR, p-ERK1/2/ERK1/2, p-STAT3/STAT3, COX-2/β-actin, and eNOS/β-actin ratio. Data are presented as mean ± SEM; * *P<0.05* versus saline group; #*P<0.05* versus LPS group. Data are representative of at least four separate experiments.

We examined the effect of gefitinib on macrophage infiltration and expression of ICAM-1, TNF-α, TGF-β, and IL-1-β. Analysis of kidney sections by immunochemistry showed a prominent interstitial infiltration of macrophages after LPS-induced kidney injury, which was significantly reduced by gefitinib (Figure 6A & 6B). The expression levels of ICAM-1, TNF-α, TGF-β, and IL-1-β were increased after LPS-treated mice, all of them except for IL-1-β were also markedly reduced by gefitinib (Figure 7A-7E). These data suggest that gefitinib suppresses macrophage infiltration and proinflammatory cytokines by inhibiting EGFR activity in LPS-induced AKI mice model.

### Gefitinib blocked LPS-induced activation of EGFR/ERK1/2 or STAT3 and the expression of COX-2 and eNOS in kidney tissues

**Figure 8:**
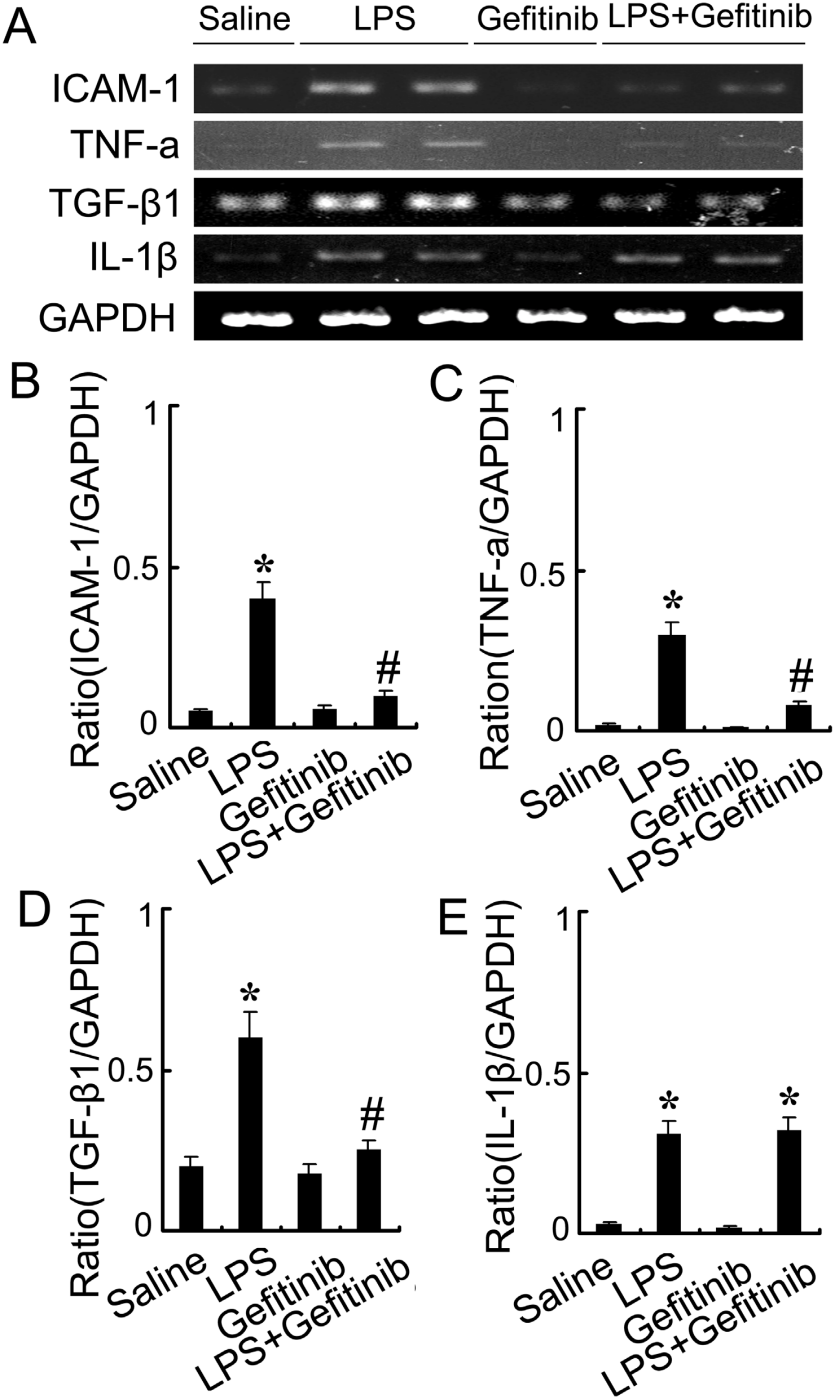
Gefitinib suppressed expression of ICAM-1, TNF-α, and TGF-β1 by blocking EGFR activation in a LPS induced AKI model Male C57 mice were injected with 10 mg/kg LPS with or without 100 mg/kg gefitinib. As a control, the mice were injected with saline. Kidney tissues were collected at 24 h after treatment. **(A)** RT-PCR analysis of expression of ICAM-1, TNF-α, TGF-β1, and IL-1β. **(B-E)** Densitometric analysis of the ICAM-1/GAPDH, TNF-α/GAPDH, and TGF-β1/GAPDH, IL-1β**/** GAPDH ratio. Data are presented as mean ± SEM; * *P<0.05* versus saline group; #*P<0.05* versus LPS group. Data are representative of at least four separate experiments.

We further determined the involvement of ERK1/2 and STAT3 signaling in EGFR signaling *in vivo* during LPS-induced AKI mice model. LPS induced phosphorylation of ERK1/2 and STAT3 in kidney tissues, indicative of the activation of the ERK1/2 and STAT3 signaling for expression of COX-2 and eNOS, and production of proinflammatory cytokines respectively. Notably, two pathways were significantly suppressed by gefitinib at LPS-treated mice (Figure 8). These *in vivo* data were consistent with our cell culture results (Figures 4 & 5), further suggesting that both ERK1/2 and STAT3 signaling were downstream of EGFR during LPS-induced AKI mice.

### CLP induced kidney injury and renal dysfunction was ameliorated in Wa-2 mice

**Figure 9:**
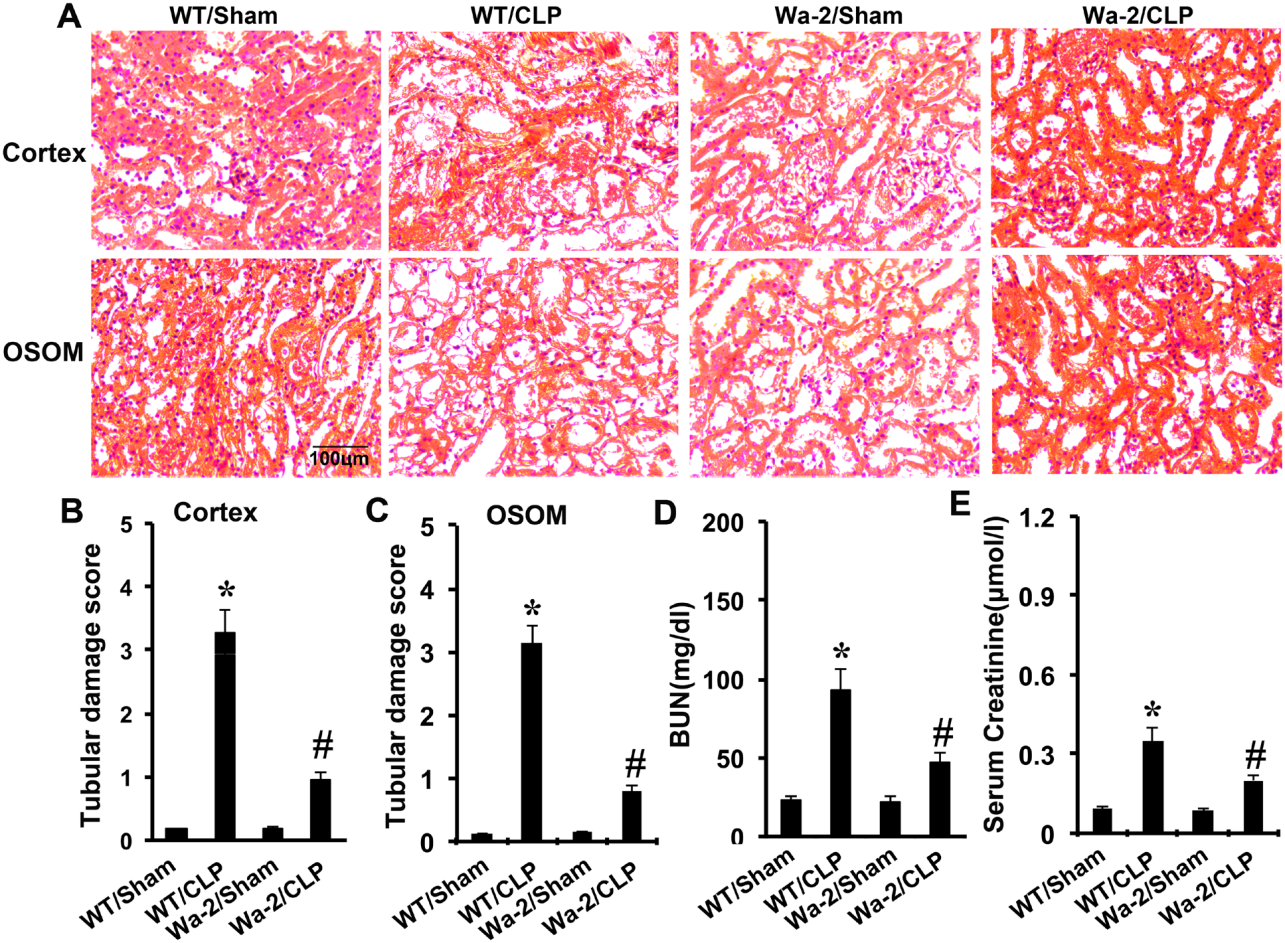
CLP induced AKI is attenuated in Wa-2 mice Wild-type and Wa-2 littermate mice were subjected to CLP. Sham-operated mice were used as a control. Kidney tissues and blood samples were collected at 18 h after treatment. **(A)** Kidney sections were stained with hematoxylin and eosin to assess tubular damage. Tubular damage scores of kidney cortex **(B)**, tubular damage scores of OSOM **(C)**, BUN **(D)**, and serum creatinine **(E)**. Data are shown as mean ± S.E.M. (n=8); * *P<0.05* WT/Sham group; #*P<0.05* versus CLP group. Data are representative of at least four separate experiments. Original magnification, x200 in A.

CLP-induced kidney injury was shown in the tubules in both renal cortex and OSOM, which was markedly attenuated in Wa-2 mice (Figure 9A-9C). As shown in Figure 9D and 9E, CLP induced significant increases in both BUN and serum creatinine levels, which were markedly reduced in Wa-2 mice.

### CLP induced renal cell apoptosis was ameliorated in Wa-2 mice

**Figure 10:**
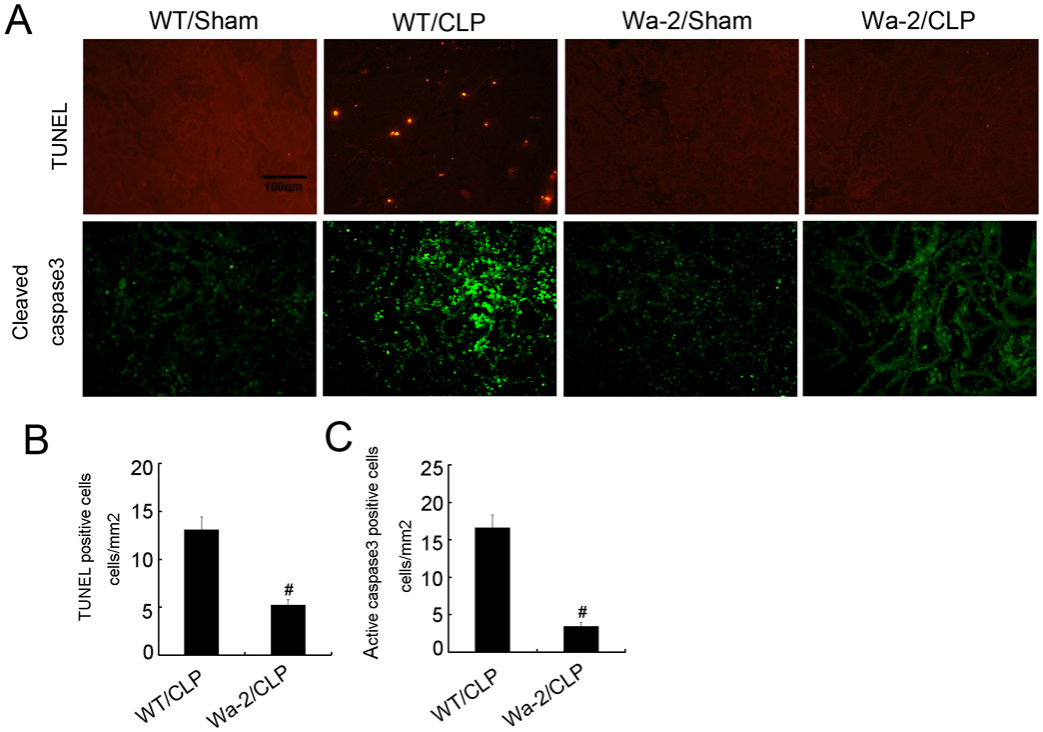
CLP induced renal cell apoptosis is reduced in Wa-2 mice Wild-type and Wa-2 littermate mice were subjected to CLP. Sham-operated mice were used as a control. Kidney tissues were collected at 18 h after treatment. **(A)** TUNEL assay and cleaved caspase3 to reveal apoptosis (original magnification, ×200). Quantification of TUNEL-positive cells **(B)** and cleaved caspase3 **(C)** in CLP-treated cortical tissues. Data were expressed as means ± SD; * *P<0.05* versus Wa-2 with CLP group. Data are representative of at least four separate experiments.

Previous study has demonstrated apoptosis play a pivotal role in AKI [22], whether EGFR promotes apoptosis in septic AKI need to be investigated. The terminal deoxynucleotidyl transferase mediated digoxigenin deoxyuridine nick-end labeling (TUNEL) staining for analysis of apoptosis, and immunofluorescence for active caspase 3 in kidney cortical tissues. The positive cells of TUNEL and active caspase 3 were lower in the kidney tissues of sham operated mice, after CLP treatment, it was significantly increased in kidney cortical tissues in wild-type mice, and the rise was markedly suppressed in Wa-2 mice (Figure 10A). The number of positive cells of TUNEL and active caspase 3 in cortical and outer medulla regions further demonstrated the above-mentioned observation (Figure 10B & 10C).

### The macrophages infiltration and expression of ICAM-1, TNF-α, and TGF-β were suppressed in Wa-2 mice

Inflammation plays an important role in CLP induced AKI. Hence, we examined macrophage infiltration and expression of ICAM-1, TNF-α, and TGF-β. The immunochemistry showed a prominent interstitial infiltration of macrophages after CLP-induced kidney injury, which was significantly suppressed in Wa-2 mice (Supplementary Figure 1A & 1B). The expression levels of ICAM-1, TNF-α, and TGF-β were increased after CLP-treated mice, which were also markedly reduced in Wa-2 mice (Supplementary Figure 2A & 2B). These data suggest that EGFR is responsible for macrophage infiltration and proinflammatory cytokines in CLP-induced AKI mice model.

### EGFR is required for ERK1/2 and STAT3 activation and the expression of COX-2 and eNOS in Wa-2 mice

Firstly, we determined whether EGFR is responsible for activation of ERK and STAT3 and expression of COX-2 and eNOS in CLP-induced AKI mice model. After CLP surgery, the phosphorylation of ERK1/2 and STAT3 and expression of COX-2 and eNOS were significantly increased in WT mice (Supplementary Figure 3A & 3B), which was markedly suppressed in Wa-2 mice. These *in vivo* data were consistent with gefitinib inhibitor (Figures 4 & 5), which further supported that EGFR is required for activation of ERK1/2 and STAT3 in septic-induced AKI.

### Down regulated genes were selected by analysis of functional enrichment and KEGG in Wa-2 mice with CLP treatment

To gain a comprehensive understanding of EGFR-regulated gene expression in septic AKI, we conducted a global gene expression analysis. To this end, RNA samples were isolated from kidney cortex, amplified, and hybridized to a cDNA microarray containing 44, 000 genes. We selected the differentially expressed genes from all microarray data by filtering on expression level (≥ 2-fold). Using these selection criteria, we found 4831 genes to be up regulated and 5667 genes to be down regulated in the wide type kidneys with CLP treatment (Supplementary Tables 1-2). Compared with the wide type kidneys after CLP, we found 1255 genes to be up regulated and 995 genes to be down regulated in the Wa-2 mice kidneys (Supplementary Tables 3-4). Moreover, GO analysis, a functional analysis associating differentially expressed mRNAs with GO categories, was carried out to determine the gene and gene product enrichment. Fisher’s exact test is used to find whether there is more overlap between the DE list and the GO annotation list than would be expected by chance, the lower the P-value, the more significant the GO term (P-value cut-off of ≤0.05 was recommended). We found that the highest enriched GOs targeted by up regulated or down regulated transcripts were cellular metabolic process (GO: biological processes), intracellular component (GO: cellular components), and protein binding (GO: molecular functions) (Supplementary Figure 4A & 4B). Of note, 995 down-regulated genes in Wa-2 mice were associated with cell death, JAK-STAT cascade, ERK1 and ERK2 cascade, positive regulation of MAPK cascade, macrophage differentiation, I-κB kinase/NF-κB signaling, and nitric oxide biosynthetic process (Supplementary Table 5 ). Furthermore, the expression levels of homeodomain interacting protein kinase 2 (HIPK-2), activating tranSrciption factor 3 (ATF3), NACHT, LRR and PYD domains-containing protein 3 (NLRP3), and Rho-associated protein kinase 2 (ROCK2 ) were significantly up regulated in CLP induced AKI model, which was markedly suppressed in Wa-2 mice were subjected to CLP (Supplementary Figure 5). Based on the latest Kyoto Encyclopedia of Genes and Genomes (KEGG) database, we provided pathway analysis for differentially expressed mRNAs. This analysis allowed us to determine the biological pathway, which showed a significant enrichment of differentially expressed mRNAs. The pathway analysis indicated that there were 10 pathways corresponding to the up regulated transcripts (Supplementary Figure 6A). By contrast, there were 10 pathways involved in the down regulated transcripts including tumor necrosis factor (TNF), advanced glycosylation end products (AGE) - receptor for advanced glycation end products (RAGE), mitogen-activated protein kinase (MAPK), and NOD like receptor signaling pathway. Significant pathways corresponding to down regulated mRNAs appeared to be responsible for cell death, inflammation, proliferation (Supplementary Figure 6B) , which played a critical role in CLP-induced AKI.

## DISCUSSION

Growing evidence suggests that the EGFR plays a renoprotective role during folic acid and ischemic induced AKI. However, the role and underlying mechanism in septic AKI remains unclear. These studies for the first time demonstrate that LPS induced Src phosphorylation to activate EGFR, activation EGFR mediated phosphorylation of ERK and STAT3, which increase expression of COX2 and eNOS, and production of cytokine to mediate kidney injury respectively. Furthermore, the inflammation and tubular injury were significantly ameliorated by using genetic and pharmacological approaches in LPS or CLP induced AKI.

Previous studies has demonstrated that Toll Like Receptor 4 (TLR4) rapidly transactivates the EGFR via protease-mediated EGFR ligand shedding, which established a link between LPS and EGFR signaling [15, 23]. However, the mechanism by which LPS activates EGFR signaling is still unclear**.** In current study, in HK-2 cells, we showed that the c-Src inhibitor dasatinib significantly blocked LPS induced EGFR phosphorylation, and knock down by EGFR siRNA had no effect on LPS induced c-Src phosphorylation (Figure 3). These data indicate that c-Src function as an upstream signaler to mediate EGFR transactivation by LPS, which is consistent with these studies that Ang II and TGF-β activated EGFR signaling pathway via c-Src [4, 6].

Although recent studies shown that EGFR kinase activity was required for TLR4-mediated activation of NF-κB signaling during septic shock [9, 12], whether it regulates ERK1/2 and STAT3 signaling pathways remains unclear. In this study, inhibition of EGFR by gefitinib and Wa-2 mice, significantly suppressed activation of the two signaling pathway (Figures 2 & 7 and Supplementary Figure 3), which suggested that EGFR kinase activity was required for TLR4-mediated activation of them during septic AKI. Firstly, we observed rapid (15min) activation of ERK1/2 signaling following by LPS treatment, which was supported by those of previous studies demonstrating TLR4-dependent increases in ERK1/2 phosphorylation after LPS exposure [24, 25]. As we know, both eNOS and COX-2 are injury mediators for AKI [19, 20]. Interestingly, inhibition of ERK1/2 by U0126 of a pharmacological inhibitor significantly suppressed the expression of eNOS and COX-2 (Figure 4A & 4B). These data suggested that ERK1/2 is responsible for EGFR mediated expression of eNOS and COX-2 (Figure 7, and Supplementary Figure 3). Secondly, the janus kinase-signal transduction and activator of tranSrciption (JAK-STAT) signaling pathway is essential for cytokine receptor signaling involved in immune and inflammatory responses, and activated STAT proteins bind specific sequences and promote transcription of cytokines and inflammatory cytokines [26]. Previous study revealed that inhibition of the JAK/STAT pathway attenuated sepsis induced multiple organ dysfunction [27–29]. In line with those observations, we here demonstrate that S3I-201, a STAT3 inhibitor, significantly suppressed LPS induced expression levels of ICAM-1, TNF-α, and TGF-β excluding IL-β (Figure 4C & 4D), which was also suppressed by using genetic and pharmacological approaches in kidney from LPS or CLP induced AKI models for 24h or 18h (Figure 8, and Supplementary Figure 2). However, recent one study reported that gefitinib had no effect on serum TNF-α after LPS for 4 h in lung tissue [9]. The possibility explanation is that the examination time point and organ release of TNF-α has significant different. These data suggested that STAT3 is responsible for EGFR mediated expression of ICAM-1, TNF-α, and TGF-β. Collectively, current observations indicate that inhibition of EGFR protects kidney injury and is associated, at least in part, with significant inhibition of ERK1/2 and STAT3 signaling in mice with sepsis.

To further clarify the EGFR-mediated gene expression in septic AKI, we used a global gene expression analysis using a cDNA microarray containing 44,000 genes. In wild-type mice, CLP induced AKI for 18 hours led to the up regulation of 4831 genes and the down regulation of 5667 genes. Notably, compared with wild-type tissues, 1255 genes to be up regulated, and 995 genes were suppressed in Wa-2 kidneys during CLP induced AKI, suggesting that these genes are subjected to EGFR in AKI. Interestingly, the down regulated genes of 995 include important regulators of cell death, JAK-STAT cascade, ERK1/2 cascade, positive regulation of MAPK cascade, macrophage differentiation, I-kB kinase/NF-kB signaling, and nitric oxide biosynthetic process (Supplementary Table 5 ), which consisted with the *in vitro* finding (Figures 2 & 4). Immunoblot analysis further confirmed the induction of several interesting genes (i.e., HIPK-2, ATF3, NLRP3, and ROCK2) during CLP induced AKI in wild-type mice, which was suppressed in Wa-2 mice (Supplementary Figure 5). HIPK2 is a serine/threonine protein kinase that participates in the regulation of diverse cellular activities including cell apoptosis [30]. ATF3 is also a member of the mammalian activation transcription factor/cAMP responsive element-binding (CREB) protein family of transcription factors, and involved in cell apoptosis [31]. The down regulation of HIPK2 and ATF3 in Wa-2 mice with CLP treatment suggests that EGFR may also be involved in renal cell apoptosis in AKI. In support of this possibility, Wa-2 mice showed less cell apoptosis in renal cortex and medulla after CLP induced AKI (Figure 10). In addition, although EGFR usually mediates cell proliferation and anti-apoptotic effects, our current study showed that activation of EGFR induced apoptosis, the possibility explanation is that activation of EGFR significantly increased proinflammatory cytokines production(Figure 4C & 4D), including TNF-α and TGF-β, which can promote kidney cell apoptosis [32, 33]. Furthermore, NLRP3 plays a crucial role in innate immunity and inflammation, which has been reported in CLP induced AKI [34]. ROCK2 involves in cellular motility, migration, adhesion, and inflammation, inhibition of it ameliorated the ischemic induced AKI [35]. Thus, the pathologic role of EGFR in AKI may not be limited to the regulation of renal cell death/survival; rather, it may be extended to the regulation of inflammation, which was possibility supported by Wa-2 mice showed less macrophage infiltration and lower expression of ICAM-1,, TNF-α, and TGF-β. Furthermore, the KEGG analysis was used to determine the biological pathway, which showed a significant enrichment of differentially expressed mRNAs. In the pathway analysis, 10 pathways were indentified to corresponding to the upregulated transcripts (Supplementary Figure 6A). By contrast, there were 10 pathways involved in the down regulated transcripts, the TNF, AGE-RAGE, MAPK, and NOD like receptor signaling pathway of them associated with cell death and inflammation suggested involvement in EGFR-mediated CLP induced AKI. It is important to recognize that the differential gene expression shown in Wa-2 and wild-type kidney tissues after CLP induced AKI may also be secondary to the lower injury associated with the Wa-2 model.

In summary, our data show a key role for EGFR activation in mediating development of inflammation and renal cell apoptosis after CLP or LPS induced AKI. Mechanistically, EGFR kinase activity mediated activation of ERK1/2 and STAT3 signaling pathways to induce inflammation and renal cell injury. Global gene expression analysis has further identified 995 genes were induced in CLP induced AKI through EGFR, which may regulate cell death, inflammation, proliferation, and signal transduction. Additional investigation in these directions will generate significant new insights into EGFR regulation of kidney injury. The pharmacological inhibition of the EGFR activation may represent a novel treatment for patients suffering from sepsis induced AKI by microbial infection.

## MATERIALS AND METHODS

### Antibodies and reagents

Antibodies were purchased from the following sources: p-EGFR, EGFR, p-ERK1/2, ERK1/2, p-STAT3, STAT3, p-Src, Src, COX-2, eNOS, HIPK-2, ATF3, NLRP3, and ROCK2 were purchased from Cell Signaling Technology (Dancers, MA); The secondary antibodies for immunoblot analysis were from Jackson Immunoresearch (West Grove, PA). LPS was purchased from Sigma (St. Louis, MO). Gefitinib, dasatinib, U0126, and S3I-201 were purchased from AstraZeneca (Macclesfield, England).

### Cell culture and grouping

HK-2 cells were cultured in DMEM (Sigma-Aldrich, St. Louis, MO) containing 10% fetal bovine serum, 0.5% penicillin, and streptomycin in an atmosphere of 5% CO2–95% air at 37°C. Cells were treated with 10μg/ml LPS or LPS plus different reagents for indicated times.

### Transfection of EGFR siRNA

Sequence of EGFR siRNA was desrcibed by previous research [7]. Cells were plated at 0.5 × 10^6^ cells per 35-mm dish to reach 50%–60% confluence after overnight growth. The cells were then transfected with 50 nm EGFR siRNA or control siRNA using Lipofectamin 2000 (Invitrogen, Carlsbad, CA). The cells were subjected to experimental treatment after 24 hours of transfection.

### Septic AKI models

The mice (male, aged 10–12 weeks) were injected intraperitoneally with LPS (from Escherichia coli O111:B4, 10 mg/kg; Santa Cruz Biotechnology, Santa Cruz, CA). Gefitinib at a dosage of 100 mg/kg was given intraperitoneally at 1h after LPS. The control group was administered with saline. Renal tissues were harvested for various biochemical and morphologic studies at 24h hours after LPS. Cecal ligation and puncture (CLP) induced AKI was established in male Wa-2 mice (maintained on a C57BL/6 JeixC3H/HeSnJ background) and their littermates aged 10–12 weeks as desrcibed by previous [13, 36–38]. Briefly, cecum was tightly ligated with a 4-0 silk suture at 1.5 cm from the tip and punctured twice with a 21-gauge needle. Approximately a 1-mm column of fecal material was expressed by gently squeezing. The cecum was separated but neither ligated nor punctured in sham-operated mice. After surgery, 1 ml of prewarmed saline was injected by intraperitoneally in all mice, and then put them in individual cages on a heating pad. Animal experiments were performed in accordance with the set by the Institutional Committee for the Care and Use of Laboratory Animals of Second Xiangya Hospital, China. C57BL/6 mice were housed on a 12-hour light/dark cycle, and were allowed free access to food and water.

### Microarray

Total RNA was isolated from kidney cortical tissues for reverse tranSrciption using the RNasey Mini Kit (Qiagen p/n 74104). The synthesized cRNAs were fragmented and biotin-labeled using the Quick Amp Labeling Kit, One-Color (Agilent p/n 5190-0442). The labeled cRNAs were then hybridized onto the Affymetrix Mouse Gene 2.0ST Array using Agilent Gene Expression Hybridization Kit (Agilent p/n 5188-5242). After 17 hours of hybridization, the arrays were washed and stained using Affymetrix GeneChip Fluidics Station 450 Systems. The stained arrays were scanned with an Agilent Microarray Scanner (Agilent p/n G2565BA).

### Analysis of renal function, histology, and TUNEL assay

Renal failure or loss of renal function was indicated by serum creatinine and blood urea nitrogen (BUN) using commercial kits as previously desrcibed [39–43]. For histology, kidney tissues were fixed with 4% paraformaldehyde for paraffin embedding and H&E staining. Histologic changes in the cortex and the outer stripe of the outer medulla (OSOM) were scored by the percentage of renal tubules with loss of brush border, cellular necrosis, cast formation, tubule dilation, and vacuolization (0, no damage; 1, <25%; 2, 25%–50%; 3, 50%–75%; 4, >75%). The *In Situ* Cell Death Detection Kit from Roche Applied Science was using for TUNEL assay. For quantification, we randomly selected 10–20 fields from each tissue section to count the TUNEL-positive cells per millimeter [39].

### Immunohistochemistry and immunoblot analysis

Immunohistochemical Analyses were performed using anti-macrophage and active caspase3 according to the previous protocol [39]. The total number of macrophage in tubulointerstitium was quantiied by counting the number of stained cells per field as previous desrcibed [44]. For immunoblot analysis, briefly, cells or kidney tissues were treated with a lysis buffer (Sigma-Aldrich) containing phosphatase inhibitors (Calbiochem). Equal amounts of proteins were loaded in each well for electrophoresis using SDS-CPAGE gel, blotting, and antibody exposure according to standard procedures.

### Real-time polymerase chain reaction

Real-time quantitative reverse transrciptase PCR amplifications were performed in 20μL reactions as desrcibed previously [44]. The primer sets used for various genes were as follows: ICAM-1 (intercellular adhesion molecule-1): forward 5’-CTTCCAGCTACCATCCCAAA-3’, reverse 5’-CTTCAGAGGCAGGAAACAGG-3’; TNF-α: forward 5’-TAGCCAGGAGGGAGAACAGA -3’, reverse 5’-TTTTCTGGAGGGAGATGTGG-3’; TGF-β1: forward 5’-TGAGTGGCTGTCTT TTGACG-3’, reverse 5’- AGCCCTGTATTCCGTCTCCT-3’; IL-1β: forward 5’-CCCAAGCAA TACCCAAAGAA-3’, reverse 5’-GCTTGTGCTCTGCTTGTGAG -3’; GADPH: forward 5’- TG CTGAGTATGTCGTGGAGTCTA-3’, reverse 5’-AGTGGGAGTTGCTGTTG AAATC-3’.

### Statistical analysis

Qualitative data, including immunoblots, RT-PCR and tissue histology images, are representatives of at least three experiments. Data were expressed as mean±SEM (standard error of the mean). One-way ANOVA, followed by the Tukey’s post hoc test, was used to compare multiple treatment groups. Two-way ANOVA was used to assess the statistical significance of the differences between multiple treatment groups at different time points. Statistical significance was set at *P<0.05*.

### SUPPLEMENTARY MATERIALS FIGURES AND TABLES





## Abbreviations

AKI: acute kidney injury
I/R: ischemia-reperfusion
HK-2: human proximal tubular epithelial cells
ICU: intensive care unit
c-Src: proto-oncogene tyrosine-protein kinase Src
EGFR: epidermal growth factor receptor
ALI: acute lung injury
OSOM: outer medulla
STAT: signal transduction and activator of transcription
TLR4: Toll Like Receptor 4
CLP: cecal ligation and puncture
HIPK-2: the expression of homeodomain interacting protein kinase 2
ATF3: activating transrciption factor 3
NLRP3: NACHT, LRR and PYD domains-containing protein 3
ROCK2: Rho-associated protein kinase 2
KEGG: Kyoto Encyclopedia of Genes and Genomes

## References

[1] Uchino S, Kellum JA, Bellomo R, Doig GS, Morimatsu H, Morgera S, Schetz M, Tan I, Bouman C, Macedo E, Gibney N, Tolwani A, Ronco C (2005). Acute renal failure in critically ill patients: a multinational, multicenter study. JAMA.

[2] Schrier RW, Wang W (2004). Acute renal failure and sepsis. N Engl J Med.

[3] Waikar SS, Liu KD, Chertow GM (2008). Diagnosis, epidemiology and outcomes of acute kidney injury. Clin J Am Soc Nephrol.

[4] Qian Y, Peng K, Qiu C, Skibba M, Huang Y, Xu Z, Zhang Y, Hu J, Liang D, Zou C, Wang Y, Liang G (2016). Novel epidermal growth factor receptor inhibitor attenuates angiotensin II-induced kidney fibrosis. J Pharmacol Exp Ther.

[5] Liu N, Wang L, Yang T, Xiong C, Xu L, Shi Y, Bao W, Chin YE, Cheng SB, Yan H, Qiu A, Zhuang S (2015). EGF receptor inhibition alleviates hyperuricemic nephropathy. J Am Soc Nephrol.

[6] Liu N, Guo JK, Pang M, Tolbert E, Ponnusamy M, Gong R, Bayliss G, Dworkin LD, Yan H, Zhuang S (2012). Genetic or pharmacologic blockade of EGFR inhibits renal fibrosis. J Am Soc Nephrol.

[7] Chen J, Chen JK, Nagai K, Plieth D, Tan M, Lee TC, Threadgill DW, Neilson EG, Harris RC (2012). EGFR signaling promotes TGFbeta-dependent renal fibrosis. J Am Soc Nephrol.

[8] Tang J, Liu N, Tolbert E, Ponnusamy M, Ma L, Gong R, Bayliss G, Yan H, Zhuang S (2013). Sustained activation of EGFR triggers renal fibrogenesis after acute kidney injury. Am J Pathol.

[9] Chattopadhyay S, Veleeparambil M, Poddar D, Abdulkhalek S, Bandyopadhyay SK, Fensterl V, Sen GC (2015). EGFR kinase activity is required for TLR4 signaling and the septic shock response. EMBO Rep.

[10] Smith JA, Stallons LJ, Schnellmann RG (2014). Renal cortical hexokinase and pentose phosphate pathway activation through the EGFR/Akt signaling pathway in endotoxin-induced acute kidney injury. Am J Physiol Renal Physiol.

[11] He S, Liu N, Bayliss G, Zhuang S (2013). EGFR activity is required for renal tubular cell dedifferentiation and proliferation in a murine model of folic acid-induced acute kidney injury. Am J Physiol Renal Physiol.

[12] De S, Zhou H, DeSantis D, Croniger CM, Li X, Stark GR (2015). Erlotinib protects against LPS-induced endotoxicity because TLR4 needs EGFR to signal. Proc Natl Acad Sci U S A.

[13] Miyaji T, Hu X, Yuen PS, Muramatsu Y, Iyer S, Hewitt SM, Star RA (2003). Ethyl pyruvate decreases sepsis-induced acute renal failure and multiple organ damage in aged mice. Kidney Int.

[14] Finzi L, Shao MX, Paye F, Housset C, Nadel JA (2009). Lipopolysaccharide initiates a positive feedback of epidermal growth factor receptor signaling by prostaglandin E2 in human biliary carcinoma cells. J Immunol.

[15] McElroy SJ, Hobbs S, Kallen M, Tejera N, Rosen MJ, Grishin A, Matta P, Schneider C, Upperman J, Ford H, Polk DB, Weitkamp JH (2012). Transactivation of EGFR by LPS induces COX-2 expression in enterocytes. PLoS One.

[16] Thuringer D, Hammann A, Benikhlef N, Fourmaux E, Bouchot A, Wettstein G, Solary E, Garrido C (2011). Transactivation of the epidermal growth factor receptor by heat shock protein 90 via Toll-like receptor 4 contributes to the migration of glioblastoma cells. J Biol Chem.

[17] Howe CL (2005). Modeling the signaling endosome hypothesis: why a drive to the nucleus is better than a (random) walk. Theor Biol Med Model.

[18] Meloche S, Pouyssegur J (2007). The ERK1/2 mitogen-activated protein kinase pathway as a master regulator of the G1- to S-phase transition. Oncogene.

[19] Albuszies G, Vogt J, Wachter U, Thiemermann C, Leverve XM, Weber S, Georgieff M, Radermacher P, Barth E (2007). The effect of iNOS deletion on hepatic gluconeogenesis in hyperdynamic murine septic shock. Intensive Care Med.

[20] Matsuyama M, Yoshimura R, Hase T, Kawahito Y, Sano H, Nakatani T (2005). Study of cyclooxygenase-2 in renal ischemia-reperfusion injury. Transplant Proc.

[21] Chen J, Shetty S, Zhang P, Gao R, Hu Y, Wang S, Li Z, Fu J (2014). Aspirin-triggered resolvin D1 down-regulates inflammatory responses and protects against endotoxin-induced acute kidney injury. Toxicol Appl Pharmacol.

[22] Zarjou A, Agarwal A (2011). Sepsis and acute kidney injury. J Am Soc Nephrol.

[23] Kuper C, Beck FX, Neuhofer W (2012). Toll-like receptor 4 activates NF-kappaB and MAP kinase pathways to regulate expression of proinflammatory COX-2 in renal medullary collecting duct cells. Am J Physiol Renal Physiol.

[24] Lu YC, Yeh WC, Ohashi PS (2008). LPS/TLR4 signal transduction pathway. Cytokine.

[25] Lin M, Tang SC (2014). Toll-like receptors: sensing and reacting to diabetic injury in the kidney. Nephrol Dial Transplant.

[26] Darnell JE, Kerr IM, Stark GR (1994). Jak-STAT pathways and transcriptional activation in response to IFNs and other extracellular signaling proteins. Science.

[27] Matsukawa A (2007). STAT proteins in innate immunity during sepsis: lessons from gene knockout mice. Acta Med Okayama.

[28] Zhang M, Wang X, Wang X, Hou X, Teng P, Jiang Y, Zhang L, Yang X, Tian J, Li G, Cao J, Xu H, Li Y (2013). Oxymatrine protects against myocardial injury via inhibition of JAK2/STAT3 signaling in rat septic shock. Mol Med Rep.

[29] Gyurkovska V, Ivanovska N (2015). Tyrosine kinase inhibitor tyrphostin AG490 reduces liver injury in LPS-induced shock. Eur J Pharmacol.

[30] Wook Choi D, Yong Choi C (2014). HIPK2 modification code for cell death and survival. Mol Cell Oncol.

[31] Zhao Q, Yang X, Cai D, Ye L, Hou Y, Zhang L, Cheng J, Shen Y, Wang K, Bai Y (2016). Echinacoside protects against MPP(+)-induced neuronal apoptosis via ROS/ATF3/CHOP pathway regulation. Neurosci Bull.

[32] Guo Y, Song Z, Zhou M, Yang Y, Zhao Y, Liu B, Zhang X (2017). Infiltrating macrophages in diabetic nephropathy promote podocytes apoptosis via TNF-alpha-ROS-p38MAPK pathway. Oncotarget.

[33] Gentle ME, Shi S, Daehn I, Zhang T, Qi H, Yu L, D'Agati VD, Schlondorff DO, Bottinger EP (2013). Epithelial cell TGFbeta signaling induces acute tubular injury and interstitial inflammation. J Am Soc Nephrol.

[34] Cao Y, Fei D, Chen M, Sun M, Xu J, Kang K, Jiang L, Zhao M (2015). Role of the nucleotide-binding domain-like receptor protein 3 inflammasome in acute kidney injury. FEBS J.

[35] Kentrup D, Reuter S, Schnockel U, Grabner A, Edemir B, Pavenstadt H, Schober O, Schafers M, Schlatter E, Bussemaker E (2011). Hydroxyfasudil-mediated inhibition of ROCK1 and ROCK2 improves kidney function in rat renal acute ischemia-reperfusion injury. PLoS One.

[36] Yasuda H, Yuen PS, Hu X, Zhou H, Star RA (2006). Simvastatin improves sepsis-induced mortality and acute kidney injury via renal vascular effects. Kidney Int.

[37] Wu L, Gokden N, Mayeux PR (2007). Evidence for the role of reactive nitrogen species in polymicrobial sepsis-induced renal peritubular capillary dysfunction and tubular injury. J Am Soc Nephrol.

[38] Wang Z, Sims CR, Patil NK, Gokden N, Mayeux PR (2015). Pharmacologic targeting of sphingosine-1-phosphate receptor 1 improves the renal microcirculation during sepsis in the mouse. J Pharmacol Exp Ther.

[39] Zhang D, Liu Y, Wei Q, Huo Y, Liu K, Liu F, Dong Z (2014). Tubular p53 regulates multiple genes to mediate AKI. J Am Soc Nephrol.

[40] Zhang D, Li Y, Liu Y, Xiang X, Dong Z (2013). Paclitaxel ameliorates lipopolysaccharide-induced kidney injury by binding myeloid differentiation protein-2 to block Toll-like receptor 4-mediated nuclear factor-kappaB activation and cytokine production. J Pharmacol Exp Ther.

[41] Peng J, Li X, Zhang D, Chen JK, Su Y, Smith SB, Dong Z (2015). Hyperglycemia, p53, and mitochondrial pathway of apoptosis are involved in the susceptibility of diabetic models to ischemic acute kidney injury. Kidney Int.

[42] Chen J, Wang J, Li H, Wang S, Xiang X, Zhang D (2016). p53 activates miR-192-5p to mediate vancomycin induced AKI. Sci Rep.

[43] Zhang D, Pan J, Xiang X, Liu Y, Dong G, Livingston MJ, Chen JK, Yin XM, Dong Z (2017). Protein kinase Cdelta suppresses autophagy to induce kidney cell apoptosis in cisplatin nephrotoxicity. J Am Soc Nephrol.

[44] Zhang L, Xu X, Yang R, Chen J, Wang S, Yang J, Xiang X, He Z, Zhao Y, Dong Z, Zhang D (2015). Paclitaxel attenuates renal interstitial fibroblast activation and interstitial fibrosis by inhibiting STAT3 signaling. Drug Des Devel Ther.

