# Supplementary material for: Genetic or pharmacologic inhibition of EGFR ameliorates sepsis-induced AKI
Source: Oncotarget. 2017 Sep 23;8(53):91577–92. doi: 10.18632/oncotarget.21244 (PMC5710948; doi:10.18632/oncotarget.21244)
Supplement: Supplementary file 1 [file oncotarget-08-91577-s001.pdf]

Genetic or pharmacologic inhibition of EGFR ameliorates sepsis-induced AKI

SUPPLEMENTARY MATERIALS

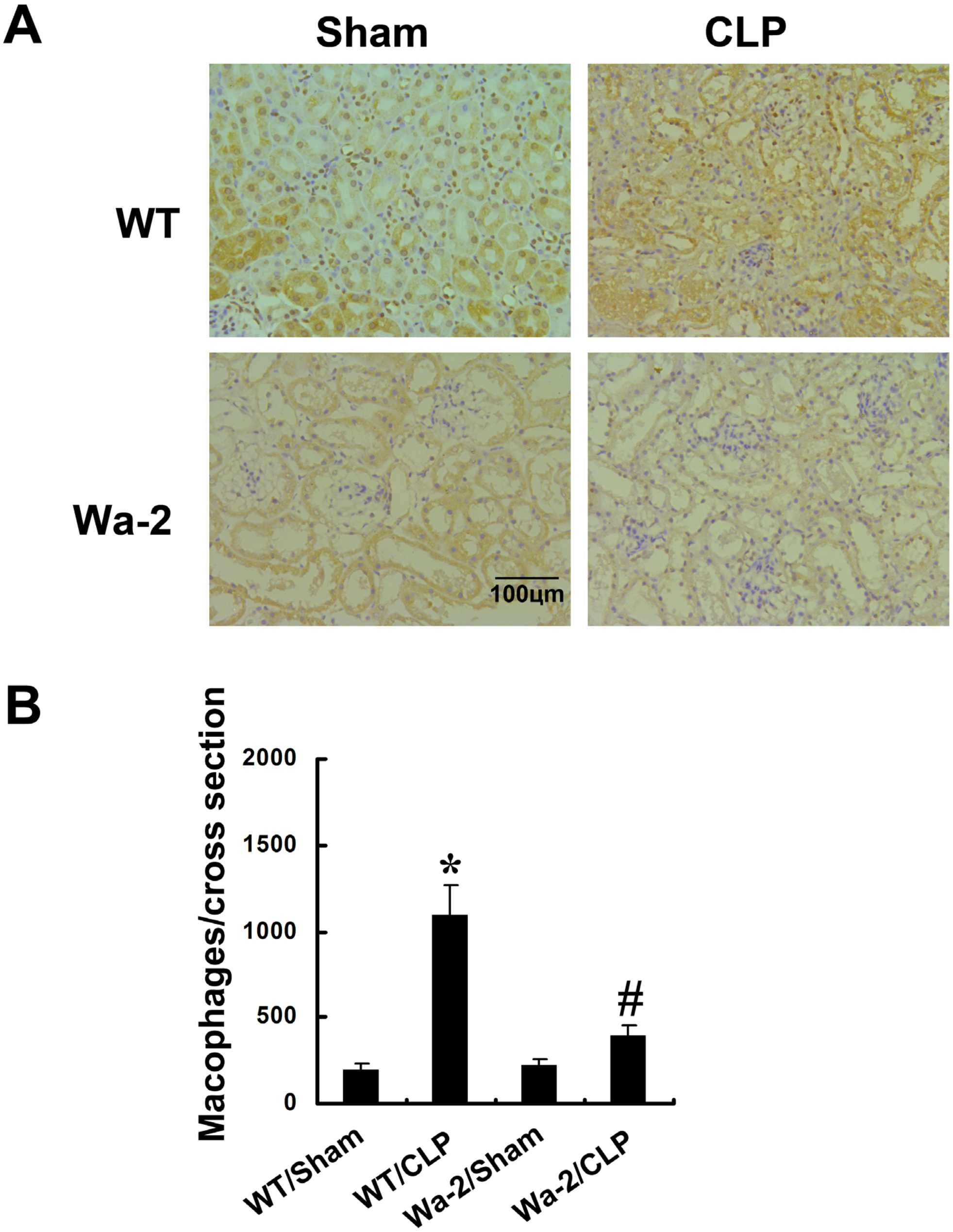

Supplementary Figure 1: Wa-2 inhibited infiltration of macrophages in CLP induced AKI model. Wild-type and Wa-2 littermate mice were subjected to CLP. Sham-operated mice were used as a control. Kidney tissues were collected at 18 h after treatment. **(A)** Representative macrophage immunohistochemistry from kidney cortical tissues of different groups at 18h (original magnification, ×400). **(B)** Quantitation of macrophage-positive cells per total cross-sectional area was performed from each group (n=8). Data are presented as mean ± SEM; * *P<0.05* WT/Sham group; #*P<0.05* versus CLP group. Data are representative of at least four separate experiments.

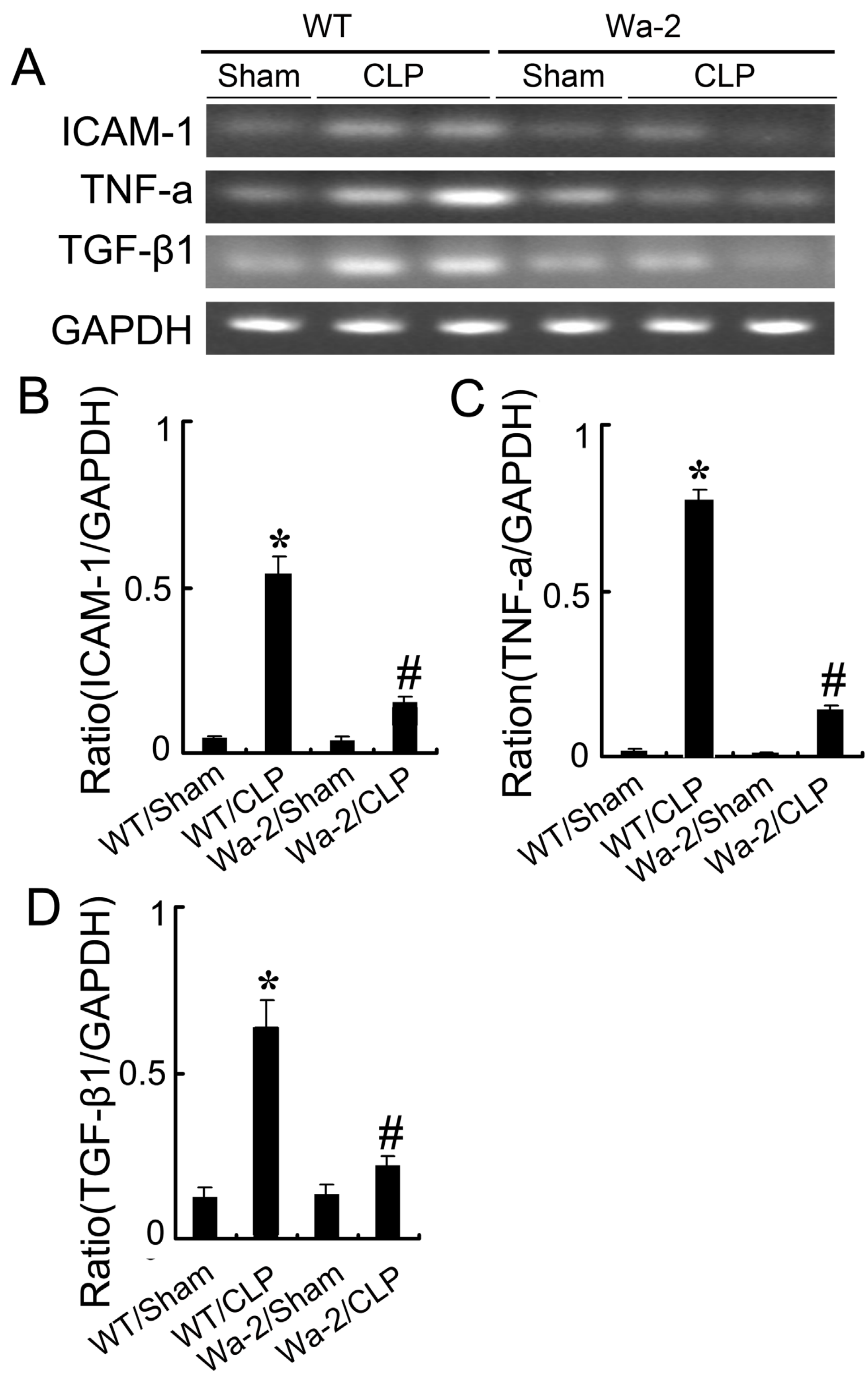

Supplementary Figure 2: The expression of ICAM-1, TNF-α, and TGF-β1 was suppressed in Wa-2 CLP mice. Wild-type and Wa-2 littermate mice were subjected to CLP. Sham-operated mice were used as a control. Kidney tissues were collected at 18 h after treatment. **(A)** RT-PCR analysis of expression of ICAM-1, TNF-α, and TGF-β1. **(B-D)** Densitometric analysis of the ICAM-1/GAPDH, TNF-α/GAPDH, and TGF-β1/GAPDH ratio. Data are presented as mean ± SEM (n=8); * *P<0.05* WT/Sham group; # *P<0.05* versus CLP group. Data are representative of at least four separate experiments.

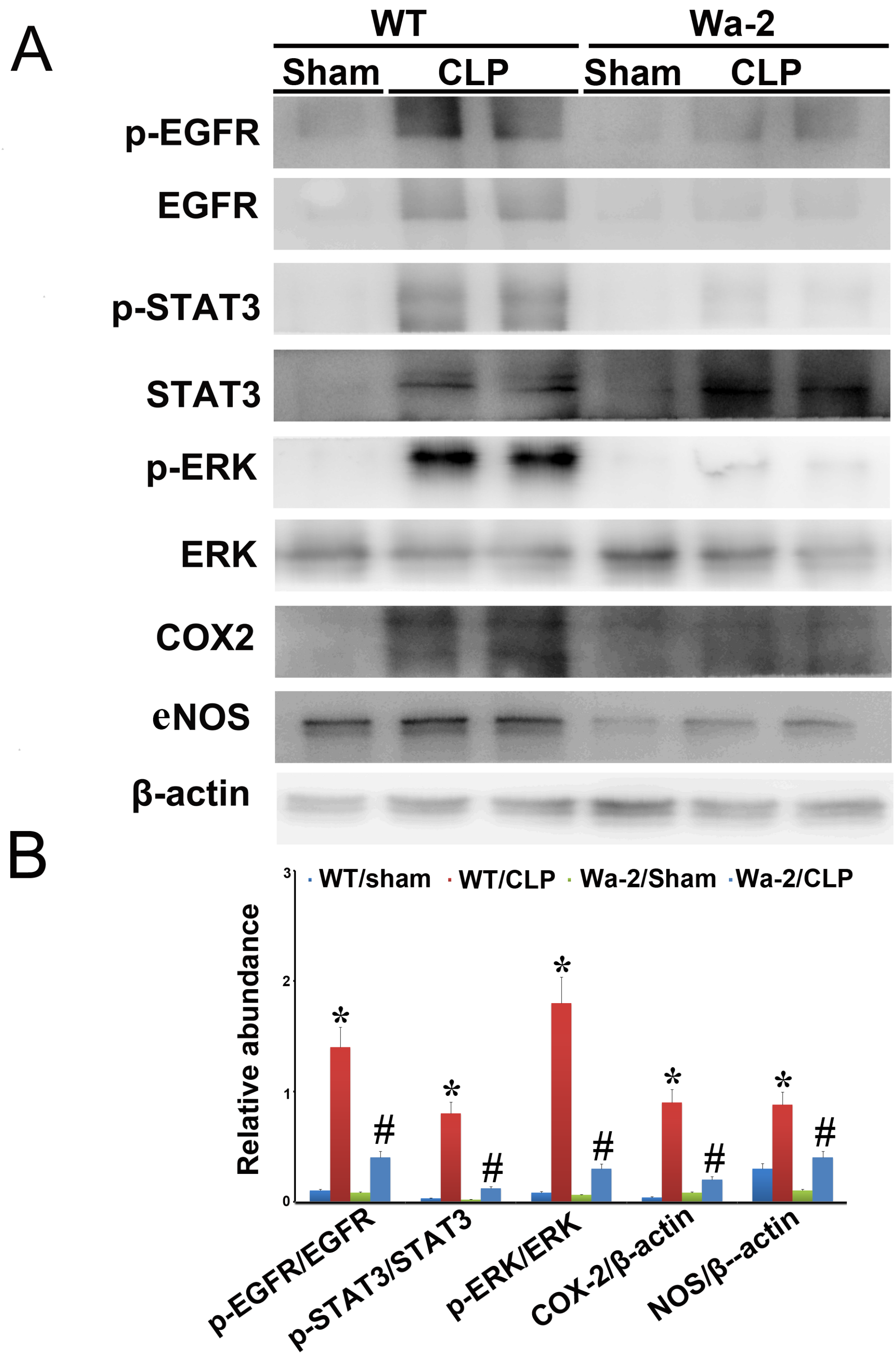

Supplementary Figure 3: EGFR activation is required for phosphorylation of ERK1/2 and STAT3, and expression of COX-2 and eNOS in CLP induced AKI model. Wild-type and Wa-2 littermate mice were subjected to CLP. Sham-operated mice were used as a control. Kidney tissues were collected at 18 h after treatment. **(A)** Immunoblot analysis of expression and activation of EGFR, STAT3, and ERK1/2, and expression of COX-2 and eNOS. **(B)** Densitometric analysis of the p-EGFR/EGFR, p-ERK1/2/ERK1/2, p-STAT3/STAT3, COX-2/β-actin, and eNOS/β-actin ratio. Data are presented as mean ± SEM (n=8); * *P<0.05* WT/Sham group; #*P<0.05* versus CLP group. Data are representative of at least four separate experiments.

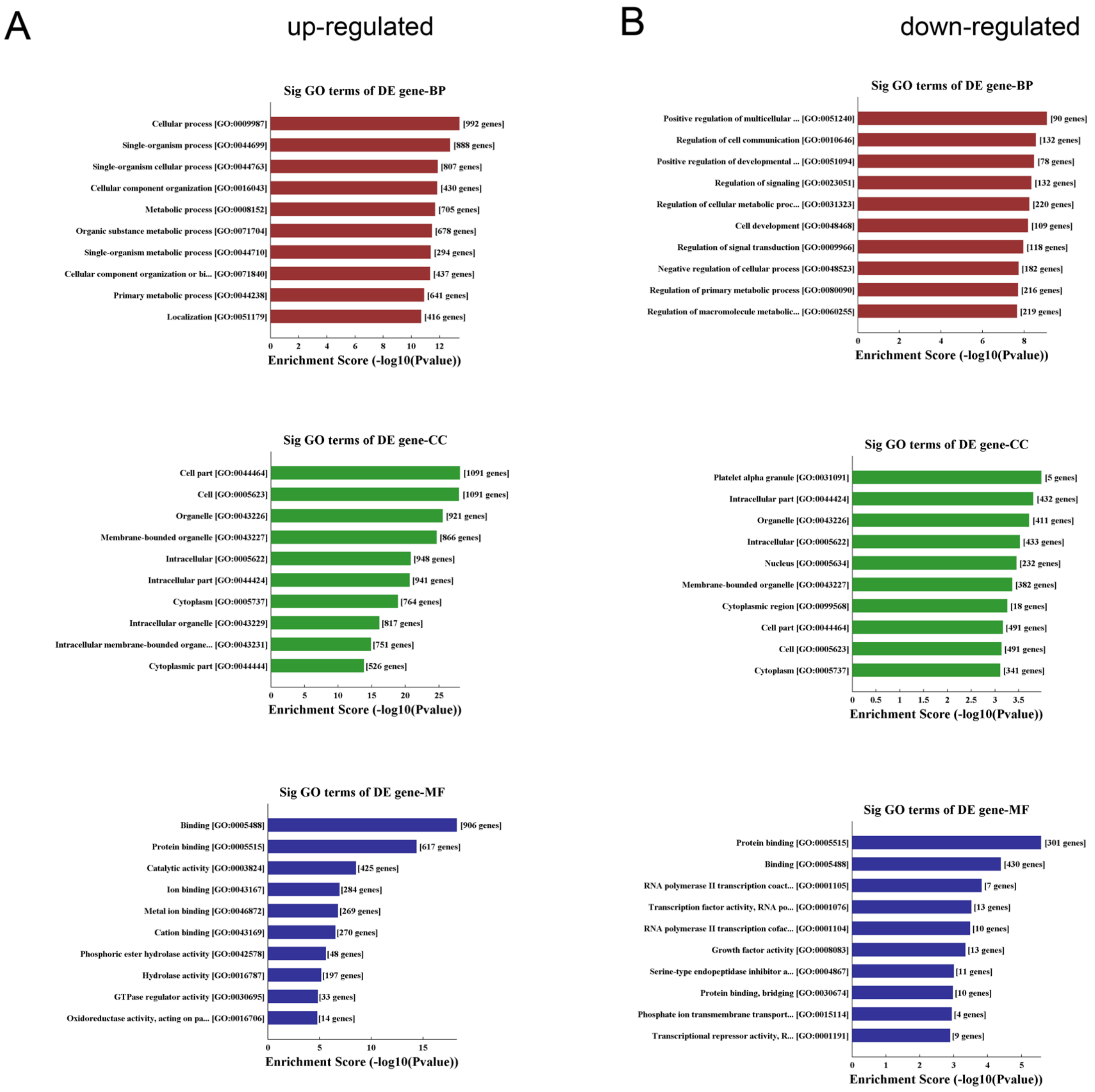

Supplementary Figure 4: The Gene ontology (GO) analysis of the genes in kidney tissues of Wa-2 with CLP versus Wild-type with CLP. **(A)** Go up regulated. **(B)** Down regulated. The Y-axis indicates the number of a specific GO term of genes in the corresponding main category, and various GO terms in a category. The X-axis represents P-value of the significant of GO terms enrichment in the DE genes. The lower P-value, the more significant GO term (*P<0.05* was recommended as the cut-off value).

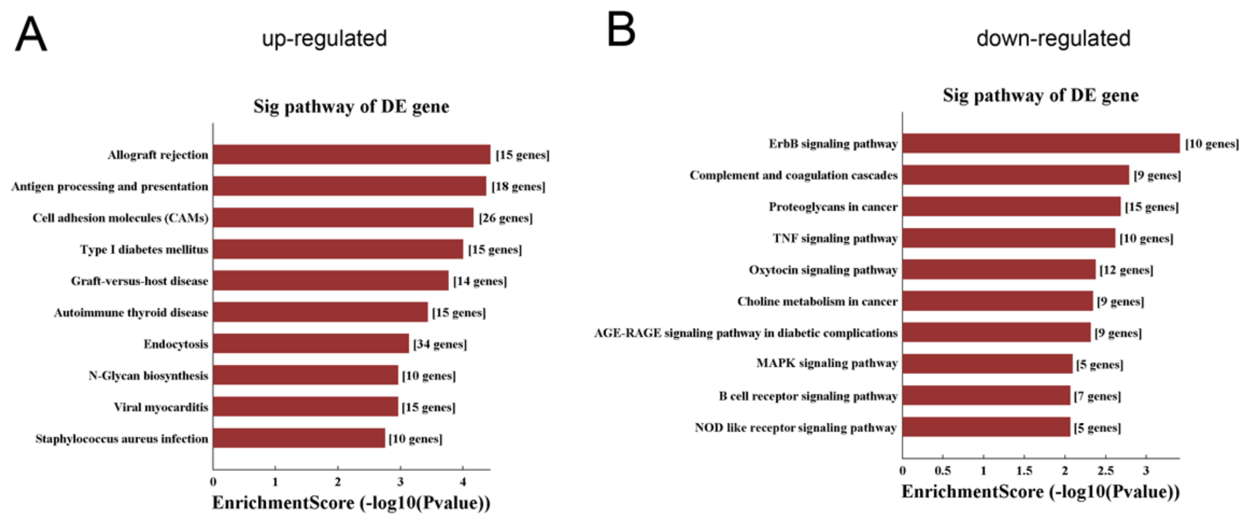

Supplementary Figure 5: The expression of HIPK-2, ATF3, NLRP3, and ROCK2 is suppressed in CLP induced AKI model. Wild-type and Wa-2 littermate mice were subjected to CLP. Sham-operated mice were used as a control. Kidney tissues were collected at 18 h after treatment. **(A)** Immunoblot analysis of expression of HIPK-2, ATF3, NLRP3, and ROCK2. **(B)** Densitometric analysis of HIPK-2/β-actin, ATF3/β-actin, NLRP3/β-actin, and ROCK2/β-actin ratio. Data are presented as mean ± SEM (n=8); * $P<0.05$ WT/Sham group; # $P<0.05$ versus CLP group. Data are representative of at least four separate experiments.

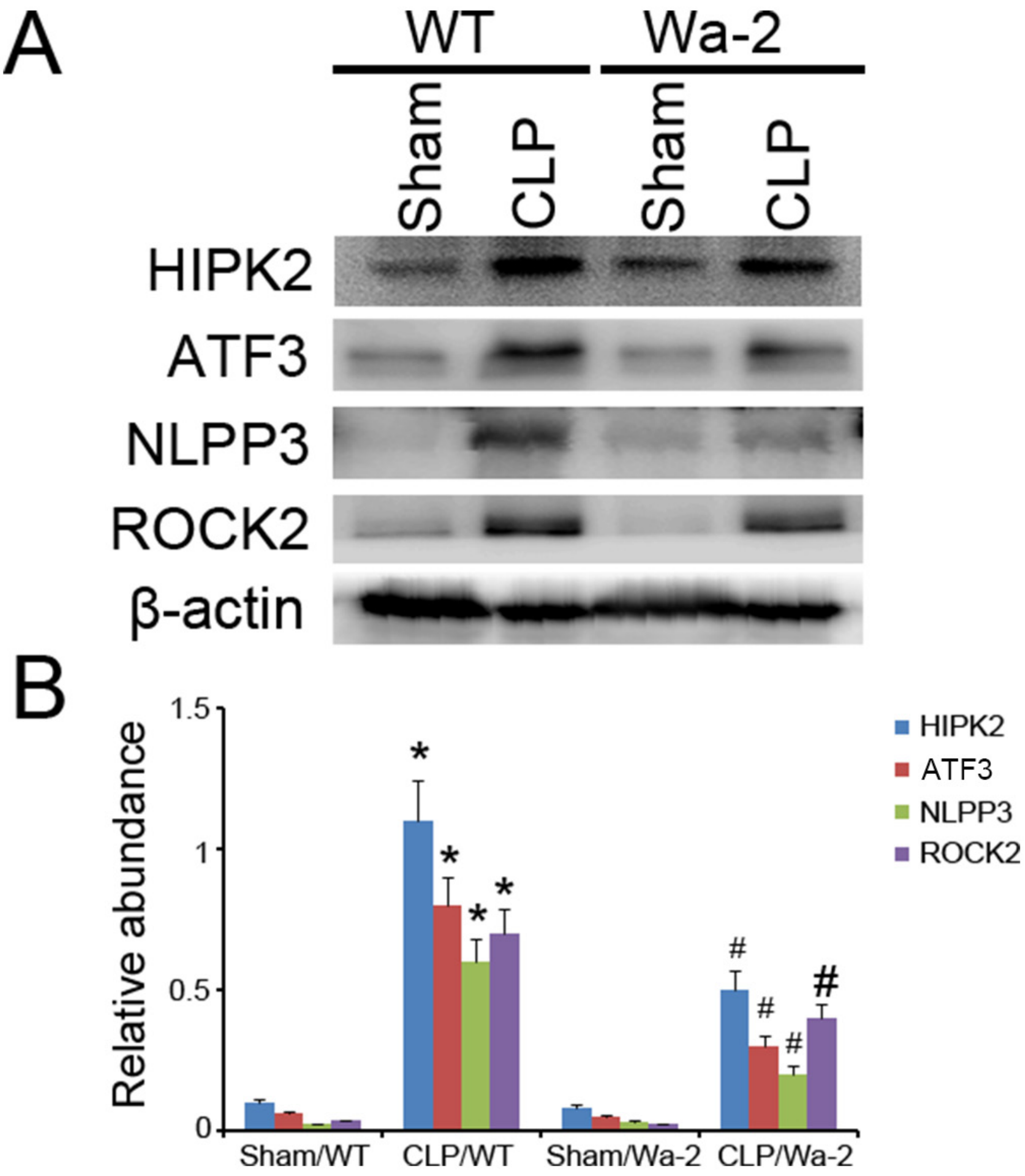

Supplementary Figure 6: The signaling pathway of differentially expressed up regulated **(A)** and down regulated **(B)** genes in kidney tissues of Wa-2 with CLP versus Wild-type with CLP. The Y-axis indicates the signaling pathway and the genes number of in specific signaling pathway. The X-axis represents P-value of the significant of GO terms enrichment in the DE genes. The lower P-value, the more significant GO term (*P<0.05* was recommended as the cut-off value).

Supplementary Table 1: CLP-induced AKI with EGFR WT VS Sham UP regulate genes

See Supplementrary File 1

Supplementary Table 2: CLP-induced AKI with EGFR WT VS Sham down regulate genes

See Supplementrary File 1

Supplementary Table 3: CLP-induced AKI with Wa-2 VS Sham with CLP-induced AKI EGFR WT up regulate genes

See Supplementrary File 1

Supplementary Table 4: CLP-induced AKI with Wa-2 VS Sham with CLP-induced AKI EGFR WT down regulate genes

See Supplementrary File 1

Supplementary Table 5: Gene ontology (GO) enrichment analysis of the 995 down-regulated genes in Wa-2 mice with CLP treatment

Go Term	Coun	P-Value	Down-regulated genes
Cell death	74	0.007	IL6//JUN//SFN//PMAIP1//SP110//AKT2//BIRC3//DFFB//FAP//HIPK2//CARD14//MEF2A//PRKCB//SGMS1//SHB//ELMO3//MAPK3//CLIC4//MAP3K9//DFNA5//KRT20//PLSCR3//GULP1//RPS6KB1//F3//NLRP3//PAFAH2//BCL3//MUC4//HIF1A//IGF1//KRT18//SLC9A1//SMARCA4//TFAP2B//CAMK1D//SYCP2//MAD2L1//FIGNL1//ROCK2//CAV1//QK//EGR1//IRAK2//PTGIS//RASSF3//RASSF2//GDF15//ECT2//REST//SPDEF//GDF5//KRAS//MT1//NR4A2//VSTM2L//XRCC2//OPTN//CDK5R1//RNF4//RAD18//TXNDC12//CECR2//CSF3//PTPRZ1//FGB//FGA//FGG//BMPR1B//FBXO18//PTPN2//ATF3//TLE1//CAMK2B
JAK-STAT cascade	12	0.004	LIF//HPX//PTPN2//IL6//NF2//IGF1//CAV1//CISH//FLRT3//LRRC15//STRA6
ERK1 and ERK2 cascade	18	0.001	ATF3//CAV1//FBLN1//IGF1//LIF//PTPN2//FGB//FGA//FGF18//GCNT2//IL6//JUN//CCL17//NDRG4//MAPK3//C1QTNF3//FGG//OXTR
positive regulation of MAPK cascade	23	0.008	KISS1R//MAP3K6//DRD4//FGF1//IGF1//FGF18//KRAS//HIPK2//RASSF2//DIXDC1//FGB//FGA//GCNT2//IL6//JUN//CCL17//NDRG4//MAPK3//C1QTNF3//FGG//KSR1//LIF//C1QTNF1
macrophage differentiation	4	0.014	PTPN2//ZBTB46//LIF//TRIB1
I-kappaB kinase/NF-kappaB signaling	12	0.032	NLRP3//C1QTNF3//CPNE1//ECT2//PRKCB//SLC20A1//MAP3K14//TLE1//OPTN//IRAK2//BCL3//ROCK2
Nitric oxide biosynthetic process	5	0.042	CAV1//PTGIS//IGF1//AKT2//IL6
