# Supplementary material for: Genetic or pharmacologic inhibition of EGFR ameliorates sepsis-induced AKI
Source: Oncotarget. 2017 Sep 23;8(53):91577–92. doi: 10.18632/oncotarget.21244 (PMC5710948; doi:10.18632/oncotarget.21244)
Supplement: Supplementary file 2 [file oncotarget-08-91577-s002.docx]

**Supplementary Table 1: CLP-induced AKI with EGFR WT VS Sham UP regulate genes**

| **Fold Change** | **Regulation** | **GenbankAccession** | **GenomicCoordinates** | **GeneSymbol** |
| --- | --- | --- | --- | --- |
| 2.0522038 | up | NM_009532 | chr7:25358268-25358327 | Xrcc1 |
| 3.5619296 | up | NR_027955 | chr3:65331565-65331506 | 4931440P22Rik |
| 3.9881426 | up | NM_001198984 | chr18:60975715-60975656 | Tcof1 |
| 4.0400331 | up | AK020900 | chr19:40373983-40374041 | A930028N01Rik |
| 24.3979495 | up | NM_026929 | chr2:119179990-119180049 | Chac1 |
| 2.2797316 | up | NM_009949 | chr4:107576650-107576591 | Cpt2 |
| 7.2143259 | up | NM_172693 | chr4:47135796-47135855 | Galnt12 |
| 6.2646183 | up | AK047890 | chr19:16318217-16318276 | LOC553096 |
| 5.2960675 | up | NM_023182 | chr8:108456554-108456495 | Ctrl |
| 2.2348588 | up | NM_015764 | chr12:16678514-16678455 | Greb1 |
| 3.6084741 | up | NM_001197024 | chr17:25346523-25346581 | Unkl |
| 2.7017852 | up | NM_009370 | chr4:47427736-47427795 | Tgfbr1 |
| 6.0395623 | up | NM_001011535 | chr2:88286192-88286176 | Olfr1182 |
| 3.5426624 | up |  | chr5:135113276-135113217 |  |
| 8.4242719 | up | NM_019937 | chr3:65751624-65750823 | Ccnl1 |
| 7.138899 | up | NM_008872 | chr8:23892531-23892590 | Plat |
| 3.5436094 | up |  | chr11:62180505-62180446 | Ncor1 |
| 8.4840963 | up | NM_001081141 | chr4:46737186-46731752 | Gabbr2 |
| 8.7710879 | up | NM_010846 | chr16:97668703-97668644 | Mx1 |
| 2.212803 | up | NM_001242363 | chr7:112527208-112527149 | Fam160a2 |
| 2.9796358 | up | NM_001142731 | chr18:15127254-15127195 | Kctd1 |
| 2.1898226 | up | NM_029921 | chr6:38065689-38065748 | Tmem213 |
| 3.2706265 | up | NM_001101038 | chr18:78240412-78240353 | Siglec15 |
| 4.1974682 | up | AK052374 | chr6:141448762-141448821 | Pde3a |
| 5.3435496 | up | AK032614 | chr10:13407074-13407015 | 6430706H07Rik |
| 6.4024634 | up | NM_011719 | chr11:103588941-103588882 | Wnt9b |
| 3.5008858 | up |  | chr2:177674032-177673973 |  |
| 2.4029216 | up |  | chr5:145976694-145976753 |  |
| 5.0395765 | up | AK045831 | chr5:32776516-32776575 | E230008O15Rik |
| 2.708665 | up | NM_009354 | chr13:73786429-73786488 | Tert |
| 2.4414941 | up | XM_003946335 | chr4:140978740-140978799 | LOC101056474 |
| 5.2864748 | up | NM_027560 | chr8:73359124-73359065 | Arrdc2 |
| 2.9132954 | up | NM_001242389 | chr17:57402779-57402838 | Trip10 |
| 4.3003945 | up | NM_028228 | chr14:64538435-64538494 | Pinx1 |
| 2.0320592 | up | NM_015759 | chr13:49358570-49358511 | Fgd3 |
| 2.7717997 | up |  | chr13:046793490-046793431 |  |
| 3.1416686 | up | NM_020559 | chr9:106136343-106136284 | Alas1 |
| 2.7278173 | up | NM_023125 | chr16:23081700-23081759 | Kng1 |
| 5.2006336 | up | NM_010806 | chr17:13989328-13989387 | Mllt4 |
| 7.6037047 | up | NM_001081431 | chr5:146052279-146052338 | Zscan25 |
| 3.5548446 | up | NM_183149 | chr17:24818901-24818960 | Zfp598 |
| 3.1467117 | up | NM_001081750 | chr5:125365082-125365141 | Zfp664 |
| 2.1327842 | up | NM_025291 | chr18:36827074-36827015 | Sra1 |
| 2.1983468 | up |  | chr4:115205300-115205359 |  |
| 194.5421558 | up | NM_001004153 | chr7:3155339-3155280 | AU018091 |
| 4.6976541 | up | NM_172339 | chr2:26218353-26218294 | Snapc4 |
| 3.0552885 | up | NM_001011787 | chr2:111785008-111784949 | Olfr1307 |
| 3.099486 | up | NM_009366 | chr14:76907276-76907335 | Tsc22d1 |
| 3.0441005 | up | NM_011488 | chr11:100746373-100746432 | Stat5a |
| 14.7436927 | up | NM_008103 | chr5:017758450-017758509 | Gcm1 |
| 2.7379802 | up | NM_011236 | chr6:119872539-119872598 | Rad52 |
| 5.3232757 | up | NM_023799 | chr19:45842426-45842367 | Mgea5 |
| 2.3449822 | up | NM_177354 | chr6:016809027-016808968 | Vash1 |
| 4.2912061 | up | AK029403 | chr12:109355344-109355285 | Setd3 |
| 7.214765 | up | AK141715 | chr13:114765014-114765073 | 6720427H10Rik |
| 2.6948267 | up | NM_017479 | chr14:22490036-22490095 | Kat6b |
| 5.4336276 | up | NM_023516 | chr6:29225141-29225200 | Hilpda |
| 2.0244682 | up | NM_022305 | chr4:40756815-40756756 | B4galt1 |
| 2.0648754 | up | NR_033554 | chr17:24547214-24547069 | D330041H03Rik |
| 2.379926 | up | NM_175375 | chr18:36817732-36817791 | Ankhd1 |
| 3.3139807 | up | NR_038130 | chr12:100399548-100399489 | 4930474N09Rik |
| 2.1543356 | up | NM_001146023 | chr7:29937592-29937533 | Fam98c |
| 21.0771895 | up | NM_001081361 | chr1:186611226-186611167 | Marc1 |
| 11.3004914 | up | NM_001170935 | chr10:79324535-79324476 | BC005764 |
| 65.4858999 | up | NM_001170787 | chr9:9673903-9673844 | Cntn5 |
| 2.9037857 | up | NM_029478 | chr11:86398313-86398254 | Vmp1 |
| 4.2543193 | up | NM_001033159 | chr16:3865349-3865290 | Zfp597 |
| 2.2415816 | up | NM_172717 | chr5:110600898-110600957 | Chfr |
| 3.4555995 | up | NM_001110327 | chr5:9132516-9132457 | Dmtf1 |
| 6.0432713 | up | NM_028544 | chr7:52894134-52894193 | Rasip1 |
| 3.205388 | up | NM_007483 | chr12:8504810-8504751 | Rhob |
| 2.8697956 | up | NM_026375 | chr1:181676542-181676483 | Ahctf1 |
| 4.3777219 | up | AK138480 | chr9:14523423-14523482 |  |
| 4.5940578 | up | AK029875 | chr8:73649716-73649775 | Gm3643 |
| 2.2171062 | up | NM_008273 | chr2:74522136-74522195 | Hoxd11 |
| 2.3689668 | up | NM_146071 | chr16:32786202-32786143 | Muc20 |
| 5.4697656 | up | NM_182990 | chr2:84885915-84886454 | Ssrp1 |
| 4.3099302 | up | NM_023739 | chr4:40971847-40971906 | Nfx1 |
| 7.1829128 | up | NM_023476 | chr4:129843103-129843044 | Tinagl1 |
| 2.8444762 | up | NM_028420 | chr16:20619400-20619341 | Camk2n2 |
| 2.4768715 | up | NM_001081382 | chr6:47974284-47974225 | Zfp777 |
| 2.125893 | up | NM_172839 | chr19:40922847-40922906 | Ccnj |
| 10.8131301 | up | NM_001081062 | chr13:113780810-113780869 | Ccno |
| 5.0233044 | up | NM_021498 | chr4:62185404-62185138 | Pole3 |
| 12.803071 | up | NM_153143 | chr11:69693686-69693627 | Kctd11 |
| 14.3011821 | up | XR_032476 | chr7:3813627-3813568 | Gm10693 |
| 8.8986594 | up | NM_207655 | chr11:16813815-16813874 | Egfr |
| 2.1818441 | up | NM_146248 | chr17:35667707-35667766 | Cchcr1 |
| 2.5081951 | up | NM_026192 | chr15:102537538-102537479 | Calcoco1 |
| 2.4165318 | up | NM_007484 | chr3:104597203-104597262 | Rhoc |
| 8.1628843 | up | NM_009965 | chr11:77533002-77532943 | Cryba1 |
| 2.56656 | up |  | chr12:113169224-113169165 |  |
| 25.1383491 | up | NM_009778 | chr17:57343475-57343416 | C3 |
| 39.747337 | up | NM_144520 | chr11:3997333-3997274 | Sec14l2 |
| 2.9654807 | up | NM_001039076 | chr14:35374748-35374689 | Ldb3 |
| 2.8031394 | up | AK007003 | chr7:29221914-29221972 | 1700085B13Rik |
| 6.3158285 | up | XM_003945637 | chr8:128040891-128040832 | LOC101056077 |
| 4.8012112 | up | NM_007443 | chr4:62804711-62804386 | Ambp |
| 2.108535 | up | AK084110 | chr12:79350742-79350801 | D130095D21Rik |
| 2.7453095 | up | NM_033614 | chr19:38258322-38258381 | Pde6c |
| 8.838855 | up | NM_008037 | chr5:32460076-32460135 | Fosl2 |
| 2.4761917 | up | NM_008233 | chr17:56239821-56239880 | Hdgfrp2 |
| 10.2122652 | up | NM_001039120 | chr2:152333593-152333534 | Defb26 |
| 5.726528 | up | NM_027195 | chr4:148319162-148319221 | Casz1 |
| 2.0105698 | up |  | chr4:153978566-153978625 |  |
| 9.9961268 | up | NM_028460 | chr3:87564206-87564147 | Pear1 |
| 3.3847306 | up | AF399829 | chr11:46569677-46569736 | Havcr1 |
| 2.9281986 | up | NM_019703 | chr13:6583872-6581718 | Pfkp |
| 4.8652391 | up | AK079318 | chr11:77911970-77912029 | Fam222b |
| 4.8026344 | up |  | chr16:16885499-16885558 | Top3b |
| 2.4143009 | up | NM_011614 | chr11:69500401-69500342 | Tnfsf12 |
| 10.4117257 | up | NM_146153 | chr4:125849065-125849006 | Thrap3 |
| 3.3565288 | up | NM_028385 | chr6:113103335-113103394 | Setd5 |
| 557.4185872 | up | NM_181849 | chr3:82846393-82846334 | Fgb |
| 3.6000312 | up | NM_026083 | chr14:75730024-75730083 | Zc3h13 |
| 4.53168 | up |  | chr3:101855473-101855532 |  |
| 2.397428 | up | NM_010548 | chr1:132920943-132921002 | Il10 |
| 2.7759912 | up | NM_080553 | chr17:27259102-27259161 | Itpr3 |
| 4.8525914 | up | NM_021409 | chr2:167912863-167912922 | Pard6b |
| 25.74244 | up | NM_011448 | chr11:112648809-112648868 | Sox9 |
| 23.9199256 | up |  | chrX:154587994-154588053 |  |
| 2.493003 | up | NR_027959 | chr16:17834458-17834517 | B830017H08Rik |
| 7.1883866 | up | NM_025793 | chr11:121199940-121199881 | Wdr45b |
| 2.6363575 | up | NM_183112 | chr2:92223699-92223758 | 1700029I15Rik |
| 6.7355885 | up | NM_023821 | chr13:93853267-93853208 | Cmya5 |
| 4.2522959 | up | AK011142 | chr8:124140522-124140463 | 2600002B07Rik |
| 3.5638114 | up | NM_019432 | chr1:121964169-121964110 | Tmem37 |
| 2.1138614 | up | NM_001195730 | chr4:129287659-129287600 | Dcdc2b |
| 4.2913551 | up | AK043214 | chr8:42104435-42104376 | Mtus1 |
| 2.7137244 | up | XR_140605 | chr3:79376385-79376444 |  |
| 12.5612837 | up |  | chr8:074048674-074048733 |  |
| 50.1784152 | up | NM_001168294 | chr12:105492556-105492615 | Serpina3f |
| 4.4390101 | up | AK017326 | chr3:51875612-51875553 | 5430420F09Rik |
| 3.3339866 | up | NM_001042557 | chr8:4245752-4245811 | Map2k7 |
| 11.3391611 | up | NM_145980 | chr6:116602564-116602623 | 8430408G22Rik |
| 2.9292554 | up | NM_027338 | chr8:23317324-23318373 | Vps36 |
| 5.1199581 | up | NM_028035 | chr6:51540112-51540171 | Snx10 |
| 5.5877001 | up | AK082796 | chr11:66850429-66850488 | C330020G15Rik |
| 2.2837235 | up | NM_172779 | chrX:53760732-53760791 | Ddx26b |
| 2.771034 | up | NM_026346 | chr15:58011811-58011752 | Fbxo32 |
| 3.3025216 | up | AK046028 | chr1:5148366-5148425 | B230334L07Rik |
| 10.4932894 | up | AK008922 | chr10:79221282-79221341 | Fgf22 |
| 2.4080377 | up | NM_011544 | chr9:71692086-71692059 | Tcf12 |
| 17.3980414 | up | AK148702 | chr9:10377916-10377857 |  |
| 3.5751778 | up |  | chr17:035440553-035440494 |  |
| 6.5600463 | up | NM_009716 | chr15:80087902-80087961 | Atf4 |
| 4.6561644 | up |  | chr15:008395464-008395405 |  |
| 7.9838117 | up | AK078279 | chr14:60840473-60824784 | Nupl1 |
| 13.2123941 | up | NM_029587 | chr6:32008760-32008819 | 1700012A03Rik |
| 5.4056796 | up | NM_001039104 | chr7:71371607-71371666 | Trpm1 |
| 18.1726398 | up | NM_001204129 | chr11:118310045-118310104 | C1qtnf1 |
| 5.1979725 | up | NM_028748 | chr9:61801668-61801609 | Paqr5 |
| 2.3410871 | up | NR_030715 | chr8:10924722-10924663 | 3930402G23Rik |
| 5.3128965 | up | NM_177379 | chr9:32068263-32068322 | Arhgap32 |
| 2.6640829 | up | NM_145384 | chr4:138854545-138854486 | Pqlc2 |
| 3.5819008 | up | NM_001024917 | chr5:66198590-66198649 | N4bp2 |
| 2.6258891 | up | NM_172778 | chrX:16286486-16286427 | Maob |
| 3.3918383 | up | NM_021467 | chr1:137705328-137706203 | Tnni1 |
| 5.2851888 | up | AK028898 | chr11:50012927-50012868 | Sqstm1 |
| 3.4247294 | up | NM_013872 | chr15:81781601-81781542 | Pmm1 |
| 2.1772665 | up | NM_001111143 | chr3:107014272-107014213 | Cym |
| 3.554595 | up | NM_026672 | chr3:107729256-107729218 | Gstm7 |
| 49.6019042 | up | NM_001004174 | chr2:122466640-122466699 | AA467197 |
| 9.78987 | up | BC056758 | chr19:3866971-3867030 | Chka |
| 2.1944555 | up |  | chr1:44113714-44113655 |  |
| 2.1773238 | up |  | chr6:091395941-091395882 |  |
| 12.0681933 | up | NM_025749 | chr18:52799280-52799339 | Zfp474 |
| 2.106633 | up |  | chr18:035655445-035655504 |  |
| 2.1854403 | up | NM_175639 | chr17:72007156-72007215 | Wdr43 |
| 2.5020403 | up | NM_001081151 | chr8:119728779-119728838 | Gan |
| 3.7922919 | up | AK018043 | chr3:16111728-16111787 | 5830468K08Rik |
| 6.2625534 | up | AK020044 | chr17:88457036-88456977 | 5930436O19Rik |
| 7.4438537 | up | CB320031 | chrY_random:23367439-23367380 | 8030402F09Rik |
| 2.1868973 | up | NM_001007570 | chr8:72708357-72708298 | Slc25a42 |
| 2.2473113 | up | NM_008970 | chr6:147200985-147200926 | Pthlh |
| 7.7408769 | up | NM_172051 | chr10:94050729-94050788 | Tmcc3 |
| 5.7427211 | up | NM_001033225 | chr4:33332947-33332888 | Pnrc1 |
| 13.0568049 | up | NM_001128606 | chr4:131563352-131563293 | Epb4.1 |
| 2.2119227 | up | NM_007782 | chr4:125721478-125721537 | Csf3r |
| 3.3128487 | up | NM_145211 | chr5:121346794-121346735 | Oas1a |
| 37.836145 | up | NM_181584 | chrX:72235404-72234128 | Gab3 |
| 3.1711036 | up | AK036004 | chr4:147829016-147829075 | Mtor |
| 3.1164293 | up | NM_001122899 | chr4:101485725-101485784 | Lepr |
| 5.1022037 | up | NM_172435 | chrX:104300217-104300276 | P2ry10 |
| 2.0669398 | up | NM_001081413 | chr4:43277637-43277696 | Unc13b |
| 4.5498819 | up | NM_001037707 | chr7:30679812-30679753 | Zfp27 |
| 3.7582968 | up | NM_001035533 | chr4:57905932-57905991 | Akap2 |
| 10.8523577 | up | NM_173070 | chr3:92304254-92304195 | Sprr4 |
| 6.5156902 | up | NM_025831 | chr13:34719769-34719710 | Pxdc1 |
| 4.0521197 | up |  | chr8:080016983-080017042 |  |
| 2.4796268 | up | NM_145823 | chr11:107070255-107070196 | Pitpnc1 |
| 12.197441 | up | XM_906502 | chr7:25479872-25479931 | Gm1096 |
| 3.9756479 | up | NM_020042 | chr17:49594644-49594703 | Mocs1 |
| 2.8445156 | up | NM_172385 | chr7:38264311-38264252 | Zfp536 |
| 3.8025705 | up | NM_010544 | chr1:74991965-74991906 | Ihh |
| 3.1796889 | up | NM_001163855 | chr17:34596960-34597019 | BC051142 |
| 2.3857578 | up | NM_001162947 | chr8:23238818-23238759 | Nek3 |
| 3.5918099 | up | NM_027498 | chr9:46029257-46029316 | Sik3 |
| 6.1191574 | up |  | chr7:085987798-085987739 |  |
| 3.354323 | up | NM_019588 | chr19:38857137-38857196 | Plce1 |
| 2.651528 | up |  | chr11:033338823-033338882 |  |
| 8.6954965 | up | NM_030696 | chr11:120820030-120820089 | Slc16a3 |
| 3.1178344 | up | NM_001042528 | chr2:24473264-24473205 | Cacna1b |
| 10.2540555 | up | AK009785 | chr16:93792460-93792401 | 2310043M15Rik |
| 3.0935156 | up | NM_028965 | chr11:96628930-96628871 | Snx11 |
| 7.2121405 | up | AK050695 | chr5:32304923-32304982 | Bre |
| 10.227129 | up | XR_142057 | chr3:120874073-120874132 | Gm10652 |
| 2.9731346 | up | NM_199304 | chr2:154472419-154472478 | Zfp341 |
| 2.2333319 | up | NM_023884 | chr1:158739163-158739104 | Ralgps2 |
| 2.2579445 | up | NM_009208 | chr1:75555599-75555658 | Slc4a3 |
| 8.7308245 | up | NM_182957 | chr5:15029133-15029074 | Gm17019 |
| 3.5970295 | up | NM_010413 | chrX:7507320-7507261 | Hdac6 |
| 2.297857 | up | NM_019402 | chr14:55516049-55516108 | Pabpn1 |
| 6.4944583 | up |  | chr16:056703668-056703609 |  |
| 2.6961869 | up | NM_138680 | chr6:38558870-38558929 | Luc7l2 |
| 2.182111 | up | NM_031368 | chr3:88172732-88172673 | Bglap3 |
| 5.3676236 | up | AK134919 | chr6:29749493-29749552 | Ahcyl2 |
| 3.5186434 | up |  | chr5:149776457-149776398 |  |
| 9.673694 | up | NM_008987 | chr3:66029245-66029304 | Ptx3 |
| 3.0772307 | up | NM_015783 | chr4:155573552-155573533 | Isg15 |
| 2.0137422 | up | NM_207653 | chr1:58811116-58811175 | Cflar |
| 2.7623865 | up | CK128530 | chr10:92981534-92981593 | Hal |
| 3.5497101 | up | AK018020 | chr14:47937086-47937145 | 5830458C19Rik |
| 2.3971896 | up | NM_173369 | chr8:91229427-91229486 | Cyld |
| 2.9934911 | up | NM_016699 | chr4:147954469-147954528 | Exosc10 |
| 9.8240098 | up | NM_007778 | chr3:107544392-107544333 | Csf1 |
| 2.2394007 | up | NM_001110832 | chr17:48526745-48526686 | Nfya |
| 9.1673115 | up | AK016828 | chr15:84410013-84409954 | 4933416A02Rik |
| 31.8562424 | up | NM_146843 | chr2:86730449-86730390 | Olfr1097 |
| 3.8420758 | up | XR_141057 | chr11:79545184-79545241 | Gm10387 |
| 3.1858515 | up | NM_001081409 | chr15:66426480-66426539 | Phf20l1 |
| 5.1308456 | up | NM_181593 | chr7:27992475-27992416 | Itpkc |
| 37.4644145 | up | NM_009803 | chr1:173147776-173147835 | Nr1i3 |
| 6.0176825 | up | NR_003278 | chr17:39985058-39985117 | Rn18s |
| 2.498943 | up | NM_148917 | chr4:122975265-122975324 | Pabpc4 |
| 2.0559106 | up | NR_036457 | chr3:121340110-121340051 | A730020M07Rik |
| 3.8515496 | up | NM_021606 | chr2:38441649-38441708 | Nek6 |
| 4.5439662 | up | NR_045840 | chr8:28260098-28260157 | 4933416M07Rik |
| 2.8209176 | up | NM_026107 | chr11:65642681-65642740 | Zkscan6 |
| 7.2218778 | up | AK042039 | chr6:39069696-39069755 |  |
| 2.1175238 | up | NM_019765 | chr5:124029337-124029278 | Clip1 |
| 2.6077011 | up | AK004107 | chr17:87682699-87682758 | Ttc7 |
| 2.2813383 | up | NM_010282 | chr13:14146020-14145961 | Ggps1 |
| 5.3620624 | up | NM_175836 | chr11:30059308-30054358 | Sptbn1 |
| 2.3288375 | up | NM_001113362 | chr5:36833313-36833254 | Tbc1d14 |
| 2.4816764 | up | NM_146148 | chr4:104488538-104488479 | C8a |
| 4.0932032 | up | NM_198012 | chr7:109826406-109826347 | Trim68 |
| 4.656839 | up | NR_033506 | chrUn_random:551587-551528 | Gm3893 |
| 68.6262682 | up | AK019662 | chr12:72484608-72484549 | 1700083H02Rik |
| 3.2284999 | up | NM_031998 | chr6:30603789-30603730 | Cep41 |
| 3.8601842 | up | NM_079835 | chr17:34506361-34506420 | Btnl2 |
| 29.1292045 | up | NM_148933 | chr2:180209483-180209542 | Slco4a1 |
| 3.0870204 | up |  | chr6:015355616-015355675 |  |
| 4.4928161 | up | NM_146085 | chr18:36830873-36830814 | Apbb3 |
| 2.9326339 | up | NM_183140 | chr4:118842372-118842313 | Zfp691 |
| 11.5058017 | up | NM_008416 | chr8:87501940-87501881 | Junb |
| 11.9307224 | up |  | chr9:044102039-044101980 |  |
| 3.9927856 | up | BY326470 | chr12:71776353-71776412 |  |
| 3.1898304 | up |  | chr6:048016024-048015965 |  |
| 3.5002501 | up | NM_175454 | chr11:115209083-115209024 | Hid1 |
| 2.9079249 | up | NM_010706 | chr7:29626661-29626720 | Lgals4 |
| 4.2761946 | up | NM_011176 | chr9:30897308-30897249 | St14 |
| 2.0080432 | up | NM_134050 | chr12:77899050-77898991 | Rab15 |
| 52.0578455 | up | NM_018866 | chr5:96389861-96389920 | Cxcl13 |
| 2.6462079 | up | NM_001271490 | chrX:7352069-7352128 | Tfe3 |
| 2.351312 | up | NM_026932 | chr4:118298510-118298569 | Ebna1bp2 |
| 2.1711369 | up |  | chr13:012794813-012794872 |  |
| 13.1577144 | up | NM_029660 | chr7:114354371-114354430 | Rbmxl2 |
| 3.3863585 | up | NM_001252539 | chr4:115242872-115242931 | Cyp4a31 |
| 8.4595376 | up | AK039759 | chr5:37680788-37680729 | Evc |
| 4.7060231 | up | AK144522 | chr7:52980170-52980229 | Fam83e |
| 2.718538 | up | NM_001199677 | chr5:135548883-135548824 | Vps37d |
| 2.1289602 | up | NM_011914 | chr5:34240890-34240831 | Nelfa |
| 2.5020212 | up | NM_009848 | chr19:40816008-40816067 | Entpd1 |
| 2.0584329 | up | NM_013831 | chr18:78120684-78120743 | Pstpip2 |
| 2.4089538 | up |  | chr13:067552666-067552607 |  |
| 5.630773 | up | NM_001081396 | chr15:57784469-57784528 | Tbc1d31 |
| 3.6074247 | up | NM_134037 | chr11:100365577-100365518 | Acly |
| 3.299156 | up | NM_001253717 | chr6:114809893-114809952 | Atg7 |
| 2.2554792 | up | NM_009287 | chr7:109585312-109585371 | Stim1 |
| 2.5815449 | up | NM_008807 | chr7:52777657-52777716 | Tulp2 |
| 6.813561 | up | NM_001164504 | chr18:77700553-77700494 | Rnf165 |
| 4.0443095 | up | NM_177301 | chr7:29605608-29605667 | Hnrnpl |
| 2.7616548 | up | NM_145836 | chr12:88221727-88221668 | Irf2bpl |
| 6.2205623 | up |  | chr3:112985328-112985269 |  |
| 7.1941224 | up | NM_020518 | chr9:37351727-37351786 | Vsig2 |
| 13.6356635 | up |  | chr7:048038111-048038170 |  |
| 2.1290806 | up | AK168315 | chr2:164576735-164576794 | Dnttip1 |
| 5.5000217 | up | NM_009238 | chr13:29042915-29042856 | Sox4 |
| 12.8672094 | up | NM_010664 | chr15:101862341-101862400 | Krt18 |
| 3.1837565 | up | NM_028923 | chr2:29814108-29814167 | Gle1 |
| 2.6725446 | up | NM_027992 | chr6:13037120-13037179 | Tmem106b |
| 9.0247439 | up | NM_001081212 | chr8:11004549-11004490 | Irs2 |
| 5.7912449 | up | XM_003945380 | chr4:98963069-98963128 | Gm12689 |
| 2.1386992 | up | NM_029315 | chr13:78165362-78165421 | Pou5f2 |
| 13.1591737 | up | AB041803 | chr6:31115582-31115523 | AB041803 |
| 2.0029828 | up | NM_028047 | chr7:25207609-25207668 | Smg9 |
| 18.974269 | up | NM_016983 | chr16:17981114-17981173 | Vpreb2 |
| 2.5248313 | up | NM_016767 | chr12:87049872-87049929 | Batf |
| 22.5554162 | up | NM_010418 | chr7:63486603-63486662 | Herc2 |
| 5.701162 | up | NM_183029 | chr16:22059319-22059260 | Igf2bp2 |
| 3.317722 | up | NM_011950 | chr17:28915417-28915476 | Mapk13 |
| 5.9065062 | up | NM_001033158 | chr9:65260445-65260504 | Rasl12 |
| 4.1919349 | up | NR_038172 | chr12:70954599-70954658 | 4931403G20Rik |
| 2.153003 | up | BI558937 | chr1:87559346-87559405 | Sp100 |
| 2.8507015 | up | AK076679 | chr5:38659067-38659126 | Tmem128 |
| 4.5204519 | up | NM_016668 | chr13:94386915-94386856 | Bhmt |
| 3.6638997 | up | NM_178087 | chr9:58065141-58065082 | Pml |
| 2.0062508 | up |  | chrX:007615759-007615700 |  |
| 12.5432474 | up | NM_010442 | chr8:77620797-77620856 | Hmox1 |
| 2.2026704 | up | AK135410 | chr11:102398041-102397982 | Gpatch8 |
| 2.8183426 | up | NM_138313 | chr2:118354555-118354496 | Bmf |
| 2.2299293 | up | NM_009502 | chr14:21848574-21849035 | Vcl |
| 3.931126 | up | NM_015787 | chr13:23713766-23713707 | Hist1h1e |
| 4.8381081 | up | NM_021565 | chr10:79621032-79621091 | Midn |
| 5.8627446 | up |  | chr10:121023334-121023275 |  |
| 7.5023574 | up | NM_027057 | chr1:79723101-79723042 | Wdfy1 |
| 2.0755702 | up | XM_003689368 | chr5:24728725-24728666 |  |
| 4.4432921 | up | NM_001037298 | chr8:125005680-125005621 | Piezo1 |
| 2.148621 | up | NM_008972 | chr1:88425798-88426068 | Ptma |
| 13.9223515 | up | AK009987 | chr12:117619193-117619252 | 2310058N22Rik |
| 30.4114352 | up | NM_207548 | chr7:24539427-24539486 | Vmn1r174 |
| 2.1081149 | up | NM_009524 | chr14:29336664-29336723 | Wnt5a |
| 44.0489676 | up | AK016494 | chr7:135345676-135345617 | 4931431B13Rik |
| 2.7792596 | up | NM_146776 | chr10:129471243-129471302 | Olfr821 |
| 3.8879455 | up | AK157330 | chr14:048706339-048706398 |  |
| 16.3853093 | up | NM_028784 | chr13:36959273-36959214 | F13a1 |
| 2.0461708 | up | NM_027297 | chr4:62087853-62087912 | Prpf4 |
| 2.2728839 | up | NM_027947 | chr2:33309244-33309185 | Zbtb43 |
| 8.0683407 | up | NM_001025392 | chr10:20059589-20059648 | Bclaf1 |
| 2.0029689 | up | NM_019661 | chr11:5867151-5867210 | Ykt6 |
| 5.0122126 | up | NR_040403 | chr3:89202640-89202699 | Gm15417 |
| 3.6166225 | up | NM_133665 | chr3:87972971-87973030 | Mef2d |
| 2.2388859 | up | NM_175375 | chr18:36801311-36802602 | Ankhd1 |
| 2.0738923 | up | NM_023684 | chr2:181118195-181118254 | Lime1 |
| 2.7209429 | up | NM_172610 | chr15:83688813-83688872 | Mpped1 |
| 2.482824 | up | NM_054078 | chr10:127565977-127566036 | Baz2a |
| 2.0197648 | up | NM_013691 | chr3:89030667-89030726 | Thbs3 |
| 4.2238965 | up | NM_054096 | chr9:34994995-34994936 | Tirap |
| 2.085024 | up | NM_172134 | chr10:77903501-77903442 | Pdxk |
| 3.4992628 | up | BC054084 | chr4:3126607-3126548 |  |
| 14.8942833 | up | NM_198959 | chr4:129807528-129807469 | Hcrtr1 |
| 2.7186531 | up | NM_001077698 | chr11:103059008-103059067 | Fmnl1 |
| 3.9052232 | up | NM_027996 | chr14:21542778-21542837 | Zswim8 |
| 2.2079942 | up | NM_053252 | chr19:5708230-5708171 | Ehbp1l1 |
| 2.0493977 | up | AK039657 | chr7:150176697-150176638 |  |
| 3.7494007 | up | NM_023380 | chr16:75859172-75859113 | Samsn1 |
| 2.4665688 | up | XM_003946169 | chrUn_random:1477428-1477487 | AA792892 |
| 4.0504817 | up | AK083262 | chr7:107790063-107790122 | Rab6a |
| 6.0230503 | up | NM_029671 | chr6:41635597-41635656 | 1700034O15Rik |
| 9.6758808 | up |  | chr9:053825164-053825223 |  |
| 2.5104065 | up | NM_175087 | chr15:99435849-99435908 | Aqp6 |
| 2.4548768 | up | NM_007560 | chr3:141500753-141500694 | Bmpr1b |
| 2.2553784 | up | NM_173755 | chr11:116399438-116399379 | Ube2o |
| 40.4992504 | up | XM_003086612 | chr7:72780723-72780664 |  |
| 4.2191313 | up | AK012053 | chr8:11622453-11622394 | 2610319H10Rik |
| 2.0177344 | up |  | chr1:100163699-100163758 |  |
| 2.8496051 | up | NM_001081223 | chr18:11901214-11901273 | Rbbp8 |
| 10.1263335 | up | NM_027237 | chr19:4496987-4496928 | 2010003K11Rik |
| 2.205589 | up | NM_010828 | chr10:17444690-17444749 | Cited2 |
| 5.0120489 | up | NM_025818 | chr11:72896557-72896616 | 1200014J11Rik |
| 8.3965461 | up | NM_010821 | chr19:12537697-12537756 | Mpeg1 |
| 3.1720797 | up | NM_177429 | chrX:162828963-162828904 | Ofd1 |
| 2.2764188 | up | NM_030096 | chr11:83775627-83775686 | Ddx52 |
| 3.5603158 | up | NM_029953 | chr3:132822856-132822797 | Arhgef38 |
| 2.1471709 | up | AK019470 | chr14:32875310-32875369 | Galnt15 |
| 6.9189379 | up | NM_001085373 | chr18:44589329-44589270 | Mcc |
| 2.1191908 | up | NM_001204875 | chr2:29927229-29927288 | Set |
| 6.2024362 | up | NM_020583 | chr7:86065136-86065195 | Isg20 |
| 7.8293037 | up | NM_010415 | chr18:36664797-36664738 | Hbegf |
| 3.4314587 | up | NM_001109691 | chr2:92200991-92201050 | Phf21a |
| 2.5993327 | up | NM_018742 | chr7:148041064-148041005 | Bet1l |
| 2.4586023 | up | NM_009561 | chr7:25076195-25076136 | Zfp61 |
| 5.1311777 | up | NM_008580 | chr10:19860474-19861330 | Map3k5 |
| 6.4274897 | up | NM_013687 | chr17:28203753-28203694 | Tcp11 |
| 2.6437816 | up | NM_173867 | chr4:140279076-140279135 | Rcc2 |
| 15.5108624 | up |  | chr11:093829247-093829188 |  |
| 4.7552922 | up | NM_133362 |  | Erdr1 |
| 2.7721845 | up | NM_153457 | chr12:73313335-73313276 | Rtn1 |
| 5.9091212 | up |  | chr12:114507530-114507471 |  |
| 3.676917 | up | NM_011772 | chr10:128069580-128069521 | Ikzf4 |
| 2.6768235 | up | NM_025907 | chr14:32292043-32291984 | Mettl6 |
| 2.46102 | up | NM_001012322 | chr1:121960008-121960067 | Sctr |
| 5.5304773 | up | NM_011065 | chr11:68923364-68923423 | Per1 |
| 3.4314611 | up | NM_025663 | chr3:87859825-87859884 | Gpatch4 |
| 2.2537445 | up | NM_026671 | chr15:74562743-74562684 | Lypd2 |
| 5.9192976 | up | NM_011068 | chr7:86882282-86882223 | Pex11a |
| 4.9545451 | up |  | chr4:027794323-027794264 |  |
| 2.5732471 | up | NM_173453 | chr11:60678107-60678048 | Tmem11 |
| 5.4416018 | up | NM_008713 | chr5:23889636-23889695 | Nos3 |
| 2.6054905 | up | NM_001033202 | chr5:114572874-114572933 | Usp30 |
| 2.1808991 | up | NM_001040111 | chr7:108560338-108560397 | Arap1 |
| 2.5051099 | up | NM_026252 | chr11:31831509-31831568 | Cpeb4 |
| 8.7933464 | up | AK136006 | chrX:16688055-16687996 | Gm4203 |
| 2.5886143 | up | NM_011814 | chr11:69466722-69466781 | Fxr2 |
| 9.0117669 | up | NM_007706 | chr10:94875125-94875066 | Socs2 |
| 2.2192159 | up | AK141859 | chr5:32913716-32913657 | Gm10461 |
| 2.0812583 | up | NM_008933 | chr16:10791608-10791549 | Prm2 |
| 2.087533 | up | NM_001162533 | chr4:125827922-125827863 | Sh3d21 |
| 2.296303 | up | NM_029012 | chr5:115547547-115547606 | Sppl3 |
| 3.2611414 | up | NM_011444 | chr6:143776971-143776945 | Sox5 |
| 2.1626908 | up | NM_029035 | chr4:149272135-149272076 | Spsb1 |
| 3.0611718 | up | NM_053082 | chr7:148679261-148679320 | Tspan4 |
| 12.9011163 | up | NM_213659 | chr11:100749657-100749598 | Stat3 |
| 3.5720285 | up | NM_145562 | chr5:92052938-92052997 | Parm1 |
| 3.4291129 | up | XM_003945946 | chr13_random:225099-225040 | LOC101056555 |
| 3.1431781 | up | NM_029810 | chr19:46963134-46963075 | Nt5c2 |
| 2.4306413 | up | AK018780 | chr19:4257014-4256955 | 1500032F14Rik |
| 21.9442643 | up | XM_356811 | chr15:54143707-54143648 | Gm5215 |
| 7.1930179 | up | NM_010735 | chr17:35340334-35340275 | Lta |
| 4.2837382 | up | AK045785 | chr5:116017016-116017075 |  |
| 2.1934025 | up |  | chr6:086237275-086237334 |  |
| 2.7495662 | up | NM_025996 | chr2:163879381-163879322 | Tomm34 |
| 2.9885523 | up | AK081893 | chr7:117263565-117263506 | D7Wsu130e |
| 3.6789116 | up | NM_008331 | chr19:34723796-34723855 | Ifit1 |
| 2.5255547 | up | NM_027724 | chr13:38097993-38097934 | Cage1 |
| 5.080531 | up | NM_033321 | chr11:72986128-72986187 | P2rx5 |
| 3.017877 | up | NM_027511 | chr4:43740921-43740980 | Hrct1 |
| 3.3314841 | up | NM_013475 | chr11:108275610-108275669 | Apoh |
| 4.5253645 | up | NM_138672 | chr14:31952347-31952288 | Stab1 |
| 23.5827197 | up | NM_008392 | chr14:103455171-103455231 | Irg1 |
| 13.6098145 | up | NM_146706 | chr11:73935624-73935683 | Olfr401 |
| 7.4253298 | up | NM_177357 | chr16:34014252-34014193 | Kalrn |
| 2.0650666 | up | NR_028427 | chr19:53526210-53526151 | 5830416P10Rik |
| 5.2828319 | up | NM_198021 | chr10:89102960-89102901 | Scyl2 |
| 10.7956612 | up | NM_009690 | chr3:87174823-87174882 | Cd5l |
| 2.3680992 | up | NM_001039048 | chr4:133881557-133881616 | Trim63 |
| 85.6736471 | up | NM_008458 | chr12:105385411-105385352 | Serpina3c |
| 2.3450065 | up | NM_013683 | chr17:34330854-34330913 | Tap1 |
| 2.1252536 | up | NM_146012 | chr10:126434239-126434298 | Ctdsp2 |
| 5.2681338 | up |  | chr7:032717751-032717692 |  |
| 18.4240267 | up | NM_010104 | chr13:42403101-42403160 | Edn1 |
| 2.9153714 | up | NM_001146687 | chr10:80777803-80777862 | Pip5k1c |
| 2.3169678 | up | NM_139149 | chr7:135125378-135125437 | Fus |
| 2.0992733 | up | NM_009963 | chr2:92243866-92243807 | Cry2 |
| 11.5558542 | up | NM_029083 | chr10:59412545-59412486 | Ddit4 |
| 2.3693347 | up |  | chr15:025611930-025611989 |  |
| 6.7973669 | up | NM_007836 | chr6:66985435-66985376 | Gadd45a |
| 15.8218391 | up | NM_001177848 | chr2:164405847-164405788 | Wfdc6a |
| 2.7272009 | up | AK029220 | chr12:92995813-92995754 | Gm2347 |
| 2.6056386 | up | NM_022028 | chr12:71066181-71066122 | Sav1 |
| 5.1114372 | up | NM_001024716 | chr15:78835807-78835866 | Triobp |
| 3.6923702 | up | NM_178403 | chr5:23246595-23246536 | Pus7 |
| 2.1017171 | up | NM_172903 | chr7:87495636-87495577 | Man2a2 |
| 2.2327721 | up | NM_025340 | chr15:76177529-76177470 | Sharpin |
| 2.3908506 | up | NM_011609 | chr6:125312376-125312435 | Tnfrsf1a |
| 2.8336377 | up | NR_040758 | chr6:108527104-108527045 | 0610040F04Rik |
| 2.1992027 | up | NM_010923 | chr2:157387978-157388037 | Nnat |
| 5.6800223 | up | NM_001177951 | chrX:9753190-9753131 | Rpgr |
| 5.3001985 | up | NM_016868 | chr7:17636585-17636526 | Hif3a |
| 8.0765894 | up |  |  |  |
| 2.3005678 | up | NM_001033550 | chr5:105915147-105915206 | Lrrc8b |
| 17.1305445 | up | NM_017391 | chr16:92079366-92079425 | Slc5a3 |
| 4.6676099 | up | NM_152813 | chr11:102931695-102931636 | Plcd3 |
| 3.5278006 | up | NM_146054 | chr14:46078561-46078502 | Fermt2 |
| 4.8018589 | up | NM_198191 | chr2:32439232-32439291 | Pip5kl1 |
| 2.4252559 | up |  | chr11:095685325-095685266 |  |
| 5.3385743 | up | NM_020610 | chr7:116901632-116901573 | Nrip3 |
| 9.3038833 | up | AK089567 | chr2:98502597-98502655 |  |
| 63.1233558 | up | AK087371 | chr7:151610225-151610284 | Shank2 |
| 10.3064244 | up | NM_001013581 | chr8:129950493-129950552 | Pard3 |
| 2.2209471 | up | NM_001033285 | chr1:181970271-181970330 | Cdc42bpa |
| 2.9820811 | up | NM_028000 | chr8:26835202-26835261 | Ppapdc1b |
| 11.4759167 | up | NM_001005421 | chr9:44915486-44915545 | Amica1 |
| 3.92877 | up | NM_001042699 | chr12:106168273-106168214 | Syne3 |
| 21.9371971 | up | AK147342 | chr5:139476432-139476491 |  |
| 4.0915668 | up | NM_011723 | chr17:74233570-74233511 | Xdh |
| 2.0939914 | up | NM_183428 | chr4:131479403-131479344 | Epb4.1 |
| 3.7011561 | up | NM_011276 | chrX:101158037-101157978 | Rlim |
| 18.6985825 | up | NM_025731 | chr19:7713570-7713629 | Hrasls5 |
| 3.3805196 | up | NM_023420 | chr13:97407091-97407150 | Col4a3bp |
| 2.7282862 | up | NM_145823 | chr11:107073551-107073492 | Pitpnc1 |
| 3.5053172 | up | NM_080428 | chr3:84781446-84781505 | Fbxw7 |
| 2.2136243 | up | NM_139059 | chr11:120830494-120830435 | Csnk1d |
| 10.0647261 | up | NM_153163 | chr6:23213567-23213508 | Cadps2 |
| 20.0490754 | up | AJ237586 | chr6:71341160-71341101 | Rmnd5a |
| 2.6627777 | up | NM_020557 | chr12:27164119-27164178 | Cmpk2 |
| 5.9821608 | up | NM_001190466 | chr12:72419790-72419849 | Dact1 |
| 3.9412329 | up | NM_001164598 | chr8:129114767-129114708 | Irf2bp2 |
| 4.8602786 | up | NM_028958 | chrX:130994717-130994658 | Taf7l |
| 2.4672895 | up | NM_007561 | chr1:59927276-59927335 | Bmpr2 |
| 53.7844865 | up | AK029588 | chr18:32272076-32272135 | 4933435E02Rik |
| 9.7262455 | up | AK030345 | chr2:52729313-52729372 | 5230400M03Rik |
| 2.8905139 | up | NM_001146275 | chr18:60551352-60551411 | Iigp1 |
| 2.9528642 | up | NR_015464 | chr2:91077989-91078048 | A330069E16Rik |
| 2.3892387 | up | NM_001099296 | chr4:133807108-133807049 | Grrp1 |
| 2.7981888 | up | NR_027987 | chr19:27507000-27507059 | C030016D13Rik |
| 4.9824043 | up | NM_011381 | chr17:86023349-86023408 | Six3 |
| 2.0209822 | up | NM_011161 | chr15:88973218-88973159 | Mapk11 |
| 3.2299392 | up | NM_001195130 | chr4:128430039-128430098 | Phc2 |
| 2.4493739 | up | AK158124 | chrX:99267221-99267280 | Nhsl2 |
| 3.0399424 | up |  | chr6:122849805-122849746 |  |
| 6.7933477 | up |  | chr17:035538828-035538887 |  |
| 7.2466956 | up | NM_001177668 | chr2:29835660-29835719 | Sptan1 |
| 7.5009982 | up | NM_017379 | chr6:121176046-121176105 | Tuba8 |
| 2.0479578 | up | NM_001029937 | chr11:3978896-3978955 | Sec14l3 |
| 2.6171561 | up | NM_009920 | chr19:5092927-5092871 | Cnih2 |
| 3.107049 | up | NM_027102 | chr9:37345795-37345854 | Esam |
| 2.3695728 | up | NM_026470 | chr4:111447424-111447483 | Spata6 |
| 104.0240282 | up | NM_007669 | chr17:29237377-29237436 | Cdkn1a |
| 2.2793603 | up | NM_011504 | chr3:108628984-108628925 | Stxbp3a |
| 2.5320867 | up | AF138742 | chr16:19065521-19065462 |  |
| 7.1059882 | up | NM_001033276 | chr15:98665230-98665171 | Kmt2d |
| 3.3431398 | up | NM_172590 | chr13:95780290-95780349 | Wdr41 |
| 2.4448941 | up | NM_009274 | chr5:23033720-23031536 | Srpk2 |
| 3.6071365 | up | NM_001277116 | chr5:54044777-54044836 | Rbpj |
| 14.7551809 | up | NM_011706 | chr11:62413739-62413798 | Trpv2 |
| 2.0754123 | up | NM_026931 | chr8:25548213-25548154 | 1810011O10Rik |
| 2.0798556 | up | NM_177577 | chr13:25297328-25297387 | Dcdc2a |
| 4.3444833 | up | NM_172807 | chr13:104995717-104995658 | Ppwd1 |
| 32.5877626 | up |  | chr11:089170620-089170679 |  |
| 3.7767074 | up | NM_021313 | chr1:74640388-74640329 | Rnf25 |
| 89.7237118 | up | NM_016693 | chr4:132808672-132808731 | Map3k6 |
| 3.0217954 | up | AK171768 | chr12:3235525-3235584 |  |
| 2.2062343 | up | NM_007397 | chr9:119342560-119342619 | Acvr2b |
| 5.9270873 | up | NM_023910 | chr5:138189022-138189081 | Tsc22d4 |
| 2.3653751 | up | NM_001035526 | chr4:126524498-126525070 | AU040320 |
| 2.5264612 | up | NM_001134829 | chr1:193603657-193603716 | Lpgat1 |
| 2.4555922 | up | NM_029466 | chr2:15000722-15000781 | Arl5b |
| 10.7963639 | up | AK005516 | chr17:71531232-71531291 | 1600022D10Rik |
| 3.1685922 | up | NM_144800 | chr15:58775067-58775008 | Mtss1 |
| 23.3528674 | up |  | chr17:21231478-21231537 |  |
| 3.8135669 | up |  | chr4:022148606-022148665 |  |
| 3.3813093 | up |  | chr12:100687023-100686964 |  |
| 6.8101251 | up |  | chr13:109008861-109008802 |  |
| 16.5298898 | up | NM_001282006 | chr11:72158290-72158231 | Tekt1 |
| 3.3700897 | up |  |  |  |
| 3.4234537 | up | NM_175341 | chr14:120803865-120803924 | Mbnl2 |
| 2.8001284 | up | NM_013842 | chr11:5425816-5425875 | Xbp1 |
| 3.8506746 | up | NM_001205234 | chr19:6533156-6533215 | Nrxn2 |
| 4.3389916 | up | NR_045715 | chr10:126639568-126639627 | F420014N23Rik |
| 3.6858358 | up | NM_001025439 | chr3:126545798-126545857 | Camk2d |
| 3.2843047 | up | BC004748 | chr1:60135040-60134982 | Wdr12 |
| 2.2187109 | up | NM_011198 | chr1:151954744-151954803 | Ptgs2 |
| 5.2938011 | up | NM_145552 | chr4:124732442-124732501 | Gnl2 |
| 2.1225923 | up | U80890 | chr8:19697800-19697859 | LOC101056094 |
| 2.7557387 | up | NM_011166 | chr13:27404501-27404560 | Prl6a1 |
| 4.3072528 | up | NM_009778 | chr17:57343737-57343678 | C3 |
| 20.0578491 | up | NM_033601 | chr7:20393885-20393826 | Bcl3 |
| 2.771112 | up | AK090084 | chr2:76221740-76221799 | Rbm45 |
| 5.4808996 | up | NM_009375 | chr15:66682078-66682137 | Tg |
| 2.7241176 | up | NM_001271544 | chr18:36700885-36700944 | Slc4a9 |
| 3.1400269 | up |  | chr9:066358778-066358837 |  |
| 25.075883 | up | NM_019465 | chr9:40781069-40781010 | Crtam |
| 2.5997496 | up | NM_001110791 | chr19:6377677-6377736 | Sf1 |
| 7.6508816 | up | NM_001097644 | chr1:64770179-64770238 | Ccnyl1 |
| 2.5323754 | up | NM_194346 | chr14:56222187-56222246 | Rnf31 |
| 2.7143627 | up |  | chr10:087167302-087167243 |  |
| 2.8074922 | up | NM_027460 | chr4:149118772-149118713 | Slc25a33 |
| 6.4507683 | up | NM_028295 | chr16:35407779-35407720 | Pdia5 |
| 2.0865295 | up | NM_213614 | chr16:18623070-18623011 | Sept5 |
| 2.2332937 | up | AK047211 | chr12:109441873-109441814 | B930036G03Rik |
| 3.2201201 | up | BC025048 | chr9:106277943-106278000 | Dusp7 |
| 4.5114703 | up | NM_176998 | chr15:44503571-44503512 | Sybu |
| 5.5943945 | up | BU053129 | chr19:47099350-47099409 | Ina |
| 35.1304536 | up |  | chrY_random:026246267-026246208 | |
| 8.0301643 | up | NM_001003961 | chr2:153513403-153513462 | Dnmt3b |
| 4.009563 | up | NM_019550 | chr3:119422343-119422284 | Ptbp2 |
| 13.0720908 | up | XM_003945422 | chr7:123217303-123217362 | LOC101055734 |
| 8.3112127 | up | NM_011333 | chr11:81850650-81850709 | Ccl2 |
| 65.7112691 | up | NM_001102662 | chr4:111702084-111702143 | Skint1 |
| 2.444595 | up | NM_028227 | chr5:122137162-122137221 | Brap |
| 2.5101438 | up |  | chrX:067772816-067772875 |  |
| 3.8178595 | up | NM_016778 | chr1:095592276-095592335 | Bok |
| 6.8089097 | up | NM_001101486 | chr6:29240613-29240672 | Fam71f2 |
| 2.2109503 | up | NM_001243306 | chr4:134925884-134925825 | Ncmap |
| 2.488677 | up |  | chr8:114760172-114760113 |  |
| 2.2258232 | up |  | chr11:006459044-006458985 |  |
| 2.3160381 | up | NM_019803 | chr10:77108514-77108573 | Ube2g2 |
| 2.9977349 | up | NM_001009818 | chr5:93596653-93596712 | Sept11 |
| 3.7603798 | up | NM_172453 | chr9:65442945-65443004 | Pif1 |
| 4.0291737 | up | NM_031408 | chr5:137967174-137967233 | Gigyf1 |
| 2.6576879 | up |  |  |  |
| 6.2914406 | up | AK008891 | chr9:115142756-115142815 | 2210411A11Rik |
| 18.1914105 | up | E05351 | chr6:70468774-70468833 |  |
| 9.7575767 | up | NM_027717 | chr14:41875263-41875204 | Dydc2 |
| 3.0111413 | up | NM_177707 | chr10:126945799-126945858 | Stac3 |
| 4.738469 | up | AK036810 | chr15:76239777-76239836 | Mroh1 |
| 19.9078001 | up | NM_013820 | chr6:82675115-82675056 | Hk2 |
| 2.3210196 | up | NM_007780 | chr15:78181168-78181227 | Csf2rb |
| 2.2369647 | up | NM_170591 | chr14:60840473-60838990 | Nupl1 |
| 2.9530648 | up | NM_025534 | chr9_random:417312-417371 | Ccdc82 |
| 5.2431507 | up | NM_001034864 | chrX:23484609-23484668 | Gm4907 |
| 4.3794995 | up | NM_011112 | chr12:107075105-107075164 | Papola |
| 4.6767795 | up | XR_168467 | chr10:39475171-39475230 | LOC101056155 |
| 2.4200516 | up | NM_146193 | chr7:88938222-88938163 | Btbd1 |
| 9.0511318 | up | NM_013794 |  | Klra16 |
| 2.52048 | up | AK019926 | chr13:100272491-100272432 | 5330431K02Rik |
| 3.5513314 | up | NM_138590 | chr4:44944989-44945048 | Zcchc7 |
| 2.1996021 | up | NM_177687 | chr6:134807823-134807882 | Crebl2 |
| 2.814976 | up | AK020481 | chr2:155917800-155917741 | Rbm12 |
| 3.455918 | up | NM_011661 | chr7:94632473-94632414 | Tyr |
| 7.0560462 | up | BB237529 | chrY_random:049672509-049672450 | |
| 2.119431 | up | NM_009557 | chr4:135849676-135849735 | Zfp46 |
| 2.9712479 | up | NM_153085 | chr18:7927006-7927065 | Wac |
| 2.7366129 | up | NM_028152 | chr19:42018494-42018435 | Mms19 |
| 5.3480539 | up | BC057675 | chr7:18126906-18126965 |  |
| 3.2362656 | up | NM_008989 | chr18:36447832-36447891 | Pura |
| 4.5207192 | up | CX224531 | chr4:57020840-57020781 | Epb4.1l4b |
| 4.0832013 | up | NM_028175 | chr8:4237411-4237470 | Lrrc8e |
| 2.0683825 | up |  | chr19:042238797-042238738 |  |
| 3.5016049 | up |  | chr4:34842664-34842723 | Cga |
| 2.1274709 | up | NM_145532 | chr2:127530185-127530126 | Mall |
| 56.5184972 | up | NM_009710 | chr7:109259395-109259454 | Art1 |
| 2.0771235 | up | NM_021332 | chr17:31073261-31073320 | Glp1r |
| 4.7465589 | up | NM_001017966 | chr4:141239761-141239702 | Ddi2 |
| 2.2059652 | up | NM_010065 | chr2:32170471-32170412 | Dnm1 |
| 2.2306467 | up | NM_175311 | chr5:31501419-31501360 | Zfp513 |
| 2.923973 | up | NM_025533 | chr7:52332727-52332786 | Nosip |
| 28.4266384 | up |  | chr2:173098578-173098519 |  |
| 2.1660327 | up | NM_025446 | chr10:13372655-13372596 | Aig1 |
| 2.9077596 | up | NM_172496 | chr11:12275844-12269667 | Cobl |
| 2.6003629 | up | NM_152220 | chr19:11852097-11852038 | Stx3 |
| 3.1324294 | up |  | chr12:034531694-034531753 |  |
| 2.3791001 | up | XM_003086644 | chr7:134655656-134655715 | Srcap |
| 2.1045001 | up | EU616813 | chr12:110968992-110969051 | Mirg |
| 2.1092952 | up |  | chr14:027385862-027385921 |  |
| 5.0590973 | up | AK051948 | chr11:104299137-104299078 | D030002E05Rik |
| 3.009175 | up | NM_172294 | chr1:12850302-12850361 | Sulf1 |
| 2.638704 | up | NM_011841 | chr11:61302433-61302374 | Mapk7 |
| 2.4825693 | up | BC057871 | chr16:97963599-97963540 | Ripk4 |
| 8.6327607 | up | NM_023320 | chr3:95792824-95792765 | Plekho1 |
| 3.8369065 | up |  | chr8:035032341-035032400 |  |
| 3.8397304 | up | NM_030209 | chr8:122575417-122575476 | Crispld2 |
| 3.7658635 | up | NM_009052 | chrX:132674125-132674184 | Bex1 |
| 3.0261406 | up | NM_152895 | chr1:136528046-136528105 | Kdm5b |
| 21.032654 | up | AK142273 | chr10:72124449-72124508 | 2610034E01Rik |
| 2.6210342 | up | NM_013509 | chr6:124710447-124710388 | Eno2 |
| 3.5943029 | up | NM_007829 | chr17:34052404-34052463 | Daxx |
| 2.4985317 | up | NM_194333 | chr1:75122176-75122117 | Slc23a3 |
| 2.2778772 | up |  | chr14:015639430-015639371 |  |
| 2.6962676 | up | NM_001033217 | chr15:93329605-93329546 | Prickle1 |
| 4.6244423 | up | NM_009206 | chr5:31836427-31836486 | Slc4a1ap |
| 4.1659725 | up | NM_153516 | chr6:120842801-120842860 | Bcl2l13 |
| 14.1161181 | up | NM_176912 | chr7:16820160-16820101 | C5ar2 |
| 7.3653251 | up | NM_001270806 | chr14:3196624-3196683 | Gm5795 |
| 3.9601855 | up | NR_033535 | chr14:80261916-80261857 | Gm10845 |
| 8.8188003 | up |  | chr12:115776449-115776390 |  |
| 8.1806155 | up | NM_001163136 | chr12:120705295-120705354 | Macc1 |
| 2.2162291 | up | NM_021455 | chr5:135613705-135613764 | Mlxipl |
| 2.8252 | up | NM_011347 | chr1:166079660-166079719 | Selp |
| 3.2684916 | up | NM_019874 | chr4:42971494-42971553 | Dnajb5 |
| 2.0222859 | up | NM_153806 | chr3:121987377-121987989 | Dnttip2 |
| 3.1455494 | up | NM_013815 | chr12:55994057-55993998 | Baz1a |
| 4.0240733 | up | NM_007735 | chr1:82449192-82449133 | Col4a4 |
| 3.0005605 | up | NM_011653 | chr15:98780293-98780278 | Tuba1a |
| 4.9352289 | up | NR_045170 | chr13:12277840-12277781 | Gm10336 |
| 9.5548373 | up | AK090034 | chr14:43968031-43968090 | Gm3161 |
| 3.0239368 | up | NM_001134741 | chr1:158185567-158185508 | Tdrd5 |
| 2.8398714 | up | NM_010461 | chr11:96146576-96146635 | Hoxb8 |
| 4.8741073 | up | NM_207225 | chr1:93829613-93829554 | Hdac4 |
| 8.6144799 | up | NM_013661 | chr16:35663660-35663719 | Sema5b |
| 9.2901662 | up | NM_001172074 | chr1:122018168-122018227 | 3110009E18Rik |
| 2.3473289 | up | NM_010697 | chr19:46107546-46107487 | Ldb1 |
| 3.6273388 | up | NM_018815 | chr6:90963123-90963064 | Nup210 |
| 5.755483 | up | NM_053179 | chr4:46515457-46515516 | Nans |
| 2.1933104 | up | NM_013571 | chr11:78840982-78840923 | Ksr1 |
| 3.9205267 | up |  | chr14:077743594-077743536 |  |
| 12.1643253 | up | NR_033782 | chr2:179713659-179713718 | 4921531C22Rik |
| 8.0923948 | up | AK041925 | chr18:5058328-5058387 | Svil |
| 4.3204752 | up | NM_030024 | chr6:54280124-54280183 | Prr15 |
| 2.4734935 | up |  | chr6:29423406-29423465 | Gm9047 |
| 3.7553594 | up | XR_140480 | chr1:189062538-189062597 | Gm21710 |
| 2.4606901 | up | BG080185 | chrX:131544251-131544310 | C85181 |
| 3.1294345 | up | NM_016680 | chr7:20166463-20166404 | Clasrp |
| 2.1105955 | up | AK157932 | chr2:146255247-146255188 | Ralgapa2 |
| 2.084583 | up | NM_175380 | chr9:114811487-114811428 | Gpd1l |
| 7.4414925 | up | NM_001201341 | chr11:88547346-88547287 | Msi2 |
| 2.3552529 | up | NM_145836 | chr12:88222611-88222552 | Irf2bpl |
| 3.0945461 | up | NM_001110197 | chr10:29064323-29064264 | Rnf146 |
| 6.7596545 | up | NM_133857 | chr3:122636794-122636735 | Usp53 |
| 2.2618245 | up | NM_023684 | chr2:181118273-181118332 | Lime1 |
| 11.06005 | up | AK084284 | chr1:72321346-72321405 | D230017M19Rik |
| 2.3387431 | up | NM_172281 | chr7:134746835-134746894 | Rnf40 |
| 5.8892569 | up | XM_003086377 | chr4:129632708-129632767 | Spocd1 |
| 3.2612131 | up | NM_172380 | chr16:38525846-38525787 | Poglut1 |
| 9.1589645 | up | NM_172418 | chr7:52900705-52900764 | Mamstr |
| 20.9721374 | up | NM_011211 | chr4:77857565-77857506 | Ptprd |
| 3.2290505 | up | NM_001252097 | chr6:149099408-149099467 | Mettl20 |
| 4.2374237 | up | NR_028584 | chr4:63261748-63261689 | Gm11213 |
| 2.6601406 | up | NM_001160326 | chr14:76932685-76932626 | Serp2 |
| 7.7157291 | up | AK076101 | chr9:107483891-107483950 |  |
| 7.6765411 | up | BC094332 | chr9:96918853-96918794 | Spsb4 |
| 13.8290515 | up | NM_010277 | chr11:102748923-102748864 | Gfap |
| 2.5987665 | up | NM_001004176 | chr3:51491724-51491665 | Maml3 |
| 4.648513 | up | NM_145356 | chr18:76307764-76307823 | Zbtb7c |
| 2.7609485 | up | NM_053197 | chr19:45130804-45130863 | Sfxn3 |
| 2.1887373 | up | NM_001001333 | chr11:121082854-121083535 | Hexdc |
| 30.0172665 | up | NM_172290 | chr9:28804029-28803970 | Ntm |
| 2.3540682 | up | XR_105412 | chr10:77949529-77949470 |  |
| 2.7250166 | up | NM_172628 | chr18:62175070-62175129 | Sh3tc2 |
| 10.025366 | up |  | chr11:070232842-070232783 |  |
| 3.750079 | up | NM_178892 | chr3:65357710-65357769 | Tiparp |
| 3.7903007 | up | NM_183161 | chr2:180476906-180476965 | Slc17a9 |
| 5.0864673 | up | AK081553 | chr17:71102941-71103000 | 5031415H12Rik |
| 4.0727747 | up | NM_183162 | chr2:180962496-180962437 | Helz2 |
| 2.3361761 | up | NM_019827 | chr16:38241177-38241236 | Gsk3b |
| 7.3479123 | up | NM_026331 | chr14:69902856-69902797 | Slc25a37 |
| 10.3211298 | up | NM_021050 | chr6:18270974-18271033 | Cftr |
| 28.7807982 | up | NM_177607 | chr4:62208945-62209004 | 4933430I17Rik |
| 21.4636659 | up | NM_001164312 | chr1:168559184-168559125 | Gm4847 |
| 2.0285628 | up |  | chr11:076415008-076415067 |  |
| 2.6320666 | up | NM_001024728 | chr8:3584703-3584762 | C330021F23Rik |
| 3.0512244 | up | NM_009402 | chr7:19475662-19475721 | Pglyrp1 |
| 8.8992965 | up | NM_001164493 | chr12:5086418-5086359 | Klhl29 |
| 3.1241549 | up | AK018474 | chr1:174115805-174115864 | 8430437O03Rik |
| 2.4566835 | up | NM_177723 | chr1:174493788-174493847 | Vsig8 |
| 4.9821274 | up | NM_153564 | chr3:142184779-142184838 | Gbp5 |
| 2.0032071 | up | NM_134095 | chr15:81830010-81829951 | Desi1 |
| 2.070936 | up |  | chr9:073023265-073023206 |  |
| 2.1764125 | up | NM_009287 | chr7:109585303-109585362 | Stim1 |
| 11.3754152 | up | AK162496 | chr5:28807454-28807513 | 9530036O11Rik |
| 4.8332513 | up | AK082324 | chr12:109506897-109506838 | C230037L09 |
| 5.6149454 | up | NM_019984 | chr14:56319091-56319032 | Tgm1 |
| 3.3428949 | up | NM_026409 | chr5:125017952-125018011 | Ddx55 |
| 2.3899029 | up | NM_013885 | chr4:134772957-134772898 | Clic4 |
| 2.4195605 | up | NM_019766 | chr10:127514251-127514310 | Ptges3 |
| 3.8219306 | up | NM_024242 | chr13:38148091-38149059 | Riok1 |
| 5.8078734 | up |  | chr2:164393986-164394045 |  |
| 7.1321547 | up | NM_172304 | chr4:48443893-48443834 | Tex10 |
| 3.4940494 | up | NM_007564 | chr12:81209081-81209022 | Zfp36l1 |
| 3.4488402 | up | AK037456 | chr10:60903823-60903764 | A130019P10Rik |
| 3.5352249 | up | AK087888 | chr18:9928488-9928429 |  |
| 2.095906 | up | NM_025701 | chr8:3679496-3679555 | Trappc5 |
| 9.8203905 | up |  | chr10:088993283-088993342 |  |
| 2.8197032 | up | NM_144537 | chr6:30581460-30581519 | Cpa5 |
| 2.8044901 | up | NM_172945 | chr11:77284055-77283996 | Ankrd13b |
| 5.4945221 | up |  | chr3:124291608-124291549 |  |
| 3.2391651 | up | NM_016877 | chr6:34995272-34995213 | Cnot4 |
| 5.1371906 | up | XR_141056 | chr11:79387077-79387136 | D130012P04Rik |
| 11.3693274 | up | NM_007408 | chr4:86302906-86302847 | Plin2 |
| 2.9492369 | up | NM_001163754 | chr1:187107784-187107843 | Rab3gap2 |
| 6.6318207 | up | NM_172946 | chr11:99095539-99095480 | Krt222 |
| 6.2006708 | up | NM_007424 | chr7:86259741-86259801 | Acan |
| 92.4695908 | up | AB021861 | chr4:132808784-132808843 | Map3k6 |
| 3.1681152 | up | NM_008147 | chr10:51201615-51201674 | Gp49a |
| 2.1108193 | up | NM_020260 | chr16:38598862-38598803 | Arhgap31 |
| 2.1640473 | up | NM_144917 | chr6:72515975-72515916 | Elmod3 |
| 2.9195803 | up |  |  |  |
| 2.1659141 | up |  | chr15:099425214-099425273 |  |
| 2.2807764 | up | NM_001276767 | chr1:135516919-135516860 | Zc3h11a |
| 3.6467025 | up | AU018794 | chr8:122108154-122108213 | AU018794 |
| 2.5016362 | up | NM_181818 | chr2:86646343-86646284 | Olfr141 |
| 2.4683649 | up | CB183091 | chr2:71367411-71367470 | Dlx1 |
| 7.7337171 | up | NM_008378 | chr18:13148387-13149059 | Impact |
| 3.1546589 | up | AK041359 | chr4:46645568-46645509 | Tbc1d2 |
| 5.5137341 | up | AK049485 | chr10:107621913-107621972 | C430003N24Rik |
| 2.3344732 | up | NM_016907 | chr2:119074872-119074931 | Spint1 |
| 2.0552729 | up | NM_026050 | chr5:144325276-144325335 | Fam220a |
| 8.7718947 | up | NM_001033254 | chr2:118522911-118522970 | Pak6 |
| 3.327242 | up | NM_177412 | chr6:115971514-115971455 | Tmcc1 |
| 2.2174353 | up |  | chr14:073378542-073378483 |  |
| 3.0136252 | up | AK020978 | chr4:3482292-3482233 | B230117O15Rik |
| 62.413188 | up | XM_910904 | chr3:90843719-90843778 | Gm7166 |
| 4.0272248 | up | NM_010925 | chr10:77864705-77864646 | Rrp1 |
| 2.1876 | up | NM_015731 | chr2:168460022-168459963 | Atp9a |
| 3.0511572 | up | NM_053100 | chr19:46590678-46590737 | Trim8 |
| 10.8601114 | up | NM_139298 | chr11:59146953-59147012 | Wnt9a |
| 3.5307826 | up | AK042438 | chr6:120980718-120980777 | Gm9878 |
| 2.0141731 | up |  | chr11:075476336-075476395 |  |
| 3.4663973 | up | NM_146200 | chr7:133708009-133707950 | Eif3c |
| 10.7326022 | up |  | chrX:005459060-005459119 |  |
| 6.1086073 | up | NM_019494 | chr5:92788699-92788640 | Cxcl11 |
| 157.9476213 | up | NM_009521 | chr11:103679038-103679097 | Wnt3 |
| 2.587034 | up | NM_010851 | chr9:119245381-119245322 | Myd88 |
| 5.2070162 | up | NM_146823 | chr2:88327556-88327615 | Olfr1184 |
| 5.3172342 | up | NM_146118 | chr2:32270334-32270275 | Slc25a25 |
| 7.1912691 | up | NM_013807 | chr4:116801376-116801317 | Plk3 |
| 4.1627664 | up |  | chr1:82232389-82232448 |  |
| 2.0416888 | up | NM_080559 | chr4:133683905-133683846 | Sh3bgrl3 |
| 9.6529439 | up | NM_029332 | chr7:82842265-82849254 | Akap13 |
| 4.2163189 | up | NM_025845 | chr3:108708129-108708070 | Prpf38b |
| 4.669435 | up | NM_023716 | chr13:34219151-34219092 | Tubb2b |
| 3.0507977 | up | NM_175172 | chr19:59048100-59048041 | 4930506M07Rik |
| 2.3721332 | up | NM_009564 | chr2:168751005-168750946 | Zfp64 |
| 2.2335817 | up | AK020954 | chr2:76227469-76227528 | B230104C14Rik |
| 2.766215 | up | NM_020506 | chr14:58201169-58201110 | Xpo4 |
| 6.0610343 | up |  | chr15:093243972-093244031 |  |
| 5.1478692 | up | AK137149 | chr11:94149299-94149240 | Luc7l3 |
| 2.1360054 | up | NM_009693 | chr12:8012697-8012756 | Apob |
| 13.1828098 | up |  | chr10:124924625-124924566 |  |
| 3.0278694 | up | D38613 | chr5:112438094-112438174 | Cplx2 |
| 10.437215 | up | AK082315 | chr2:14526818-14526877 | Cacnb2 |
| 8.340329 | up | BC051068 | chr2:135840255-135840314 | Plcb4 |
| 2.3684267 | up | NM_001081202 | chr4:98405084-98405143 | L1td1 |
| 3.6275789 | up | NM_145940 | chr11:109435199-109435140 | Wipi1 |
| 2.2790696 | up | XR_106099 | chr1:139826743-139826802 |  |
| 3.3916411 | up | AI464814 | chr1:166394061-166394120 |  |
| 41.0608902 | up | NM_134244 | chr13:22420557-22420616 | Vmn1r197 |
| 13.5298553 | up | NM_027943 | chr7:126630194-126630135 | Pdilt |
| 3.1143174 | up | NM_013699 | chr9:113875655-113878454 | Ubp1 |
| 2.6108779 | up | NM_172783 | chrX:157029014-157029073 | Phka2 |
| 3.0267844 | up | NR_002863 | chr19:59499715-59499656 | Emx2os |
| 17.4417718 | up |  | chr6:130009540-130009481 |  |
| 3.0900946 | up | NM_001099628 | chr12:4948845-4949984 | Atad2b |
| 2.750848 | up | NM_001034871 | chr17:28689407-28689466 | Clpsl2 |
| 4.5703391 | up | BX514619 | chr1_random:368261-368320 |  |
| 2.4271125 | up | NM_026644 | chr17:12412423-12412482 | Agpat4 |
| 5.0777276 | up | NM_197986 | chr6:34823772-34823831 | Tmem140 |
| 2.190404 | up |  | chr9:73023342-73023283 |  |
| 2.3316431 | up | NM_021534 | chr2:154412995-154412936 | Pxmp4 |
| 2.0972059 | up | NM_178892 | chr3:65358749-65358808 | Tiparp |
| 6.6091819 | up | XR_108174 |  | 0610042G04Rik |
| 2.3274562 | up | AK011851 | chr11:109241587-109241646 | Gna13 |
| 2.8489257 | up | NM_001205355 | chr3:84248102-84248043 | Fhdc1 |
| 12.1017401 | up |  | chr4:111479067-111479008 |  |
| 2.4766517 | up | NM_023852 | chr13:110850583-110850524 | Rab3c |
| 5.6186051 | up | NM_001081292 | chr7:28441583-28441524 | Map3k10 |
| 2.4472051 | up | NM_001272024 | chr3:94977179-94977238 | Sema6c |
| 4.9320944 | up | XM_003945465 | chr10:83956560-83956619 | LOC101055743 |
| 2.1455016 | up | NM_145140 | chr17:46440245-46440186 | Abcc10 |
| 6.6752552 | up | NR_027821 | chr11:116537048-116537107 | 1810032O08Rik |
| 5.189811 | up | NM_001113379 | chr7:105650282-105650341 | Lrrc32 |
| 8.2782826 | up | XR_168420 | chr5:76131939-76131998 | LOC101055927 |
| 3.7867508 | up | NM_025994 | chr4:141414527-141414468 | Efhd2 |
| 3.2655817 | up | NM_007833 | chr10:96980233-96980292 | Dcn |
| 3.1964437 | up | NM_177864 | chr4:112058750-112058691 | Skint9 |
| 4.99622 | up | AK171382 | chr1:196853519-196853578 | A330023F24Rik |
| 21.7364585 | up | NM_001033263 | chr10:126530166-126530225 | Agap2 |
| 2.92225 | up | NM_028711 | chr17:43779147-43779088 | Slc25a27 |
| 2.7429321 | up | NM_021371 | chr5:131315985-131316044 | Caln1 |
| 6.3315424 | up | NM_001256055 | chr2:121866905-121867335 | Eif3j2 |
| 2.3766736 | up | NM_201639 | chr7:74877688-74877629 | Synm |
| 4.3388088 | up | NM_008690 | chr17:45700000-45700059 | Nfkbie |
| 2.2458331 | up | NM_145712 | chr9:15667651-15667592 | Mtnr1b |
| 2.6219188 | up | AK042408 | chr9:121768066-121768125 |  |
| 2.9575202 | up | BB713741 | chr13:68498549-68498608 |  |
| 2.1205948 | up | NM_001040106 | chr6:86951854-86951913 | Aak1 |
| 24.5656703 | up |  | chr1:006370828-006370887 |  |
| 2.9130182 | up | NM_011408 | chr11:82883791-82883850 | Slfn2 |
| 79.2137406 | up | NM_009463 | chr8:85821788-85821847 | Ucp1 |
| 5.732375 | up | NM_024452 | chr4:136096899-136096958 | Luzp1 |
| 3.0964003 | up | AK020094 | chr12:77636293-77636352 | 6330522J23Rik |
| 3.2175108 | up | NM_133919 | chr5:104284222-104284281 | Aff1 |
| 7.858613 | up | AK145429 | chr2:177809848-177809789 |  |
| 8.624919 | up | NM_009705 | chr12:80252972-80253031 | Arg2 |
| 9.0714212 | up | NM_009974 | chr8:97970106-97970047 | Csnk2a2 |
| 2.4427622 | up | NM_011056 | chr13:110741493-110741552 | Pde4d |
| 2.5810455 | up | NM_024450 | chr19:44289995-44290054 | Scd3 |
| 22.1320272 | up | AK016455 | chr11:75403290-75403231 |  |
| 2.4490021 | up | NM_145987 | chr4:141170338-141170279 | Tmem82 |
| 2.6254787 | up | NM_001286653 | chr17:44176406-44176465 | Rcan2 |
| 5.1365312 | up | AK013548 | chr2:117123888-117123829 | Rasgrp1 |
| 5.5387315 | up | NM_001003917 | chr1:75184319-75184260 | Atg9a |
| 4.2192995 | up | AK161464 | chr7:107048764-107048705 | A930030B08Rik |
| 2.390238 | up | NM_024279 | chr8:12600663-12600722 | Spaca7 |
| 3.6631181 | up | AK047917 | chr14:121567393-121567452 |  |
| 24.5241988 | up | NM_028864 | chr6:38266384-38266325 | Zc3hav1 |
| 4.7947879 | up | NM_025465 | chr8:69000413-69000354 | Tma16 |
| 8.8462922 | up | NM_173864 | chr6:124575730-124575671 | Gm5077 |
| 4.9983364 | up | AK047962 | chr17:5168401-5168460 | LOC552902 |
| 2.9717567 | up | NM_026457 | chr14:75982821-75982762 | Spert |
| 2.8244677 | up | XM_486686 | chrX:57298421-57298362 | Gm5637 |
| 5.734023 | up | S74315 | chr18:6374095-6374154 |  |
| 2.2852912 | up |  |  |  |
| 3.1064254 | up | XR_105445 | chr11:104155645-104155586 | Gm10844 |
| 27.2620661 | up |  |  |  |
| 3.34668 | up | NM_023835 | chr7:111448558-111448499 | Trim12a |
| 4.152948 | up | NM_001163462 | chr5:108071566-108071625 | Rpap2 |
| 12.8031988 | up | BB237529 | chr10:047467268-047467209 |  |
| 2.0732871 | up | NM_175770 | chr18:37802400-37802341 | Taf7 |
| 7.5954635 | up | NM_001081047 | chr4:133784957-133784898 | Cnksr1 |
| 4.5875462 | up | NM_001159571 | chr5:137815646-137815705 | Ephb4 |
| 4.846432 | up | AK013505 | chr19:24552642-24552701 |  |
| 2.5452864 | up | NM_146112 | chr1:89347288-89347347 | Gigyf2 |
| 6.352826 | up | NM_011019 | chr15:6763745-6763686 | Osmr |
| 16.0518937 | up | NM_007569 | chr10:96085358-96085417 | Btg1 |
| 3.2899895 | up | NM_030241 | chr5:124897355-124900704 | Setd8 |
| 2.1701331 | up | NM_172670 | chr2:92205359-92205300 | Gyltl1b |
| 2.7469533 | up | XR_141934 | chr1:183158554-183158613 | A430110L20Rik |
| 7.5261571 | up | NM_001005341 | chr11:86750364-86750305 | Ypel2 |
| 9.9204977 | up | AK220476 | chr9:64748424-64748483 | Dennd4a |
| 6.2400669 | up | NM_007720 | chr9:120003941-120004000 | Ccr8 |
| 11.6600203 | up |  | chr9:069335473-069335532 |  |
| 4.8139662 | up | NM_026163 | chr16:16272648-16272707 | Pkp2 |
| 2.8445886 | up | NM_009924 | chr4:135476052-135476111 | Cnr2 |
| 12.7758768 | up | NM_027834 | chr17:41013364-41013305 | 9130008F23Rik |
| 3.5426192 | up | NM_001033168 | chr18:35890459-35890518 | 1700066B19Rik |
| 2.1953059 | up | NM_025593 | chr7:148658045-148657986 | Polr2l |
| 2.9488471 | up | NM_027381 | chr10:80788877-80788936 | Cactin |
| 5.1377547 | up | NM_144853 | chr16:85456636-85456577 | Cyyr1 |
| 3.9898901 | up | AK020016 | chr11:32599486-32599545 | 5830420C07Rik |
| 2.2663222 | up | NM_207030 | chr6:132906989-132906930 | Tas2r131 |
| 2.199765 | up |  | chr19:3808776-3808835 | Suv420h1 |
| 5.8362913 | up | NM_011932 | chr3:137595763-137595704 | Dapp1 |
| 9.1389435 | up | NM_001195049 | chrX:140226257-140226316 | Pak3 |
| 2.3537498 | up | NM_008131 | chr1:155756765-155756824 | Glul |
| 2.9458742 | up | NM_175481 | chr9:42328629-42328570 | Grik4 |
| 2.082124 | up | AK040705 | chr11:61681355-61681296 | A530017D24Rik |
| 2.2429229 | up | NM_009449 | chr6:145569930-145569989 | Tuba3b |
| 3.138319 | up | NM_172796 | chr11:82793871-82793812 | Slfn9 |
| 4.7086566 | up | NM_183160 | chr19:24754076-24754135 | Tmem252 |
| 31.413657 | up | NM_001081269 | chr8:26825091-26825150 | Whsc1l1 |
| 4.0402848 | up | NM_023114 | chr9:46041193-46041134 | Apoc3 |
| 2.035868 | up | XM_003945560 | chr16:36875665-36875724 | LOC101055680 |
| 2.0427731 | up | NM_147003 | chr11:73858019-73857960 | Olfr139 |
| 5.3642497 | up | NM_183138 | chr6:83352802-83352743 | Tet3 |
| 12.9513672 | up | NM_010732 | chr1:134836381-134836440 | Lrrn2 |
| 6.6976547 | up | NM_146153 | chr4:125855498-125855439 | Thrap3 |
| 4.7569043 | up | NM_001042534 | chr6:72512913-72512972 | Capg |
| 3.0931569 | up | NM_010117 | chr11:32109646-32109587 | Rhbdf1 |
| 6.0205421 | up | NM_001242372 | chr3:88700413-88700472 | Gon4l |
| 11.1181358 | up | NM_028244 | chr17:32193829-32193888 | Rrp1b |
| 12.6122136 | up | NM_001170884 | chr9:88979714-88979655 | Trim43b |
| 2.3390359 | up | NM_031182 | chr16:4544770-4544711 | Tfap4 |
| 14.3767832 | up | NM_010559 | chr3:89674113-89674054 | Il6ra |
| 4.1430407 | up |  | chr2:073802377-073802318 |  |
| 10.308069 | up | XR_106099 | chr1:139826681-139826740 |  |
| 6.1114826 | up | NM_019985 | chr6:129355286-129355345 | Clec1b |
| 4.4255385 | up | EU568236 | chr6:068102545-068102604 |  |
| 6.2957402 | up | NM_019937 | chr3:65760878-65760819 | Ccnl1 |
| 3.5622693 | up | NM_007751 | chr7:148084911-148084852 | Cox8b |
| 4.8729634 | up |  | chr8:093648502-093648561 |  |
| 2.2161139 | up | NM_026676 | chr17:25379615-25379674 | Tsr3 |
| 2.6792446 | up | NM_181395 | chr12:30700635-30700694 | Pxdn |
| 4.0361422 | up | NM_001081365 | chr18:36552965-36553024 | Cystm1 |
| 3.4682945 | up |  | chr10:085312833-085312892 |  |
| 3.2376858 | up | NM_011790 | chr9:108505377-108505318 | Arih2 |
| 2.5658871 | up | NM_001163539 | chrX:83136656-83136715 | 5430427O19Rik |
| 2.1507564 | up | AK015254 | chr9:117790977-117790918 |  |
| 3.2502732 | up | NM_172393 | chr10:43670506-43670447 | Aim1 |
| 12.2808312 | up | NM_001081361 | chr1:186610771-186610712 | Marc1 |
| 3.1057592 | up | NM_001282120 | chr11:29445083-29445142 | Mtif2 |
| 6.8916465 | up | S74547 | chr6:68253070-68253129 |  |
| 2.1576577 | up | NM_145503 | chr19:45101463-45101522 | Lzts2 |
| 12.2004639 | up |  | chr7:123689201-123689260 |  |
| 23.1421051 | up | NM_010426 | chr8:123611944-123612003 | Foxf1 |
| 3.6294361 | up | NM_001165941 | chr2:14917568-14917509 | Nsun6 |
| 3.4253675 | up | NM_138311 | chr6:115900157-115900216 | H1foo |
| 2.2040867 | up | NM_001172160 | chr2:140484324-140484265 | Flrt3 |
| 2.8156519 | up | NM_001037737 | chr3:95301081-95301140 | Arnt |
| 6.897832 | up |  | chr16:052869142-052869083 |  |
| 3.2227092 | up | NM_027444 | chr16:50202678-50202619 | Bbx |
| 3.3841394 | up | AK078921 | chr4:135905891-135905950 | 9130020K20Rik |
| 2.3807729 | up | NM_001033173 | chr7:128791876-128791817 | Usp31 |
| 7.7358976 | up | AK143198 | chr10:41967347-41967288 | Foxo3 |
| 3.4295873 | up | AK053112 | chr1:74036446-74036387 | Tns1 |
| 9.4675616 | up | NM_008534 | chr1:173523614-173523555 | Ly9 |
| 3.9543758 | up | XR_140478 | chr1:174599174-174599165 | Gm2710 |
| 2.3434508 | up | NM_008348 | chr9:45062296-45062237 | Il10ra |
| 5.7731271 | up | NM_145416 | chr9:21077983-21077924 | Kri1 |
| 2.4435828 | up | NM_001168502 | chr17:37147440-37147499 | Zfp57 |
| 2.0646697 | up | NM_026002 | chr15:34066470-34066529 | Mtdh |
| 3.2888883 | up | NM_009289 | chr19:47716708-47716767 | Slk |
| 4.4872031 | up |  | chr12:086781607-086781666 |  |
| 2.7299887 | up |  | chr4:133476548-133476607 |  |
| 2.5124485 | up | NM_025976 | chr16:13702719-13702778 | Bfar |
| 8.035891 | up | NM_008361 | chr2:129190406-129190347 | Il1b |
| 5.1748154 | up | AK141640 | chr1:97227735-97227794 | BC055308 |
| 3.7495893 | up | NM_001025576 | chr2:76892655-76887778 | Ccdc141 |
| 3.1975214 | up | NM_018738 | chr11:58020927-58020986 | Igtp |
| 17.0810789 | up | NM_198666 | chr5:14911400-14911341 | Gm9758 |
| 2.8718259 | up | NM_029494 | chr7:99985062-99985121 | Rab30 |
| 2.3431436 | up | NM_178782 | chrX:45737228-45737287 | Bcorl1 |
| 8.4040363 | up | NM_023045 | chr14:71054144-71054085 | Xpo7 |
| 100.2077584 | up | NM_001285436 | chr11:118357725-118357666 | Rbfox3 |
| 2.0243649 | up |  | chr6:069961196-069961137 |  |
| 2.4284733 | up |  | chr11:053599044-053598985 |  |
| 3.6216748 | up | AK086846 | chr2:32083770-32083829 | E030004H24Rik |
| 2.2836095 | up | NM_198862 | chr11:69636700-69636641 | Nlgn2 |
| 5.7827014 | up | NM_001105180 | chr7:92091726-92091667 | Vmn2r65 |
| 5.0534663 | up | NM_013532 | chr10:51216160-51216219 | Lilrb4 |
| 2.4181545 | up | NM_177588 | chr2:21136476-21136535 | Thnsl1 |
| 8.3540936 | up | NR_028578 | chr7:139509811-139509870 | Gm10584 |
| 2.2051615 | up |  | chr4:124476658-124476717 |  |
| 11.8894156 | up | NM_001252292 | chr6:30692160-30692219 | Mest |
| 3.1612147 | up |  | chr14:59497730-59497671 |  |
| 31.2134907 | up | NM_028180 | chr15:99762052-99761993 | 1700030F18Rik |
| 2.2602306 | up | NM_019632 | chr2:148521154-148521095 | Napb |
| 3.598002 | up | AK172643 | chr5:108550644-108550585 | Tmed5 |
| 3.0529626 | up | NM_008959 | chr13:67099000-67099059 | Ptdss1 |
| 3.4800798 | up | NM_009723 | chr6:113695898-113695839 | Atp2b2 |
| 2.3972638 | up | NM_001205234 | chr19:6532125-6532184 | Nrxn2 |
| 2.28635 | up | NM_007398 | chr2:163552640-163552581 | Ada |
| 5.8629226 | up | NM_001113533 | chr17:13159728-13159669 | Wtap |
| 2.8522562 | up | NM_009653 | chrX:147004993-147005052 | Alas2 |
| 2.1250047 | up | NM_030206 | chr11:116507555-116507496 | Cygb |
| 3.9254727 | up | NM_001035532 | chr4:57907544-57907603 | Akap2 |
| 3.3506454 | up | AK082151 | chr15:84882380-84882439 | Fam118a |
| 2.5330525 | up | NM_010786 | chr10:117126069-117126010 | Mdm2 |
| 4.5284763 | up | NM_176930 | chr12:45699758-45699817 | Nrcam |
| 8.015466 | up | AK090034 | chr14:43967961-43968020 | Gm3161 |
| 2.7595825 | up | AK157043 | chr4:74104716-74104775 |  |
| 5.5755101 | up | AK084195 | chr9:8104676-8104617 |  |
| 2.1612665 | up | NM_016979 | chrX:75007747-75007688 | Prkx |
| 3.944743 | up | NM_001039000 | chr10:126662856-126662797 | Kif5a |
| 2.484605 | up | CB248850 | chrX:58438815-58438874 |  |
| 7.0874959 | up |  | chr8:123244129-123244070 |  |
| 3.9838933 | up | XR_168441 | chr8:3674177-3674118 | BB094273 |
| 2.4847936 | up | NM_001081000 | chr16:18253535-18253594 | Trmt2a |
| 5.8611185 | up | NM_029816 | chr10:75923795-75923854 | 2610028H24Rik |
| 12.256898 | up | NM_008006 | chr3:37247934-37295153 | Fgf2 |
| 2.7620668 | up | NM_030730 | chr9:106590475-106590416 | Rad54l2 |
| 2.8889089 | up |  | chr13:045641798-045641857 |  |
| 2.6187053 | up | NM_146201 | chr7:134381189-134381248 | Zfp553 |
| 4.1680512 | up | AK149472 | chr19:4064579-4064520 |  |
| 2.8156148 | up | NM_007469 | chr7:20274890-20274831 | Apoc1 |
| 3.1533297 | up | NM_021568 | chr10:76224663-76224604 | Pcbp3 |
| 5.3491031 | up | NM_172276 | chr5:130056705-130060319 | Sfswap |
| 2.3987242 | up | XR_107623 | chr4:118627495-118627436 |  |
| 4.2164154 | up | NM_008539 | chr8:81880293-81880234 | Smad1 |
| 2.353466 | up | NM_007949 | chr7:19980982-19981041 | Ercc2 |
| 2.0314924 | up | NM_173761 | chr2:180639143-180639084 | Ythdf1 |
| 80.0417886 | up | NM_008491 | chr2:32240258-32240199 | Lcn2 |
| 42.8492783 | up |  | chr16:70382352-70382293 |  |
| 2.3202616 | up |  | chr4:063048355-063048414 |  |
| 2.3240724 | up | NM_145076 | chr6:37915817-37915876 | Trim24 |
| 4.8667815 | up |  | chr18:035790941-035791000 |  |
| 2.0353882 | up | NR_040344 | chr8:108662559-108662618 | 1810019D21Rik |
| 2.651994 | up | NM_001276316 | chrX:20260246-20260305 | Uba1 |
| 3.9875538 | up | NM_031159 | chr6:122527967-122527908 | Apobec1 |
| 2.1795692 | up | NM_053195 | chr2:145467614-145467673 | Slc24a3 |
| 3.0520255 | up | AK048763 | chr5:113389708-113389649 | Adrbk2 |
| 5.927349 | up | NM_013562 | chr12:40929986-40929927 | Ifrd1 |
| 3.3257207 | up | AK042442 | chr10:14376636-14376577 | 9030203C11Rik |
| 5.6561328 | up | DV053137 |  | AI790442 |
| 3.0260007 | up | NM_025788 | chr8:87194369-87194350 | Nacc1 |
| 7.2974327 | up | NM_011817 | chr13:51943733-51943792 | Gadd45g |
| 2.7433538 | up | NM_026058 | chr8:4523988-4524047 | Cers4 |
| 2.0089992 | up | NM_057172 | chr3:151896691-151896750 | Fubp1 |
| 18.9125829 | up | NM_026290 | chr17:28675824-28675883 | Armc12 |
| 2.2239532 | up | NM_177321 | chr12:60209686-60209745 | Mia2 |
| 2.633743 | up | NM_029499 | chr19:11500973-11501032 | Ms4a4c |
| 3.5105029 | up | X05545 | chr10:8320766-8320825 |  |
| 7.1560343 | up | NM_009404 | chr17:57247062-57247121 | Tnfsf9 |
| 3.7881315 | up | NM_023815 | chr2:166621236-166621295 | Trp53rk |
| 83.4947511 | up | NM_203492 | chr7:150949710-150949651 | Mrgprg |
| 17.1753548 | up |  | chr4:125047375-125047434 |  |
| 11.2051884 | up | NM_025778 | chr6:134388265-134388324 | Bcl2l14 |
| 13.2503568 | up | NM_011562 | chr9:110843158-110843099 | Tdgf1 |
| 7.7252322 | up | NM_001177319 | chr2:84281081-84281022 | Tfpi |
| 11.6629594 | up | AK017575 | chr1:78591675-78591616 | 5730419F03Rik |
| 11.7994965 | up | NM_178630 | chr6:34807807-34807866 | Agbl3 |
| 2.3244184 | up | NM_008104 | chr13:41196806-41196796 | Gcm2 |
| 2.1711148 | up | NM_001040400 | chr3:133126810-133126751 | Tet2 |
| 2.8312124 | up | BC002306 | chr16:20722256-20722314 | Polr2h |
| 5.2030606 | up | AK079655 | chr16:84974213-84974154 |  |
| 3.7236668 | up | AF037047 | chr6:65279004-65279063 | Gm8479 |
| 3.785524 | up | NM_027427 | chr11:83320160-83320219 | Taf15 |
| 2.1853923 | up | NM_001081352 | chr13:76325331-76325390 | Ttc37 |
| 4.4203052 | up |  | chrX:099334419-099334478 |  |
| 3.2197117 | up | NM_029274 | chr7:31353965-31353906 | Kmt2b |
| 4.3075093 | up |  | chr4:145954351-145954410 |  |
| 5.7416007 | up | BC002231 | chr2:130104989-130105049 | Nop56 |
| 3.4517393 | up | NM_013764 | chr6:83430316-83430257 | Dguok |
| 13.6617728 | up | NM_001081376 | chr4:151764244-151764303 | Chd5 |
| 3.0577342 | up | NM_001085528 | chr4:88400981-88401040 | Gm13271 |
| 16.0972908 | up | NM_011803 | chr13:5869371-5869430 | Klf6 |
| 4.3174845 | up | NM_001163689 | chr7:148645928-148645987 | Pnpla2 |
| 2.8278972 | up | AK014684 | chr15:76944221-76944162 | 4833412C15Rik |
| 6.0053503 | up | NM_153396 | chr6:120979738-120979679 | Mical3 |
| 4.2000398 | up | NM_024193 | chr2:130104982-130105041 | Nop56 |
| 3.5976329 | up | NM_028881 | chr3:90067950-90068009 | Crtc2 |
| 2.3171348 | up | AK034200 | chr13:25033208-25033267 |  |
| 2.3376703 | up | NM_146114 | chr2:3370687-3370746 | Dclre1c |
| 3.8202483 | up | NM_178897 | chr5:130760952-130761011 | Tyw1 |
| 5.6869614 | up | NR_104450 | chr12:110809678-110809737 | Meg3 |
| 3.0724829 | up | NR_003953 | chr5:24154568-24154627 | 1700022A21Rik |
| 9.483097 | up | NM_172659 | chr2:26876950-26876891 | Slc2a6 |
| 2.8176777 | up | NM_018807 | chr2:153054295-153054236 | Plagl2 |
| 2.1842496 | up | XM_978251 | chr8:72411753-72411694 | Yjefn3 |
| 5.8624959 | up | NM_183000 | chr5:23922359-23922418 | Asic3 |
| 3.8036107 | up | NM_133853 | chr3:103819715-103819656 | Magi3 |
| 2.9373415 | up | AK164147 | chr8:125984097-125984156 | Def8 |
| 3.8008621 | up | NM_023336 | chr2:27306014-27305955 | Brd3 |
| 2.7667911 | up |  | chr9:043828913-043828972 |  |
| 3.2772779 | up |  | chrY:002082146-002082205 |  |
| 3.2934567 | up | AK042931 | chr5:108548393-108548334 | Tmed5 |
| 2.1474298 | up | NM_175016 | chr5:114574006-114573947 | Alkbh2 |
| 2.4360977 | up | NM_019726 | chr11:69729964-69730023 | Gps2 |
| 4.3562523 | up | AK014606 | chr7:4988085-4988144 | 4632433K11Rik |
| 2.6942141 | up | NM_001024846 | chr11:49030294-49030353 | Zfp62 |
| 6.7952268 | up | NM_172053 | chr13:70867207-70867148 | Adamts16 |
| 2.4332524 | up | NM_021366 | chr7:71082913-71082854 | Klf13 |
| 2.011637 | up | NM_025427 | chr14:79688776-79688717 | Rgcc |
| 2.5374898 | up | NM_144800 | chr15:58774968-58774909 | Mtss1 |
| 93.42997 | up | NM_017370 | chr8:112099104-112099045 | Hp |
| 6.4899439 | up | NM_173018 | chr9:59717487-59717546 | Myo9a |
| 4.3313513 | up | NM_011952 | chr7:133908255-133908314 | Mapk3 |
| 5.3148538 | up |  | chr17:073802434-073802375 |  |
| 2.7111154 | up |  | chr11:059391773-059391714 |  |
| 2.4786559 | up | NM_008268 | chr11:96167130-96167189 | Hoxb5 |
| 4.8762 | up | NR_045884 | chr12:3859419-3859360 | Dnmt3aos |
| 3724.715451 | up | NM_011314 | chr7:54009622-54009681 | Saa2 |
| 6.4106427 | up | NM_139221 | chr8:23015980-23015921 | Defb11 |
| 3.3538299 | up | AK141316 | chr5:123599893-123599952 | Setd1b |
| 2.0280103 | up | NM_145528 | chr2:91514850-91514791 | Atg13 |
| 15.6780867 | up | NM_001033713 | chr7:74378810-74378751 | Mef2a |
| 2.3601933 | up | AK076764 | chr8:41228531-41228590 |  |
| 4.919693 | up | NM_013680 | chrX:20437700-20437641 | Syn1 |
| 5.3283413 | up | NM_010704 | chr4:101452666-101452725 | Lepr |
| 43.4164542 | up | NM_001136259 | chr8:77583100-77583159 | Tom1 |
| 4.344873 | up |  | chr3:062977423-062977364 |  |
| 2.9224461 | up | AK149336 | chr16:21546331-21546390 | Vps8 |
| 25.1582994 | up |  | chr14:045310204-045310145 |  |
| 15.5411138 | up | NM_183287 | chr16:17118599-17118540 | 2610318N02Rik |
| 2.2158014 | up | AK019121 | chr19:23210140-23210199 | 2410080I02Rik |
| 4.8418084 | up | NM_020015 | chrX:151523308-151523249 | Magea1 |
| 10.0437422 | up | AK045926 | chr2:84481933-84481874 | AI225934 |
| 4.7950229 | up | NM_173444 | chr1:60338707-60338766 | Nbeal1 |
| 4.0910155 | up | NM_028748 | chr9:61803928-61803869 | Paqr5 |
| 15.9666285 | up | NM_001109973 | chr17:47952344-47952285 | Mdfi |
| 2.0576292 | up | NM_139117 | chr6:131314961-131314902 | Ybx3 |
| 6.5270948 | up | NM_001001738 | chr19:47969169-47969110 | Itprip |
| 2.788939 | up |  | chr19:012347564-012347623 |  |
| 4.1206902 | up | NM_146156 | chr4:133831670-133831611 | Pdik1l |
| 8.3785175 | up | NM_011302 | chrX:157232740-157232799 | Rs1 |
| 6.6746167 | up | AK138782 | chr17:45628468-45628527 |  |
| 3.9567639 | up |  | chr11:020346618-020346677 |  |
| 4.3368033 | up | XM_003946445 |  | LOC101056478 |
| 15.1352185 | up | NM_025759 | chr5:15128398-15129200 | Speer4d |
| 10.1413352 | up | AK005775 | chr11:5543813-5544933 | Ankrd36 |
| 2.2266163 | up | NM_025670 | chr16:4765674-4765615 | Cdip1 |
| 2.2637145 | up | NM_016887 | chr11:69781255-69781314 | Cldn7 |
| 11.8497195 | up | AK141540 | chr13:35097399-35097340 |  |
| 2.0278463 | up |  | chr1:185505847-185505788 |  |
| 3.1059067 | up | NM_207652 | chr14:76906896-76906955 | Tsc22d1 |
| 13.7710723 | up | NM_001277149 | chr4:8793852-8793911 | Chd7 |
| 3.005675 | up | NM_001271601 | chr15:89203198-89203257 | Ncaph2 |
| 2.9491363 | up | NM_010706 | chr7:29626555-29626614 | Lgals4 |
| 2.0721264 | up | NM_001164268 | chr16:34178442-34178383 | Kalrn |
| 4.2194033 | up | NM_176902 | chr11:116300285-116300345 | Ubald2 |
| 4.6263314 | up | NM_146114 | chr2:3371015-3371074 | Dclre1c |
| 2.0001616 | up | AK031665 | chr1:135269813-135269872 | Etnk2 |
| 3.6082818 | up | NM_029101 | chr15:82946332-82946273 | Rrp7a |
| 3.2585045 | up | NM_175498 | chr14:67535939-67535998 | Pnma2 |
| 7.6912709 | up | NM_178591 | chr8:32928559-32928500 | Nrg1 |
| 7.0110552 | up | NR_045765 | chr4:16193240-16193299 | A530072M11Rik |
| 3.5884579 | up | NM_027129 | chr2:131039190-131039249 | Ap5s1 |
| 6.9477556 | up |  | chr3:038091247-038091306 |  |
| 2.8230984 | up | NM_173002 | chr6:90332399-90332458 | Zxdc |
| 4.9600226 | up | NR_040271 | chrX:140598575-140598634 | A730046J19Rik |
| 9.7621059 | up | AJ277812 | chr6:043031576-043031517 |  |
| 5.4806789 | up | AK042822 | chr2:4709866-4709925 | Bend7 |
| 20.4247696 | up | NM_009220 | chrY_random:9329694-9329635 | Ssty1 |
| 7.7563075 | up | NM_027014 | chr2:150756868-150756927 | Gins1 |
| 2.6533519 | up | NM_001017426 | chr11:69212236-69212177 | Kdm6b |
| 4.3929193 | up | AK149714 | chr15:88529340-88529281 | Brd1 |
| 5.463112 | up | NM_178909 | chr11:17134613-17134672 | Wdr92 |
| 5.1215259 | up | AK088445 | chr12:52744933-52744874 | 5730526G10Rik |
| 4.1699324 | up | NM_010206 | chr8:26685072-26685131 | Fgfr1 |
| 5.3017127 | up |  | chr2:089037036-089036977 |  |
| 6.5799517 | up | NM_146477 | chr17:37222285-37222226 | Olfr90 |
| 2.3172585 | up | NM_146030 | chr11:101024072-101024013 | Plekhh3 |
| 2.0178139 | up | BU936515 | chr18:83275853-83275794 | 1700095A13Rik |
| 2.4898745 | up | NM_145501 | chr19:42196480-42196539 | Pi4k2a |
| 2.5751974 | up | NM_010171 | chr3:121437717-121437776 | F3 |
| 11.6138894 | up | AK163492 | chr4:120569114-120569173 | Rims3 |
| 2.0036687 | up | NM_198604 | chr6:125312878-125312819 | Plekhg6 |
| 3.9811466 | up | NM_028516 | chr1:185857094-185857035 | 1700056E22Rik |
| 7.2519215 | up | NM_172492 | chr6:119265233-119265174 | Lrtm2 |
| 2.8018894 | up | NM_001286604 | chr9:35024971-35024912 | Fam118b |
| 4.6241936 | up | NM_010901 | chr8:108615965-108616024 | Nfatc3 |
| 4.2143087 | up | NM_001033767 | chr18:60407406-60407465 | Gm4951 |
| 3.714468 | up | NM_010050 | chr12:91963294-91963235 | Dio2 |
| 7.3430428 | up | AK018050 | chr1:89693292-89693351 |  |
| 2.5232419 | up | NM_030180 | chr14:21368927-21368868 | Usp54 |
| 2.3617492 | up | NM_007719 | chr11:99005610-99005551 | Ccr7 |
| 2.5826164 | up | AK165339 | chr16:20738166-20738225 | Chrd |
| 2.1403014 | up | NM_007554 | chr14:47003450-47003391 | Bmp4 |
| 2.3065446 | up | NM_027354 | chr9:106252135-106252194 | Poc1a |
| 4.0424886 | up | NM_011671 | chr7:107647809-107647868 | Ucp2 |
| 3.2612352 | up | NM_001198984 | chr18:60974672-60974613 | Tcof1 |
| 3.2137551 | up | NM_013490 | chr19:3893815-3893874 | Chka |
| 3.1427511 | up | AK142804 | chr8:111266627-111266686 | D8Ertd158e |
| 4.6821463 | up | NM_018734 | chr3:142236032-142236091 | Gbp3 |
| 21.8303764 | up | NM_178793 | chr18:66230936-66230877 | Ccbe1 |
| 2.9379768 | up | NM_011498 | chr6:108616309-108616368 | Bhlhe40 |
| 2.1210057 | up | NM_033325 | chr14:70095254-70095313 | Loxl2 |
| 4.9818929 | up | NM_013646 | chr9:69226774-69226833 | Rora |
| 11.5289323 | up | AK046953 | chr5:71352334-71352275 | Gabra2 |
| 73.4535341 | up | NM_001033960 | chr2:37351140-37351199 | Rabgap1 |
| 2.4537819 | up | NM_009089 | chr11:69547971-69547912 | Polr2a |
| 2.9748419 | up | NM_172513 | chr1:58584253-58584194 | Fam126b |
| 4.5960911 | up | NM_030249 | chr3:104806798-104806739 | Cttnbp2nl |
| 9.1721948 | up | XM_484483 | chr15:75865599-75865658 | K230010J24Rik |
| 2.4349666 | up | NM_010512 | chr10:87395924-87395983 | Igf1 |
| 2.008381 | up | NM_001002239 | chr18:75162851-75162910 | Rpl17 |
| 9.2186494 | up | NM_178081 | chr1:52717711-52717652 | Mfsd6 |
| 5.7659271 | up | NM_001281472 | chr11:48759689-48759630 | Psme2b |
| 9.2103405 | up |  | chr17:089675670-089675729 |  |
| 2.1632959 | up | AK031375 | chr7:103358859-103358918 | Tenm4 |
| 6.1359767 | up | NR_045141 | chr6:77330313-77330254 | 6330415B21Rik |
| 2.6466898 | up | AK160591 | chr15:77757112-77757053 | Txn2 |
| 2.1739767 | up | NM_028943 | chr3:131025930-131025871 | Sgms2 |
| 2.1076043 | up | NR_002840 | chr1:162968608-162968667 | Gas5 |
| 3.7325951 | up | NM_020575 | chr2:60085955-60086014 | March7 |
| 7.1196682 | up | XR_141214 | chr14:64766757-64766698 | 5330427O13Rik |
| 2.1830168 | up | NM_145475 | chr15:85970832-85970773 | Cerk |
| 2.1554164 | up | AK052941 | chr6:115626966-115627025 |  |
| 14.6249826 | up |  | chr15:57793758-57793817 | Tbc1d31 |
| 5.0428839 | up | NM_001252580 | chr2:126584484-126584543 | Usp8 |
| 7.5703867 | up | NR_015605 | chr9:46721808-46728651 | 2900052N01Rik |
| 3.3467296 | up | NM_133240 | chr2:164618328-164618269 | Acot8 |
| 4.0012534 | up | XM_003945430 | chr8:20015825-20015766 | LOC101056104 |
| 2.6601729 | up | NM_027990 | chr2:49804191-49804250 | Lypd6b |
| 2.0477499 | up | NM_145147 | chr5:110533058-110532999 | Gtpbp6 |
| 2.9638717 | up | NM_019763 | chr4:141023878-141023819 | Spen |
| 3.0547432 | up | NM_011753 | chr9:20242832-20242773 | Zfp26 |
| 3.3095299 | up | NM_001177503 | chr12:81825086-81825145 | Plekhd1 |
| 2.6190598 | up | NM_001252457 | chr17:35390546-35390605 | Ddx39b |
| 3.595666 | up | NM_011772 | chr10:128069663-128069604 | Ikzf4 |
| 3.2417178 | up | NM_026152 | chr19:42144788-42144847 | Hoga1 |
| 2.8621554 | up | AK046698 | chr6:93867818-93867759 | B430316J06Rik |
| 4.8045256 | up | NM_001177630 | chr2:20681212-20681271 | Etl4 |
| 124.2323854 | up | NM_008176 | chr5:91321845-91321904 | Cxcl1 |
| 3.3706604 | up | NM_030705 | chr7:91029065-91029006 | Mesdc1 |
| 3.6956215 | up | NM_182809 | chr7:85445967-85445908 | Ntrk3 |
| 5.6130581 | up | NM_178087 | chr9:58066408-58066349 | Pml |
| 14.0052031 | up | NM_133974 | chr9:123081462-123081403 | Cdcp1 |
| 2.7584314 | up | NM_207275 | chr2:143899652-143899711 | Banf2 |
| 2.4567215 | up | NM_026931 | chr8:25549107-25549048 | 1810011O10Rik |
| 4.5908777 | up | NM_008528 | chr19:41003485-41003426 | Blnk |
| 3.0355977 | up | NM_175201 | chr4:44151692-44151633 | Rnf38 |
| 2.1466307 | up | NM_013892 | chrX:7500267-7500326 | Pcsk1n |
| 3.1377578 | up | NM_001163159 | chr16:32472042-32472101 | Pcyt1a |
| 5.1405382 | up | NM_008958 | chr4:116787255-116787314 | Ptch2 |
| 3.2989959 | up | AK133172 | chr2:180322448-180322389 | LOC552877 |
| 2.2659063 | up |  | chr5:105588579-105588520 |  |
| 6.7104518 | up | NM_001163774 | chr7:134015428-134015105 | Taok2 |
| 12.5310813 | up | NM_029152 | chr12:34086067-34086126 | Efcab10 |
| 2.1675024 | up | NM_026403 | chr14:56374169-56374228 | Nop9 |
| 42.8298702 | up | NM_172495 | chr10:30366694-30366635 | Ncoa7 |
| 2.3091947 | up | AK084170 | chr9:41420788-41420847 |  |
| 2.8118351 | up | NM_177045 | chr4:108306634-108306693 | Cc2d1b |
| 3.4613288 | up | NM_001163103 | chr12:77539089-77539378 | Ppp1r36 |
| 4.6735732 | up | NM_009072 | chr12:16994437-16994496 | Rock2 |
| 2.2096124 | up | NM_001177471 | chr8:22040356-22040297 | Gm15056 |
| 3.9043544 | up | AK081327 | chrX:48363026-48362967 |  |
| 5.4233741 | up |  | chr12:114899984-114899925 |  |
| 3.3853852 | up | NM_010439 | chr5:149861405-149861346 | Hmgb1 |
| 2.5603198 | up |  |  |  |
| 2.5905379 | up | NM_145934 | chr17:56136717-56136658 | Stap2 |
| 5.6608567 | up | NM_144491 | chr11:74994823-74994416 | Dph1 |
| 3.1503111 | up | NM_023835 | chr7:111452818-111452759 | Trim12a |
| 5.874231 | up |  | chr5:045701575-045701516 |  |
| 2.3092302 | up | NM_153550 | chr16:35695262-35695203 | Dirc2 |
| 30.4412438 | up | NM_009841 | chr18:36885028-36884969 | Cd14 |
| 7.4433548 | up | NM_025635 | chr10:72131730-72131789 | Zwint |
| 4.9625798 | up | NM_007670 | chr4:88952335-88952276 | Cdkn2b |
| 2.1235615 | up | NM_198023 | chr12:112347115-112348043 | Rcor1 |
| 8.6820498 | up |  | chr1:092524453-092524512 |  |
| 2.6017764 | up | AK044604 | chr10:62803455-62803514 |  |
| 3.341044 | up | BC049953 | chr15:75584848-75584843 | Zc3h3 |
| 2.410861 | up | NM_147034 | chr7:114180510-114180569 | Olfr713 |
| 12.1096352 | up | NM_009519 | chr7:106001985-106002045 | Wnt11 |
| 2.8648083 | up | XR_168559 | chr17:51896681-51896622 | LOC101056045 |
| 2.5452211 | up | AK044527 | chr16:34953958-34954017 | Mylk |
| 7.4575027 | up | NM_001034102 | chr14:52331665-52331606 | Gm5800 |
| 3.3387522 | up | NM_025602 | chr10:105282445-105282504 | Ccdc59 |
| 14.6703562 | up | AB041803 | chr6:31115489-31115484 | AB041803 |
| 15.9385668 | up | XR_141327 | chr17:86897132-86897073 |  |
| 11.0820264 | up | NM_001242941 | chr14:5266722-5266781 | Gm3317 |
| 2.1541624 | up | NM_001205339 | chr19:15979681-15979622 | Psat1 |
| 10.1547189 | up | NM_008256 | chr3:98114596-98114655 | Hmgcs2 |
| 7.3882844 | up | NM_001167730 | chr6:112570681-112570622 | Rad18 |
| 11.9144628 | up | AK039451 | chr19:46957956-46957897 | A330044H09 |
| 3.8678745 | up | NM_008397 | chr2:71694756-71694815 | Itga6 |
| 3.186196 | up | NM_026396 | chr15:10406014-10405955 | Brix1 |
| 2.8855673 | up | NM_001271540 | chr7:51893776-51893717 | Myh14 |
| 2.9754255 | up | NM_026295 | chr18:80604787-80604728 | Ctdp1 |
| 3.2750052 | up | NM_028149 | chr11:97947351-97947292 | Fbxl20 |
| 4.3768884 | up | AK167257 | chr18:25449350-25449409 | AW554918 |
| 24.0731729 | up | NM_172612 | chr15:98500378-98500319 | Rnd1 |
| 3.168612 | up | NM_172938 | chr10:42680516-42680575 | Scml4 |
| 26.1773491 | up | AK049871 | chr7:77418275-77418216 | LOC624549 |
| 6.209676 | up | NM_009058 | chr2:28408531-28408590 | Ralgds |
| 4.7117379 | up | XR_168557 | chr17:46543945-46543886 | Gm9706 |
| 2.8378139 | up | NM_027547 | chr6:65886216-65886275 | Prdm5 |
| 2.3462764 | up | NM_153175 | chr6:48651725-48651666 | Gimap6 |
| 23.6251503 | up | AK167816 | chr3:94901187-94901128 | Pip5k1a |
| 2.8644193 | up | NM_172381 | chr4:58874471-58874412 | AI314180 |
| 2.6883482 | up | NM_145215 | chr5:135487978-135488038 | Abhd11 |
| 6.4700103 | up |  | chr11:58739630-58739689 | Btnl10 |
| 2.0895873 | up | NM_177323 | chr5:23326056-23326115 | Rint1 |
| 2.1698029 | up | NM_008990 | chr7:20302077-20302018 | Pvrl2 |
| 4.5490459 | up | NM_133838 | chr2:119916176-119916117 | Ehd4 |
| 4.1110058 | up | NM_146148 | chr4:104499334-104499275 | C8a |
| 2.3884721 | up | AU018847 | chr9:18126719-18126778 | D9Ertd720e |
| 3.5203874 | up | NM_133659 | chr16:95582013-95581954 | Erg |
| 2.4028623 | up | NM_029441 | chr8:119098386-119098327 | Cdyl2 |
| 7.3365053 | up | NM_001004468 | chr7:137908170-137908229 | Tacc2 |
| 2.079414 | up | NM_175121 | chr15:96518550-96518491 | Slc38a2 |
| 22.5268424 | up | NM_133239 | chr1:141131423-141131364 | Crb1 |
| 9.705935 | up | NM_001005488 | chr7:114958961-114959020 | Olfr467 |
| 3.2694263 | up | NM_001256096 | chr5:136508675-136508734 | Dtx2 |
| 3.4687263 | up | NM_009946 | chr13:54481316-54481375 | Cplx2 |
| 35.0394761 | up |  | chr14:044152768-044152827 |  |
| 4.919039 | up | AK080218 | chr3:94807256-94807315 |  |
| 2.2239556 | up | NM_198107 | chr10:79357514-79357455 | Med16 |
| 3.5881821 | up |  | chr8:100400382-100400441 |  |
| 14.7341875 | up | BC119495 | chr6:145173657-145173598 | Kras |
| 3.3318189 | up | NM_027133 | chr2:74360333-74360274 | Lnp |
| 4.7408574 | up |  | chr18:004011172-004011231 |  |
| 2.0639099 | up | NM_172443 | chr11:119008527-119008468 | Tbc1d16 |
| 2.611916 | up | NM_146698 | chr19:12758013-12758072 | Olfr1443 |
| 2.76221 | up | AK157781 | chr15:13101015-13100956 |  |
| 3.5472394 | up | NM_029983 | chr2:156698670-156698658 | Sla2 |
| 3.2407511 | up | NM_007789 | chr8:72619014-72618955 | Ncan |
| 5.6798176 | up | NM_026866 | chr1_random:48425-48366 | Disp1 |
| 3.0420973 | up | NM_019648 | chr10:93321764-93321705 | Metap2 |
| 3.9787344 | up | NM_133733 | chr9:40591652-40591711 | Clmp |
| 21.2574111 | up | NR_040342 | chr18:45843256-45843197 | A330093E20Rik |
| 4.106502 | up | NM_182939 | chr6:100814708-100815087 | Ppp4r2 |
| 2.0235134 | up | XM_003086464 | chr5:105011519-105011578 | LOC100046091 |
| 6.8873453 | up | NM_175188 | chr8:68992804-68992863 | March1 |
| 2.8005778 | up |  | chr7:077634799-077634858 |  |
| 10.5143029 | up |  |  |  |
| 2.3797483 | up | NM_001195531 | chr8:87591547-87591488 | Gm5741 |
| 2.1897036 | up | NM_145566 | chr5:138104107-138104048 | Agfg2 |
| 14.4466752 | up | NM_010086 | chr8:41767082-41767141 | Adam24 |
| 4.6655365 | up | NM_008514 | chr6:134404782-134404723 | Lrp6 |
| 8.2878414 | up | NM_030704 | chr5:116859122-116859063 | Hspb8 |
| 2.8162623 | up | NM_001081061 | chr13:100835960-100835901 | Bdp1 |
| 3.1613374 | up | NM_199322 | chr10:80257008-80257067 | Dot1l |
| 2.1456002 | up |  |  |  |
| 3.5446707 | up | NM_001009819 | chr4:128446483-128446542 | A3galt2 |
| 2.5299778 | up | NM_153075 | chr2:121220178-121220119 | Catsper2 |
| 7.7030111 | up | AK016863 | chr19:37541387-37541446 | 4933421H12Rik |
| 3.4676108 | up | NM_019986 | chr13:64287216-64287275 | Habp4 |
| 3.4649007 | up | NM_198664 | chr4:46617331-46617272 | Tbc1d2 |
| 3.3653954 | up |  | chr9:085581022-085580963 |  |
| 4.3797484 | up | NM_183428 | chr4:131482484-131482425 | Epb4.1 |
| 3.0042219 | up | NM_001099688 | chr11:72132859-72132918 | Fbxo39 |
| 8.4533489 | up | NM_001081345 | chr7:80660672-80660613 | Chd2 |
| 2.4873284 | up | NM_029999 | chr17:73288852-73288911 | Lbh |
| 2.268918 | up | NM_008517 | chr10:92947503-92947562 | Lta4h |
| 4.2039293 | up |  | chr7:080700120-080700061 |  |
| 4.0234109 | up | AK017287 | chr14:59840458-59840517 | Rcbtb1 |
| 3.5879008 | up | XM_001471750 | chr7:20080677-20080736 | Exoc3l2 |
| 4.8644173 | up | AK046648 | chr1:38392357-38392298 | Aff3 |
| 4.1039211 | up |  | chr15:097132952-097132893 |  |
| 2.1820074 | up | NM_152817 | chr17:75255836-75255895 | Ttc27 |
| 8.046731 | up | AK090155 | chr15:79086401-79086460 | Pick1 |
| 6.9629082 | up | NM_008654 | chr7:52778379-52778320 | Ppp1r15a |
| 2.8342374 | up | AK020767 | chr1:188576739-188576798 | A430105J06Rik |
| 2.9692921 | up | AK081485 | chr6:134037295-134037354 | LOC552909 |
| 3.202673 | up | XR_168498 | chr12:117518924-117518865 | Gm4132 |
| 9.015863 | up | NM_001081147 | chr6:112423745-112423686 | Oxtr |
| 4.5284552 | up | NM_015783 | chr4:155573708-155573649 | Isg15 |
| 5.4145547 | up | NM_007489 | chr7:120457261-120457320 | Arntl |
| 2.0162534 | up | NM_011104 | chr17:86568035-86568094 | Prkce |
| 22.4994854 | up | NM_198959 | chr4:129807520-129807461 | Hcrtr1 |
| 5.5140143 | up | AK132138 | chr9:58403560-58403619 |  |
| 2.4037569 | up | NM_001170561 | chr15:89118776-89118717 | Sbf1 |
| 24.5104659 | up | NM_183278 | chr14:35166894-35165215 | Fam25c |
| 4.8165896 | up | NM_026989 | chr3:157675803-157675744 | Srsf11 |
| 23.1909337 | up | NM_177866 | chr4:133767944-133767885 | Catsper4 |
| 3.0508579 | up | NM_172991 | chr5:3595331-3595272 | Rbm48 |
| 25.2090527 | up | NM_177191 | chr2:178080059-178080000 | Sycp2 |
| 4.2316139 | up | NM_001037809 | chr8:109079933-109079992 | Cdh3 |
| 5.945075 | up |  | chrX:13131806-13131747 | Cask |
| 5.5305789 | up | AK039021 | chr4:141093482-141093541 | Gm4123 |
| 2.5718479 | up | AK048710 | chr2:33874036-33873977 | C230014O12Rik |
| 3.7088083 | up | AK012274 | chr14:57286010-57285951 | 2700022O18Rik |
| 3.5279431 | up | NM_007702 | chr18:67527351-67527410 | Cidea |
| 2.3810006 | up | NM_011530 | chr17:34353116-34353175 | Tap2 |
| 3.7009817 | up | NM_177089 | chr8:26265153-26265094 | Tacc1 |
| 2.3833965 | up | NM_146083 | chr17:80600800-80600741 | Srsf7 |
| 2.1649519 | up |  | chr2:142402952-142403011 |  |
| 8.5419651 | up | AK079056 | chr3:97718922-97718981 | Sec22b |
| 2.3690325 | up | NM_133872 | chr4:136106565-136106506 | Kdm1a |
| 2.1058213 | up | NM_026193 | chr3:103625786-103625845 | Ap4b1 |
| 2.7473334 | up | NM_172702 | chr4:129930882-129930823 | Serinc2 |
| 6.6398571 | up | NM_146289 | chr17:37711588-37711529 | Olfr113 |
| 2.052228 | up | NM_178598 | chr1:174437450-174437506 | Tagln2 |
| 2.5504067 | up | NM_053112 | chr14:44500674-44500615 | Ear10 |
| 2.9713948 | up | NM_001286785 | chr6:93628301-93628242 | Magi1 |
| 11.1690742 | up | NM_183126 | chrX:36315735-36315794 | 6030498E09Rik |
| 2.4606482 | up | NM_146815 | chr9:38685510-38685569 | Olfr926 |
| 2.0746486 | up |  | chr13:008452550-008452491 |  |
| 33.2642131 | up | NM_008655 | chr10:80394367-80394426 | Gadd45b |
| 2.2422296 | up | NM_001276361 | chr2:25784787-25784728 | Camsap1 |
| 8.0125808 | up | NM_009731 | chr6:34372905-34372964 | Akr1b7 |
| 12.7042121 | up | AK028741 | chr12:92558311-92558252 | Cep128 |
| 2.046122 | up | NM_001253888 | chr14:56270566-56270625 | Tssk4 |
| 3.792101 | up | NM_009260 | chr11:30007757-30007698 | Sptbn1 |
| 3.5535308 | up | NM_010707 |  | Lgals6 |
| 5.0368264 | up | NM_183187 | chr14:9130333-9130274 | Fam107a |
| 6.0417354 | up | NM_172741 | chr7:35021831-35021772 | 4931406P16Rik |
| 2.2348841 | up | NM_133667 | chr11:94887728-94887669 | Pdk2 |
| 2.3088765 | up | NM_026046 | chr7:13391639-13391580 | Zfp329 |
| 3.8028325 | up | NM_011095 | chr7:3664196-3664137 | Pirb |
| 6.8233915 | up | NM_172253 | chr12:34122794-34122853 | Twistnb |
| 3.5408998 | up | NM_175675 | chr5:30961940-30961999 | Slc35f6 |
| 3.082309 | up | NM_010706 | chr7:29626658-29626717 | Lgals4 |
| 5.6855346 | up | AK020394 | chr5:108792842-108792901 | 9330198I05Rik |
| 28.1759237 | up | BC029726 | chr6:86477847-86477906 | 1600020E01Rik |
| 2.0330518 | up | NM_001113331 | chr3:89233868-89233927 | Shc1 |
| 8.8477094 | up | AK049648 | chr17:63208928-63208869 | C530014P21Rik |
| 2.2565786 | up |  | chr4:057426182-057426123 |  |
| 2.2606741 | up | NM_133772 | chr8:73121515-73121456 | Ssbp4 |
| 4.8917918 | up | NM_030074 | chr3:94810684-94810625 | Zfp687 |
| 4.2109645 | up | NM_029389 | chr14:35081829-35081770 | Fam35a |
| 29.1570044 | up | NM_001039077 | chr8:28160027-28160086 | Prosc |
| 11.3500284 | up | NM_031185 | chr10:5987266-5987207 | Akap12 |
| 3.446586 | up | NM_015742 | chr8:73884459-73884518 | Myo9b |
| 3.1729021 | up | NM_023805 | chr9:107553884-107553825 | Slc38a3 |
| 3.7941323 | up | NM_001253353 | chr4:43045441-43045382 | Fam214b |
| 2.9893058 | up |  | chr5:148994534-148994475 |  |
| 2.1725797 | up | NM_010303 | chr11:109257528-109257587 | Gna13 |
| 5.2641204 | up | NM_011742 | chr8:114194523-114194582 | Zfp1 |
| 65.0348359 | up | NM_001033764 | chr1:26739213-26739154 | 4931408C20Rik |
| 5.8902628 | up | BC029005 | chr12:77496204-77496263 | Zbtb1 |
| 8.0631301 | up | NM_011352 | chr9:57810607-57810666 | Sema7a |
| 11.6083659 | up | NM_015747 | chr2:129037286-129037345 | Slc20a1 |
| 3.1767232 | up | NM_011090 | chr7:3860336-3860277 | Lilra6 |
| 7.8299495 | up | NM_013774 | chr12:106442920-106442979 | Tcl1b4 |
| 3.577627 | up | NM_022305 | chr4:40751798-40751739 | B4galt1 |
| 6.7856922 | up | NM_016678 | chr4:43943868-43943927 | Reck |
| 3.3529652 | up |  | chr7:080457379-080457438 |  |
| 2.1658668 | up | XM_003086873 | chr2:111684694-111684635 | Gm13931 |
| 2.9575448 | up | NM_001038619 | chr1:163920514-163920455 | Dnm3 |
| 4.6729309 | up | AK036921 | chr10:41548868-41548927 | 9930024M15Rik |
| 3.141968 | up | NM_001162917 | chr9:64765272-64765331 | Dennd4a |
| 5.6567131 | up | NR_045506 | chr11:53991350-53991291 | 4933405E24Rik |
| 2.9569802 | up | NM_145584 | chr7:121184084-121184143 | Spon1 |
| 2.1536563 | up | NM_001271585 | chr2:181284236-181284295 | Dnajc5 |
| 2.0934987 | up |  |  |  |
| 2.0677847 | up | NM_008882 | chr1:196642958-196643017 | Plxna2 |
| 5.0009272 | up | AK045050 | chr2:118959276-118959335 | Rad51 |
| 5.5216839 | up | NM_008793 | chr10:79784153-79784094 | Pcsk4 |
| 4.5299559 | up |  | chr10:031294363-031294304 |  |
| 2.0473418 | up | NM_015797 | chr4:147519956-147519897 | Fbxo6 |
| 3.9690804 | up | NR_003964 | chr12:11889108-11889049 | Tubb2a-ps2 |
| 2.5059722 | up | NM_029348 | chr11:69592830-69592889 | Zbtb4 |
| 2.0768528 | up | NM_001110337 | chr11:114733852-114733911 | Gprc5c |
| 12.1058083 | up | AK167004 | chr12:24554268-24554327 |  |
| 4.2319336 | up | NM_194346 | chr14:56222239-56222405 | Rnf31 |
| 10.4645691 | up | NM_053087 | chr5:91464119-91464178 | Epgn |
| 4.7695009 | up | NR_003649 | chr12:36818258-36818317 | Gm5434 |
| 16.1936837 | up | NM_021521 | chrX:98494213-98494272 | Med12 |
| 2.1551809 | up | NM_019747 | chr5:138581000-138580941 | Zfp113 |
| 2.4081619 | up | NM_024479 | chr5:135418429-135418488 | Wbscr27 |
| 6.413809 | up | AK019493 | chr19:34955719-34955778 |  |
| 3.3702743 | up |  | chr8:073995628-073995569 |  |
| 2.1368345 | up | AK078348 | chr15:73254479-73254538 | LOC497255 |
| 2.7702973 | up | NM_175272 | chr7:56664216-56664275 | Nav2 |
| 13.8215202 | up | NM_019964 | chr6:88173124-88173183 | Dnajb8 |
| 4.5958191 | up | NM_133867 | chr3:107695731-107695790 | Eps8l3 |
| 5.9107164 | up | NM_011428 | chr2:136607709-136607768 | Snap25 |
| 2.9642693 | up | AB546195 | chr16:91665798-91665857 | Son |
| 2.4663593 | up | NM_145076 | chr6:37916102-37916161 | Trim24 |
| 11.0215949 | up | NM_001168294 | chr12:105458989-105459048 | Serpina3f |
| 2.1382096 | up |  | chr3:078749990-078749931 |  |
| 3.5303837 | up | NM_153388 | chr19:4611852-4611793 | Lrfn4 |
| 5.7362544 | up |  | chr15:059584175-059584116 |  |
| 2.1593753 | up | NM_028275 | chr14:23403747-23415685 | 1700112E06Rik |
| 3.2184224 | up |  | chr14:054767961-054767902 |  |
| 4.365323 | up | NM_173410 | chr7:139177184-139177243 | Gpr26 |
| 4.5253291 | up | AK082631 | chr11:76153830-76153771 | C230071I02Rik |
| 6.2084796 | up | AK028075 | chr10:17938171-17938112 | Ccdc28a |
| 2.6512579 | up |  | chr2:082347866-082347925 |  |
| 47.7662197 | up | NM_178712 | chrX:156934689-156934748 | Gpr64 |
| 4.3346665 | up |  | chr2:050377410-050377351 |  |
| 12.9806885 | up | AK037728 | chr2:152649115-152649056 | BB166591 |
| 5.2975302 | up | NM_139220 | chr8:19497669-19497728 | Defb7 |
| 3.2453667 | up | NM_001159417 | chr14:56228806-56228865 | Irf9 |
| 2.5570027 | up | XM_003946188 | chr6:72825219-72825160 | LOC101056333 |
| 3.2169968 | up | NM_001010836 | chr7:19963802-19963861 | Ppp1r13l |
| 5.3702554 | up | AK132788 | chr6:86670276-86670217 | Gmcl1 |
| 6.1564919 | up | AK082949 | chr14:32291565-32291506 | Mettl6 |
| 4.2520071 | up |  |  |  |
| 3.5754851 | up | NM_145939 | chr16:20606610-20606032 | Alg3 |
| 2.8656265 | up | NM_009948 | chr15:89247165-89247106 | Cpt1b |
| 3.0053854 | up | AK137366 | chr9:106783901-106783842 |  |
| 17.910711 | up | NM_181857 | chr5:34349906-34349847 | Poln |
| 2.7931063 | up | NM_145505 | chr19:57464017-57464076 | Fam160b1 |
| 2.0283344 | up | NM_013691 | chr3:89030682-89030741 | Thbs3 |
| 5.3838753 | up | AF528078 | chr11:3233674-3233733 | Pik3ip1 |
| 3.4061713 | up | NM_001284190 | chr4:110252720-110252779 | Agbl4 |
| 3.0222353 | up | AK047130 | chr14:120740823-120740882 | B930025B16Rik |
| 2.0894082 | up | NM_001114333 | chr10:10408712-10408653 | Grm1 |
| 3.122897 | up | NR_033603 | chr10:110938208-110938267 | Gm5176 |
| 3.7163235 | up | NM_145857 | chr8:91212245-91212304 | Nod2 |
| 2.1737228 | up | NM_025822 | chr3:66798460-66798519 | Rsrc1 |
| 2.072127 | up | XR_168752 | chr16:93605154-93605213 | Gm9510 |
| 3.8178182 | up | NM_147051 | chr2:36960291-36960232 | Olfr362 |
| 2.4554669 | up | NM_133919 | chr5:104276766-104276825 | Aff1 |
| 4.7157443 | up | NR_045743 | chr15:102953565-102953506 | D930007P13Rik |
| 6.0857012 | up | NM_001081037 | chr10:121218110-121218051 | Srgap1 |
| 2.6957076 | up | NM_008449 | chr2:49630228-49630287 | Kif5c |
| 3.3168758 | up |  | chr7:010341269-010341210 |  |
| 2.4474054 | up | NM_194345 | chr14:70984934-70984875 | Fam160b2 |
| 4.4195749 | up | NM_016692 | chr19:9946937-9946878 | Incenp |
| 2.3784203 | up | NM_010811 | chr14:21543317-21543258 | Ndst2 |
| 2.4863774 | up | AK052079 | chr15:80663562-80663621 | D230044B12Rik |
| 2.9232054 | up | NM_001167872 | chr7:30809941-30810000 | Zfp568 |
| 9.4069041 | up | NM_177343 | chr2:5214625-5214566 | Camk1d |
| 3.8645719 | up | AK044744 | chr2:156347886-156347945 | Epb4.1l1 |
| 2.9292656 | up | NM_153287 | chr9:119880516-119880457 | Csrnp1 |
| 2.2455331 | up | XR_105390 | chr10:119819551-119819610 |  |
| 3.1524118 | up | NM_007859 | chr4:153339151-153339092 | Dffb |
| 6.658848 | up | AK013575 | chr5:65787196-65787255 | Lias |
| 2.3649849 | up | NM_001081259 | chr1:192829890-192829831 | Mfsd7b |
| 2.9416147 | up | NM_029397 | chr2:155920704-155920645 | Rbm12 |
| 4.988442 | up | NM_177092 | chr10:120219111-120219052 | Msrb3 |
| 2.7229038 | up | NM_024478 | chr5:36815817-36815876 | Grpel1 |
| 2.1786418 | up |  | chrX:019817443-019817502 |  |
| 3.4218938 | up | NM_010807 | chr4:129192947-129193006 | Marcksl1 |
| 3.2962293 | up | NM_172730 | chr6:91849772-91849831 | Ccdc174 |
| 3.9963765 | up | NM_011181 | chr7:53063154-53063095 | Cyth2 |
| 2.219438 | up | AF433163 | chr6:70557780-70557839 |  |
| 6.9742322 | up | NM_018733 | chr2:66173557-66173498 | Scn1a |
| 2.2410246 | up | NM_028326 | chr4:62794820-62794879 | Zfp618 |
| 4.0757021 | up | NM_001114663 | chr1:55753465-55753524 | Plcl1 |
| 6.6416519 | up | NM_001100614 | chr11:99676526-99676467 | Gm11564 |
| 8.3825972 | up |  | chr7:058410672-058410613 |  |
| 2.3053711 | up | NM_011669 | chr5:147546498-147546439 | Usp12 |
| 3.447838 | up | NM_173863 | chr7:87733271-87733212 | Crtc3 |
| 2.0314877 | up | NM_033073 | chr15:101253718-101253777 | Krt7 |
| 81.086453 | up | NM_016659 | chr6:130314129-130314070 | Klra1 |
| 3.3778868 | up | NM_010413 | chrX:7507308-7507249 | Hdac6 |
| 4.0859753 | up | NM_145839 | chr5:99646507-99646448 | Rasgef1b |
| 3.3976753 | up | NM_001163728 | chr11:72029221-72029280 | 4930563E22Rik |
| 2.3982815 | up | AK084992 | chr14:76831890-76831949 | D430022A14Rik |
| 5.0237929 | up |  | chr8:010927359-010927300 |  |
| 2.0463432 | up | NM_001033139 | chr19:7431033-7430974 | AI846148 |
| 8.7082571 | up | AK014609 | chr3:121490477-121490418 | 4633401B06Rik |
| 2.4370626 | up | NM_026480 | chr9:78224863-78224804 | Ooep |
| 2.676458 | up | AK009062 | chr5:110436368-110436427 | Zfp932 |
| 2.3420242 | up | NM_010258 | chr18:11085039-11085097 | Gata6 |
| 3.0602807 | up | AK018447 | chr8:112069242-112069183 | 8430428J23Rik |
| 2.1137577 | up | NM_012020 | chr9:98856894-98856950 | Foxl2 |
| 24.0461247 | up | NR_030676 | chr3:103768517-103768459 | Phtf1os |
| 3.895901 | up | AK157986 | chr7:82748128-82748187 | Akap13 |
| 2.0169789 | up | NM_016740 | chr3:93330022-93330081 | S100a11 |
| 2.2248892 | up | NM_022995 | chr2:173050025-173049966 | Pmepa1 |
| 2.0412261 | up | NM_181444 | chr6:135034590-135034649 | Gprc5a |
| 2.8891798 | up | NM_001205353 | chr15:85967864-85967923 | Gramd4 |
| 7.0567911 | up | NM_007893 | chr17:24580940-24580881 | E4f1 |
| 4.8318645 | up | AK041735 | chr11:119322624-119322565 | A930037H05Rik |
| 5.0168403 | up | NM_001113333 | chr2:92251476-92251417 | Cry2 |
| 2.5637543 | up | NM_144940 | chr6:90314337-90314396 | Uroc1 |
| 2.0389641 | up | AK051328 | chr15:76926237-76926178 | 1700109K24Rik |
| 6.5763145 | up | NM_009072 | chr12:16984727-16984785 | Rock2 |
| 3.6059975 | up | CA491940 |  | 0610008F07Rik |
| 2.3109996 | up | NM_177638 | chr17:57205264-57205323 | Crb3 |
| 6.3849973 | up | NM_010155 | chr7:26028406-26028347 | Erf |
| 2.2588092 | up | NM_001081100 | chr4:154519401-154519460 | Morn1 |
| 15.3264079 | up | NR_028111 | chr1:162002065-162002124 | 4930523C07Rik |
| 3.1544402 | up | NM_009794 | chr1:184397454-184397395 | Capn2 |
| 3.6253493 | up | NM_172400 | chr4:132108959-132109018 | Dnajc8 |
| 2.4024177 | up | NM_178933 | chr9:65260764-65260705 | Slc51b |
| 3.730259 | up | AK170993 | chr8:73053083-73053142 | Fkbp8 |
| 4.8167265 | up | AK144920 | chr6:98956738-98956679 | Foxp1 |
| 2.8449904 | up | NM_010118 | chr10:67004798-67004857 | Egr2 |
| 2.746153 | up | NM_001037940 | chr5:30112861-30112920 | Dnajb6 |
| 3.5140775 | up | NM_025404 | chr11:101528989-101529048 | Arl4d |
| 2.2310402 | up | AK156907 | chr5:92776675-92776616 | Cxcl10 |
| 2.6379002 | up | NM_009396 | chr12:112692959-112693018 | Tnfaip2 |
| 2.1700684 | up | NM_172403 | chr11:58693668-58693727 | 2810021J22Rik |
| 2.3544988 | up | NM_001110276 | chr16:91832536-91832595 | Itsn1 |
| 17.2726915 | up | NM_001039934 | chr1:66488543-66488602 | Map2 |
| 10.3901725 | up | NM_145634 | chr11:114977592-114977533 | Cd300lf |
| 16.8959504 | up | NM_199364 | chr8:71659638-71659579 | Lzts1 |
| 3.7257452 | up | NM_198294 | chr2:59684116-59684175 | Tanc1 |
| 2.9083427 | up | NM_172713 | chr5:92713192-92713133 | Sdad1 |
| 4.5767018 | up | AK146852 | chr11:77593610-77593669 | Myo18a |
| 2.0531628 | up | NM_026574 | chr2:119232623-119232564 | Ino80 |
| 4.7092728 | up | BG295362 |  | D9Ertd788e |
| 3.1030222 | up | NM_001085373 | chr18:44584886-44584827 | Mcc |
| 11.7019058 | up | NM_029372 | chr19:58627516-58627575 | Ccdc172 |
| 2.4866406 | up | NM_021274 | chr5:92775840-92775781 | Cxcl10 |
| 9.6089314 | up | NM_013602 | chr8:96704165-96704224 | Mt1 |
| 2.9303256 | up | BC094664 | chr11:95930376-95930317 | Atp5g1 |
| 3.4347464 | up | NM_028125 | chr2:181146463-181146404 | Zbtb46 |
| 8.2955923 | up | NM_010139 | chr4:140885139-140885198 | Epha2 |
| 3.3942057 | up | AK153738 | chr4:141643535-141643594 |  |
| 2.0907385 | up |  |  |  |
| 7.7291277 | up | AK153771 | chr13:29040885-29040826 | Sox4 |
| 11.0849588 | up | NM_027418 | chr9:75236652-75236593 | Mapk6 |
| 5.5455344 | up | AK144547 | chr6:84783404-84783345 | Exoc6b |
| 10.3185869 | up | NM_001037709 | chr4:43439862-43439921 | Rusc2 |
| 2.9720739 | up | NM_177382 | chr7:121693828-121693769 | Cyp2r1 |
| 7.5229213 | up | NM_001163810 | chr7:25118174-25118115 | Tescl |
| 5.0378026 | up | NM_008109 | chr2:155768109-155768050 | Gdf5 |
| 2.3095066 | up | NM_177052 | chr17:49894503-49897747 | Kif6 |
| 7.7654095 | up | NM_001033260 | chr10:62122321-62122262 | Stox1 |
| 2.0302572 | up |  | chr14:041653541-041653482 |  |
| 3.7416111 | up | NM_001110203 | chr12:71364786-71364727 | Trim9 |
| 4.9341138 | up | NM_133904 | chr5:114700702-114700761 | Acacb |
| 2.250852 | up | NM_153508 | chr6:124380847-124380788 | Clstn3 |
| 5.9063686 | up | NM_018832 | chrX:7251951-7251892 | Magix |
| 2.5152059 | up | BC010546 | chr1:175677611-175677552 | Ifi204 |
| 2.3583944 | up | AK020830 | chr7:139951010-139950951 | Fam53b |
| 4.8838368 | up | NM_183321 | chr7:31324214-31324155 | BC053749 |
| 2.5501456 | up | NR_015604 | chr6:125189888-125189947 | E130112N10Rik |
| 2.1533517 | up | NM_026323 | chr2:164393947-164394006 | Wfdc2 |
| 3.4450311 | up | NM_001145972 | chr8:97467119-97467178 | Gpr114 |
| 3.2866401 | up | NM_001113530 | chr3:107548609-107548550 | Csf1 |
| 3.5620851 | up | NM_018747 | chr10:25003825-25003766 | Akap7 |
| 2.1254252 | up | XR_141835 | chr17:42548730-42548789 | Gm9753 |
| 4.0827267 | up | AK157835 | chr13:49356984-49356925 | Fgd3 |
| 2.6893117 | up | NM_001162919 | chr13:21513977-21513918 | Pgbd1 |
| 28.7055243 | up | NM_178373 | chr6:113374689-113374630 | Cidec |
| 15.1832634 | up | AK051536 | chr17:25407250-25407191 | Ube2i |
| 5.4041773 | up | NM_001256019 | chr4:122989965-122989906 | Bmp8a |
| 2.6294103 | up | NM_009769 | chr14:99702656-99712388 | Klf5 |
| 3.1726991 | up | AK012244 | chr12:100598140-100598081 | 3300002A11Rik |
| 7.4083764 | up | NM_001081037 | chr10:121222357-121222298 | Srgap1 |
| 22.2071385 | up | NM_146787 | chr9:38564040-38564099 | Olfr920 |
| 2.2102528 | up | CB850364 |  | BE949265 |
| 13.0851785 | up |  | chr4:116318368-116318309 |  |
| 2.0274861 | up | NM_011548 | chr10:79872698-79872639 | Tcf3 |
| 3.186821 | up | NM_001277867 | chr5:124701750-124701691 | Mphosph9 |
| 17.1099104 | up | NM_011094 | chr7:3789780-3789721 | Pira7 |
| 6.6319646 | up | NM_013589 | chr12:86124226-86124167 | Ltbp2 |
| 2.1702365 | up | AK143961 | chr11:110306327-110306268 | Gm10836 |
| 8.7275553 | up | NM_178710 | chr9:50700976-50700917 | Sik2 |
| 12.7372551 | up | AK035822 | chr2:52778282-52778341 | D2Ertd295e |
| 3.7784347 | up | NM_134041 | chr12:114403580-114403639 | 4930427A07Rik |
| 7.9325653 | up | NM_001034902 | chr6:85068626-85068567 | Gm5878 |
| 5.6916778 | up | AK136342 | chr4:137574239-137574298 | 8030494B02Rik |
| 2.3733915 | up |  | chr8:036115242-036115183 |  |
| 4.7295896 | up | NM_021455 | chr5:135614195-135614252 | Mlxipl |
| 3.2735613 | up | XM_003945714 | chr4:42104766-42104825 |  |
| 3.1337433 | up | NM_146119 | chr2:32780704-32780763 | Fam129b |
| 10.6170025 | up | AK140493 | chr7:148649023-148648964 | Efcab4a |
| 4.4803766 | up | NM_001166584 | chr7:120042204-120042263 | Tead1 |
| 2.8741282 | up | NM_198005 | chr2:70926599-70926658 | Dcaf17 |
| 3.8701702 | up | NM_026515 | chr9:65749748-65749807 | 2810417H13Rik |
| 8.1436243 | up | NM_001195767 | chr12:88104669-88104728 | Gm6772 |
| 2.6179971 | up | AK051413 | chr19:29879815-29879756 | 9930021J03Rik |
| 5.070023 | up |  | chrY_random:017330790-017330731 | |
| 6.0603831 | up | NM_007706 | chr10:94875527-94875468 | Socs2 |
| 3.1997341 | up | NM_001039551 | chr1:36583700-36583759 | Cnnm3 |
| 4.1818992 | up | NM_011905 | chr3:83640403-83640344 | Tlr2 |
| 9.822326 | up | AK156446 | chr12:56223629-56223688 |  |
| 25.3852647 | up | NM_001285952 | chr4:104689395-104689454 | 1700024P16Rik |
| 51.0145898 | up | NM_001172472 | chr11:116397591-116397650 | Sphk1 |
| 2.934534 | up |  | chrX:099975046-099975105 |  |
| 2.073372 | up | NM_146534 | chr13:21234085-21234026 | Olfr1368 |
| 2.1285308 | up | AK052167 | chr19:41670568-41670627 | AI606181 |
| 3.5671528 | up | NM_028347 | chr9:56991347-56991288 | Neil1 |
| 3.7145386 | up | NM_001159724 | chr7:20309924-20309866 | Pvrl2 |
| 28.1244816 | up | NM_020576 | chr17:35671531-35671590 | Psors1c2 |
| 4.1356669 | up | XM_003945440 | chr8:48770752-48770811 | AA386476 |
| 2.2067497 | up | AK013276 | chr13:49333551-49333492 | Susd3 |
| 2.922265 | up | NM_001162485 | chr2:24780934-24780875 | Arrdc1 |
| 3.3528989 | up | NM_001034856 | chrX:35391445-35391386 | 4930430D24Rik |
| 3.3194334 | up | NM_001005510 | chr12:77022219-77023953 | Syne2 |
| 5.1234762 | up | NM_178404 | chr2:128816832-128816891 | Zc3h6 |
| 8.6252585 | up | NM_207018 | chr6:132843899-132843958 | Tas2r113 |
| 2.2222252 | up |  | chr11:049057523-049057582 |  |
| 2.1704317 | up | NM_144927 | chr8:32270484-32270543 | Tti2 |
| 53.8038685 | up | NM_013932 | chr9:35349594-35349535 | Ddx25 |
| 2.4480449 | up | NM_001271585 | chr2:181283486-181283545 | Dnajc5 |
| 2.452348 | up | AK085785 | chr11:89996670-89996611 |  |
| 9.7214667 | up | NM_001146007 | chr7:111488610-111488551 | Trim12c |
| 2.6671806 | up | NM_008791 | chr16:96747241-96747300 | Pcp4 |
| 9.7287139 | up | NM_026114 | chr12:79987922-79987981 | Eif2s1 |
| 2.6804085 | up | NM_007920 | chr14:79973368-79973425 | Elf1 |
| 4.8740904 | up | AK008077 | chr4:40217805-40217864 |  |
| 6.1912701 | up | NM_001281514 | chr5:14974556-14974497 | Gm10354 |
| 16.7206265 | up |  | chr10:61812630-61812689 |  |
| 4.9624532 | up | AK014981 | chr17:56773945-56774004 | Catsperd |
| 7.6448617 | up | NM_015811 | chr1:146092021-146091962 | Rgs1 |
| 5.2576183 | up | NM_201372 | chr9:44221035-44220543 | Ccdc84 |
| 9.1271327 | up | NR_001460 | chr4:43505906-43505847 | Rmrp |
| 5.1576933 | up | NM_026864 | chr5:147658864-147658923 | Rasl11a |
| 2.4521496 | up | NM_010062 | chr8:87435251-87435310 | Dnase2a |
| 2.623655 | up | AK086235 | chr11:121208399-121208341 | Wdr45b |
| 2.9304252 | up | NM_183099 | chr15:76202458-76202399 | Tssk5 |
| 2.4999768 | up | AK087451 | chr1:138392182-138392241 | Kif14 |
| 2.793912 | up | NM_025974 | chr9:120483689-120483748 | Rpl14 |
| 11.0630786 | up | NM_029098 | chr15:98734411-98734352 | Lmbr1l |
| 6.7367464 | up | NM_001243748 | chr9:107464532-107464591 | Rassf1 |
| 2.2314199 | up | NM_021402 | chr4:155331683-155331742 | Ube2j2 |
| 2.3690686 | up | NM_021552 | chr13:97905309-97905250 | Nsa2 |
| 7.7544314 | up | AK080089 | chr9:55088579-55088520 | Nrg4 |
| 7.3693603 | up | NM_008039 | chr17:18030540-18030599 | Fpr2 |
| 4.1352054 | up | NM_026578 | chr3:129527914-129527855 | Gar1 |
| 2.0271291 | up | AK080460 | chr7:80882874-80882815 | A830073O21Rik |
| 10.5016637 | up | NR_003967 | chr7:113567980-113567921 | Gm4759 |
| 22.6916718 | up | U18577 | chr6:70622312-70622371 |  |
| 2.2571574 | up | NM_026890 | chr14:55642880-55642939 | Ngdn |
| 9.6920409 | up | NM_027219 | chr15:78681251-78681310 | Cdc42ep1 |
| 4.3471895 | up | NR_004415 | chr11:87285025-87285084 | Rnu3b1 |
| 4.7906121 | up | XR_168593 | chrX:107931978-107932037 | Gm4354 |
| 5.0642267 | up | NM_175472 | chr4:108151673-108151732 | Zcchc11 |
| 2.1255792 | up | NM_001081241 | chr8:108146035-108146094 | Fam65a |
| 2.5281043 | up |  | chr7:068073862-068073803 |  |
| 9.8246451 | up | NM_001029842 | chr11:109312429-109312371 | Slc16a6 |
| 5.8824535 | up | NM_001253804 | chr8:108467552-108467493 | Slc12a4 |
| 3.0743789 | up | NM_009293 |  | Sts |
| 4.6009124 | up | NM_001083927 | chr9:61266180-61266239 | Tle3 |
| 8.8653605 | up | NM_001033528 | chr11:118126215-118126156 | Usp36 |
| 2.1740061 | up | NM_130860 | chr2:32561526-32561467 | Cdk9 |
| 2.0536651 | up | NM_138315 | chr10:41206772-41206831 | Mical1 |
| 2.0525552 | up | NM_144559 | chr1:172959738-172959797 | Fcgr4 |
| 2.3084348 | up | BC006653 | chr6:140402520-140402579 |  |
| 5.3549979 | up | NM_027389 | chr3:88639218-88640969 | Gon4l |
| 3.1147509 | up | BE853708 | chr3:32512374-32512433 |  |
| 2.0537485 | up | NM_029245 | chr6:83718202-83718261 | Ankrd53 |
| 5.3806818 | up | NM_010329 | chr4:142857398-142857339 | Pdpn |
| 2.20406 | up | NM_021397 | chr7:31374761-31374702 | Zbtb32 |
| 3.3569941 | up | NM_031880 | chr11:69664773-69664714 | Tnk1 |
| 2.9052204 | up | NM_144926 | chr7:134113725-134113784 | Sez6l2 |
| 2.0009568 | up | NM_018744 | chr18:47407607-47407548 | Sema6a |
| 2.6550868 | up | NM_021462 | chr10:80128218-80128159 | Mknk2 |
| 5.4522284 | up | NM_001005425 | chr2:165177033-165176974 | Zfp663 |
| 2.770989 | up | XR_104791 | chr3:94820933-94820992 |  |
| 59.9197417 | up | NM_030560 | chr2:77734351-77734292 | Cwc22 |
| 3.8315178 | up | NM_010501 | chr19:34663105-34663164 | Ifit3 |
| 4.6191423 | up | AK144717 | chr19:4062498-4062439 |  |
| 5.4122519 | up | NM_001082960 | chr7:135261755-135261814 | Itgam |
| 2.5020697 | up | NM_028596 | chr5:110791902-110791843 | Fbrsl1 |
| 3.5847264 | up | AK021252 | chr15:83228272-83228213 | C430045I18Rik |
| 3.7916965 | up | NM_001204983 | chr10:53021388-53021329 | Cep85l |
| 14.4159749 | up | NM_009946 | chr13:54481348-54481407 | Cplx2 |
| 4.213568 | up | AK078161 | chr9:100448797-100448856 | Gm7111 |
| 2.5685892 | up | NM_001032298 | chr3:88181881-88181822 | Bglap2 |
| 3.4051645 | up | NM_146017 | chr11:33450978-33450919 | Gabrp |
| 2.0256672 | up | NM_031863 | chr17:41068659-41068600 | Cenpq |
| 2.1827698 | up | NM_017462 | chr7:86595438-86595379 | Polg |
| 3.0998341 | up | NM_153533 | chr15:101946406-101946465 | Tenc1 |
| 3.1375194 | up | NM_023190 | chr14:55303878-55298161 | Acin1 |
| 3.5573847 | up | XR_035714 | chr19:55923148-55923207 | Ppnr |
| 2.1304438 | up | NM_010145 | chr1:182924183-182924124 | Ephx1 |
| 2.6523832 | up | NM_001242372 | chr3:88698374-88698433 | Gon4l |
| 3.052876 | up | NM_029338 | chr17:46266338-46266279 | Rsph9 |
| 2.0306009 | up | NM_015748 | chr19:41674808-41674749 | Slit1 |
| 3.2435786 | up |  | chr11:017366191-017366250 |  |
| 2.0866481 | up | NM_026815 | chr7:31388170-31388111 | Upk1a |
| 3.6865686 | up | NM_176847 | chr11:115176566-115176507 | Ush1g |
| 6.1867811 | up | NM_019952 | chr19:4222480-4222539 | Clcf1 |
| 4.03458 | up | NM_001166552 | chr1:135552546-135552487 | Zbed6 |
| 5.1716405 | up | AK007108 | chr1:34636314-34636255 |  |
| 25.5371496 | up |  | chr6:140289821-140289762 |  |
| 3.0428263 | up | NM_010215 | chr7:52096115-52096174 | Il4i1 |
| 2.8305845 | up | NM_172715 | chr5:101328054-101328113 | Agpat9 |
| 15.9185277 | up | NM_146630 | chr17:37933191-37933250 | Olfr123 |
| 2.0547996 | up | NM_011037 | chr19:44909895-44909954 | Pax2 |
| 3.7542832 | up | NM_178922 | chr16:17263319-17263378 | Hic2 |
| 2.2975738 | up | NM_023456 | chr6:49773743-49777505 | Npy |
| 6.5921794 | up | NM_013568 | chr6:126658483-126658424 | Kcna6 |
| 21.6371188 | up | NM_010755 | chr15:79189442-79189501 | Maff |
| 2.3359494 | up | NM_001081178 | chr17:43596447-43596506 | Gpr116 |
| 2.2826273 | up | NM_176976 | chr9:15127032-15126973 | 5830418K08Rik |
| 7.3041632 | up | NM_145475 | chr15:85969648-85969589 | Cerk |
| 2.1799469 | up | NM_026198 | chr3:108359451-108359392 | Tmem167b |
| 2.631824 | up | NM_007764 | chr16:17486238-17486297 | Crkl |
| 2.6826112 | up | NM_001286518 | chr19:9151349-9151408 | Ahnak |
| 4.1222786 | up | AK046997 | chr9:45670928-45670869 | Rnf214 |
| 8.9770411 | up | NM_007609 | chr9:5336658-5336717 | Casp4 |
| 2.5843259 | up | NM_175314 | chr6:92723110-92723051 | Adamts9 |
| 2.4005497 | up | NM_021877 | chr14:70973263-70973322 | Hr |
| 2.9931384 | up | NM_001033311 | chr5:117804886-117804945 | Vsig10 |
| 2.3427943 | up | AK133032 | chr5:122475403-122475462 | Gm3970 |
| 23.0183928 | up | BC019386 | chr19:55390705-55390764 | Vti1a |
| 16.3346043 | up | NR_040409 | chr3:96359946-96359887 | Gm15441 |
| 3.2277873 | up | NM_021897 | chr4:11101174-11101233 | Trp53inp1 |
| 7.7968479 | up | AK087890 | chr10:89206423-89206364 | E330037I15Rik |
| 3.458264 | up | NM_177244 | chr2:69524952-69524893 | Fastkd1 |
| 4.1376577 | up | NM_001100184 | chr7:27943506-27943565 | Cyp2t4 |
| 4.7657012 | up |  | chr5:023515561-023515502 |  |
| 253.9142231 | up | NM_173400 | chr4:86247191-86247132 | Haus6 |
| 6.1034417 | up | AK046501 | chr14:61818444-61818503 | Sacs |
| 2.4741586 | up | NM_010091 | chr4:155233151-155233210 | Dvl1 |
| 3.8543191 | up | NM_001114125 | chr2:35582971-35583030 | Dab2ip |
| 3.3137221 | up | NM_153074 | chr8:73144658-73144717 | Lrrc25 |
| 2.2644599 | up | NM_017377 | chr4:117546012-117545953 | B4galt2 |
| 2.037656 | up | NM_011909 | chr6:121220874-121220933 | Usp18 |
| 2.548646 | up | NM_025482 | chr2:181250024-181250083 | Tpd52l2 |
| 2.7943699 | up | NM_013872 | chr15:81786866-81786807 | Pmm1 |
| 2.7883603 | up | NM_009700 | chr18:15551423-15551364 | Aqp4 |
| 6.2100677 | up | NM_022657 | chr6:127030758-127030817 | Fgf23 |
| 2.0910405 | up | NM_011752 | chr9:46089549-46089608 | Zfp259 |
| 2.0128658 | up | NM_025745 | chr11:30829848-30829789 | Erlec1 |
| 2.2620763 | up | NM_021899 | chr6:122789504-122789563 | Foxj2 |
| 2.0160905 | up | NM_008401 | chr7:135212140-135212199 | Itgam |
| 2.467157 | up | NR_001461 | chr7:150434079-150434020 | Kcnq1ot1 |
| 5.0765768 | up | NM_007513 | chr5:149140016-149139957 | Slc7a1 |
| 2.3633741 | up | XR_140812 | chr7:46529329-46529270 | Gm20749 |
| 2.2734811 | up | NM_028667 | chr3:41562363-41562422 | D3Ertd751e |
| 4.7969686 | up | NM_207677 | chr7:25987918-25987859 | Dedd2 |
| 2.7762298 | up |  |  |  |
| 2.4687504 | up | NM_144850 | chr15:97575483-97575424 | Rapgef3 |
| 2.5228353 | up | NM_001163300 | chr17:56745602-56745661 | Safb |
| 2.0502114 | up | AK078562 | chr4:97560763-97560822 | D4Ertd681e |
| 10.9141479 | up | AK036896 | chr2:135487286-135487345 | Plcb4 |
| 2.3220693 | up | NM_029211 | chr7:109168724-109168665 | Rnf121 |
| 2.3051458 | up | NM_028170 | chr8:74984369-74984428 | 1700030K09Rik |
| 2.2812855 | up | NM_023502 | chr3:51059675-51059616 | Elf2 |
| 15.5752131 | up | NM_001159593 | chr2:129029990-129030049 | Slc20a1 |
| 24.4012187 | up | NM_172641 | chr19:56561121-56561180 | Plekhs1 |
| 2.0408859 | up |  | chr4:145718257-145718198 |  |
| 4.2381757 | up | NM_027892 | chr10:107714572-107714631 | Ppp1r12a |
| 2.2552791 | up | NM_011986 | chr4:126421054-126420995 | Ncdn |
| 2.6943342 | up |  | chr9:061321653-061321594 |  |
| 6.1858721 | up | NM_133765 | chr8:124073553-124073494 | Fbxo31 |
| 2.2852738 | up |  | chr2:160995928-160995987 |  |
| 4.1932426 | up | NM_001033275 | chr15:93075066-93075007 | Gxylt1 |
| 4.7685522 | up | NM_001122992 | chr4:131777679-131777620 | Gmeb1 |
| 4.6896719 | up | AK032738 | chr3:148593879-148593820 | 6720422M22Rik |
| 3.2123689 | up | NM_008827 | chr12:86510337-86508335 | Pgf |
| 2.0035554 | up | NM_010689 | chr7:133507778-133507719 | Lat |
| 11.2245803 | up |  | chr7:091715197-091715256 |  |
| 3.3671757 | up | NM_001001735 | chr8:26788359-26788418 | Whsc1l1 |
| 2.6197358 | up | NM_175402 | chr9:106787229-106787170 | Rbm15b |
| 3.3486463 | up | NM_001252587 | chr2:165677592-165677533 | Zmynd8 |
| 2.9063835 | up | NM_023844 | chr16:84816558-84816617 | Jam2 |
| 3.7641914 | up | AK051800 | chr5:149860284-149860226 | Hmgb1 |
| 2.4444563 | up | NM_019765 | chr5:124029228-124029169 | Clip1 |
| 21.4912427 | up | NM_007608 | chr8:124440289-124440230 | Car5a |
| 3.706037 | up | NM_019737 | chr18:20843285-20843226 | B4galt6 |
| 7.6829784 | up | NM_013763 | chr5:135633455-135633514 | Tbl2 |
| 4.9253594 | up | CB193388 | chr8:118225388-118225329 |  |
| 2.3537897 | up | AK019875 | chr9:72890116-72890175 | 5031420N21Rik |
| 3.4569303 | up | NM_177993 | chr12:32614386-32614327 | Hbp1 |
| 2.0249332 | up | NR_033560 | chr9:51648322-51648263 | Arhgap20os |
| 2.1419384 | up | NM_001282055 | chr13:55780414-55780473 | Ddx46 |
| 2.4030955 | up | NM_025699 | chr2:163231624-163231565 | Oser1 |
| 9.0640109 | up | AK142114 | chr16:52417305-52417246 | 6720473M08Rik |
| 3.9540535 | up |  | chr2:126730684-126730625 |  |
| 6.9264734 | up | NM_177732 | chr4:102844382-102844323 | Slc35d1 |
| 3.0103894 | up | NM_009928 | chr4:47283786-47283845 | Col15a1 |
| 2.4404277 | up | NM_013640 | chr8:108459736-108459677 | Psmb10 |
| 2.2580071 | up | NM_027162 | chr11:115469328-115469269 | Mif4gd |
| 26.5875254 | up | NM_001085530 | chr4:41842107-41842166 | Gm13298 |
| 4.1712188 | up | BC016099 | chr7:51507630-51507689 | 2410002F23Rik |
| 2.0362532 | up | NM_173737 | chr6:87886546-87886605 | Hmces |
| 12.9182129 | up | NM_027699 | chr12:36944770-36940957 | Lrrc72 |
| 2.0873883 | up | NM_001278256 | chr2:131744902-131744961 | Prnp |
| 2.9349794 | up | NM_011119 | chr10:127994853-127994822 | Pa2g4 |
| 2.4562161 | up | NM_024215 | chr4:133799291-133799232 | Zfp593 |
| 2.0864611 | up | NM_001163006 | chr4:138658156-138658097 | Minos1 |
| 2.1170366 | up | NM_023438 | chr11:82259736-82259795 | Tmem132e |
| 2.3556418 | up | NM_009123 | chr7:139789115-139789056 | Nkx1-2 |
| 2.5780857 | up |  |  |  |
| 6.7555677 | up | NR_033736 | chr1:155252016-155251957 | E330020D12Rik |
| 6.9034475 | up | NM_001164075 | chr17:71193688-71193629 | Tgif1 |
| 13.2526907 | up | NM_146013 | chr11:3947935-3947994 | Sec14l4 |
| 9.7614671 | up | XM_003945356 | chr2:145502134-145502193 | LOC101055765 |
| 2.0579515 | up | NM_001284248 | chr11:102057135-102057076 | Hdac5 |
| 3.5178598 | up | NM_026110 | chr16:91017176-91017117 | Paxbp1 |
| 19.7259448 | up | NM_001007569 | chr7:17546747-17546806 | Pnmal1 |
| 2.7147095 | up | NM_008466 | chr14:61993203-61993144 | Kpna3 |
| 2.4836106 | up |  | chr15:051708036-051708095 |  |
| 2.1699939 | up | NM_001164581 | chr8:74492659-74492718 | Zfp961 |
| 7.6603112 | up | NM_019942 | chrX:34452471-34452412 | Sept6 |
| 10.1012078 | up | AK138853 | chr5:66335518-66335577 | Chrna9 |
| 2.4820308 | up | NM_001252466 | chr17:56851111-56851170 | Ranbp3 |
| 5.8114872 | up | NM_009681 | chr18:46950403-46950462 | Ap3s1 |
| 2.8855273 | up | NM_178397 | chr13:54762893-54762952 | Faf2 |
| 27.2832972 | up | AV452492 | chr14:106252349-106252290 |  |
| 9.9586598 | up |  | chr3:127501256-127501315 |  |
| 10.8720569 | up |  | chr1:014720902-014720961 |  |
| 5.9222648 | up | NM_001145859 | chr5:34906205-34906264 | Sh3bp2 |
| 2.598055 | up | AK162966 | chr11:61705399-61705340 | Akap10 |
| 4.5711622 | up | AK032648 | chr13:109910773-109910832 | Pde4d |
| 2.7145049 | up | NM_177411 | chr10:128115593-128115534 | Rab5b |
| 3.1968514 | up | NR_004051 | chr17:34625272-34625213 | Btnl5-ps |
| 2.200346 | up | NM_001003911 | chr9:90094878-90094937 | Adamts7 |
| 8.3059443 | up | NM_027655 | chr8:108233975-108234034 | 4933405L10Rik |
| 3.9145886 | up | NM_010588 | chr12:114146946-114146887 | Jag2 |
| 2.0899755 | up | NM_010756 | chr11:120490380-120490321 | Mafg |
| 4.2467377 | up | AK088506 | chr19:45836443-45836384 | Mgea5 |
| 2.8432383 | up | NM_020570 | chr5:25196103-25196044 | Xrcc2 |
| 2.3847466 | up |  | chrX:067673772-067673713 |  |
| 2.7705204 | up | NM_001005748 | chr13:43229312-43229371 | Phactr1 |
| 34.2870577 | up | AK157102 | chr13:93696217-93696158 | Gm10753 |
| 2.7186379 | up | NM_023663 | chr16:97963608-97963549 | Ripk4 |
| 3.0652272 | up | NM_001282017 | chr3:94836686-94836627 | Psmd4 |
| 3.4405733 | up | NM_033444 | chr17:35195587-35195646 | Clic1 |
| 2.8754445 | up |  | chr18:088492419-088492478 |  |
| 3.2028606 | up | NM_153807 | chr11:94418607-94418548 | Acsf2 |
| 2.3711912 | up | NM_173867 | chr4:140277252-140277311 | Rcc2 |
| 57.6073664 | up | NM_013650 | chr3:90473778-90473837 | S100a8 |
| 4.1885358 | up | NM_018729 | chr1:173515388-173515447 | Cd244 |
| 2.7233934 | up | CF969522 | chr11:88854469-88854410 |  |
| 7.5158366 | up | NM_028096 | chr1:37668656-37668597 | 2010300C02Rik |
| 5.0285216 | up | AK149188 | chr12:19437792-19437851 | Gm5784 |
| 3.4906241 | up | NM_026531 | chr7:86053197-86053256 | Aen |
| 5.2282919 | up | NM_025920 | chr1:95646575-95646516 | Thap4 |
| 2.2057282 | up | NM_011180 | chr11:118025748-118025689 | Cyth1 |
| 2.9890023 | up | XR_106165 | chr8:108161022-108148323 | Gm5914 |
| 3.4456042 | up | NM_012055 | chr6:7625228-7625171 | Asns |
| 3.7559673 | up | NM_026780 | chr4:134490381-134490440 | Syf2 |
| 3.8207723 | up | NM_198292 | chr11:106363455-106363448 | Tex2 |
| 3.1380373 | up | NM_011284 | chr4:132333990-132334049 | Rpa2 |
| 2.4213189 | up | NM_020044 | chr5:135076388-135076329 | Lat2 |
| 3.4674705 | up |  | chr16:018829472-018829531 |  |
| 3.1000524 | up | NM_001008700 | chr7:132722922-132722981 | Il4ra |
| 5.3978486 | up | NM_018884 | chr6:101100005-101099946 | Pdzrn3 |
| 3.8891045 | up | NM_023126 | chr8:74700227-74700286 | Rab8a |
| 2.1106992 | up |  | chr7:035000280-035000221 |  |
| 3.2976413 | up | NM_145153 | chr5:121307929-121307988 | Oas1f |
| 10.4900162 | up | NM_178795 | chr2:121162205-121162146 | Ppip5k1 |
| 106.993304 | up | NM_009117 | chr7:53996129-53996070 | Saa1 |
| 2.7928324 | up | NM_030558 | chr16:17835428-17835369 | Car15 |
| 5.2009927 | up | AK139292 | chr2:18194584-18194525 |  |
| 7.4540113 | up | NM_020618 | chr11:99071406-99071347 | Smarce1 |
| 12.1612667 | up | NM_027416 | chr13:3802236-3802177 | Calml3 |
| 4.7164455 | up | NM_001002008 | chr17:21725548-21725607 | Zfp948 |
| 4.1655486 | up |  | chr1:052641966-052642025 |  |
| 3.5342877 | up | AK137931 | chr3:79056191-79056132 |  |
| 3.0058012 | up |  | chr13:077274995-077274936 |  |
| 2.0117435 | up | NM_001113356 | chr6:124525309-124525368 | C1rb |
| 4.0137585 | up | NM_026695 | chr7:50699462-50699521 | Etfb |
| 2.5298825 | up | NM_001243002 | chrX:70420933-70420874 | Spin2d |
| 13.4393243 | up |  | chr7:131102865-131102924 |  |
| 4.6814573 | up | NM_177567 | chr11:51067255-51067196 | BC049762 |
| 2.3196007 | up |  | chr6:127619086-127619145 |  |
| 3.5125102 | up | AK153817 | chr11:104266983-104266924 | D11Ertd726e |
| 3.0968167 | up | NM_027307 | chr13:59736525-59736466 | Golm1 |
| 8.2467833 | up | NM_001042711 | chr3:113084840-113084781 | Amy2a5 |
| 7.3884442 | up | NM_001254953 | chr17:43671940-43671881 | Ankrd66 |
| 4.0821427 | up | NM_029116 | chr8:15027026-15027085 | Kbtbd11 |
| 2.1215044 | up | XR_107383 | chr2:104842303-104842244 | LOC552890 |
| 6.6778579 | up | NM_001163415 | chr9_random:47791-47732 | Ppp2r3d |
| 2.1480387 | up | BB453506 | chr2:53022723-53022664 |  |
| 5.1912393 | up | NM_145997 | chr6:120380280-120381871 | Kdm5a |
| 3.4283562 | up | AK170523 | chr17:28318896-28318955 | Zfp523 |
| 2.4501718 | up | NM_199311 | chr6:122884578-122884637 | Clec4a1 |
| 4.2720138 | up | NM_010266 | chr19:21465882-21465823 | Gda |
| 2.8743919 | up |  |  |  |
| 2.3833982 | up | NM_013685 | chr18:69842843-69842902 | Tcf4 |
| 2.9957246 | up | NM_025811 | chr19:56673142-56673201 | Nhlrc2 |
| 3.60516 | up | NM_009871 | chr11:80293969-80294028 | Cdk5r1 |
| 2.2584798 | up | NM_009212 | chr19:3259483-3259424 | Ighmbp2 |
| 4.0664475 | up | NM_001159344 | chr4:148327349-148327408 | Casz1 |
| 2.5410134 | up | NM_172605 | chr14:87945240-87945299 | Tdrd3 |
| 2.9142802 | up | NM_012018 | chr2:35034211-35034270 | Cep110 |
| 2.3366494 | up | NM_175111 | chr16:35828473-35828532 | Hspbap1 |
| 3.9808431 | up | AK044695 | chrX:7196193-7196252 | Cacna1f |
| 3.4297965 | up | NM_019518 | chr15:101063123-101063182 | Grasp |
| 7.2562794 | up |  | chr2:029906910-029906969 |  |
| 3.1084039 | up | NM_001145978 | chr14:57278506-57278565 | Parp4 |
| 11.6994516 | up | NR_028384 | chr4:21775079-21775020 | 4930528A17Rik |
| 2.1540901 | up | NM_001083118 | chr8:109599479-109599420 | Terf2 |
| 12.8295032 | up | NM_019943 | chr5:143004380-143004321 | Papolb |
| 2.3954732 | up | NM_027968 | chr10:11011329-11015032 | Fbxo30 |
| 50.9516514 | up | NM_001169153 | chr11:114977855-114977796 | Cd300lf |
| 16.4719449 | up |  | chr8:064151123-064151182 |  |
| 5.067727 | up | NM_001136086 | chr18:43483940-43483881 | Dpysl3 |
| 2.1073472 | up | NM_008422 | chr7:51859259-51859318 | Kcnc3 |
| 8.8492341 | up | AK042816 | chr8:48589658-48589599 | Trappc11 |
| 10.5681812 | up | NM_010615 | chr19:37495755-37495814 | Kif11 |
| 7.5060557 | up |  |  |  |
| 2.7212585 | up | NM_019726 | chr11:69730034-69730093 | Gps2 |
| 4.0281014 | up | NM_207651 | chr18:78342970-78342911 | Slc14a2 |
| 2.357569 | up | NM_021895 | chr7:29678348-29678289 | Actn4 |
| 2.4563668 | up | NM_053168 | chr11:58804863-58804922 | Trim11 |
| 4.1445487 | up | NM_011040 | chr2:24276206-24276147 | Pax8 |
| 3.5780975 | up | NM_144904 | chr4:59527206-59527147 | Ptbp3 |
| 3.4444055 | up | XM_003085120 | chr17:8094998-8094971 | Gm8457 |
| 2.4260502 | up | NM_026054 | chr6:149283926-149283985 | 2810474O19Rik |
| 2.2334166 | up | NM_207205 | chr3:101264580-101264639 | Igsf3 |
| 2.3755705 | up | NR_002889 | chr4:155331600-155331655 | Gm5801 |
| 4.8822606 | up | AK075670 | chr1:141981344-141981285 | Cfh |
| 8.9152571 | up | NM_013623 | chr4:63018910-63020393 | Orm3 |
| 9.0122916 | up | NM_029951 | chrX:34393386-34393327 | C330007P06Rik |
| 13.3979001 | up | NM_007746 | chr18:4331658-4331599 | Map3k8 |
| 3.3044748 | up | NM_138667 | chr10:7626064-7626005 | Tab2 |
| 3.302854 | up | NM_207202 | chrX:7309502-7309443 | Ccdc120 |
| 49.4766872 | up |  | chr14:006548593-006548534 |  |
| 2.6593211 | up | NM_010178 | chr4:135424198-135424257 | Srsf10 |
| 4.4639442 | up | AK158814 | chr10:95022468-95022409 | C81189 |
| 6.4078972 | up | NM_001270542 | chr7:32498851-32498910 | Scgb1b7 |
| 3.6822843 | up | NM_028894 | chrX:33906756-33906815 | Lonrf3 |
| 4.577237 | up | AK029808 | chr4:44924836-44924895 | Zcchc7 |
| 2.7707182 | up | NM_001081337 | chr8:127942073-127942014 | Sipa1l2 |
| 5.2219089 | up | NM_146224 | chr9:72209853-72209912 | Zfp280d |
| 2.6849477 | up | NM_001113470 | chr10:126436972-126437031 | Ctdsp2 |
| 4.6019761 | up | AK020613 | chr8_random:677586-677527 | 9530057J20Rik |
| 2.1996002 | up |  | chr15:095632523-095632464 |  |
| 2.3039321 | up | NM_009367 | chr1:188449392-188449333 | Tgfb2 |
| 2.8869458 | up | NM_144549 | chr15:59487662-59487721 | Trib1 |
| 17.4103922 | up | AK031795 | chr5:20530466-20530407 | BB070754 |
| 2.700747 | up | NM_001276350 | chr18:6489218-6489159 | Epc1 |
| 2.1049096 | up | NM_177279 | chr8:125745721-125745780 | Spata33 |
| 2.9278345 | up | AK160395 | chr10:17619047-17618988 | Heca |
| 2.713045 | up | NM_001277944 | chr7:20257086-20257027 | Apoc2 |
| 49.5080699 | up | AI987986 | chr14:27022692-27022751 | AI987986 |
| 2.6377448 | up | AK148316 | chr17:30347694-30347753 | Zfand3 |
| 15.0879929 | up | NM_007679 | chr16:15888459-15888518 | Cebpd |
| 3.1097552 | up | NM_001142918 | chr19:56006638-56006697 | Tcf7l2 |
| 2.1174912 | up |  | chr7:054263862-054263921 |  |
| 2.1173298 | up | AK041359 | chr4:46646548-46646489 | Tbc1d2 |
| 2.5162338 | up | HQ446585 | chr6:69450442-69450383 |  |
| 4.2780312 | up |  | chr10:069072752-069072693 |  |
| 3.3958137 | up | NM_022009 | chr11:60532411-60532352 | Flii |
| 2.8100288 | up | NM_019750 | chr9:107486119-107486178 | Nat6 |
| 2.3437562 | up | NM_181418 | chr8:73908232-73908173 | Ushbp1 |
| 2.1418133 | up | NM_001256180 | chr3:96237777-96237718 | BC107364 |
| 3.9157851 | up | NM_001195065 | chr10:12933786-12933727 | Phactr2 |
| 10.7184813 | up |  | chr14:025563451-025563392 |  |
| 4.4444195 | up | NM_152813 | chr11:102931688-102931629 | Plcd3 |
| 2.0532994 | up | NM_199299 | chr11:51627020-51626961 | Jade2 |
| 2.6585876 | up | NM_001163819 | chr17:28463401-28463460 | Fance |
| 3.8949721 | up | NM_001278256 | chr2:131744512-131744571 | Prnp |
| 5.3306483 | up |  | chr3:094506773-094506832 |  |
| 3.5590539 | up | AK135304 | chr15:38173131-38173072 |  |
| 2.1153823 | up | NM_212450 | chr2:121819108-121819167 | Ctdspl2 |
| 2.0171089 | up | NM_030258 | chr5:139872290-139872349 | Gpr146 |
| 2.7211793 | up | NM_009899 | chr3:144393509-144393450 | Clca1 |
| 2.381887 | up | NM_025303 | chr1:16335846-16335787 | Stau2 |
| 8.2950156 | up | NM_009793 | chr18:33355362-33355421 | Camk4 |
| 6.9957333 | up | NM_019680 | chrX:45766717-45766658 | Elf4 |
| 9.5813829 | up | AK080903 | chr13:114889712-114889771 | B430203I24Rik |
| 2.3716113 | up | NM_010194 | chr7:87522702-87522643 | Fes |
| 2.8180394 | up |  | chr14:114588499-114588440 |  |
| 2.1319334 | up | NM_010437 | chr10:13870069-13870128 | Hivep2 |
| 4.0114497 | up | BC093506 | chr4:46482861-46482920 | Anp32b |
| 12.6641584 | up | AK016661 | chr3:122567827-122567768 | 4933405D12Rik |
| 2.2068557 | up | NM_021544 | chr9:119394907-119394848 | Scn5a |
| 8.2808961 | up | AK082443 | chr11:65934670-65934611 | Dnah9 |
| 16.9538603 | up | NM_001024141 | chrX:132607052-132606993 | Nxf3 |
| 3.6808246 | up | NM_001282126 | chr6:113274570-113274629 | Brpf1 |
| 3.3151543 | up | NM_016748 | chr4:120212548-120212489 | Ctps |
| 3.0411395 | up | XM_003085153 | chr17:65226384-65226443 | Gm4701 |
| 4.7949042 | up | NM_001253386 | chr4:129931303-129931244 | Serinc2 |
| 3.5325647 | up | NM_134060 | chr13:39024386-39024327 | Slc35b3 |
| 3.8516791 | up | NM_001017966 | chr4:141239825-141239766 | Ddi2 |
| 5.2008232 | up | NM_013692 | chr15:38224314-38224255 | Klf10 |
| 3.0305633 | up | NM_134086 | chr15:96406852-96406793 | Slc38a1 |
| 20.1703121 | up | BY709204 | chr4:82501947-82502006 | Gm11266 |
| 2.6571635 | up | XR_168777 | chr19:6530945-6530886 |  |
| 2.5539123 | up | NM_001161798 | chr4:147430258-147430317 | Mthfr |
| 2.7577372 | up | BC026478 | chr4:117541416-117541357 | Ccdc24 |
| 2.9131399 | up | NM_024468 | chr17:36396085-36396026 | Trim39 |
| 19.1422326 | up | NM_008037 | chr5:32455088-32455147 | Fosl2 |
| 2.8414631 | up | NM_001110824 | chr17:48004772-48004713 | Foxp4 |
| 6.1616383 | up | NM_145482 | chr16:93583765-93583706 | Setd4 |
| 2.4133548 | up | NM_001002764 | chr11:74977872-74977931 | Smg6 |
| 86.0824181 | up | NM_031168 | chr5:30346367-30346426 | Il6 |
| 2.483214 | up | NM_178279 | chr14:8996590-8996649 | Pxk |
| 2.9014969 | up |  | chr15:085969567-085969508 |  |
| 2.3750362 | up | NM_001205239 | chr9:59498017-59498076 | Parp6 |
| 4.1099362 | up | AK134268 | chr2:120189107-120189048 | Tmem87a |
| 5.8726905 | up |  | chr7:026203341-026203282 |  |
| 2.4510885 | up | AK162913 | chr2:155073316-155073257 | Pigu |
| 5.7455818 | up | AK031601 | chr8:20009955-20009896 |  |
| 2.8256916 | up | NM_001045483 | chr7:146027590-146027531 | Mapk1ip1 |
| 6.1902342 | up | AK085126 | chr5:92558851-92558910 | D430040L24Rik |
| 2.4318378 | up | NM_011957 | chr2:91822565-91822506 | Creb3l1 |
| 2.1169204 | up | NM_023248 | chr5:130721771-130721712 | Sbds |
| 12.6262822 | up | NM_009491 | chr5:109425025-109424966 | Vmn2r10 |
| 2.8337549 | up | NM_001166581 | chr5:104949957-104950016 | BC005561 |
| 3.4005137 | up | NM_001024952 | chr1:162895873-162897790 | Rc3h1 |
| 2.4589561 | up | NM_029291 | chr11:4583248-4583307 | Ascc2 |
| 8.726954 | up | AK030805 | chr16:50372798-50372739 | A730021G18Rik |
| 2.779873 | up | NM_001177884 | chr5:34218806-34218865 | Whsc1 |
| 3.5240674 | up | NM_176849 | chr8:8683865-8683806 | Arglu1 |
| 2.619085 | up | NR_033332 | chr11:53599050-53598991 | Gm12216 |
| 4.3329963 | up | NM_016872 | chr6:72318207-72318148 | Vamp5 |
| 6.1597867 | up | AK016515 | chr5:113246309-113246250 | Myo18b |
| 2.2392435 | up | AK038155 | chr1:140058573-140058514 | A130071D04Rik |
| 13.9021297 | up | AK049998 | chr15:79746081-79746140 | Apobec3 |
| 2.9674847 | up | NM_175093 | chr2:152163220-152163161 | Trib3 |
| 2.2052344 | up |  | chr8:053281203-053281144 |  |
| 2.2983635 | up | NM_176916 | chr1:177892526-177892467 | Pld5 |
| 2.7498559 | up | NR_024078 | chr4:116791882-116791823 | Btbd19 |
| 5.3191731 | up | NR_024257 | chr2:9808308-9808367 | 4930412O13Rik |
| 6.7134144 | up | NM_001034097 | chr11:69496138-69496079 | BC096441 |
| 13.8461118 | up | NM_020033 | chr19:42118566-42118625 | Ankrd2 |
| 4.4934826 | up |  | chr12:047579821-047579762 |  |
| 30.793452 | up |  | chr14:042737009-042737068 |  |
| 2.2804125 | up | AK079275 | chr11:61692304-61692245 | A430106A12Rik |
| 5.0227832 | up | NM_019445 | chr1:176752018-176752077 | Fmn2 |
| 2.7098369 | up |  | chr9:083238640-083238699 |  |
| 2.0151655 | up |  | chr4:043613144-043613085 |  |
| 73.2386148 | up | NM_001160399 | chr18:46447337-46447278 | Ccdc112 |
| 3.1487473 | up | NM_175164 | chr18:39535145-39535204 | Arhgap26 |
| 2.1254525 | up | NM_011197 | chr3:100844926-100844867 | Ptgfrn |
| 3.2232934 | up | NM_029568 | chr11:61302147-61302206 | Mfap4 |
| 17.3555899 | up | NM_011517 | chr10:87935675-87935734 | Sycp3 |
| 2.1400751 | up | AK028871 | chr7:28425375-28425434 | Akt2 |
| 2.2698107 | up | NM_178637 | chr19:5603076-5603017 | Kat5 |
| 25.0494791 | up | NM_026571 | chr17:28720438-28720497 | Lhfpl5 |
| 3.4131599 | up | NM_029865 | chr8:73896997-73897056 | Ocel1 |
| 3.0382416 | up | NM_028460 | chr3:87553208-87553149 | Pear1 |
| 3.974856 | up | NR_028425 | chr7:3220957-3221016 | D7Ertd143e |
| 9.0064153 | up | XR_168434 | chr7:116898572-116898630 | 4930431P19Rik |
| 15.9732171 | up | NM_001033773 | chr4:100162746-100162805 | Ube2u |
| 2.6062858 | up | NM_011330 | chr11:81876072-81876131 | Ccl11 |
| 3.1843228 | up | NM_008575 | chr1:134886485-134886426 | Mdm4 |
| 4.3457916 | up | NM_198934 | chr1:167805698-167805639 | Pou2f1 |
| 3.339869 | up | NM_175445 | chr2:131818699-131818640 | Rassf2 |
| 5.5389672 | up | NR_002862 | chr14:90299026-90299085 | Gm5088 |
| 2.7730526 | up | NM_027658 | chr11:103000446-103000505 | Hexim2 |
| 6.3669701 | up | NM_001014390 | chr10:118296701-118296642 | Dyrk2 |
| 6.9329497 | up | NM_025620 | chr6:146981900-146981959 | Rep15 |
| 5.0514816 | up | XM_003945462 | chr10:7512380-7512321 | 6530403G13Rik |
| 5.8540822 | up | NM_139198 | chr5:100982856-100982797 | Plac8 |
| 5.2213092 | up | AY512949 | chr4:104204781-104204840 | AY512949 |
| 4.6923187 | up | NM_008851 | chr19:4113906-4113965 | Pitpnm1 |
| 13.1587496 | up | NM_007986 | chr2:62339060-62339001 | Fap |
| 7.0350906 | up | AK004108 | chr8:109837834-109837893 | 1110035E04Rik |
| 8.12566 | up | NM_172460 | chr9:103918902-103918961 | Nphp3 |
| 2.8413361 | up | NM_133915 | chr5:116004015-116004074 | Pxn |
| 4.3993275 | up | NM_025747 | chr8:47255818-47255877 | 4933411K20Rik |
| 6.0182832 | up | NM_027560 | chr8:73359113-73359054 | Arrdc2 |
| 2.811238 | up | NM_031380 | chr10:79244916-79244975 | Fstl3 |
| 7.1536548 | up |  | chr18:046801187-046801246 |  |
| 3.8583125 | up | NM_028261 | chr18:35893751-35893692 | Tmem173 |
| 2.7425996 | up | NM_001163576 | chr15:76063492-76063433 | Parp10 |
| 2.2182301 | up | NM_178119 | chr1:91791793-91791852 | Agap1 |
| 8.0534056 | up | NM_010918 | chr9:121657241-121657300 | Nktr |
| 3.1534142 | up | NM_001081956 | chrX:34151933-34151874 | Akap17b |
| 4.052122 | up | AK134048 | chr15:80448346-80448405 | 5830419E12Rik |
| 4.0517619 | up | NM_008272 | chr15:102814735-102814794 | Hoxc9 |
| 2.0167217 | up | NM_026314 | chr9:72820802-72820861 | Dyx1c1 |
| 2.0466191 | up | NM_010919 | chr2:147009008-147008949 | Nkx2-2 |
| 2.344385 | up | AK016858 | chr8:79837140-79837199 | 4933421D24Rik |
| 5.6858506 | up | NM_178250 | chr2:87329326-87329267 | Pramel7 |
| 2.09232 | up | NM_053140 | chr18:37635651-37635710 | Pcdhb15 |
| 3.2832873 | up | NM_030693 | chr7:52067686-52067627 | Atf5 |
| 15.9913519 | up |  | chr16:086053378-086053319 |  |
| 2.2776373 | up | NM_139206 | chr18:38132443-38132384 | Arap3 |
| 4.8810922 | up | NM_153599 | chr5:147043316-147043375 | Cdk8 |
| 4.6283575 | up | AK010742 | chr2:165636473-165636532 | 2410087M07Rik |
| 2.6380392 | up | NM_181415 | chr19:57806075-57806134 | Atrnl1 |
| 8.5339224 | up | XR_105114 | chr7:46701486-46701427 | Gm5590 |
| 2.3392191 | up |  | chr13:85266663-85266604 |  |
| 2.9337928 | up | XR_106126 | chr7:28605803-28605862 | Zfp626 |
| 2.1261966 | up | NM_001013780 | chr4:141174806-141174747 | Slc25a34 |
| 2.6985642 | up | NM_028242 | chrX:54319366-54319425 | Htatsf1 |
| 3.0256498 | up | AK085995 | chr10:60936383-60936324 | D830039M14Rik |
| 2.4348935 | up | NM_010577 | chr15:103175081-103175022 | Itga5 |
| 8.4286934 | up | NM_080795 | chr5:147828891-147828832 | Lnx2 |
| 2.4739597 | up | BC017131 | chr8:125756097-125756158 | Cdk10 |
| 2.0837878 | up | NM_053104 | chr15:76914343-76914284 | Rbfox2 |
| 2.1636363 | up | NM_020507 | chr15:81679547-81679488 | Tob2 |
| 5.3106281 | up | AK079970 | chr16:19244060-19244119 | A530030E21Rik |
| 13.7010219 | up | AK021218 | chr13:66443281-66443222 | C330022B21Rik |
| 5.0200631 | up |  | chr14:043542015-043542074 |  |
| 10.2817148 | up | NM_011331 | chr11:81916764-81916823 | Ccl12 |
| 6.0264997 | up | NM_001099631 | chr4:137815848-137815907 | Sh2d5 |
| 3.2839223 | up | NM_010120 | chr18:46768754-46768813 | Eif1a |
| 4.6126825 | up | NM_001122992 | chr4:131781669-131781610 | Gmeb1 |
| 2.2420136 | up | NM_011145 | chr17:28438298-28438357 | Ppard |
| 3.0045252 | up | NM_023738 | chr9:107886328-107886387 | Uba7 |
| 11.2058641 | up | AK051660 | chr13:99568627-99568568 | D130062J10Rik |
| 8.4450408 | up | NM_001011867 | chr7:147760787-147760846 | Olfr538 |
| 2.5985838 | up | NM_134048 | chr12:32171997-32171938 | Cbll1 |
| 3.0373223 | up | NM_013737 | chr17:43749032-43749091 | Pla2g7 |
| 3.1652832 | up | NM_001123371 | chr14:51584365-51584424 | Pnp2 |
| 6.2861269 | up | NM_001082545 | chr16:36404089-36404032 | Stfa2 |
| 3.1376926 | up | NM_001037722 | chr3:89143622-89143563 | Adam15 |
| 9.6211297 | up | NM_030720 | chr15:103138729-103138670 | Gpr84 |
| 3.1310914 | up |  | chr5:93037341-93037282 | Ccdc158 |
| 2.0390378 | up | AK014919 | chr13:49655644-49655585 | Cenpp |
| 3.5168944 | up | AK156275 | chr4:5356416-5356357 |  |
| 2.0545572 | up | NM_001003949 | chr10:79439924-79439865 | Tmem259 |
| 4.0125184 | up | NM_001081387 | chr2:172919169-172919110 | Ctcfl |
| 3.2189088 | up | NM_028820 | chr9:57101661-57101602 | 1700017B05Rik |
| 4.9672391 | up | XM_003945517 | chr13:99483110-99483051 | Gm9465 |
| 29.9167054 | up | AK135408 | chr11:106962178-106962119 | Gm4098 |
| 5.9571612 | up |  | chrX:066247582-066247641 |  |
| 31.443393 | up | BC026387 | chr15:75931971-75931912 | Eppk1 |
| 6.1260749 | up | NM_138598 | chr11:113523086-113523027 | Fam104a |
| 2.8381326 | up | NM_194345 | chr14:70983437-70983378 | Fam160b2 |
| 2.7299698 | up | NM_145515 | chr1:186722262-186722203 | Mark1 |
| 2.9246326 | up | NM_011691 | chr17:57467307-57467366 | Vav1 |
| 3.8783177 | up | NM_009780 | chr17:34865387-34865328 | C4b |
| 3.4974305 | up | BB325154 | chr12:004482322-004482263 |  |
| 2.218104 | up | NM_023054 | chr5:88985007-88985066 | Utp3 |
| 2.8787829 | up | NM_178337 | chr13:14100910-14098774 | Tbce |
| 28.4810178 | up | NM_013606 | chr16:97782327-97782386 | Mx2 |
| 25.7254071 | up | NM_008333 | chr4:88466410-88466469 | Ifna11 |
| 5.4688129 | up | NM_175308 | chr4:115508702-115508761 | Mob3c |
| 10.3280909 | up | NM_145209 | chr5:115387861-115387920 | Oasl1 |
| 2.1003984 | up | NM_011492 | chr10:79590785-79590844 | Stk11 |
| 10.4756423 | up | NM_013468 | chr19:36186868-36186809 | Ankrd1 |
| 2.4995459 | up | NM_009510 | chr17:6942666-6942607 | Ezr |
| 22.4993279 | up | NM_011819 | chr8:73153352-73153293 | Gdf15 |
| 2.2608715 | up | NM_019834 | chr5:115179848-115179789 | Git2 |
| 2.7771681 | up | NR_026907 | chr5:135476389-135476330 | Wbscr25 |
| 4.7937677 | up | NM_133721 | chr9:118807900-118807959 | Itga9 |
| 4.2308235 | up | NM_010107 | chr3:89075762-89075703 | Efna1 |
| 2.2713603 | up | NM_177377 | chr4:43439997-43439938 | Fam166b |
| 25.1966233 | up | NM_001081567 | chr5:103355162-103355103 | Mapk10 |
| 2.2591803 | up | NM_001163610 | chrX:99278207-99278266 | Nhsl2 |
| 2.1044497 | up |  | chr7:086843338-086843397 |  |
| 2.6507186 | up | NM_001081152 | chr9:53383673-53383732 | Npat |
| 2.5944757 | up | NM_009842 | chr7:148656555-148656614 | Cd151 |
| 2.1769831 | up | NM_011538 | chr7:133929000-133929059 | Tbx6 |
| 4.5839897 | up | AK044226 | chr11:107532715-107532774 | Helz |
| 3.0597257 | up | NM_001122948 | chr13:40811103-40811044 | Tfap2a |
| 2.0234427 | up | NM_021303 |  | Noc2l |
| 4.8337454 | up | NM_007918 | chr8:28385887-28385946 | Eif4ebp1 |
| 2.3383794 | up | NM_172119 | chr12:111518792-111518851 | Dio3 |
| 10.3474473 | up | XR_168729 | chr14:54969202-54969143 | 5330426L24Rik |
| 2.4170738 | up | NM_010153 | chr10:128006483-128006424 | Erbb3 |
| 18.7273609 | up | NM_153761 | chr7:19443884-19443943 | Mill2 |
| 2.8931084 | up | NM_001142747 | chr10:73969284-73969343 | Pcdh15 |
| 3.1642851 | up | NM_001177371 | chr13:55575437-55575378 | Dbn1 |
| 3.0151093 | up | NM_001005784 | chr4:98164262-98164321 | Inadl |
| 12.6324853 | up | NM_001166206 | chr2:131681477-131681418 | Erv3 |
| 4.1095841 | up | AK049699 | chr1:69045020-69044961 | C530042K13Rik |
| 7.1681459 | up | AK034835 | chr3:8927959-8928018 | 9130403I23Rik |
| 2.0801904 | up | NM_011808 | chr9:32561826-32561885 | Ets1 |
| 3.1912172 | up | XR_105627 | chr14:75332991-75333050 | Gm10847 |
| 2.8562921 | up | NM_001045486 | chr7:24892175-24892234 | Zfp180 |
| 2.6970925 | up | NM_027530 | chr5:89071637-89071696 | Rufy3 |
| 2.9788366 | up | NM_008679 | chr2:165898682-165898741 | Ncoa3 |
| 5.3383689 | up |  | chr12:083595728-083595669 |  |
| 9.8220318 | up | AK014508 | chr8:83539505-83539564 | Gm9725 |
| 2.0232358 | up | AK043043 | chr8:4348678-4348737 | Ccl25 |
| 3.1187761 | up | NM_007570 | chr1:135972153-135972094 | Btg2 |
| 2.1130985 | up | NM_001104645 | chr7:13237991-13237932 | Vmn2r55 |
| 2.7406494 | up | NM_028536 | chr8:126364804-126364745 | Ccsap |
| 67.1783753 | up | NM_025367 | chr11:116397908-116397967 | Sphk1 |
| 3.4370268 | up | C78535 | chr7:125336397-125336456 | D7Ertd183e |
| 8.3870973 | up |  | chrY_random:057880655-057880596 | |
| 5.6666355 | up | AK133045 | chr1:157821558-157821617 | Gm9694 |
| 2.4458493 | up | NM_011319 | chr3:108229598-108229539 | Sars |
| 6.9519803 | up | NM_008273 | chr2:74525014-74525073 | Hoxd11 |
| 2.3450505 | up | NM_023348 | chr16:17428424-17428483 | Snap29 |
| 2.7161168 | up | NM_001167963 | chr12:52466242-52466301 | G2e3 |
| 2.2539247 | up | BC117807 | chr4:128762917-128762976 | Rnf19b |
| 2.4654346 | up | NM_001285927 | chr1:80498092-80498033 | Dock10 |
| 5.5327844 | up | AK156640 | chr2:59606985-59606926 |  |
| 3.0449235 | up | NM_007987 | chr19:34402164-34402223 | Fas |
| 2.0423895 | up |  | chr17:023779387-023779446 |  |
| 12.1971147 | up | NR_037975 | chr3:137711818-137711759 | Gm5105 |
| 5.5061206 | up | NM_009851 | chr2:102654337-102654278 | Cd44 |
| 92.6824965 | up | NM_008230 | chr2:126419575-126419516 | Hdc |
| 3.6157785 | up | AK050942 | chr8:131653716-131653657 |  |
| 10.380435 | up | NM_008381 | chr1:121312219-121312160 | Inhbb |
| 3.5055731 | up | NM_011659 | chr4:155390639-155390698 | Tnfrsf4 |
| 3.1541173 | up | NM_011699 | chr2:109737705-109737764 | Lin7c |
| 4.0277091 | up | NM_030682 | chr5:65316174-65316115 | Tlr1 |
| 11.6928969 | up | NM_001008533 | chr1:136095892-136095833 | Adora1 |
| 2.7170737 | up | AK090020 | chr2:164328561-164328620 | Pigt |
| 4.5262015 | up | AK082458 | chr2:26289596-26289537 | Sec16a |
| 2.3822825 | up | BC024859 | chr17:35379420-35379479 | Ddx39b |
| 3.1836947 | up | NM_178098 | chr13:60944319-60944260 | 4930486L24Rik |
| 2.2532057 | up | NM_001025360 | chr12:113044907-113044966 | Klc1 |
| 2.4224695 | up | NM_019995 | chr3:54516023-54516082 | Supt20 |
| 112.013546 | up | NM_001033324 | chr9:48462478-48462419 | Zbtb16 |
| 2.1052171 | up | NM_175482 | chr9:48850540-48850599 | Usp28 |
| 7.4234015 | up | XR_105474 | chr12:8485935-8485894 | BC106175 |
| 12.0694456 | up |  | chr1:003522754-003522813 |  |
| 17.3821881 | up | NM_172612 | chr15:98503035-98502976 | Rnd1 |
| 2.1128795 | up | NM_010757 | chr5:140278221-140278280 | Mafk |
| 3.0219542 | up | NM_001243021 | chr7:46732717-46732776 | Zfp939 |
| 2.1166797 | up | NM_007951 | chr12:81735069-81735010 | Erh |
| 2.7600741 | up | NM_009422 | chr2:25374445-25374386 | Traf2 |
| 3.5541855 | up | NM_027410 | chr5:144956373-144956314 | Tecpr1 |
| 34.0960994 | up | NM_080457 | chr16:32782278-32782337 | Muc4 |
| 3.8650793 | up | NM_019795 | chr11:100444220-100444161 | Dnajc7 |
| 4.1193594 | up | AK138015 | chr16:23236477-23236536 | BB163080 |
| 2.7319404 | up | NM_145377 | chr11:48619979-48619920 | Trim41 |
| 34.026185 | up | XR_035282 | chr9:101845249-101845308 | 9630041A04Rik |
| 2.5243272 | up | NM_009518 | chr1:74850633-74850692 | Wnt10a |
| 3.5052995 | up | AK046753 | chr3:115603768-115603827 | Dph5 |
| 2.3064511 | up | NM_001003920 | chr7:4667538-4667597 | Brsk1 |
| 2.5513775 | up | NM_009986 | chr5:136755401-136755342 | Cux1 |
| 2.3369653 | up | NM_016919 | chr9:20574651-20574592 | Col5a3 |
| 7.8217439 | up | AK019978 | chr13:52991384-52991325 | Auh |
| 6.0863487 | up | NM_001145874 | chr16:32777571-32777512 | Muc20 |
| 2.0016624 | up | NM_009353 | chr8:109600760-109600701 | Terf2 |
| 149.8114934 | up | NR_003548 | chr3:92179092-92179151 | Sprr2g |
| 4.7321966 | up | NM_176987 | chr13:54652565-54652624 | Simc1 |
| 2.0826429 | up | NM_001029979 | chr17:56702446-56702387 | Safb2 |
| 6.0150595 | up | NM_173749 | chr2:102483126-102483185 | Pamr1 |
| 2.2899657 | up | NM_133884 | chr4:133147563-133147622 | Gpn2 |
| 2.4723939 | up |  | chr11:098629386-098629445 |  |
| 2.7958598 | up | NR_045093 | chr15:54931687-54931628 | Gm9920 |
| 2.3254759 | up | AK047818 | chr13:110433307-110433366 | A730009E18Rik |
| 3.1775746 | up | AK134699 | chr7:35069331-35069272 | 4931406P16Rik |
| 13.1878456 | up | NM_197945 | chr2:130459382-130459323 | Lzts3 |
| 3.3096439 | up | NM_018790 | chr15:74499651-74499592 | Arc |
| 7.3272183 | up | NM_021487 | chrX:138740390-138740331 | Kcne1l |
| 7.8872698 | up | AK089567 | chr9:3017151-3017210 |  |
| 22.989607 | up | NM_021400 | chr1:152297126-152297067 | Prg4 |
| 4.6071929 | up | NM_028725 | chr8:120185411-120185352 | Sdr42e1 |
| 4.2472882 | up | NM_025661 | chr11:98442667-98442608 | Ormdl3 |
| 3.150662 | up | NR_033217 | chr8:67610184-67610125 | BC030870 |
| 5.0762225 | up | AK089791 | chr4:11738574-11738633 | Cdh17 |
| 2.1892771 | up |  | chr9:039860597-039860656 |  |
| 8.3470418 | up | AK050412 | chr7:72951188-72951247 | 1810008I18Rik |
| 4.5403799 | up |  | chr1:004870313-004870254 |  |
| 4.1061491 | up | NM_001252539 | chr4:115243923-115243982 | Cyp4a31 |
| 2.6381824 | up | NM_198294 | chr2:59681626-59681685 | Tanc1 |
| 4.648765 | up |  | chr3:022150502-022150561 |  |
| 3.0640637 | up | NM_028455 | chr15:84602532-84602591 | Arhgap8 |
| 2.4504879 | up | NM_001110824 | chr17:48004778-48004719 | Foxp4 |
| 18.0902319 | up |  | chr8:093262385-093262444 |  |
| 3.9044504 | up | NM_172722 | chr5:121890054-121890113 | Naa25 |
| 2.5735571 | up | NM_019662 | chr8:107152591-107152532 | Rrad |
| 2.5288825 | up |  | chr14:087316838-087316779 |  |
| 3.5252462 | up | AK136460 | chr6:108421112-108421053 |  |
| 2.202984 | up | NM_026407 | chr16:38591377-38591436 | Tmem39a |
| 3.4739669 | up |  | chr3:033903748-033903689 |  |
| 2.4263539 | up | NM_010469 | chr2:74566866-74566925 | Hoxd4 |
| 51.7324854 | up | NR_040767 | chr9:60865274-60865333 | Gm5122 |
| 2.6386365 | up | NM_080420 | chr11:87620136-87620077 | Lpo |
| 4.3300735 | up | NM_144881 | chr1:194520114-194520055 | Hhat |
| 3.5792805 | up | NM_177390 | chr11:80470941-80453875 | Myo1d |
| 6.4882639 | up | NM_021385 | chr6:112599602-112594696 | Rad18 |
| 5.2367753 | up | NM_145070 | chr5:124452389-124452448 | Hip1r |
| 4.4843954 | up | NM_011132 | chr5:110741964-110742023 | Pole |
| 2.1671896 | up | NM_011510 | chr7:53359980-53359921 | Abcc8 |
| 2.1095043 | up | NM_177239 | chr4:94608822-94608763 | Mysm1 |
| 2.3546712 | up | NM_172633 | chr18:86886865-86886924 | Cbln2 |
| 3.2746318 | up | AK032962 | chr11:20222485-20222426 | D930023I05Rik |
| 2.2657026 | up | NR_033146 | chrY_random:010464446-010464387 | Gm17821 |
| 2.6263751 | up | AK154578 | chr15:101043956-101044015 | Acvr1b |
| 2.0888342 | up | NM_020331 | chr5:134833618-134833559 | Gtf2ird1 |
| 2.6040263 | up |  | chr1:173268198-173268139 | Nit1 |
| 5.0565851 | up | XM_003945315 | chr1:82910557-82910616 | A030005L19Rik |
| 2.4018624 | up | AK015504 | chr5:24615868-24615927 |  |
| 2.4679574 | up | NM_144528 | chr10:79221407-79221348 | Rnf126 |
| 2.1787174 | up | XM_003946044 | chr1:137503176-137503117 | LOC101056010 |
| 2.3085904 | up |  | chr4:073159361-073159301 |  |
| 66.9437934 | up |  | chr7:011430866-011430807 |  |
| 25.3094105 | up | NM_145487 | chr17:36109594-36109535 | Prr3 |
| 2.624992 | up | NM_025461 | chr12:82571760-82571701 | Cox16 |
| 602.7720451 | up | NM_023256 | chr11:99289835-99289776 | Krt20 |
| 2.0280737 | up | AK038598 | chr7:74349027-74348968 | 1700112J16Rik |
| 4.5922742 | up | NR_028126 | chr4:3643888-3643947 | 6330407A03Rik |
| 79.7839696 | up | NM_009141 | chr5:91190490-91190549 | Cxcl5 |
| 69.0724025 | up | NM_026945 | chr3:137994039-137994098 | Adh6a |
| 3.0983884 | up | NM_198100 | chr11:96997546-96997487 | Tbkbp1 |
| 2.1302533 | up | NM_001163769 | chr17:31784139-31784080 | U2af1 |
| 8.6936945 | up | NM_007819 | chr5:138335471-138335412 | Cyp3a13 |
| 5.8345363 | up | NM_139227 | chr14:14936341-14936400 | Atxn7 |
| 5.2597018 | up | NM_176860 | chr9:40822158-40822099 | Ubash3b |
| 2.647629 | up | NR_028428 | chr8:19994758-19994699 | 2610005L07Rik |
| 4.5734853 | up | NM_010909 | chr17:35357180-35357121 | Nfkbil1 |
| 6.342339 | up | AK017368 | chr7:51740718-51740777 | 5430431A17Rik |
| 19.2831401 | up |  | chr18:047101919-047101860 |  |
| 2.0300257 | up | NM_019553 | chr10:62044921-62044862 | Ddx21 |
| 5.3951957 | up | NM_133834 | chr6:117873933-117873992 | Hnrnpf |
| 8.2391071 | up | NM_001164838 | chr9:111102268-111102327 | Lrrfip2 |
| 13.5348895 | up | NM_133218 | chr3:9430272-9430213 | Zfp704 |
| 10.6490586 | up | NM_001135151 | chr14:70703453-70703394 | Slc39a14 |
| 9.273933 | up | BC038884 | chr17:25377970-25378029 | Tsr3 |
| 2.7454126 | up |  | chrX:049281637-049281696 |  |
| 3.1907708 | up |  | chr1:017385601-017385660 |  |
| 5.6611769 | up | NM_173053 | chr11:3243445-3243386 | Limk2 |
| 2.1574169 | up | NM_010228 | chr5:148373850-148373791 | Flt1 |
| 2.3226521 | up | AK135384 | chr5:130386044-130386103 | Nupr1l |
| 2.200228 | up | AK032932 | chr9:79925329-79925388 | 6720474J12Rik |
| 3.9522138 | up | NM_145368 | chr4:49392787-49392728 | Acnat2 |
| 3.0310898 | up | AK083698 | chr9:96894619-96894560 | Spsb4 |
| 21.4440574 | up | NM_177718 | chr16:28827886-28827827 | Mb21d2 |
| 2.6382019 | up |  | chr9:114556938-114556879 |  |
| 3.0456982 | up | NM_011682 | chr10:12221287-12221228 | Utrn |
| 4.259224 | up | NM_001159953 | chr6:56073050-56072991 | Pde1c |
| 6.2245088 | up | NM_001043317 | chr7:31651308-31651249 | Cd22 |
| 2.6663996 | up | AK044967 | chr1:121487378-121487319 | AU019278 |
| 4.0914196 | up | NM_009948 | chr15:89246895-89246836 | Cpt1b |
| 10.7603269 | up | NM_001042501 | chr5:3559681-3561080 | Fam133b |
| 2.2459146 | up | NM_010432 | chr3:103547916-103547857 | Hipk1-2 |
| 9.7977264 | up | NM_053145 | chr18:37666914-37666973 | Pcdhb20 |
| 3.8877946 | up |  | chr14:044844891-044844950 |  |
| 3.6596736 | up | NM_001033769 | chrUn_random:2889834-2889775 | B020031M17Rik |
| 2.8720602 | up | NM_011672 | chr16:18834344-18834403 | Ufd1l |
| 2.4199597 | up | NM_001081086 | chr2:69574122-69574181 | Ppig |
| 3.951707 | up | NM_022883 | chr2:160731469-160731528 | Lpin3 |
| 3.3442646 | up | NM_021398 | chr2:84797987-84798046 | Slc43a3 |
| 2.6199168 | up | AK038868 | chr9:121707962-121707903 | Ccdc13 |
| 12.5579372 | up | NM_001039554 | chr4:147870043-147869984 | Angptl7 |
| 3.3311423 | up | NM_130860 | chr2:32563080-32563021 | Cdk9 |
| 3.894379 | up | NM_001172117 | chr2:152977080-152977139 | Hck |
| 2.075925 | up | NM_080455 | chr2:169711933-169711992 | Tshz2 |
| 2.1131013 | up | NM_173368 | chr2:160834146-160818015 | Chd6 |
| 4.7909827 | up | NM_153588 | chr16:13409769-13412310 | Mkl2 |
| 9.1476737 | up | NM_001163548 | chr5:144469066-144469125 | Cyth3 |
| 2.8741792 | up | NM_001146292 | chr18:25637675-25637616 | Celf4 |
| 7.9748081 | up |  | chr5:013770144-013770203 |  |
| 2.0333004 | up | AF141019 | chr17:13525609-13525668 | Rps6ka2 |
| 4.1478853 | up | NM_001204910 | chr1:175653722-175653663 | AI607873 |
| 2.298636 | up | NM_001139516 | chr2:157000820-157000761 | Rbl1 |
| 5.046517 | up | NM_001142920 | chr19:55994029-55994088 | Tcf7l2 |
| 3.6372889 | up |  | chrX:029775272-029775213 |  |
| 2.2610345 | up | NM_133641 | chr6:83102511-83102570 | Rtkn |
| 4.680785 | up | AK133183 | chr8:73694625-73694684 |  |
| 8.6153076 | up | NM_026473 | chr18:67562310-67562369 | Tubb6 |
| 2.7925525 | up | NM_001199147 | chr2:26202557-26202616 | Gpsm1 |
| 9.6693084 | up | NM_001271496 | chr19:3893508-3893567 | Chka |
| 3.4046684 | up | NR_033305 | chr17:34122175-34122234 | AA388235 |
| 4.0965051 | up | NM_021304 | chr5:31256786-31256925 | Abhd1 |
| 2.1062464 | up | AK028863 | chr14:63394713-63394654 | 4931440J10Rik |
| 2.18129 | up | NM_001037221 | chr14:47721800-47721859 | Samd4 |
| 4.4526463 | up | NM_009630 | chr10:74797461-74797520 | Adora2a |
| 34.285223 | up |  |  |  |
| 2.9530361 | up |  | chr7:058197666-058197607 |  |
| 2.5185452 | up |  | chr15:041754043-041754102 |  |
| 2.0345292 | up | AK135158 | chr8:106701744-106701685 | Gm10631 |
| 9.2819891 | up | NM_010784 | chr2:91771006-91770245 | Mdk |
| 2.5511337 | up | NM_172810 | chr14:63011586-63011527 | Gucy1b2 |
| 2.0004548 | up | NM_011029 | chr9:120040269-120040849 | Rpsa |
| 3.5871839 | up | M57966 | chr5:115398433-115398374 | Hnf1a |
| 4.2251396 | up | NM_001177302 | chr11:98836197-98836256 | Rara |
| 2.5881065 | up | NM_175286 | chr2:25125304-25125363 | Tprn |
| 6.8011264 | up | AK036131 | chr11:90124966-90125025 |  |
| 2.0827389 | up | CA496883 | chr15:36659282-36659341 | A830021M18Rik |
| 13.7015433 | up |  |  |  |
| 8.8814855 | up | NM_177635 | chr17:36685754-36685813 | H2-M11 |
| 15.4432876 | up | NM_178717 | chr15:10963536-10963477 | Rxfp3 |
| 2.3137035 | up | NM_001204233 | chr5:104868502-104868561 | Spp1 |
| 4.313832 | up | NM_030022 | chr5:141040412-141039628 | Grifin |
| 4.5932522 | up |  | chr13:065537619-065537560 |  |
| 5.5511205 | up | NM_028733 | chr2:91104768-91104827 | Pacsin3 |
| 4.0817307 | up |  | chr16:038477269-038477328 |  |
| 3.223778 | up | NM_025752 | chr19:42128034-42128093 | 4933411K16Rik |
| 2.2935033 | up | AI429812 | chr10:82112940-82112881 |  |
| 3.9534635 | up | NM_001142957 | chr17:33441423-33441482 | Zfp955b |
| 2.6837024 | up | BG072043 | chr6:41983091-41983032 | AU046084 |
| 3.0649637 | up | NM_001166584 | chr7:120049844-120049903 | Tead1 |
| 2.5888457 | up | NM_018878 | chr5:28067291-28067232 | Paxip1 |
| 3.744852 | up | NM_013490 | chr19:3864699-3864758 | Chka |
| 15.8627786 | up | NM_010849 | chr15:61821417-61821476 | Myc |
| 6.8987597 | up | NM_175370 | chr1:58715075-58715016 | Als2cr12 |
| 8.1172734 | up | NM_001081428 | chr10:53366857-53366798 | Fam184a |
| 4.6452138 | up | AK016440 | chr14:22688093-22688034 | 4931407E12Rik |
| 2.2312103 | up | NM_011057 | chr15:79826435-79826376 | Pdgfb |
| 3.5904481 | up | NM_001085491 | chr4:129169137-129169196 | Fam229a |
| 7.2598438 | up | NM_178912 | chr12:66232171-66232230 | Fancm |
| 7.5556428 | up | NR_004414 | chr11:101488770-101488829 | Rnu2-10 |
| 3.4566284 | up |  |  |  |
| 3.2147405 | up | AK082690 | chr2:109834067-109834008 | LOC552907 |
| 11.4380388 | up | NM_001122661 | chr5:14933810-14933751 | Speer4e |
| 8.3349295 | up | NM_009895 | chr9:107204196-107204255 | Cish |
| 10.9391596 | up |  | chr4:143724133-143724192 |  |
| 8.3586294 | up | NM_019738 | chr7:133767037-133766978 | Nupr1 |
| 3.7753725 | up | NM_023547 | chr6:83074744-83074685 | Ino80b |
| 2.3394072 | up | NM_144835 | chr13:12531103-12531160 | Heatr1 |
| 6.3469831 | up | NM_026925 | chr19:58755296-58755355 | Pnlip |
| 64.9598052 | up | AK004627 | chr2:167653261-167653202 | 1200007C13Rik |
| 8.5570018 | up | NM_007914 | chr2:103106886-103106827 | Ehf |
| 2.4886516 | up | NM_173784 | chr11:32418614-32418673 | Ubtd2 |
| 2.2824844 | up | NM_175183 | chr3:108005234-108005175 | Atxn7l2 |
| 4.2718515 | up | AK081067 | chr9:96620659-96620718 | Gm6328 |
| 2.5895026 | up | NM_201236 | chrX:34971934-34971993 | Rhox4e |
| 3.1296226 | up | NM_001081373 | chr9:45617910-45617851 | Cep164 |
| 4.8492516 | up | NM_026918 | chr7:134193896-134193837 | Zg16 |
| 3.2525066 | up | NM_001282028 | chr2:177670757-177670698 | Gm14326 |
| 2.4088925 | up | NM_175021 | chr7:29185378-29185319 | Samd4b |
| 3.897219 | up | NM_146087 | chr18:61736169-61736228 | Csnk1a1 |
| 15.8762458 | up | NR_026561 | chr9:48262769-48262828 | Gm8884 |
| 2.2014416 | up | AK137372 | chr2:104836598-104836539 | Gm10796 |
| 4.0552482 | up | AY283181 | chr12:111514980-111514921 | Dio3os |
| 6.9929248 | up | NM_027950 | chr8:121970077-121970136 | Osgin1 |
| 2.0381151 | up | NM_010773 | chr18:70740406-70740465 | Mbd2 |
| 4.5568409 | up | BC040217 | chr3:87975777-87975836 | Mef2d |
| 2.2996162 | up | NM_001045542 | chr11:58254964-58255023 | Gm12253 |
| 10.7247269 | up | NM_029639 | chr9:50307294-50307353 | Plet1 |
| 4.1471973 | up | NM_001135115 | chr11:58003608-58003667 | Gm12250 |
| 16.9447964 | up | NM_010861 | chr5:122556732-122556791 | Myl2 |
| 2.6230718 | up | NM_030091 | chr2:72975687-72975628 | Ola1 |
| 2.8934807 | up | NM_178663 | chr2:4721847-4721906 | Bend7 |
| 7.5218237 | up | NM_007483 | chr12:8506364-8506305 | Rhob |
| 11.5317159 | up | NM_001122594 | chr8:112464677-112464736 | Phlpp2 |
| 2.8985472 | up | NM_172051 | chr10:94053581-94053640 | Tmcc3 |
| 5.1109646 | up | NM_001177594 | chr5:120983969-120984028 | Slc8b1 |
| 2.5347738 | up | NM_133905 | chr13:93917594-93917535 | Papd4 |
| 3.5793336 | up | NM_025840 | chr12:36836300-36834179 | Bzw2 |
| 7.813235 | up | NM_198411 | chr12:113853708-113853767 | Inf2 |
| 2.8565841 | up | NM_008876 | chr11:70371548-70371607 | Pld2 |
| 13.3673761 | up | NM_022413 | chr6:41570683-41570624 | Trpv6 |
| 4.5284006 | up | NM_183190 | chr19:11348514-11348455 | Ms4a5 |
| 3.1073574 | up | NM_001039533 | chr16:13834206-13834147 | Pdxdc1 |
| 11.1835747 | up | NM_020581 | chr17:33911939-33911880 | Angptl4 |
| 6.9221154 | up | NM_008714 | chr2:26319499-26319440 | Notch1 |
| 2.915464 | up | NM_174857 | chr19:23377648-23377589 | Mamdc2 |
| 2.0508529 | up |  |  |  |
| 4.0736886 | up | NM_010862 | chr11:60341812-60341871 | Myo15 |
| 2.3113504 | up | NM_146054 | chr14:46079538-46079479 | Fermt2 |
| 3.0187536 | up | NM_146119 | chr2:32780714-32780773 | Fam129b |
| 10.1715829 | up | NR_036630 | chr7:31307152-31307211 | Arhgap33os |
| 9.3643827 | up |  | chr13:012683008-012682949 |  |
| 5.6729592 | up |  | chr4:019465304-019465363 |  |
| 2.423451 | up | NM_001286260 | chr8:109882604-109882663 | Nfat5 |
| 5.8389651 | up | NM_001077495 | chr13:102454465-102454406 | Pik3r1 |
| 13.683538 | up | NM_008842 | chr17:29630740-29630799 | Pim1 |
| 3.973666 | up | NM_011237 | chr19:4195443-4195384 | Rad9a |
| 38.6273419 | up | NM_001271018 | chr11:95961294-95961235 | Calcoco2 |
| 6.1212573 | up | NM_007692 | chr15:89257276-89257068 | Chkb |
| 2.2859201 | up | NM_001285455 | chr11:104189496-104189555 | Mapt |
| 4.9518935 | up | NM_001252684 | chr19:23283283-23283224 | Smc5 |
| 3.6700571 | up | NM_001040111 | chr7:108560937-108560996 | Arap1 |
| 19.2297353 | up |  | chr2:158183277-158183218 |  |
| 2.1376238 | up | NM_026220 | chr2:121319150-121319091 | Mfap1a |
| 3.2415935 | up | NM_001099632 | chr17:37084871-37084930 | Rnf39 |
| 3.6483056 | up | AK048874 | chr17:17973804-17973863 | Spaca6 |
| 4.9843383 | up |  | chr18:044221717-044221776 |  |
| 5.4278758 | up | NM_011310 | chr3:90406396-90406455 | S100a3 |
| 2.7803606 | up | NM_010638 | chr19:23240410-23240469 | Klf9 |
| 5.653049 | up | AK177092 | chr10:80138629-80138570 | Mknk2 |
| 2.3746633 | up |  | chr18:011608071-011608129 |  |
| 2.8580285 | up | NM_001001735 | chr8:26776524-26776583 | Whsc1l1 |
| 2.3680885 | up | AK037820 | chr19:21734966-21735025 | 5033423O07Rik |
| 2.952328 | up | AK037038 | chr5:37062714-37062655 |  |
| 2.3192623 | up | NM_009092 | chr7:88489244-88488675 | Rps17 |
| 2.4453568 | up | NM_010908 | chr7:29544219-29544160 | Nfkbib |
| 2.1963409 | up | NM_178707 | chr7:88187476-88187535 | Zfp592 |
| 2.2295878 | up | NM_175687 | chr9:20678667-20678726 | A230050P20Rik |
| 2.3017179 | up |  | chr13:056534252-056534193 |  |
| 2.552755 | up | AK080922 | chr8:113667277-113667218 | 9430091E24Rik |
| 2.6354549 | up | NM_024495 | chr3:14662332-14662391 | Car13 |
| 7.1056942 | up | NM_001085530 | chr4:41841716-41841775 | Gm13298 |
| 26.4548916 | up | NM_053152 | chr6:129922294-129922235 | Klra22 |
| 2.5623561 | up | NM_001177812 | chr19:46731816-46731875 | Wbp1l |
| 3.2656039 | up | AK087667 | chr2:104552859-104552918 | Gm6306 |
| 50.0608541 | up | NM_133955 | chr8:126187714-126187773 | Rhou |
| 11.9251491 | up |  | chr5:077430589-077430648 |  |
| 3.6361154 | up | NM_001204276 | chr15:97623267-97623208 | Hdac7 |
| 2.7569326 | up | NM_010908 | chr7:29546647-29545252 | Nfkbib |
| 2.2766276 | up | NM_011638 | chr16:32620470-32620529 | Tfrc |
| 15.3720194 | up |  | chrY_random:006930970-006930911 | |
| 3.7163173 | up | AK038263 | chr9:77794642-77794701 | D9Wsu90e |
| 4.0128382 | up | AK163205 | chr11:101879059-101879000 | Mpp3 |
| 2.3068938 | up | NM_001037822 | chr7:149414752-149414700 | Krtap5-5 |
| 2.2300669 | up | NM_001103165 | chr15:102330414-102330473 | Pcbp2 |
| 3.5815257 | up | AK086771 | chr11:115556503-115556444 | D930050J11 |
| 10.3389875 | up |  | chr4_random:000107380-000107321 | |
| 2.4226658 | up | NM_027261 | chr9:15116035-15116094 | Taf1d |
| 2.4550542 | up | NR_033450 | chr12:105491733-105491792 | Serpina3h |
| 4.0401935 | up | NM_010766 | chr1:122371134-122371115 | Marco |
| 3.6337753 | up | AK087773 | chr6:71904893-71904952 | Polr1a |
| 3.3686901 | up | AK054449 | chr3:40601382-40601441 | 4921509J17Rik |
| 4.6475146 | up | AK013590 | chr1:182708974-182709033 | 2900024J01Rik |
| 2.5392362 | up | NM_027840 | chr8:91150813-91150754 | Snx20 |
| 3.0366613 | up | NM_027425 | chr10:62463026-62463085 | Rufy2 |
| 14.5194953 | up | NM_001034893 | chr7:50447417-50447476 | Zfp936 |
| 13.7151006 | up | NM_010824 | chr11:87617840-87617899 | Mpo |
| 2.2558404 | up | NM_133188 | chr10:79750490-79750549 | Dazap1 |
| 7.3277943 | up |  | chr11:112241546-112241487 |  |
| 3.6051418 | up | NM_144530 | chr1:135518234-135518175 | Zc3h11a |
| 3.6131186 | up | NM_173008 | chr7:4896016-4896075 | Ssc5d |
| 2.3076599 | up | AI464956 |  | AI662245 |
| 2.085807 | up | AK145507 | chr17:39985407-39985466 |  |
| 8.6750484 | up |  | chr6:131517946-131517887 |  |
| 4.6441462 | up | NM_001252662 | chr14:71003607-71003548 | Dmtn |
| 2.5704891 | up | NM_007771 | chr10:84594790-84594731 | Cry1 |
| 3.1723288 | up | NM_029004 | chr11:49793657-49793716 | Rasgef1c |
| 8.5636486 | up | NM_009988 | chr16:78359501-78359560 | Cxadr |
| 30.8678394 | up | BY763388 | chr15:98373278-98373219 | Ccnt1 |
| 3.7139585 | up | NM_029815 | chr2:170172707-170172648 | Bcas1 |
| 2.3684513 | up | NR_029468 | chr4:131866864-131866922 | Snhg12 |
| 5.6345875 | up | NM_001252200 | chr1:40081597-40081656 | Map4k4 |
| 2.4533898 | up | NM_144842 | chr14:57413270-57413211 | Zmym5 |
| 4.9444896 | up | NM_053080 | chr7:73535844-73535785 | Aldh1a3 |
| 2.2752313 | up |  | chr13:021573193-021573134 |  |
| 4.3760757 | up | NM_001033207 | chr8:97051105-97051164 | Nlrc5 |
| 2.5357011 | up |  | chr11:080237597-080237538 |  |
| 2.0042577 | up | NM_015800 | chr17:78773595-78773654 | Crim1 |
| 2.0717527 | up |  | chr7:058196082-058196023 |  |
| 2.0995221 | up | NM_021451 | chr18:66625088-66625147 | Pmaip1 |
| 2.0213316 | up | NM_009716 | chr15:80086639-80086698 | Atf4 |
| 3.3147407 | up | NM_138660 | chr11:98695061-98695120 | Casc3 |
| 5.8867583 | up | AK143978 | chr6:97274372-97274313 | Frmd4b |
| 4.4560456 | up | NM_177674 | chr4:154817160-154817219 | 2010015L04Rik |
| 2.166389 | up | XR_168670 | chr7:31074968-31075027 |  |
| 2.0235689 | up | NM_001128597 | chr17:35261700-35261759 | Gpank1 |
| 2.4288194 | up |  | chr2:069604839-069604898 |  |
| 16.2500584 | up | NM_013560 | chr5:136365366-136365425 | Hspb1 |
| 2.5715416 | up | NM_001080118 | chr11:98016615-98016556 | Med1 |
| 16.4754333 | up | NM_018857 | chr17:25885628-25885569 | Msln |
| 3.6084118 | up | NM_001114334 | chr11:86316123-86316064 | Rps6kb1 |
| 5.7720468 | up | NM_033592 | chr18:37999725-37999784 | Pcdhga9 |
| 2.309115 | up | NM_180678 | chr6:55029377-55029436 | Gars |
| 2.9198365 | up | NM_013805 | chr16:18778046-18778105 | Cldn5 |
| 4.619109 | up | NM_029139 | chr8:116179374-116179433 | Syce1l |
| 2.1383315 | up | NM_022418 | chr5:23951343-23951284 | Tmub1 |
| 4.3259278 | up | NM_007464 | chr9:7854468-7854409 | Birc3 |
| 12.0225915 | up | BG099879 | chr4:139952451-139952510 |  |
| 3.9523642 | up | NM_027963 | chr11:67738530-67738471 | Wdr16 |
| 2.978882 | up | NM_011031 | chr11:53945032-53945091 | P4ha2 |
| 6.9769573 | up | AK138152 | chr5:117788895-117788954 | Vsig10 |
| 4.3514584 | up | NM_019840 | chr4:102277888-102277947 | Pde4b |
| 2.05226 | up | NM_011551 | chr11:102167324-102167265 | Ubtf |
| 2.1358303 | up | NM_144945 | chr5:52929298-52929239 | Lgi2 |
| 3.3346238 | up | NM_011652 | chr2:76655906-76655847 | Ttn |
| 6.6733617 | up | NM_010895 | chr11:98188002-98187943 | Neurod2 |
| 6.9608124 | up | NR_045309 | chr15:80521517-80521458 | A430088P11Rik |
| 3.0226522 | up | NM_013767 | chr15:79248389-79248330 | Csnk1e |
| 13.0136495 | up | NR_033388 | chr14:3930499-3930558 | Gm3002 |
| 2.4810796 | up | NM_008739 | chr13:55335377-55340095 | Nsd1 |
| 2.9152912 | up | NM_138604 | chrX:7451900-7451959 | Otud5 |
| 3.6239876 | up |  | chr8:004967873-004967932 |  |
| 3.8650766 | up | BC022182 | chr15:98868488-98868547 | Tuba1c |
| 3.615269 | up | NM_025563 | chr19:46776300-46776359 | 2010012O05Rik |
| 15.9493553 | up | AK029486 | chr2:164018666-164018725 |  |
| 2.6885429 | up | DN174638 | chr13:34873512-34873453 |  |
| 3.6803957 | up | NM_130886 | chr11:119206302-119206361 | Card14 |
| 3.5018935 | up | NM_021362 | chr4:65018429-65018488 | Pappa |
| 6.6781958 | up |  | chrY_random:049490621-049490680 | |
| 3.8854804 | up |  | chr1:181625603-181625544 |  |
| 3.4561815 | up | AK133594 | chr2:69715865-69715806 | Mettl5 |
| 3.3345983 | up | NM_172587 | chr13:64296779-64296720 | Cdc14b |
| 4.1053388 | up | AK017853 | chr12:75033965-75034024 | Hif1a |
| 2.4925129 | up | NM_001164413 | chr6:34973414-34973355 | Cnot4 |
| 2.595868 | up | XM_003084742 | chr7:109661599-109661594 | Gm8556 |
| 11.4371128 | up |  | chr3:016140060-016140001 |  |
| 2.7693425 | up | NM_001010833 | chr17:35996478-35996537 | Mdc1 |
| 2.0185971 | up |  | chr1:072506522-072506581 |  |
| 52.9973897 | up | NM_001256382 | chr15:39513319-39513378 | Rims2 |
| 24.0483249 | up | AK015192 | chr16:70382123-70382064 |  |
| 3.8592 | up | NM_001123327 | chr2:104629903-104629844 | Qser1 |
| 3.2652571 | up | NM_147055 | chr7:111337876-111337817 | Olfr649 |
| 3.8884306 | up | NM_080563 | chr12:26992304-26992245 | Rnf144a |
| 2.2313534 | up | NM_198020 | chr8:87223627-87223686 | Trmt1 |
| 11.6227504 | up | NM_001081328 | chr18:59569946-59570005 | Chsy3 |
| 2.3283469 | up | NM_023647 | chr7:63187048-63186989 | Nipa2 |
| 2.2642747 | up | NM_028079 | chr13:63400842-63400901 | 2010111I01Rik |
| 2.1833721 | up | NM_008341 | chr11:7102442-7102501 | Igfbp1 |
| 2.9089772 | up | NM_178440 | chr11:6406614-6406555 | Myo1g |
| 4.2886512 | up | NM_001025439 | chr3:126547685-126547744 | Camk2d |
| 3.6636483 | up | XM_003688907 | chr13:63403319-63403378 | 2010111I01Rik |
| 6.4681261 | up | NM_133709 | chr7:107183146-107183205 | Chrdl2 |
| 4.0942494 | up | NM_001204156 | chr17:28468681-28468622 | Tead3 |
| 2.1784719 | up | NM_177304 | chr8:48154284-48154343 | Enpp6 |
| 16.4185032 | up | AK137618 | chr11:85532530-85532471 | Gm10372 |
| 2.3092446 | up | NM_007585 | chr9:69339532-69339591 | Anxa2 |
| 15.9528358 | up | NM_080451 | chr3:122782153-122782094 | Synpo2 |
| 2.2527528 | up | AK157679 | chrX:103257457-103257516 | Gm11123 |
| 9.1635969 | up | NM_001285952 | chr4:104689409-104689468 | 1700024P16Rik |
| 2.6560734 | up | NM_181073 | chr12:80182574-80182633 | Plekhh1 |
| 4.4408055 | up | NM_146067 | chr16:11887734-11887675 | Cpped1 |
| 3.1546064 | up | XR_106334 | chr4:144042978-144042919 |  |
| 3.8660878 | up | NM_001242372 | chr3:88684021-88684080 | Gon4l |
| 13.6042885 | up |  | chr13:003359246-003359305 |  |
| 3.3999235 | up | AK090246 | chr5:65544429-65544488 | Klhl5 |
| 4.2010798 | up | AK081211 | chr3:157737434-157737375 | B230334C09Rik |
| 19.0136948 | up | NM_030166 | chr14:32871453-32871512 | Galnt15 |
| 2.5564681 | up | AK017009 | chr9:85643977-85644036 | 4933431K14Rik |
| 2.4922866 | up | NM_177461 | chr15:78967265-78967324 | Micall1 |
| 4.1798275 | up | NM_033373 | chr11:99339452-99339393 | Krt23 |
| 2.1298173 | up | NM_178749 | chr18:43477004-43477063 | Stk32a |
| 3.3200754 | up | NM_133801 | chr17:57142888-57142829 | Gtf2f1 |
| 2.1406899 | up | NM_207695 | chr11:58379905-58379901 | Olfr224 |
| 2.8741732 | up | NM_172501 | chr3:53256411-53256352 | Nhlrc3 |
| 3.3319229 | up | NM_199028 | chr10:43235119-43235178 | Bend3 |
| 2.4730047 | up | NM_011324 | chr6:125294843-125294902 | Scnn1a |
| 6.9478596 | up | AK139147 | chr15:36180465-36180406 | 5430434G16Rik |
| 2.1948988 | up | XM_003946019 | chr18:037894913-037894971 | LOC101056541 |
| 3.0910564 | up | NM_016660 | chr17:27700543-27700602 | Hmga1 |
| 17.7448663 | up | NM_182993 | chr7:52430841-52430900 | Slc17a7 |
| 3.4310592 | up |  | chr7:011301489-011301430 |  |
| 2.0790542 | up | NM_178363 | chr12:86411405-86411464 | Ylpm1 |
| 3.3961267 | up | NM_145940 | chr11:109443745-109443686 | Wipi1 |
| 21.6631885 | up | AK172246 | chr10:70839597-70839656 | Ipmk |
| 2.6437138 | up | NM_019830 | chr7:52232207-52232148 | Prmt1 |
| 3.3940253 | up | NR_045598 | chr6:145200120-145200179 | Gm15706 |
| 2.7738485 | up |  | chr11:088834658-088834717 |  |
| 2.1533562 | up | NM_178208 | chr13:23790282-23790223 | Hist1h4c |
| 2.4226744 | up | NM_080561 | chr5:143752805-143752746 | Rnf216 |
| 3.5899432 | up | NM_028965 | chr11:96629250-96629191 | Snx11 |
| 6.1920246 | up | AK170335 | chr12:21405539-21405480 | F630048H11Rik |
| 2.6500563 | up | AK147351 | chr6:121329912-121329853 | Iqsec3 |
| 2.7813735 | up | NM_024244 | chr10:69999204-70005402 | Fam13c |
| 2.1769949 | up | NM_001163470 | chr5:121821815-121821756 | Trafd1 |
| 3.3106709 | up | NM_011414 | chr2:164179908-164179849 | Slpi |
| 2.832989 | up | NM_080559 | chr4:133683387-133683328 | Sh3bgrl3 |
| 5.3895955 | up | NM_009902 | chr5:135463284-135463342 | Cldn3 |
| 3.7001429 | up | NM_011837 | chr15:75395234-75395175 | Ly6h |
| 4.0045302 | up | NM_153563 | chr3:107424418-107424359 | Strip1 |
| 56.2086009 | up | NM_178227 | chr9:40098873-40098932 | Scn3b |
| 2.0201942 | up | NM_007951 | chr12:81735080-81735021 | Erh |
| 3.7948661 | up | NM_172757 | chr8:90695704-90695763 | Heatr3 |
| 7.3465296 | up | AK165039 | chr5:31874726-31874785 | E130118H10Rik |
| 4.82278 | up | NM_008026 | chr9:32229916-32229857 | Fli1 |
| 3.1702948 | up | AK141707 | chr5:121655179-121655238 |  |
| 5.5225593 | up | NM_001002842 | chr17:33782586-33782645 | Pram1 |
| 2.471125 | up | NM_173001 | chr6:71539171-71539112 | Kdm3a |
| 17.6465959 | up | NM_001286685 | chr3:106536273-106536332 | Lrif1 |
| 4.2049149 | up | AK050379 | chr5:128172201-128172260 | Glt1d1 |
| 2.5840622 | up | NM_181545 | chr11:82815863-82815804 | Slfn8 |
| 6.9220003 | up | NM_001040137 | chr7:127803012-127802953 | Pdzd9 |
| 7.6228136 | up | BX516485 | chr12:80932257-80932198 | Gm3596 |
| 3.3989626 | up | NM_010213 | chr4:124385251-124385310 | Fhl3 |
| 2.0849922 | up | BC083141 | chr12:81735022-81734977 | Erh |
| 3.9899647 | up | NM_146116 | chr2:25077719-25077678 | Tubb4b |
| 3.7078529 | up |  |  |  |
| 3.414148 | up | BC037393 | chr5:151451117-151451058 | N4bp2l2 |
| 11.4958943 | up | NM_011090 | chr7:3860276-3860217 | Lilra6 |
| 4.1363005 | up | NM_139294 | chr6:39557866-39557807 | Braf |
| 17.2723167 | up | NM_026090 | chr9:106472987-106472928 | Iqcf4 |
| 3.6810402 | up | NM_028979 | chr4:96235340-96235281 | Cyp2j9 |
| 4.4468067 | up | NM_001198809 | chr2:10360705-10360764 | Sfmbt2 |
| 3.5018857 | up | AK149769 | chr2:18427359-18427300 |  |
| 3.3722855 | up | NM_180588 | chr14:70948383-70948442 | Reep4 |
| 167.5252558 | up | NM_020013 | chr7:52869364-52869305 | Fgf21 |
| 3.9087995 | up | NM_001167884 | chr19:3806255-3806314 | Suv420h1 |
| 6.0204904 | up | NM_010151 | chr13:78328782-78328723 | Nr2f1 |
| 6.3807256 | up | NM_009450 | chr13:34166145-34166116 | Tubb2a |
| 5.7374115 | up | XR_168453 | chr8:64142533-64142474 | Gm3014 |
| 6.2605526 | up | XR_106168 | chr8:108929697-108929638 | A930006D01Rik |
| 2.1468069 | up | NM_009048 | chr10:17840945-17841004 | Reps1 |
| 2.7157704 | up | NM_177663 | chr3:87743554-87743614 | Isg20l2 |
| 2.025959 | up | NM_008963 | chr2:25322291-25322232 | Ptgds |
| 6.1706352 | up | NM_009896 | chr16:10784010-10783951 | Socs1 |
| 2.1210711 | up | NM_010359 | chr3:107770549-107769290 | Gstm3 |
| 8.2344426 | up | AK079528 | chr11:80281347-80281406 | D230035N22Rik |
| 8.0012017 | up | NR_028314 | chr13:91853281-91853222 | 4833422C13Rik |
| 2.6716555 | up | XM_921685 | chr1:107875986-107875927 | Gm7160 |
| 2.9831892 | up | NM_028887 | chr17:71805028-71804969 | Smchd1 |
| 4.3323893 | up | NM_173051 | chr13:32976102-32975867 | Serpinb1c |
| 4.2849332 | up | NM_016692 | chr19:9946952-9946893 | Incenp |
| 47.7084199 | up | AK019576 | chr16:12629268-12629209 | 4930414F18Rik |
| 2.2333076 | up | NM_001002239 | chr18:75162976-75163035 | Rpl17 |
| 2.5271104 | up | AK037585 | chr6:5227604-5227545 | Pon2 |
| 10.0283615 | up | NR_033455 | chr18:57096929-57096988 | Gm10536 |
| 3.068703 | up | NM_013901 | chr3:90057457-90057516 | Slc39a1 |
| 18.4859221 | up | NM_054080 | chr13:4506887-4506828 | Akr1c20 |
| 73.5127201 | up | NM_014194 | chr6:130168927-130168774 | Klra7 |
| 4.0729342 | up | NM_009046 | chr7:20191832-20191773 | Relb |
| 5.06888 | up |  | chr7:046825799-046825858 |  |
| 2.4469668 | up | NM_001081094 | chr3:145267016-145267075 | Znhit6 |
| 4.6845728 | up |  | chr8:078919365-078919306 |  |
| 6.193457 | up | AK133329 | chr14:70132878-70132936 | 4930480K23Rik |
| 7.9040058 | up | NM_144792 | chr19:32198318-32198259 | Sgms1 |
| 6.2776735 | up |  | chr6:071440095-071440036 |  |
| 5.1573477 | up | NM_023516 | chr6:29225127-29225186 | Hilpda |
| 5.0694422 | up | NM_011113 | chr7:25260569-25260628 | Plaur |
| 3.0871744 | up | NM_011777 | chr6:42308334-42308393 | Zyx |
| 4.1773172 | up | NM_001080929 | chr11:115257352-115257411 | Cdr2l |
| 4.284918 | up | NM_010571 | chr5:138084331-138084272 | Irs3 |
| 11.3151249 | up | AK011855 | chr11:33050579-33050520 | 2610201A13Rik |
| 5.0972814 | up | NM_009045 | chr19:5648070-5648129 | Rela |
| 3.6222183 | up |  | chr14:034241845-034241786 |  |
| 2.6646017 | up | NM_008225 | chr16:36963165-36963224 | Hcls1 |
| 6.9128662 | up | NM_001081291 | chr19:6919178-6919119 | Ccdc88b |
| 10.6879155 | up | DQ372760 | chr6:69581839-69581780 |  |
| 5.0484272 | up | AK145592 | chr17:26181334-26181275 | Gm10204 |
| 6.9781166 | up | NM_177077 | chr6:84953257-84953198 | Exoc6b |
| 3.3881013 | up | NM_031881 | chr18:65372799-65372858 | Nedd4l |
| 2.7044327 | up | NM_001005748 | chr13:43229364-43230593 | Phactr1 |
| 2.6447237 | up | NM_001285927 | chr1:80564631-80564572 | Dock10 |
| 2.8404057 | up |  | chr4:124478770-124478829 |  |
| 26.064607 | up | NR_027907 | chr9:27161617-27161676 | AI414108 |
| 14.6356985 | up | AK080926 | chr14:29321378-29321319 | Gm2670 |
| 2.007844 | up | AK145442 | chr4:87480852-87480793 | Mllt3 |
| 2.1502174 | up | NM_001205173 | chr4:139172650-139172709 | Iffo2 |
| 11.1743848 | up | AK049070 | chr5:65196915-65196974 | C230096K16Rik |
| 2.9468794 | up | NM_026496 | chr15:37293139-37293198 | Grhl2 |
| 3.3819234 | up | AK134090 | chr14:54050372-54050431 | Trav6-3 |
| 2.1934526 | up | NM_145156 | chr19:43738503-43738444 | Slc25a28 |
| 5.9117514 | up | NM_007403 | chr7:147164932-147164873 | Adam8 |
| 32.943723 | up | NM_008051 | chr7:52876318-52876377 | Fut1 |
| 4.8120936 | up | AK012974 | chr13:113205213-113205154 | 2810403G07Rik |
| 5.3735049 | up | NM_001281818 | chr11:61941809-61941868 | Specc1 |
| 2.8135469 | up | NM_030209 | chr8:122576633-122576692 | Crispld2 |
| 5.3553379 | up | NM_001282010 | chr1:37320149-37320208 | Cnga3 |
| 8.4286145 | up | XR_168734 | chr15:25215629-25215570 | 4930445E18Rik |
| 2.5154545 | up | NM_030016 | chr3:116284472-116284413 | Trmt13 |
| 2.229985 | up |  | chr8:053873571-053873513 |  |
| 2.1052565 | up | NM_172371 | chr11:70030546-70030487 | Slc16a13 |
| 39.6708031 | up | NM_001166066 | chr15:4898628-4898687 | Mroh2b |
| 5.1116356 | up |  | chr5:044144450-044144509 |  |
| 5.4762033 | up | NM_015759 | chr13:49372982-49372923 | Fgd3 |
| 4.4032486 | up | NM_176966 | chr4:53662695-53662754 | Fsd1l |
| 2.3350736 | up | NM_030251 | chr6:88785969-88785910 | Abtb1 |
| 12.109438 | up | NM_133234 | chr7:16903403-16903462 | Bbc3 |
| 2.4243509 | up | NM_145536 | chr2:152907403-152907462 | Ccm2l |
| 2.9905296 | up | AK051435 | chr10:87289251-87289192 |  |
| 62.8380715 | up | NM_001039959 | chr19:9074153-9074212 | Ahnak |
| 2.6346319 | up | NM_028453 | chr4:138451357-138451298 | Otud3 |
| 7.6096637 | up | NM_016868 | chr7:17616914-17616855 | Hif3a |
| 21.4226389 | up | NM_001085530 | chr4:41841838-41841897 | Gm13298 |
| 3.5354724 | up |  | chrX:010442385-010442326 |  |
| 8.6773529 | up | NM_178417 | chr11:59274783-59274724 | Zfp867 |
| 2.9888703 | up | NM_001013386 | chr11:83233482-83233541 | Rasl10b |
| 3.2984403 | up | NM_144529 | chr7:130430398-130430339 | Arhgap17 |
| 58.1748629 | up | NM_017373 | chr13:53062637-53062578 | Nfil3 |
| 4.5646239 | up | NM_001033329 | chrX:92254245-92254186 | Arhgef9 |
| 13.0978708 | up |  | chrX:083590281-083590222 |  |
| 2.929864 | up | NM_145148 | chr6:97245912-97245853 | Frmd4b |
| 4.629828 | up | XM_003946168 | chr5:64565429-64565488 |  |
| 3.9940434 | up | NM_001081169 | chr12:113365695-113365754 | Aspg |
| 2.212014 | up |  | chr11:055207831-055207772 |  |
| 2.1459999 | up | NM_001170853 | chr1:175787609-175787550 | Mndal |
| 2.8875521 | up | NM_027186 | chr11:70791245-70791304 | Rpain |
| 3.0984047 | up |  | chr17:072221629-072221570 |  |
| 2.8768819 | up | NM_020559 | chr9:106135855-106135796 | Alas1 |
| 3.1501598 | up | BI648893 | chr6:22135266-22135344 |  |
| 4.6661284 | up | BB161756 | chr3:130421199-130421140 | Image:619641 |
| 5.3226179 | up | NM_011877 | chr12:99916761-99916702 | Ptpn21 |
| 2.7510703 | up | NM_029546 | chr10:77633714-77633655 | Pwp2 |
| 12.7727742 | up | NM_011444 | chr6:143781579-143781520 | Sox5 |
| 4.4910242 | up | AK166687 | chr1:33535971-33535912 | Prim2 |
| 3.159587 | up | NM_194345 | chr14:70984032-70983973 | Fam160b2 |
| 12.0452197 | up |  | chr16:016578466-016578525 |  |
| 7.7636765 | up | NM_026338 | chr9:107834549-107834608 | Actl11 |
| 3.132132 | up | NM_175419 | chr2:158464861-158464920 | Actr5 |
| 3.3230097 | up | NM_177374 | chr12:112922052-112922111 | Trmt61a |
| 5.3080527 | up | AK089321 | chr2:180704048-180704107 | Arfgap1 |
| 16.0547022 | up | NM_007893 | chr17:24580800-24580741 | E4f1 |
| 14.2703182 | up | HQ015080 | chr6:70523164-70523223 |  |
| 25.9233363 | up | NM_018815 | chr6:91035913-91035854 | Nup210 |
| 2.8517905 | up | NM_172729 | chr6:54874066-54874007 | Nod1 |
| 6.7498868 | up | NM_007446 | chr3:113258960-113258901 | Amy1 |
| 3.8132061 | up |  | chr17:022153179-022153238 |  |
| 2.198474 | up | NM_008113 | chr17:26336287-26336228 | Arhgdig |
| 2.662655 | up | NM_001029895 | chr7:137538180-137538121 | Ate1 |
| 5.5327702 | up |  | chr10:076696588-076696529 |  |
| 5.1516765 | up | XR_104972 | chr6:18796209-18796150 | Gm20186 |
| 2.1300451 | up |  |  |  |
| 6.5703034 | up |  | chr13:113636985-113636926 |  |
| 5.919772 | up | NM_182809 | chr7:85447574-85447515 | Ntrk3 |
| 11.3011157 | up | AK082636 | chr17:71206238-71206297 | C230073G13Rik |
| 3.1010962 | up | NM_021564 | chr16:22939736-22939795 | Fetub |
| 12.6048889 | up | NR_037996 | chr1:178764827-178764768 | Hmga2-ps1 |
| 4.090612 | up |  | chr10:039382976-039383035 |  |
| 10.6647852 | up | NM_021791 | chr19:4006386-4006557 | Doc2g |
| 3.6419295 | up | NM_172205 | chr7:31540994-31541053 | Sbsn |
| 2.1860524 | up | NM_008413 | chr19:29387487-29387546 | Jak2 |
| 3.7160605 | up | NM_153126 | chr2:103561496-103561437 | Nat10 |
| 5.1445408 | up | NR_024331 | chr7:134654013-134653954 | 1700008J07Rik |
| 6.7821011 | up | NM_008819 | chr11:59784219-59784160 | Pemt |
| 4.4021723 | up | BC063063 | chr12:23041266-23041320 | 2410018L13Rik |
| 4.3759137 | up | AK032119 | chr5:77079006-77079065 |  |
| 4.9276569 | up | NM_001114085 | chr16:14169548-14169607 | Nde1 |
| 2.6710871 | up | NM_001081379 | chr8:125420007-125419948 | Ankrd11 |
| 3.8157723 | up | AK015362 | chr10:80645727-80645668 |  |
| 6.5469978 | up | NR_102315 | chr5:22063801-22063860 | 6030443J06Rik |
| 10.2970059 | up | NM_029548 | chr11:75722473-75722414 | Rph3al |
| 2.9181681 | up | NM_001038696 | chr3:113309340-113309281 | Rnpc3 |
| 3.8830907 | up | NM_025707 | chr12:66044344-66044285 | Klhl28 |
| 3.8556578 | up | NM_173371 | chr4:149353685-149353626 | H6pd |
| 2.8210789 | up |  | chrX:117426257-117426198 |  |
| 45.4014132 | up | AK082498 | chr3:8733178-8733119 | C230057A21Rik |
| 2.2788082 | up | NM_178408 | chr2:24782493-24782434 | Arrdc1 |
| 19.7578492 | up | NM_153789 | chr13:45503808-45503867 | Mylip |
| 2.1506444 | up | NM_018882 | chr8:97537978-97538037 | Gpr56 |
| 8.5965215 | up | XM_001476076 | chr7:110608879-110608820 | Olfr607 |
| 26.4162853 | up | BE864244 | chr1:138160595-138160654 | AW061096 |
| 2.5779942 | up | NM_009469 | chr5:111213573-111213514 | Ulk1 |
| 2.4551809 | up |  | chr8:086001846-086001787 |  |
| 2.6535689 | up | NM_027468 | chr10:117121930-117121989 | Cpm |
| 2.7031684 | up | NM_175332 | chr11:97488899-97488840 | E130012A19Rik |
| 3.1900654 | up | AK012880 | chr8:19688138-19688197 | LOC101056094 |
| 3.2699684 | up | AK014745 | chr1:89762433-89762492 | 4833421G17Rik |
| 3.4856145 | up | NM_027409 | chrX:50698272-50698213 | Mospd1 |
| 7.5268374 | up | AK045799 | chr4:141518847-141518790 | Fhad1 |
| 2.8665988 | up | EU234054 | chr1:88671947-88672006 |  |
| 2.2261893 | up |  | chr19:009999113-009999054 |  |
| 3.1175442 | up | NM_001271432 | chr2:93259327-93259268 | Cd82 |
| 4.5554135 | up | AK007961 | chr11:90112402-90112461 | Mmd |
| 2.5395771 | up | NM_178688 | chr19:57111731-57111672 | Ablim1 |
| 6.0122025 | up | NM_146745 | chr9:39318427-39318368 | Olfr957 |
| 7.5164774 | up | NM_001004148 | chr11:72055556-72055497 | Slc13a5 |
| 77.4622767 | up |  | chr3:051324995-051325054 |  |
| 2.6232069 | up | NM_007797 | chr13:60997469-60997410 | Ctla2b |
| 39.425414 | up |  | chr3:010813299-010813240 |  |
| 2.3572814 | up | NM_001164081 | chr10:127689823-127689882 | Timeless |
| 5.1473229 | up | NM_008871 | chr5:137537500-137537441 | Serpine1 |
| 5.8866029 | up | NM_001081166 | chr15:84615885-84615826 | Phf21b |
| 2.3265868 | up | NM_030131 | chr1:183096395-183096454 | Cnih4 |
| 2.9101118 | up | NM_001168497 | chr11:69665425-69665484 | Plscr3 |
| 5.055322 | up | AK171787 | chr3:54526555-54529943 |  |
| 2.9267571 | up | NM_010736 | chr6:125256673-125256614 | Ltbr |
| 2.236343 | up |  | chr11:97668183-97668242 | Lasp1 |
| 23.3528917 | up |  | chr6:089126183-089126124 |  |
| 4.317006 | up | NM_153402 | chr4:126032391-126032332 | Ago3 |
| 7.3831015 | up | NM_023326 | chr2:25562947-25563006 | Bmyc |
| 13.1069445 | up | NM_144847 | chr15:75916109-75916050 | Nrbp2 |
| 2.3810881 | up | NM_001164735 | chr5:109983791-109983732 | Crlf2 |
| 19.1858476 | up | NM_001077421 | chr17:24090361-24090302 | Sbpl |
| 4.0548187 | up | AK142040 | chr13:104688192-104688133 | Gm2590 |
| 11.3679168 | up | NM_008102 | chr14:47775407-47775348 | Gch1 |
| 2.1299315 | up | NM_001033306 | chr4:45436231-45436172 | Shb |
| 5.7111347 | up | AK019736 | chr12:92208142-92208083 | 4930544I03Rik |
| 7.1900385 | up |  | chr18:016297281-016297222 |  |
| 4.0567987 | up | NM_013613 | chr2:56960758-56960699 | Nr4a2 |
| 2.1355002 | up | NM_007913 | chr18:35024395-35024454 | Egr1 |
| 4.2754744 | up | NM_001081345 | chr7:80600472-80600413 | Chd2 |
| 4.3569861 | up |  | chr3:034082932-034082991 |  |
| 5.6168217 | up | NM_009890 | chr19:34548779-34548720 | Ch25h |
| 6.8247869 | up | NM_008500 | chr2:35937703-35937644 | Lhx6 |
| 2.0114683 | up | NM_172670 | chr2:92205287-92205228 | Gyltl1b |
| 12.1941433 | up | NM_013749 | chr17:23812805-23812746 | Tnfrsf12a |
| 6.1435519 | up |  | chr19:026605381-026605440 |  |
| 2.0388081 | up | NM_027168 | chr10:31047833-31047892 | Hddc2 |
| 8.7530742 | up | NM_011332 | chr8:97335770-97335829 | Ccl17 |
| 2.490162 | up | W17435 | chr5:30322750-30322691 |  |
| 4.6926287 | up | NM_172442 | chr19:12540994-12540935 | Dtx4 |
| 2.5946154 | up |  | chr16:003633304-003633245 |  |
| 2.6152477 | up | NM_177545 | chr3:101962189-101962130 | Vangl1 |
| 2.4524913 | up | NM_027093 | chr2:139996598-139996657 | Ndufaf5 |
| 16.9566386 | up | NM_170588 | chr2:155904317-155904106 | Cpne1 |
| 5.530485 | up | NM_001038887 | chr5:123105174-123105233 | P2rx7 |
| 2.5625339 | up | AK077051 | chr10:70799536-70799477 | 4932439E07Rik |
| 10.4038673 | up | NM_007413 | chr11:62079834-62079893 | Adora2b |
| 3.1066991 | up | NM_016896 | chr11:103081308-103081249 | Map3k14 |
| 21.1449567 | up |  | chr7:087391613-087391554 |  |
| 2.458588 | up | NM_025671 | chr5:124565426-124565485 | Ogfod2 |
| 5.1292895 | up | NM_001101475 | chr18:60459976-60460035 | F830016B08Rik |
| 5.0073639 | up | NR_033123 | chr4:42472710-42472769 | 4933409K07Rik |
| 4.1334364 | up |  | chr3:093007746-093007687 |  |
| 2.2594623 | up | NM_001197046 | chr17:8389274-8389333 | Fgfr1op |
| 2.0849041 | up |  | chr14:050681094-050681035 |  |
| 2.2866467 | up | NM_013673 | chr1:87605957-87606016 | Sp100 |
| 18.3748198 | up | AF176528 | chr9:109186824-109186765 | Fbxw14 |
| 6.2905118 | up | AK148445 | chr14:27456992-27457051 | AI480461 |
| 7.2574881 | up | NM_030691 | chr7:128207814-128207755 | Igsf6 |
| 7.1298835 | up | NM_146182 | chr7:19979921-19979862 | Klc3 |
| 10.6955777 | up | NM_029843 | chr5:3582209-3584260 | 1700109H08Rik |
| 3.040582 | up | NM_001256198 | chr4:137682429-137682488 | Eif4g3 |
| 4.9020789 | up | AK028644 | chr18:69730353-69730412 | 4732423E21Rik |
| 2.2633746 | up | NR_015463 | chr2:158185110-158185051 | 9430008C03Rik |
| 2.0221233 | up | NM_144825 | chr11:77351192-77351133 | Taok1 |
| 3.9267542 | up | NM_017466 | chr9:110957456-110957397 | Ccrl2 |
| 2.173188 | up | NM_146130 | chr2:75507367-75507426 | Hnrnpa3 |
| 2.0841137 | up | NM_016696 | chr1:94756711-94756770 | Gpc1 |
| 3.5340734 | up | NM_001013366 | chr6:34830619-34830560 | Wdr91 |
| 10.1376946 | up | NM_053117 | chr18:80316044-80316103 | Pard6g |
| 8.6476324 | up | NM_145365 | chr10:80547139-80547080 | Creb3l3 |
| 2.4043918 | up | CD555887 | chr17:22103274-22103215 |  |
| 3.7884119 | up | NM_009999 | chr7:26711522-26711581 | Cyp2b10 |
| 2.1204656 | up | NR_038091 | chr16:24534474-24534533 | A730098P11Rik |
| 3.06004 | up | NR_015553 | chr6:53347149-53347208 | 9430076C15Rik |
| 2.6398043 | up |  | chrX:099334065-099334124 |  |
| 3.6982429 | up | AK012892 | chr4:34802853-34802794 | 2810040C05Rik |
| 2.569408 | up | NR_034074 | chrX:50796614-50796673 | Etd |
| 3.9287174 | up | NM_177568 | chr2:118542825-118542766 | Plcb2 |
| 2.5121498 | up | NM_001163447 | chr17:25034559-25034500 | Mapk8ip3 |
| 2.0665869 | up | AK156477 | chr10:62485460-62485401 | D10Bwg1070e |
| 9.9943033 | up | XR_106391 | chr11:115753436-115753495 |  |
| 3.0917985 | up | NM_133972 | chr8:72744374-72744315 | Armc6 |
| 3.0745187 | up | NM_178762 | chr2:130243617-130243558 | Pced1a |
| 172.9480216 | up | NM_010196 | chr3:82835678-82835737 | Fga |
| 2.1653526 | up | XM_003946373 | chrX:153867506-153867565 | LOC101056611 |
| 2.5122856 | up | NM_001170954 | chr15:83057251-83057192 | A4galt |
| 14.6408387 | up | NM_011088 | chr7:3789816-3789757 | Pira11 |
| 11.3105086 | up | BY706965 | chr2:160410868-160410927 |  |
| 9.9119726 | up | NM_023483 | chr9:50571271-50571212 | 1110032A03Rik |
| 24.3160671 | up | XM_003945407 | chr6:89134673-89134614 | LOC101056056 |
| 13.1626348 | up | NM_080469 | chr4:117322006-117346399 | Eri3 |
| 4.8202167 | up | NM_144791 | chr1:157878565-157877524 | Tor1aip1 |
| 5.600793 | up | NM_182808 | chr6:96607132-96607191 | Fam19a1 |
| 3.4234418 | up | NM_001177589 | chr8:34001060-34001001 | Gm3985 |
| 2.2462399 | up | NM_010276 | chr4:11641692-11641751 | Gem |
| 15.7491712 | up | AK132033 | chrX:140711125-140711066 |  |
| 2.6093654 | up | NM_145378 | chr2:119868539-119868598 | Pla2g4b |
| 2.4873994 | up | NM_030695 | chr3:86585524-86585583 | Lrba |
| 3.0804591 | up | NM_001113530 | chr3:107548580-107548521 | Csf1 |
| 2.4074102 | up | NM_172681 | chr3:83702268-83702209 | D930015E06Rik |
| 4.1767726 | up | AK138828 | chr1:158197214-158197156 | Tdrd5 |
| 2.5269428 | up | NM_010411 | chr18:38096714-38096655 | Hdac3 |
| 3.6077066 | up | NM_030559 | chr2:130269407-130269466 | Vps16 |
| 3.8199348 | up | XR_141750 | chr14:67412515-67412574 |  |
| 3.7537384 | up | NM_177806 | chr12:66162760-66162819 | Prpf39 |
| 2.7686813 | up | NM_025976 | chr16:13703584-13703643 | Bfar |
| 2.8150856 | up | NM_026825 | chr13:24198238-24198179 | Lrrc16a |
| 57.362858 | up | NM_001281852 | chr3:90496632-90496573 | S100a9 |
| 2.446815 | up | BC030318 | chrX:7245128-7245070 | Plp2 |
| 2.1619348 | up | NM_172941 | chr11:59299085-59299026 | Zkscan17 |
| 5.2068451 | up | AK155776 | chr3:133176479-133176421 | Tet2 |
| 34.7693984 | up | NM_001039688 | chrX:34809907-34809966 | Rhox4a |
| 2.5578275 | up | NM_025891 | chr5:24098863-24098592 | Smarcd3 |
| 8.7708308 | up | NR_029382 | chr14:115445828-115445887 | Mir17hg |
| 3.408656 | up | NR_045893 | chr11:6425675-6425616 | Gm11974 |
| 32.736033 | up | NM_019493 | chr9:50927671-50927730 | Btg4 |
| 2.4114443 | up | AK083074 | chr5:66983764-66983705 | C530043K16Rik |
| 2.2637713 | up | NM_145358 | chr5:123187541-123187482 | Camkk2 |
| 4.1066472 | up | NM_023142 | chr5:145888995-145889054 | Arpc1b |
| 3.5180649 | up | NM_001080813 | chr8:28252489-28252430 | Rab11fip1 |
| 2.1031575 | up | NM_001286786 | chr6:93632171-93632112 | Magi1 |
| 2.699711 | up | AK079976 | chr7:127986261-127986202 |  |
| 2.6458257 | up | NM_022887 | chr2:28546610-28546669 | Tsc1 |
| 3.4773529 | up |  | chr18:040845710-040845651 |  |
| 4.5051995 | up |  | chr7:118277000-118276941 |  |
| 3.9331631 | up | U16671 | chr16:66756513-66756572 |  |
| 12.5037939 | up | AK082974 | chr19:32232805-32232746 | Sgms1 |
| 2.0160919 | up | AK013065 | chr1:10056871-10056930 | Cspp1 |
| 2.5125791 | up | NM_001029993 | chr8:124941196-124941255 | Zc3h18 |
| 2.9061525 | up | AK088133 | chr1:84738278-84738219 | C130021H21Rik |
| 3.3790648 | up | NM_172734 | chr6:146725000-146725059 | Stk38l |
| 2.0637955 | up | NM_001039394 | chr6:87738920-87738861 | Rab43 |
| 6.393051 | up | NM_146286 | chr9:39830808-39830867 | Olfr981 |
| 2.3132994 | up | NM_001081240 | chr8:80105178-80105237 | Prmt10 |
| 3.0016983 | up | NM_011508 | chr11:100183303-100183362 | Eif1 |
| 7.6563512 | up | NR_030738 | chr11:62418244-62418303 | 2410006H16Rik |
| 2.6094296 | up | NM_199304 | chr2:154472493-154472552 | Zfp341 |
| 2.9497977 | up | CO807821 | chr7:25074106-25074165 |  |
| 3.1565115 | up | NM_133248 | chr5:107978143-107978084 | Glmn |
| 6.5699027 | up | NM_001029984 | chr1:172837480-172837421 | Fcrlb |
| 2.1237309 | up | NM_013455 | chr15:89404729-89404788 | Acr |
| 9.898233 | up | NM_153579 | chr7:82260247-82260188 | Sv2b |
| 2.3784043 | up | NM_134034 | chr11:29119617-29119676 | Smek2 |
| 4.0980245 | up | NM_153582 | chr8:106875264-106875205 | Cmtm4 |
| 2.5155032 | up | NM_016780 | chr11:104531672-104531731 | Itgb3 |
| 2.8403604 | up | NR_028285 | chr5:149554656-149554715 | 2210417A02Rik |
| 2.7340109 | up |  | chr13:066965420-066965479 |  |
| 4.2203335 | up |  | chr9:054537959-054537900 |  |
| 2.5086114 | up | NM_027418 | chr9:75234779-75234720 | Mapk6 |
| 13.2424945 | up | AK038100 | chr14:73099088-73099029 | A130078K24Rik |
| 8.0208882 | up | NM_027708 | chr5:138053792-138053733 | Fbxo24 |
| 13.1812949 | up | NM_011825 | chr1:176764648-176764589 | Grem2 |
| 2.8249082 | up | NM_023554 | chr13:43497956-43498015 | Nol7 |
| 3.5462912 | up | NM_008808 | chr5:139452993-139452934 | Pdgfa |
| 2.5641515 | up | NM_001110275 | chr16:91871472-91871531 | Itsn1 |
| 3.7615441 | up | NM_016776 | chr11:72264991-72265050 | Mybbp1a |
| 2.2868829 | up | NM_172650 | chr1:190795038-190794979 | Kctd3 |
| 8.8846645 | up | NM_008141 | chr3:107904135-107904194 | Gnat2 |
| 3.5944387 | up |  | chr1:074859686-074859627 |  |
| 3.1153948 | up | NM_011919 | chr8:11562388-11562447 | Ing1 |
| 2.402407 | up | AK013706 | chrX:148749931-148749872 | 2900056M20Rik |
| 4.9241066 | up | AK161160 | chr4:63235046-63235105 | 6330416G13Rik |
| 4.8472846 | up | NM_177687 | chr6:134806119-134806178 | Crebl2 |
| 9.2468744 | up | XR_106371 | chr11:117893684-117893743 | Gm10099 |
| 2.9596139 | up | NM_001099276 | chr1:134997309-134997368 | Pik3c2b |
| 2.7118042 | up | NM_178771 | chr8:72974208-72974149 | Klhl26 |
| 2.8149209 | up | NR_073523 | chr11:82842175-82842116 | Slfn10-ps |
| 3.2984792 | up | NM_025821 | chr16:8660849-8660790 | Carhsp1 |
| 4.3830967 | up | NM_008546 | chr4:140570920-140570979 | Mfap2 |
| 2.2313894 | up |  | chr14:076246069-076246010 |  |
| 2.3549842 | up | NM_026374 | chr3:90291393-90291452 | Ilf2 |
| 4.1532451 | up | NM_027246 | chr10:93046261-93046202 | Snrpf |
| 90.6748784 | up | NM_001172205 | chr1:36377312-36377371 | Arid5a |
| 2.9884305 | up | NR_003270 | chr4:131908064-131908005 | Snhg3 |
| 2.1033574 | up | NM_001042743 | chr4:115979439-115979380 | Mast2 |
| 2.1443652 | up |  | chr8:083405009-083405068 |  |
| 4.1192863 | up |  | chr7:112561238-112561179 |  |
| 39.5518919 | up | NM_009436 | chr16:17900050-17900109 | Tssk2 |
| 15.7630194 | up | NM_001029842 | chr11:109314115-109314056 | Slc16a6 |
| 2.3998736 | up | NM_011599 | chr4:71779230-71779171 | Tle1 |
| 3.7359224 | up |  | chr14:008998183-008998242 |  |
| 26.0022038 | up |  | chrX:089108030-089107971 |  |
| 3.3431405 | up | NM_175560 | chr7:134516130-134516071 | Zfp747 |
| 2.1204258 | up | NM_015789 | chr7:52462975-52462916 | Dkkl1 |
| 3.5202478 | up | AK042341 | chr11:103770799-103770740 | Nsf |
| 4.2602119 | up |  | chr5:122812353-122812412 |  |
| 15.2441098 | up | NM_010345 | chr11:11833224-11833165 | Grb10 |
| 5.7889336 | up | AK082563 | chr5:69687639-69687580 | Kctd8 |
| 2.1366364 | up | NM_001253777 | chr4:41454355-41454296 | 1110017D15Rik |
| 2.2808285 | up | NM_008270 | chr11:96137748-96137807 | Hoxb9 |
| 5.9703065 | up | AK047948 | chr2:73328373-73328314 | C130023C23Rik |
| 6.653515 | up | NM_177201 | chrX:148055442-148055501 | Phf8 |
| 2.2522678 | up | NM_023900 | chr10:80259396-80259337 | Plekhj1 |
| 4.2095249 | up |  | chr4:146817783-146817842 |  |
| 5.2657955 | up | NM_028182 | chr8:70871188-70871247 | Sh2d4a |
| 2.4035803 | up | NM_007465 | chr9:7818747-7818688 | Birc2 |
| 3.5372226 | up | NM_172121 | chr15:75584936-75584877 | Zc3h3 |
| 2.5408778 | up | NM_001004144 | chr11:77321217-77321276 | Git1 |
| 2.3117702 | up | NM_001252200 | chr1:40082906-40082965 | Map4k4 |
| 2.7051254 | up | NM_009530 | chrX:102995666-102995607 | Atrx |
| 9.1988212 | up | AK013795 | chr2:129422894-129422953 | 2900076A13Rik |
| 2.8366694 | up | AK032850 | chr4:148629322-148629263 | 6720462K09Rik |
| 2.7318955 | up | NM_153782 | chr11:109534468-109534409 | Fam20a |
| 2.5545745 | up | NM_001122850 | chr8:129925900-129925959 | Pard3 |
| 5.5537556 | up | AK048087 | chr4:63689777-63689718 | C130033p17rik |
| 3.8926016 | up | NM_024450 | chr19:44318249-44318308 | Scd3 |
| 3.1129821 | up | NR_029432 | chr18:61887845-61887904 | 1500015A07Rik |
| 44.5355467 | up | NM_001039698 | chrX:35061231-35061172 | Rhox4g |
| 23.0315484 | up | DQ648020 | chr7:135832281-135832340 | Inpp5f |
| 4.1537903 | up | NM_010276 | chr4:11642081-11642140 | Gem |
| 2.8014899 | up | NM_032610 | chr7:28141461-28141402 | Sptbn4 |
| 2.9523177 | up | AK040467 | chr3:157689595-157689536 | Srsf11 |
| 3.5566658 | up | NM_178751 | chr5:136624583-136624524 | Orai2 |
| 12.649019 | up | XR_030524 | chr8_random:17727-17668 |  |
| 4.2865679 | up | NM_010439 | chr5:149860479-149860420 | Hmgb1 |
| 4.2272089 | up | NM_001033326 | chr4_random:129714-129773 | Dhrsx |
| 5.5998155 | up | AK139207 | chr4:108295595-108295536 |  |
| 6.3979894 | up | AK041614 | chr15:59976661-59976602 |  |
| 3.5013549 | up | NM_021545 | chr13:101053129-101053070 | Naip7 |
| 2.1163822 | up | NM_001243837 | chr15:4952792-4952733 | C7 |
| 32.5032866 | up | AK155848 | chr6:54795954-54796013 | 2610209C05Rik |
| 8.6869095 | up |  | chr9:3038257-3038316 |  |
| 10.1608402 | up | NM_029982 | chr10:115019404-115019463 | A930009A15Rik |
| 3.9801533 | up | AK045231 | chr6:86625725-86625666 | Snrnp27 |
| 3.2203355 | up | NM_008390 | chr11:53590746-53590805 | Irf1 |
| 2.8141258 | up | XR_140704 | chr5:37556899-37556958 | Gm5107 |
| 22.7811552 | up | NM_001080926 | chr4:107549365-107549424 | Lrp8 |
| 4.0307017 | up | NM_133765 | chr8:124076008-124075949 | Fbxo31 |
| 5.2192663 | up | NM_028810 | chr2:50986054-50985995 | Rnd3 |
| 6.5899791 | up | NM_001081159 | chr13:96247315-96247256 | S100z |
| 11.0961016 | up | NM_009855 | chr16:38486772-38486831 | Cd80 |
| 2.4115909 | up | NM_001164153 | chr10:79876022-79875963 | Tcf3 |
| 5.3040927 | up | NM_174851 | chr4:135263946-135264005 | Ifnlr1 |
| 8.7875074 | up | AK020075 | chr19:60991552-60991611 | 6030458E02Rik |
| 3.0353313 | up | NM_009920 | chr19:5092941-5092882 | Cnih2 |
| 2.1562359 | up | NM_029394 | chr18:53550323-53550382 | Snx24 |
| 5.500459 | up | NM_133206 | chr8:114146918-114146977 | Znrf1 |
| 3.261892 | up |  | chr14:121113011-121113070 |  |
| 3.0054679 | up |  | chr12:024701358-024701299 |  |
| 2.2744309 | up |  | chr16:031258120-031258061 |  |
| 6.6519485 | up | NM_007921 | chr1:137150387-137150328 | Elf3 |
| 3.8482155 | up | NM_001135657 | chr2:90270027-90269968 | Ptprj |
| 2.2620227 | up | NM_011311 | chr3:90409908-90409967 | S100a4 |
| 14.3535431 | up |  | chr5:119436656-119436597 |  |
| 4.9754382 | up | NM_021316 | chr7:148613055-148612996 | Cend1 |
| 13.885724 | up | NM_001033851 | chr15:90479705-90479028 | Cpne8 |
| 3.2668074 | up | NM_027528 | chr1:172259364-172259305 | Nos1ap |
| 2.0289678 | up |  | chr7:090115367-090115308 |  |
| 2.535257 | up | NM_001166364 | chr9:57389574-57389633 | Fam219b |
| 5.8290711 | up |  | chr8:086632314-086632373 |  |
| 2.1758695 | up | NM_001163379 | chr17:34750307-34750366 | Agpat1 |
| 2.4570599 | up | NM_028274 | chr8:113581332-113581391 | Exosc6 |
| 2.1807479 | up | NR_003634 | chr6:148304043-148304102 | Rps4y2 |
| 8.9876815 | up | NM_009883 | chr2:167515828-167515887 | Cebpb |
| 9.1293554 | up | AK135677 | chr4:90026095-90026036 | Gm12633 |
| 2.2672807 | up | NM_133953 | chr8:113334451-113334392 | Sf3b3 |
| 2.6316598 | up | NM_021424 | chr9:43615340-43615399 | Pvrl1 |
| 10.6707354 | up | NM_010751 | chr6:86599112-86599053 | Mxd1 |
| 9.8420197 | up | NM_007666 | chr15:12964040-12963981 | Cdh6 |
| 22.8913462 | up | NM_027728 | chr2:21102467-21102408 | Enkur |
| 3.3151811 | up | NM_146223 | chr9:57449699-57449640 | Cplx3 |
| 2.5425489 | up | NM_175407 | chr10:42723070-42723011 | Sobp |
| 2.3706527 | up |  | chr8:052965492-052965551 |  |
| 2.4193079 | up | AK015812 | chr6:91640040-91639981 | 4930517G19Rik |
| 4.8955061 | up | NM_001110508 | chr8:28091863-28091922 | Zfp703 |
| 2.1162954 | up | NM_001031808 | chr2:24828094-24828035 | Mrpl41 |
| 4.3658211 | up | NM_007791 | chr1:137648747-137648806 | Csrp1 |
| 3.5544664 | up | AK143547 | chr2:7514961-7497726 |  |
| 32.2080631 | up | NR_024720 | chr17:95160893-95161809 | 2700099C18Rik |
| 2.943975 | up | AK048059 | chr2:167017376-167017317 | Ptgis |
| 4.4895673 | up | AK129475 | chr3:58920427-58920486 | Med12l |
| 2.1177452 | up | NM_023755 | chr1:120577637-120577696 | Tfcp2l1 |
| 2.0393435 | up | NM_174991 | chr15:74359994-74360053 | Bai1 |
| 2.3392737 | up | NM_145149 | chr7:29935038-29935219 | Rasgrp4 |
| 2.0574288 | up | XM_001472451 | chr1:78213323-78213264 | Gm2102 |
| 3.5736169 | up | NM_008672 | chr7:150715332-150713167 | Nap1l4 |
| 2.8497888 | up | NM_011400 | chr4:118809864-118809923 | Slc2a1 |
| 2.248388 | up |  | chr14:027097030-027096971 |  |
| 3.08949 | up | NM_001014981 | chr18:63898479-63898538 | Wdr7 |
| 5.266009 | up | AK019465 | chr13:55287219-55287278 | 3930401B19Rik |
| 2.759024 | up | NM_172882 | chr5:102342192-102341192 | Wdfy3 |
| 8.0641915 | up | NM_145133 | chr3:127501092-127501151 | Tifa |
| 2.4148835 | up | NM_009036 | chr2:164240195-164240254 | Rbpjl |
| 3.969399 | up | AK136939 | chr3:116624835-116624776 | Palmd |
| 2.4910259 | up | NM_027249 | chr11:75283308-75283367 | Tlcd2 |
| 2.0763584 | up | NM_009868 | chr8:106668159-106668218 | Cdh5 |
| 5.8874067 | up | NM_172618 | chr17:30650573-30650514 | Btbd9 |
| 2.1276473 | up | NM_173185 | chr9:65890804-65890863 | Csnk1g1 |
| 2.4768675 | up | NM_175560 | chr7:134516212-134516153 | Zfp747 |
| 12.3166107 | up | NR_030709 | chr17:22919079-22919020 | Gm16386 |
| 2.9310529 | up | AK141983 | chr5:48789408-48789467 | 5730480H06Rik |
| 3.1397659 | up |  | chr14:062178241-062178300 |  |
| 2.4975897 | up | NM_011945 | chr13:112536873-112536814 | Map3k1 |
| 5.1391366 | up | XM_990056 | chr12:89327847-89327906 | Gm8587 |
| 2.1780363 | up | NM_021326 | chr5:143934072-143934013 | Rbak |
| 12.234169 | up | AK020250 | chr18:60812487-60812428 | Synpo |
| 4.5557715 | up | NR_040511 | chr2:9804803-9804744 | 9230102O04Rik |
| 2.7817003 | up | NM_008021 | chr6:128324783-128324842 | Foxm1 |
| 17.3231814 | up | NM_029346 | chr18:70400142-70400083 | Dynap |
| 4.5642759 | up |  | chr7:30317242-30317301 |  |
| 5.0870102 | up | NM_183186 | chr12:100433414-100433355 | Foxn3 |
| 15.4979447 | up | NM_144549 | chr15:59487475-59487534 | Trib1 |
| 4.106935 | up | NM_008219 | chr7:110990268-110990209 | Hbb-bh1 |
| 2.786293 | up | NM_133852 | chr2:32163381-32163440 | Golga2 |
| 8.3978033 | up | AK040896 | chr13:67992602-67992543 |  |
| 17.1832985 | up | NM_001011740 | chr11:59427180-59427239 | Olfr225 |
| 2.8453734 | up | NM_009743 | chr2:152606463-152606404 | Bcl2l1 |
| 4.8051464 | up | NM_022424 | chr5:31595318-31595259 | Fndc4 |
| 8.6806165 | up | NM_015738 | chr15:78873826-78873885 | Galr3 |
| 13.7945979 | up | NM_008748 | chr7:149267593-149267534 | Dusp8 |
| 2.1630725 | up | NM_022009 | chr11:60527828-60527769 | Flii |
| 3.0121216 | up | NM_011597 | chr19:24169790-24169731 | Tjp2 |
| 2.6679119 | up | NM_009523 | chr4:136855342-136855401 | Wnt4 |
| 6.0168087 | up | BC052524 | chr8:10903704-10903763 | 4833411C07Rik |
| 2.8972926 | up | NM_053202 | chr6:98889719-98885382 | Foxp1 |
| 5.7177082 | up | NM_177186 | chr4:154995063-154995122 | Slc35e2 |
| 6.3695259 | up | NM_001145978 | chr14:57256794-57260137 | Parp4 |
| 5.1353728 | up | CO044622 | chr9:50501017-50501076 | AI835735 |
| 3.5869807 | up | NM_028315 | chr14:99476564-99476505 | Dis3 |
| 3.5307887 | up | NM_027236 | chr19:5370188-5370247 | Eif1ad |
| 3.5402266 | up | AK143941 | chr8:63768612-63768671 | 1700001D01Rik |
| 2.6249374 | up | NM_177200 | chr6:37933828-37933769 | Svopl |
| 3.1627291 | up | NM_008186 | chr7:54078564-54078623 | Gtf2h1 |
| 10.0256092 | up | NM_028872 | chr1:138110227-138110168 | 5730559C18Rik |
| 4.3616591 | up | XR_168396 | chr2:112112622-112112681 | E330013P08Rik |
| 2.9330751 | up | NM_133985 | chr9:119149843-119149784 | Oxsr1 |
| 3.841526 | up | XM_003946061 | chr1:58932546-58932487 | LOC101056017 |
| 2.3919586 | up | NM_001033634 | chr4:107901280-107901221 | Zyg11b |
| 6.3943202 | up | AK162350 | chr4:133835336-133835277 | Pdik1l |
| 7.8963777 | up | NM_172295 | chr3:101617698-101617639 | Mab21l3 |
| 6.8459464 | up | NM_028669 | chr11:11415024-11415083 | 4930415F15Rik |
| 2.4825728 | up | AK139029 | chr1:24619672-24619613 | ND3 |
| 2.6985689 | up | NM_028000 | chr8:26833012-26833071 | Ppapdc1b |
| 17.9463569 | up | NM_009921 | chr9:109749970-109749911 | Camp |
| 9.7634133 | up | NR_040337 | chr7:148796023-148795965 | Gm16982 |
| 2.6824922 | up | NM_021898 | chrX:80732089-80732030 | Tsga8 |
| 6.7117315 | up | NM_139064 | chr5:34838838-34838779 | Tnip2 |
| 23.4413621 | up | NM_023294 | chr17:71861755-71860793 | Ndc80 |
| 8.4502967 | up | NM_001033229 | chr15:74665389-74665330 | Cyp11b1 |
| 2.3958962 | up | NM_178795 | chr2:121136701-121136642 | Ppip5k1 |
| 7.0031869 | up | NM_001114079 | chr2:163876165-163876224 | Pabpc1l |
| 2.19416 | up | AK015427 | chr3:59643782-59643841 | 4930449A18Rik |
| 2.7810564 | up |  | chr14:011270797-011270738 |  |
| 2.08379 | up | NM_153557 | chr2:25316516-25316457 | BC029214 |
| 11.2379548 | up | NM_029844 | chr16:90749858-90749917 | Mrap |
| 2.222219 | up | NM_146064 | chr15:101993657-101993716 | Soat2 |
| 2.7164623 | up | NM_028026 | chr7:88036225-88036166 | Wdr73 |
| 4.1983196 | up | NM_001167918 | chr4:123080952-123080893 | D830031N03Rik |
| 6.5116944 | up | NM_001081043 | chr9:110287653-110287594 | Ptpn23 |
| 3.0492768 | up | NM_194348 | chr19:6261741-6261800 | Atg2a |
| 2.2903737 | up | NM_013889 | chr4:34752270-34752211 | Zfp292 |
| 3.8933213 | up | NM_028064 | chr15:76442925-76442866 | Slc39a4 |
| 2.9083038 | up |  | chr3:099812522-099812581 |  |
| 25.9427768 | up | NM_001163518 | chr15:75811231-75811172 | Ccdc166 |
| 2.9539471 | up | NM_001081027 | chr1:142506597-142506656 | Kcnt2 |
| 4.6371243 | up | NM_001284381 | chr11:43588206-43588147 | Adra1b |
| 3.0954733 | up | NM_146075 | chr17:27326778-27326719 | Lemd2 |
| 4.7005146 | up | JX120343 | chr12:116532407-116532348 |  |
| 2.4797729 | up | NM_021488 | chr6:113666173-113666114 | Ghrl |
| 2.1561154 | up | NM_027875 | chr10:78047421-78047362 | Syde1 |
| 4.940355 | up |  | chr6:083712520-083712461 |  |
| 2.8413755 | up | AK036632 | chr6:129163525-129163466 | 2310001H17Rik |
| 3.1766838 | up | NM_009745 | chr5:135646972-135647031 | Bcl7b |
| 2.0647202 | up | NM_030141 | chr17:57028105-57028164 | 1700061G19Rik |
| 9.1388713 | up | XM_003085120 | chr17:8094983-8094971 | Gm8457 |
| 2.2573545 | up | NM_009112 | chr3:93368437-93368496 | S100a10 |
| 3.7187919 | up | NM_001113470 | chr10:126434210-126434269 | Ctdsp2 |
| 31.516766 | up |  | chr4:086272678-086272619 |  |
| 3.1206302 | up | NM_001134426 | chr9:103257554-103257495 | Cdv3 |
| 10.9315116 | up | NM_172736 | chr7:4099715-4099774 | Leng8 |
| 4.3956877 | up | AK141303 | chr15:10382438-10382379 | Dnajc21 |
| 2.8484092 | up | NM_028894 | chrX:33902253-33902312 | Lonrf3 |
| 2.0043166 | up | AK172714 | chr9:65399629-65399570 | Plekho2 |
| 3.6770243 | up | AK081446 | chr5:145991441-145991382 | Gm6287 |
| 4.3003116 | up | NM_026596 | chr2:179149723-179149782 | 4930591A17Rik |
| 2.6896039 | up | NM_026814 | chr11:120411418-120411359 | Ppp1r27 |
| 3.4395886 | up | NM_178029 | chr7:134943548-134943607 | Setd1a |
| 22.5583978 | up | NM_019450 | chr2:24081127-24081186 | Il1f6 |
| 2.1675286 | up | NM_026647 | chr4:82448734-82448675 | Zdhhc21 |
| 5.2367132 | up | NM_028035 | chr6:51539004-51539063 | Snx10 |
| 19.0041448 | up | NM_146426 | chr7:114966186-114966127 | Olfr469 |
| 2.7268777 | up | NM_053194 | chr7:148048916-148048975 | Ric8 |
| 2.7260845 | up | AK013968 | chr5:132000880-132000821 | 3110001N23Rik |
| 2.6634164 | up | NM_027898 | chr7:31915205-31915146 | Gramd1a |
| 5.9148107 | up | NM_011050 | chr19:54004105-54004164 | Pdcd4 |
| 2.0821053 | up | BC030682 | chr7:71032482-71032541 | Klf13 |
| 7.0219659 | up | NM_028287 | chr10:33646801-33646742 | Zufsp |
| 4.9763005 | up | AK043138 | chr15:101906341-101906400 | Gm9693 |
| 96.5411964 | up | NM_001170333 | chr6:123093890-123093949 | Clec4a2 |
| 2.5715238 | up |  | chr12:117744959-117744900 |  |
| 3.4032034 | up | NM_172863 | chr3:98233867-98233926 | Zfp697 |
| 3.137499 | up | BC116417 | chr15:77638248-77638189 | Myh9 |
| 6.4260491 | up | NM_153510 | chr5:138263239-138263180 | Pilra |
| 3.8627144 | up | NM_010748 | chr13:13869647-13869706 | Lyst |
| 8.9014539 | up | NM_001144992 | chr1:87950876-87950935 | 2810459M11Rik |
| 2.1983148 | up | NM_027279 | chr1:165927301-165927360 | Mettl18 |
| 3.0335036 | up | NM_175171 | chr13:103532130-103532071 | Mast4 |
| 2.8808417 | up | NM_001085355 | chr17:5126965-5127024 | Arid1b |
| 2.2547952 | up |  | chr12:028271386-028271445 |  |
| 4.756479 | up | BC086784 | chr2:32455925-32455984 | St6galnac4 |
| 5.3832645 | up | AK040674 | chr2:167554186-167554245 | A530013C23Rik |
| 6.0424934 | up |  | chr4:86475903-86475962 |  |
| 2.1938943 | up | NM_145478 | chr15:88695626-88695685 | Pim3 |
| 2.0502271 | up | NM_008608 | chr14:55058775-55058834 | Mmp14 |
| 6.8887442 | up | NM_172510 | chr1:133923606-133923547 | Mfsd4 |
| 2.7518789 | up | BC034366 | chr11:3028892-3028951 | Pisd-ps3 |
| 4.3989158 | up | AK148969 | chrX:99560297-99560238 | Gm14858 |
| 9.1893926 | up | NM_029777 | chr1:82440796-82440855 | Rhbdd1 |
| 3.4867985 | up | AK017987 | chr9:71758427-71758368 | 5830443J22Rik |
| 2.0562019 | up | NM_001285493 | chr16:18566713-18566772 | Gnb1l |
| 3.7399804 | up |  | chr11:97695620-97695679 | Lasp1 |
| 9.3422542 | up | NR_001582 | chr10:43939023-43939082 | Speer5-ps1 |
| 56.3603748 | up | XR_168483 | chr12:19497973-19498032 |  |
| 2.0366163 | up | AK144577 | chr4:99604866-99604925 | BC062258 |
| 4.5086949 | up |  | chr9:074909162-074909103 |  |
| 2.5099334 | up | NM_018803 | chr15:89613026-89612967 | Syt10 |
| 3.2650362 | up | NM_145931 | chr16:11136883-11136824 | Zc3h7a |
| 12.9234504 | up | NM_007470 | chr16:31297265-31297206 | Apod |
| 2.1597405 | up |  | chr4:045563352-045563411 |  |
| 8.7114327 | up |  | chr9:3024224-3024283 |  |
| 4.0088224 | up | NM_001081421 | chr12:81704808-81704866 | Galnt16 |
| 2.0765031 | up | NM_001271898 | chr11:116033275-116033216 | Acox1 |
| 2.4217635 | up | NM_023712 | chr7:133513643-133513584 | Spns1 |
| 7.4083538 | up |  | chr9:3016673-3016732 |  |
| 2.8105257 | up | NM_011764 | chr8:108949531-108949590 | Zfp90 |
| 2.0331646 | up | NM_028054 | chr2:119042724-119042783 | Zfyve19 |
| 2.206584 | up | NM_177055 | chr1:87614834-87614775 | A630001G21Rik |
| 5.8825839 | up | AK149107 | chr10:81843803-81843862 |  |
| 2.8247702 | up | AU015584 | chr2:060358396-060358337 | AU015584 |
| 3.6410335 | up | AK020413 | chr6:47414494-47414553 | 9430013L14Rik |
| 3.8141828 | up | NM_001199940 | chr12:105504738-105504797 | Serpina3i |
| 2.2977582 | up | NM_025456 | chr12:55774541-55774482 | Eapp |
| 2.7771325 | up | AK050067 | chr1:60539456-60539397 | Raph1 |
| 3.281242 | up | BC023958 | chr2:118253890-118253949 | Eif2ak4 |
| 3.1752782 | up | NM_027238 | chr4:82866273-82866215 | Ttc39b |
| 2.6242879 | up | NM_172682 | chr3:85464045-85463986 | Fam160a1 |
| 9.8498244 | up | NM_013829 | chr2:135807797-135807856 | Plcb4 |
| 8.4395127 | up | NM_183088 | chr5:130379240-130379299 | 2410018M08Rik |
| 2.2238462 | up | AK135531 | chr7:133995167-133995108 | Ino80e |
| 2.2256412 | up | CF532098 | chr5:114059554-114059495 | AI429363 |
| 4.8457051 | up | NM_008046 | chr13:115243624-115243565 | Fst |
| 2.2124732 | up | NM_001286726 | chr17:36996236-36996295 | Trim26 |
| 5.5677854 | up | NM_139139 | chr2:118998414-118998355 | Dnajc17 |
| 2.0732678 | up | AK054031 | chr14:28097418-28097477 |  |
| 4.4664546 | up | NM_027210 | chr7:18604511-18604569 | Ceacam13 |
| 14.3350934 | up | NM_175462 | chr2:25773734-25773793 | Kcnt1 |
| 2.1013872 | up | NM_009755 | chr14:70874429-70874370 | Bmp1 |
| 5.2050023 | up | AK138827 | chr11:115898087-115898146 |  |
| 7.6835499 | up | NM_139198 | chr5:101001224-101001165 | Plac8 |
| 3.6441719 | up | NM_013719 | chr2:118300892-118300951 | Eif2ak4 |
| 3.028178 | up | NM_001039522 | chr9:75314032-75314091 | Leo1 |
| 2.4394594 | up | AK156863 | chr7:148624083-148624024 | Lrdd |
| 3.011308 | up | NR_015529 | chr7:150219280-150219221 | R74862 |
| 81.1157241 | up | NM_194336 | chr5:105700115-105700056 | Gbp6 |
| 3.9328468 | up | XM_003945852 | chr10:41772417-41772361 | LOC101056559 |
| 5.473773 | up | NM_001005511 | chr7:53059420-53059479 | Lmtk3 |
| 10.4342739 | up | NM_139147 | chr11:121217551-121217492 | Rab40b |
| 2.3583762 | up |  | chr19:039127090-039127031 |  |
| 2.1302164 | up | NM_008622 | chr5:31443973-31443914 | Mpv17 |
| 2.108994 | up | NM_008592 | chr13:31902423-31902482 | Foxc1 |
| 8.6680772 | up |  | chr10:030519394-030519335 |  |
| 10.5859578 | up | NM_001252661 | chr5:90990259-90990318 | 5830473C10Rik |
| 37.1946496 | up | NM_172845 | chr1:173189952-173190011 | Adamts4 |
| 2.4442328 | up | AK052742 | chr2:144220846-144220905 | Csrp2bp |
| 3.2299676 | up | AK137878 | chr11:119693359-119693418 | A430071A18Rik |
| 8.7230412 | up | NM_001081027 | chr1:142506210-142506269 | Kcnt2 |
| 4.7159591 | up |  | chr15:074817449-074817388 |  |
| 2.0909709 | up | BB759059 | chr10:126726850-126726791 |  |
| 2.1273612 | up | NM_009185 | chr4:114715399-114715458 | Stil |
| 2.940892 | up | NM_203319 | chr5:125894984-125894925 | Dhx37 |
| 9.1922211 | up | XM_003085107 | chr16:45512019-45511960 | Gm17783 |
| 7.3019495 | up |  | chr10:120338885-120338944 |  |
| 3.9842893 | up | NM_013782 | chr7:148340473-148340532 | Ptdss2 |
| 31.1388185 | up | NM_011316 | chr7:53983721-53983662 | Saa4 |
| 3.5343184 | up | NM_021521 | chrX:98492544-98492603 | Med12 |
| 3.5269023 | up | AK016181 | chr7:105806264-105806323 | 4930558N01Rik |
| 2.5892962 | up | NM_177152 | chr10:125452006-125452065 | Lrig3 |
| 2.3789385 | up | AK047590 | chr10:85791319-85791260 | B930095M22Rik |
| 3.4774378 | up | NM_145983 | chr6:126483432-126483373 | Kcna5 |
| 2.8387347 | up | NM_010186 | chr3:96086898-96086839 | Fcgr1 |
| 27.4533576 | up | AK154954 | chr8:96703844-96703903 | Mt1 |
| 4.5180126 | up |  | chr8:035539496-035539555 |  |
| 62.2343852 | up | NM_007707 | chr11:117827539-117827480 | Socs3 |
| 2.7060817 | up | NM_013598 | chr10:99558962-99559021 | Kitl |
| 6.5513036 | up | NM_178364 | chr13:65395751-65396925 | Zfp369 |
| 12.4883243 | up | XR_141315 | chr17:45819600-45819541 | E030047D23Rik |
| 6.0886864 | up | NM_008325 | chr5:109112893-109112952 | Idua |
| 13.5014243 | up | NM_013891 | chr17:27851460-27851401 | Spdef |
| 2.6569057 | up | AK020212 | chr12:120404414-120404355 |  |
| 3.2052187 | up | NM_029332 | chr7:82881198-82881257 | Akap13 |
| 4.3504342 | up | NM_001166631 | chr11:46592056-46592115 | Havcr1 |
| 3.1455548 | up | BC044870 | chr5:152232892-152232951 | Gm3704 |
| 2.4724271 | up | NM_033072 | chr10:126719071-126719012 | Mbd6 |
| 6.6384457 | up | NM_010090 | chr2:127163930-127163989 | Dusp2 |
| 3.0603825 | up | NM_009338 | chr17:13136541-13136482 | Acat2 |
| 3.1287572 | up | NM_001114088 | chr13:55599015-55598956 | Pdlim7 |
| 3.8618858 | up | AK033136 | chr11:85587270-85587211 | Bcas3os2 |
| 2.0432698 | up | NM_009158 | chr5:103341448-103341389 | Mapk10 |
| 32.5168139 | up | NM_011337 | chr11:83461417-83461358 | Ccl3 |
| 2.1373038 | up | NM_008716 | chr17:32257907-32257848 | Notch3 |
| 6.005946 | up | AK052542 | chr1:36622399-36622458 |  |
| 4.2835731 | up | NM_010441 | chr10:119798798-119798739 | Hmga2 |
| 10.3830724 | up | AK017236 | chr6:31058936-31058877 | 5330406M23Rik |
| 3.6314834 | up | NM_001145209 | chr10:21030726-21030785 | Hbs1l |
| 2.1320237 | up | NM_026985 | chr8:3668713-3668772 | 1810033B17Rik |
| 2.7540486 | up | AK139645 | chr7:80830353-80830295 | Gm4971 |
| 3.972126 | up | XM_003945615 | chrX:165807900-165807959 | LOC101055917 |
| 2.0071842 | up | NM_139154 | chr17:26019218-26019159 | Rab40c |
| 2.0894356 | up | NM_133978 | chr9:114666016-114665957 | Cmtm7 |
| 4.8889342 | up |  | chr2:169413025-169412966 |  |
| 3.0067091 | up | XR_168493 | chr12:74909293-74909352 | Gm8075 |
| 43.2836273 | up | XM_003085258 | chrX:100209589-100209648 | Pabpc1l2b-ps |
| 3.3872477 | up | NM_001163502 | chr12:85490398-85490339 | Elmsan1 |
| 7.2845462 | up | BC096660 | chr17:6446695-6446636 | Tmem181b-ps |
| 6.1870586 | up | NM_028127 | chr12:72000812-72000871 | Frmd6 |
| 3.7672806 | up | BC037438 | chr11:79843304-79843363 | BC037438 |
| 2.2493267 | up | AK019690 | chr5:110167984-110167925 | 4930522L14Rik |
| 9.3075838 | up | AK046653 | chr11:105806503-105806562 | Gm9910 |
| 2.5747329 | up | AK044656 | chr10:127950689-127950630 | Esyt1 |
| 2.6116119 | up | NM_146471 | chr11:49094504-49094563 | Olfr1393 |
| 3.216729 | up |  | chr19:018755785-018755726 |  |
| 2.3410561 | up | AK158414 | chr18:67989800-67989859 | Cep192 |
| 2.3032013 | up | NM_145158 | chr17:71601697-71601638 | Emilin2 |
| 2.7251327 | up | NM_198168 | chr19:6227879-6227820 | Ppp2r5b |
| 2.5988836 | up |  |  |  |
| 135.7582441 | up | AK028364 | chr1:173190709-173190768 | Adamts4 |
| 2.2065325 | up | NM_146188 | chr7:35424099-35424040 | Kctd15 |
| 2.2154923 | up |  | chr16:89151489-89151548 |  |
| 3.1254783 | up | NM_016925 | chr8:125792269-125792210 | Fanca |
| 4.8087336 | up |  |  |  |
| 2.6836148 | up | NM_001276448 | chr2:25537069-25537128 | Lcn6 |
| 9.3338825 | up | NM_001100460 | chr12:113918807-113918866 | Zbtb42 |
| 3.846509 | up | NM_008534 | chr1:173518790-173518744 | Ly9 |
| 2.2992638 | up |  | chr9:057685735-057685676 |  |
| 2.8301076 | up | NM_176830 | chr11:100270122-100270063 | Leprel4 |
| 10.4824809 | up |  | chr8:013485704-013485763 |  |
| 5.3578679 | up | XR_168491 | chr12:70775569-70775510 | LOC101055722 |
| 3.6366851 | up | NM_011018 | chr11:50016044-50015985 | Sqstm1 |
| 2.754359 | up | NM_001033543 | chr9:100358198-100358139 | Il20rb |
| 15.1895266 | up | NM_199022 | chr2:125453990-125453931 | Shc4 |
| 2.5112541 | up | NM_018743 | chr8:24284271-24284212 | Agpat6 |
| 2.4324565 | up | NM_183297 | chr10:126962727-126962668 | Nxph4 |
| 4.612742 | up | NM_177115 | chr18:56921519-56921460 | March3 |
| 2.1408843 | up | NM_016695 | chr11:101919128-101919069 | Mpp2 |
| 10.847246 | up | AK051763 | chr7:144574815-144574756 | 9430038I01Rik |
| 9.3895675 | up | AK051401 | chr7:89036972-89036913 | Hdgfrp3 |
| 58.9923755 | up | NM_011747 | chr17:23713036-23712977 | Zfp13 |
| 5.3626496 | up | NM_001161365 | chr12:103589104-103589163 | Rin3 |
| 3.3337181 | up | AK158279 | chr9:64707763-64707822 | Dennd4a |
| 10.0009913 | up |  | chrY_random:026828387-026828328 | |
| 7.3019865 | up | NM_011349 | chr9:107583892-107583833 | Sema3f |
| 2.3049828 | up |  | chr19:008972133-008972192 |  |
| 10.6593998 | up | NM_011486 | chr11:100760005-100759946 | Stat3 |
| 2.731422 | up | AK154175 | chr11:11661344-11661403 | Ikzf1 |
| 3.6502721 | up | NM_175833 | chr9:103258730-103258671 | Cdv3 |
| 2.5579814 | up | NM_001142642 | chr5:110792092-110792033 | Fbrsl1 |
| 2.1891975 | up | NM_177673 | chr4:153380301-153380360 | Cep104 |
| 2.3866427 | up | NM_010508 | chr16:91506635-91506693 | Ifnar1 |
| 3.5339264 | up |  | chrY_random:022013721-022013780 | |
| 4.226591 | up | NM_011602 | chr4:43557260-43557201 | Tln1 |
| 2.2932162 | up | NM_133816 | chr1:91048945-91049742 | Sh3bp4 |
| 15.7521189 | up | NM_024245 | chr9:61767732-61767492 | Kif23 |
| 2.3953608 | up | NM_153588 | chr16:13417529-13417588 | Mkl2 |
| 4.8459271 | up | NM_001085492 | chr4:149882952-149883011 | Rere |
| 2.8653493 | up | AK030385 | chr14:99579566-99579625 | Pibf1 |
| 5.4847391 | up | NM_013784 | chr1:107418369-107418310 | Pign |
| 2.8062178 | up |  | chr9:44087921-44087862 | Abcg4 |
| 2.212664 | up | XR_140571 | chr3:95111727-95111668 |  |
| 8.4542513 | up | AK172117 | chr2:98507154-98507096 |  |
| 3.6348278 | up | NM_025404 | chr11:101529032-101529091 | Arl4d |
| 2.9378302 | up | NM_019390 | chr3:88287321-88287262 | Lmna |
| 63.1492196 | up | NM_181319 | chrX:104881102-104881161 | Tbx22 |
| 3.3007732 | up | NM_029362 | chr2:154519942-154520001 | Chmp4b |
| 2.5686085 | up | AK011603 | chr11:85627507-85627566 | Bcas3 |
| 30.7177662 | up | NM_001100454 | chr17:26014694-26014635 | Wfikkn1 |
| 2.0891202 | up |  | chr12:106568134-106568193 |  |
| 11.8026892 | up | NM_183426 | chr10:79520318-79520259 | Sbno2 |
| 3.08848 | up | NM_001025606 | chr13:99456414-99456355 | Tmem171 |
| 7.721601 | up | NM_001199275 | chr11:54724350-54724291 | Tnip1 |
| 2.13831 | up | NM_001282006 | chr11:72158373-72158314 | Tekt1 |
| 5.086786 | up | NM_029608 | chr2:172299505-172299564 | Fam209 |
| 2.0234834 | up | NM_007462 | chr18:34432157-34436232 | Apc |
| 11.467504 | up | NM_027158 | chr5:136540125-136540184 | Upk3bl |
| 2.0315685 | up | NM_207707 | chr12:77221485-77221426 | Esr2 |
| 6.6100418 | up | NR_040470 | chr17:34042803-34042862 | BC033916 |
| 3.6223526 | up | BC028266 | chr13:9689645-9689586 | Zmynd11 |
| 2.491427 | up | NM_001253894 | chr9:21308989-21309048 | Dnm2 |
| 2.3647669 | up |  | chr6:035882916-035882975 |  |
| 4.9274765 | up | NM_028783 | chr9:37221547-37221606 | Robo4 |
| 2.3446551 | up | NM_008700 | chr17:26975680-26975621 | Nkx2-5 |
| 4.150514 | up | NM_001145953 | chr14:48005414-48005473 | Lgals3 |
| 4.1348153 | up | NM_027310 | chr4:124780215-124780274 | Meaf6 |
| 3.7169905 | up |  | chr11:072064277-072064218 |  |
| 14.2289234 | up | NM_001037247 | chr2:152438270-152438329 | Defb36 |
| 2.0082794 | up |  | chr5:140439511-140439452 |  |
| 2.5820519 | up | U62523 | chr7:147232915-147232855 | Msx3 |
| 2.1514242 | up | NM_001164689 | chr2:175881836-175881895 | Gm6710 |
| 3.0450472 | up | AK082718 | chr17:35377173-35377114 | LOC545198 |
| 4.732682 | up | NM_001177792 | chr2:120425750-120425809 | Snap23 |
| 13.2815535 | up | NM_181680 | chr18:20442142-20442201 | Dsg1c |
| 4.8891389 | up | AK048857 | chr3:137806419-137806360 | C230076A16Rik |
| 2.0659862 | up | NM_001038700 | chr2:30891717-30891658 | Fnbp1 |
| 2.5762138 | up | NM_001205336 | chr18:38133176-38133117 | Arap3 |
| 6.8569975 | up | XM_001480563 | chr12:22470101-22470042 | Gm4425 |
| 4.0258488 | up | NM_153423 | chr4:132754009-132754068 | Wasf2 |
| 4.0027716 | up | AW258355 | chr7:107617166-107617225 | AA960618 |
| 3.6698752 | up | NM_172284 | chr8:113527175-113527116 | Ddx19b |
| 4.0740509 | up |  | chr3:084388864-084388923 |  |
| 6.2502721 | up | NM_001081321 | chr5:66006558-66006499 | Pds5a |
| 11.2173213 | up | NM_001163359 | chr11:11700646-11700587 | Fignl1 |
| 2.5118972 | up | AK139379 | chr9:45764023-45764081 |  |
| 9.9621249 | up | KC860259 | chr6:31116558-31116499 |  |
| 3.6774739 | up | NM_016981 | chr4:132976474-132976533 | Slc9a1 |
| 15.9472637 | up | NM_026646 | chr7:148615710-148615651 | Slc25a22 |
| 4.0188076 | up | NM_020275 | chr14:70184141-70184200 | Tnfrsf10b |
| 3.0303369 | up | NM_173382 | chr3:53277418-53277477 | Proser1 |
| 5.7067383 | up | NM_007738 | chr9:108886861-108886920 | Col7a1 |
| 4.6767856 | up | NR_027637 | chr10:121567496-121567555 | Gm4489 |
| 2.9352716 | up | AK172566 | chr2:129753866-129753807 | Gm10183 |
| 2.1556268 | up | AK048336 | chr14:22339433-22339492 | Kat6b |
| 2.5722135 | up | NM_146833 | chr17:37473601-37473542 | Olfr103 |
| 2.3136034 | up |  | chr12:112922195-112922254 |  |
| 2.0501053 | up | NM_134123 | chr17:3198605-3198664 | Scaf8 |
| 5.4482013 | up | NM_026153 | chr15:31254388-31254329 | Ankrd33b |
| 7.1824647 | up | NM_010220 | chr17:28536119-28536060 | Fkbp5 |
| 2.7368474 | up | NM_009061 | chr1:145848889-145848830 | Rgs2 |
| 2.9284629 | up | NM_010454 | chr6:52158253-52158194 | Hoxa6 |
| 3.537111 | up | NM_178793 | chr18:66450856-66450797 | Ccbe1 |
| 2.1823462 | up |  | chr4:069353560-069353619 |  |
| 4.7340436 | up | NM_019690 | chr2:174163501-174163560 | Gnas |
| 3.5243019 | up | NM_027193 | chr3:115631895-115631954 | Dph5 |
| 2.5184945 | up | NM_008239 | chr13:31652206-31652265 | Foxq1 |
| 2.4561936 | up | NM_010206 | chr8:26684061-26684120 | Fgfr1 |
| 10.3958225 | up | NM_153788 | chr11:69695215-69695156 | Acap1 |
| 3.3218149 | up | AF188007 | chr15:76063452-76063393 | Parp10 |
| 2.4923661 | up | NM_027895 | chr5:044819058-044819117 | Ulk3 |
| 3.0015777 | up | NM_022022 | chr4:148772765-148761325 | Ube4b |
| 9.1893524 | up | AK018037 | chr6:149131595-149131536 | Amn1 |
| 22.8526727 | up | NM_194063 | chrX:34798883-34798942 | Rhox3a |
| 4.7851883 | up | NM_018761 | chr4:56854493-56854434 | Ctnnal1 |
| 6.2870795 | up | NM_001037914 | chr13:113790543-113790602 | Mcidas |
| 5.6778628 | up | NM_175539 | chrX:41719518-41719459 | Dcaf12l2 |
| 2.5251099 | up | NM_009062 | chr1:171671668-171671609 | Rgs4 |
| 5.3331001 | up | NM_008317 | chr9:107481893-107481952 | Hyal1 |
| 4.0172298 | up | NM_177318 | chr9:21859963-21859904 | Zfp653 |
| 2.2791905 | up | NM_178923 | chr16:90229694-90229636 | Scaf4 |
| 2.8654148 | up | AK165743 | chr10:80258146-80258205 | Dot1l |
| 2.2906515 | up | NM_001085508 | chr4:43705459-43705518 | Tmem8b |
| 2.7296321 | up | NM_172280 | chr7:105739184-105739125 | 2210018M11Rik |
| 55.7360401 | up | NM_001044384 | chrX:20451802-20451861 | Timp1 |
| 119.4792271 | up | AK036325 | chr10:85586627-85586568 | Syn3 |
| 3.2723317 | up | NM_022015 | chr17:47625332-47625273 | Taf8 |
| 2.1387953 | up | AK145307 | chr6:50796920-50796861 |  |
| 3.0924479 | up | NM_016791 | chr18:80845498-80845439 | Nfatc1 |
| 2.3465132 | up | NM_019777 | chr1:133151868-133151809 | Ikbke |
| 2.5269829 | up | AK083799 | chr4:24667649-24667590 |  |
| 7.335082 | up |  | chr9:100445880-100445939 |  |
| 3.6589551 | up |  | chr2:114679709-114679650 |  |
| 2.4659068 | up | NM_153744 | chr1:74787273-74787214 | Prkag3 |
| 2.4801803 | up | NM_172652 | chr1:36392656-36392597 | Kansl3 |
| 2.0058007 | up | AK089751 | chr1:142010417-142010358 | F830014O18Rik |
| 26.6008917 | up | NM_053247 | chr7:117994230-117994171 | Lyve1 |
| 11.7675861 | up | AK086046 | chr10:128282471-128282530 |  |
| 2.8970629 | up | X56569 | chr14:17651483-17651424 | Rarb |
| 3.0405761 | up |  | chr1:108336736-108336795 |  |
| 2.2435271 | up | NM_197988 | chr8:123132584-123132525 | 1190005I06Rik |
| 6.6659399 | up | NM_032002 | chr9:55084303-55075745 | Nrg4 |
| 3.8152828 | up | NM_172677 | chr3:16105369-16113955 | Ythdf3 |
| 2.1317452 | up | NM_009970 | chr19:61300395-61300336 | Csf2ra |
| 6.7242483 | up | NM_007706 | chr10:94875246-94875187 | Socs2 |
| 5.9320033 | up | NM_007880 | chr10:79417377-79417436 | Arid3a |
| 5.4621025 | up | AK076305 | chr15:99900789-99900848 | 4731420N21 |
| 4.8731006 | up | NM_001252587 | chr2:165652866-165652807 | Zmynd8 |
| 2.0173503 | up | Z86033 | chr14:53364436-53364495 |  |
| 6.3792835 | up | NM_007781 | chr15:78113185-78113126 | Csf2rb2 |
| 6.3371242 | up | NM_009500 | chr2:27119268-27119209 | Vav2 |
| 16.2004614 | up |  | chr12:096310121-096310180 |  |
| 2.0389101 | up |  | chr7:026030584-026030525 |  |
| 56.5576629 | up | NM_001039198 | chr14:55680573-55680514 | Zfhx2 |
| 2.2233715 | up | NM_009284 | chr10:127083983-127084042 | Stat6 |
| 2.9484638 | up | AK159005 | chr1:38073544-38073603 | B230213L16Rik |
| 7.4036906 | up | NR_044989 | chrX:33944551-33944610 | Gm6268 |
| 2.5790315 | up | NR_073362 | chr14:42674030-42673971 | 1700091H14Rik |
| 2.294552 | up | NM_001033238 | chr16:52207889-52207948 | Cblb |
| 5.1012761 | up | AK034303 | chr8:96953788-96953729 | 9330175E14Rik |
| 5.5731022 | up |  | chr9:061857710-061857651 |  |
| 2.0240351 | up | AK038819 | chr7:96781607-96781548 |  |
| 2.0274893 | up | NM_001270431 | chr4:41813313-41813372 | Gm20878 |
| 5.351846 | up | NM_019676 | chr9:118980797-118980738 | Plcd1 |
| 2.0993397 | up | XM_003946385 |  | LOC101055644 |
| 2.7025612 | up | XM_003946322 | chr4:151237354-151237413 | 9230110K08Rik |
| 2.3096144 | up | NM_146256 | chr4:116492595-116492536 | Hpdl |
| 2.7389665 | up | NM_026054 | chr6:149279954-149280013 | 2810474O19Rik |
| 2.2757018 | up | AU258065 | chr1:71626146-71626205 | Atic |
| 2.8856073 | up | NM_001001982 | chr2:118579954-118579895 | A430105I19Rik |
| 6.1575089 | up | NM_010728 | chr18:52676971-52676912 | Lox |
| 10.8454138 | up | AK042233 | chr2:90378493-90378434 | A630073K07Rik |
| 6.6840216 | up | NM_010197 | chr18:39018333-39018274 | Fgf1 |
| 2.7985432 | up | NM_018887 | chr17:43887735-43887794 | Cyp39a1 |
| 5.1280382 | up | AK020596 | chr5:117568673-117568732 |  |
| 5.3345885 | up |  | chr14:099671720-099671661 |  |
| 2.7147531 | up | XM_003945589 | chr19:8107651-8107710 | LOC101055780 |
| 2.3014132 | up |  | chr15:045809647-045809588 |  |
| 2.0860713 | up |  | chr7:080830108-080830049 |  |
| 4.703634 | up | NM_175296 | chr1:168131642-168131583 | Mael |
| 3.9573361 | up | BX511707 | chr4:19947177-19947118 | AA416453 |
| 3.3478772 | up | NM_172054 | chr1:38040771-38040712 | Txndc9 |
| 8.2805064 | up | NM_145923 | chr5:64300986-64300927 | Rell1 |
| 2.5523134 | up | NM_020006 | chr11:113588276-113588217 | Cdc42ep4 |
| 2.7825014 | up | AK038793 | chr6:85282334-85282275 | Sfxn5 |
| 3.5310041 | up | AK143992 | chr1:20596645-20596586 | Pkhd1 |
| 3.8702973 | up | AK010974 | chr7:140121166-140121107 | 2510016G02Rik |
| 4.557539 | up |  | chr9:005907352-005907411 |  |
| 2.859319 | up | NM_153502 | chr1:36587464-36587405 | Ankrd23 |
| 2.0306773 | up | NM_029688 | chr2:151937009-151937068 | Srxn1 |
| 2.8728043 | up |  | chr4:099318278-099318337 |  |
| 22.4167853 | up | NM_008501 | chr11:4172032-4172091 | Lif |
| 2.7116651 | up | NM_177806 | chr12:66164232-66164291 | Prpf39 |
| 6.4316629 | up | NR_030688 | chr17:30531974-30531915 | Gm6402 |
| 3.74037 | up |  | chr7:024848921-024848980 |  |
| 7.4384071 | up | NM_007709 | chrX:99442835-99442776 | Cited1 |
| 2.7093241 | up | XR_001564 | chr19:9291936-9291995 | Gm6252 |
| 29.5699357 | up | AK090034 | chr14:43967907-43967966 | Gm3161 |
| 2.0188952 | up | AK019187 | chr14:9201000-9201058 | 2610318M16Rik |
| 5.3228725 | up | NM_177271 | chr10:9347580-9347521 | Samd5 |
| 2.7743023 | up | NM_181594 | chr8:108416994-108417053 | Edc4 |
| 93.8660145 | up | NM_020568 | chr17:56240114-56240055 | Plin4 |
| 3.1650581 | up | NM_001286040 | chr17:28199523-28199582 | Anks1 |
| 4.4994847 | up | NM_028589 | chr11:84986757-84986816 | 1700125H20Rik |
| 10.9029278 | up | NM_023048 | chr6:5382886-5382945 | Asb4 |
| 2.1296239 | up | NM_011375 | chr6:72104404-72104463 | St3gal5 |
| 3.2670375 | up | NM_001199275 | chr11:54724410-54724351 | Tnip1 |
| 2.0276744 | up | BC043039 | chr11:98662234-98662293 | Msl1 |
| 3.1146866 | up | NM_013742 | chr7:150743674-150743615 | Cars |
| 4.1776001 | up |  | chr7:112870254-112870313 |  |
| 3.3020981 | up | AK081287 | chr11:23637110-23637051 | C030046G05 |
| 4.755194 | up | AK090199 | chr15:77753720-77753661 | Txn2 |
| 3.3592256 | up | AK039773 | chr5:121974121-121974180 | Tmem116 |
| 2.1857992 | up | NM_178751 | chr5:136623442-136623383 | Orai2 |
| 3.930098 | up | NM_172161 | chr6:113643695-113643754 | Irak2 |
| 3.311358 | up | NM_001077694 | chr6:84160977-84161036 | Dysf |
| 2.2268082 | up | NM_021478 | chr17:28490738-28490679 | Tulp1 |
| 2.4138198 | up | NM_001038627 | chr5:145641546-145640319 | Smurf1 |
| 4.3584858 | up | NM_019515 | chr5:76769847-76769788 | Nmu |
| 3.4643063 | up | NM_015796 | chr7:29523092-29523151 | Fbxo17 |
| 11.354427 | up | AK077557 | chr16:27439696-27439755 | Ccdc50 |
| 4.0817024 | up | NM_177662 | chr3:81760551-81760610 | Ctso |
| 3.1613424 | up | AK040992 | chr6:21803053-21802994 | Gm3289 |
| 2.2046169 | up | NM_001003920 | chr7:4667524-4667583 | Brsk1 |
| 2.5451213 | up | AK052594 | chr3:132796656-132796597 | Arhgef38 |
| 16.1776764 | up | AI851140 | chr6:23202298-23202239 | C130093G08Rik |
| 4.7491075 | up | NM_172424 | chr5:119207100-119207158 | Med13l |
| 4.9571678 | up | NM_001146080 | chr4:41616815-41616756 | Cntfr |
| 2.0669106 | up |  | chr13:49326118-49326059 | Susd3 |
| 2.0936619 | up |  | chr9:057182548-057182607 |  |
| 2.5778605 | up | NM_027434 | chr2:157869169-157869228 | Rprd1b |
| 7.2769586 | up | XM_619114 | chr13:65517697-65517756 | Gm5792 |
| 2.826099 | up | AK046316 | chr1:180243520-180243461 | B230369F24Rik |
| 2.4082098 | up | NM_011889 | chr15:82122428-82122487 | Sept3 |
| 5.2871765 | up | NM_009397 | chr10:18720835-18720776 | Tnfaip3 |
| 3.911585 | up | NM_001162957 | chr10:33635445-33635504 | Rsph4a |
| 2.0847617 | up | NM_025804 | chr8:125927610-125927669 | Tcf25 |
| 3.2588659 | up | NM_029101 | chr15:82946338-82946279 | Rrp7a |
| 3.4287069 | up | AK164017 | chrX:136391021-136390962 | Morc4 |
| 6.4794583 | up | NM_010688 | chr11:97686173-97686232 | Lasp1 |
| 2.0644479 | up | NM_001038587 | chr3:89557158-89557217 | Adar |
| 8.8050976 | up | NM_027687 | chr18:12913592-12913651 | Cabyr |
| 2.2614169 | up | NM_016668 | chr13:94387040-94386981 | Bhmt |
| 30.2459485 | up | NM_008109 | chr2:155766860-155766801 | Gdf5 |
| 2.0313247 | up |  | chr3:026220966-026221025 |  |
| 2.2726334 | up | NM_029748 | chr4:129284913-129284972 | Tmem234 |
| 3.369375 | up | AK040105 | chr4:136892439-136892380 | A430061O12Rik |
| 3.3540272 | up | AK005351 | chrX:20509038-20508979 | 1500032P08Rik |
| 3.5368867 | up | AK170517 | chr16:20099659-20099718 |  |
| 3.2912066 | up | NM_001025572 | chr17:66399239-66399180 | Ankrd12 |
| 2.3346329 | up | NM_009545 | chr11:97551219-97551160 | Pcgf2 |
| 5.3808124 | up | AK032756 | chr17:12944784-12944843 | Airn |
| 5.5410101 | up | NM_133992 | chr10:127758288-127758347 | Pan2 |
| 2.2888866 | up |  | chr17:034103909-034103968 |  |
| 9.2787663 | up | NM_009621 | chr16:85795103-85795044 | Adamts1 |
| 4.0069791 | up | NM_025881 | chr17:26417299-26417358 | Luc7l |
| 6.8161638 | up | NM_146315 | chr4:118339012-118339071 | Olfr62 |
| 2.9012844 | up | NM_001081008 | chrX:98738534-98738593 | Taf1 |
| 2.097482 | up | NM_172297 | chr7:16861959-16861900 | Ccdc9 |
| 3.6577234 | up | NM_001277183 | chr7:113744877-113744818 | Gm1966 |
| 2.0298864 | up | NM_027419 | chr11:69710919-69710860 | 2810408A11Rik |
| 3.1309902 | up | NM_001081962 | chr5:138083973-138084032 | Sap25 |
| 2.7224365 | up | NM_013659 | chr7:87371351-87371410 | Sema4b |
| 3.7889313 | up | NM_009667 | chr7:117954980-117955039 | Ampd3 |
| 11.31391 | up | NM_008033 | chr8:27109337-27109278 | Fnta |
| 2.0738147 | up | NM_010065 | chr2:32164054-32163995 | Dnm1 |
| 8.1139421 | up | XM_003945668 | chr3:21974670-21974611 | 2810416G20Rik |
| 3.056521 | up |  | chr14:103410097-103410156 |  |
| 6.3207484 | up | AI595487 | chr10:107096455-107096514 | AA060545 |
| 19.0790218 | up | NR_004413 | chr3:96177965-96178024 | Rnu1b6 |
| 3.1300355 | up | NM_001161665 | chr1:180862723-180862782 | Kif26b |
| 3.394961 | up | NM_009878 | chr9:21092993-21092934 | Cdkn2d |
| 5.2626137 | up | AK083336 | chr7:104800757-104800816 | C920008N22Rik |
| 20.9268564 | up | NM_009317 | chr4:53799664-53799723 | Tal2 |
| 5.8664783 | up | NM_177898 | chr8:23184327-23184268 | Nek5 |
| 4.8683504 | up | NM_013925 | chr8:114492365-114492306 | Adat1 |
| 22.7451808 | up | NM_008005 | chr11:33017533-33017474 | Fgf18 |
| 2.8700495 | up | NM_001081336 | chr14:78969475-78969416 | Dgkh |
| 4.7245083 | up |  | chr8:080047872-080047931 |  |
| 7.2852476 | up |  | chr12:111957820-111957761 |  |
| 3.3737522 | up |  | chrY:000902455-000902396 |  |
| 2.1533353 | up | NM_001081434 | chr11:96930152-96930211 | Osbpl7 |
| 2.8955873 | up | NM_007705 | chr10:79634274-79634333 | Cirbp |
| 2.468901 | up | NM_008329 | chr1:175677706-175677647 | Ifi204 |
| 2.4411342 | up | NM_011758 | chr11:58702763-58702704 | Zfp39 |
| 11.7167699 | up | AK139323 | chr12:84193105-84193164 | Rgs6 |
| 3.0597961 | up | AK082316 | chr6:5041673-5041732 | C230037E05Rik |
| 5.3900363 | up | NM_172475 | chr2:4450849-4450908 | Frmd4a |
| 2.5569022 | up | NM_019983 | chr5:130690147-130690206 | Rabgef1 |
| 4.2001417 | up | NM_001114088 | chr13:55599836-55599777 | Pdlim7 |
| 5.6577366 | up | NM_146084 | chr18:34603614-34603555 | Fam13b |
| 2.1356283 | up | NM_001159583 | chr4:41466354-41466295 | Fam219a |
| 4.2909607 | up |  | chr7:028915308-028915367 |  |
| 2.044378 | up | NM_001242388 | chr7:49864966-49864907 | 9830147E19Rik |
| 27.9945212 | up | NM_030708 | chr3:5413028-5413087 | Zfhx4 |
| 6.5607252 | up | NR_001579 | chr3:96218756-96218697 | Terc |
| 3.4828395 | up | NM_016895 | chr4:128687940-128687999 | Ak2 |
| 2.6100022 | up | NM_024452 | chr4:136105174-136105233 | Luzp1 |
| 2.1990387 | up | AK015009 | chr2:120985051-120985110 |  |
| 2.4632661 | up | NM_019511 | chr11:6577416-6577475 | Ramp3 |
| 2.973267 | up | NM_010729 | chr9:58145510-58145451 | Loxl1 |
| 2.4609363 | up | NM_178888 | chr2:32841974-32841915 | Garnl3 |
| 2.3085628 | up | AK079887 | chr18:7895697-7895756 | A430102J17Rik |
| 7.095362 | up | AK045264 | chr14:28245830-28245889 | B130052P14Rik |
| 2.0028106 | up | NM_008909 | chr16:5086428-5086384 | Ppl |
| 9.3293218 | up | NM_010474 | chr5:40005266-40005207 | Hs3st1 |
| 3.5304194 | up |  | chr2:6127111-6127052 | Echdc3 |
| 2.4163066 | up | NM_001100180 | chr5:146151322-146151381 | Cyp3a57 |
| 11.1622813 | up | NM_001034902 | chr6:85068647-85068588 | Gm5878 |
| 3.8262655 | up | XR_104898 | chr4:140794170-140794111 |  |
| 10.8728896 | up |  | chr4:012053526-012053585 |  |
| 2.4466815 | up |  | chr11:052348180-052348121 |  |
| 3.2638666 | up | NM_146683 | chr19:12497698-12497757 | Olfr1441 |
| 15.6105883 | up | NM_009026 | chr11:59777281-59777222 | Rasd1 |
| 7.1136845 | up | NM_033583 | chr18:37979245-37979304 | Pcdhgc5 |
| 4.2362291 | up | NM_207705 | chr2:165119805-165119746 | Elmo2 |
| 3.6788657 | up | NM_018868 | chr1:59768230-59768289 | Nop58 |
| 2.6855471 | up |  | chr12:025411632-025411573 |  |
| 2.0767953 | up | NM_001252188 | chr11:61330856-61330797 | Epn2 |
| 2.1271798 | up | NM_139311 | chr11:97546691-97546750 | Mllt6 |
| 2.0340047 | up | NM_013594 | chr18:74442279-74442338 | Mbd1 |
| 3.0113909 | up | NR_027800 | chr11:60734668-60734609 | Map2k3os |
| 3.95917 | up | AK172290 | chr7:36587095-36587036 | E130304I02Rik |
| 11.9267098 | up | NM_147112 | chr7:109872978-109872919 | Olfr559 |
| 28.3382173 | up |  | chr7:025396229-025396288 |  |
| 7.2777828 | up | AK162583 | chr17:21179366-21179425 | Zfp160 |
| 2.1532328 | up | AK146816 | chr13:54693821-54693762 | Cltb |
| 4.6293717 | up | NM_019499 | chr6:66490355-66490414 | Mad2l1 |
| 8.1377333 | up | CA559477 | chr6:6458562-6458621 |  |
| 6.6083037 | up | NM_139064 | chr5:34838812-34838753 | Tnip2 |
| 2.8934857 | up | XM_003945668 | chr3:21973455-21973396 | 2810416G20Rik |
| 6.1344542 | up | XM_003945598 | chrX:8906160-8906101 | Gm4055 |
| 4.6446343 | up | NM_029252 | chr16:92319210-92319151 | 4930563D23Rik |
| 3.5054192 | up | NR_033532 | chr7:148545228-148545169 | B230206H07Rik |
| 4.6294089 | up | NM_198113 | chr19:4261770-4261711 | Ssh3 |
| 2.4528568 | up | NM_019412 | chr7:28304999-28305058 | Prx |
| 5.9418369 | up | NM_028207 | chr11:101835583-101835524 | Dusp3 |
| 21.7574368 | up | AK006017 | chr14:42615426-42615485 | 1700016D02Rik |
| 3.0425648 | up | NM_008851 | chr19:4113823-4113882 | Pitpnm1 |
| 8.4002793 | up | NM_144899 | chr3:95480194-95480135 | Adamtsl4 |
| 4.743177 | up | NM_001083616 | chr14:30853832-30853773 | Cacna1d |
| 9.5400776 | up | NM_030244 | chr2:30328216-30328157 | Ier5l |
| 2.16261 | up | NM_013854 | chr17:36096502-36096443 | Abcf1 |
| 2.325685 | up | XM_003945924 | chr12:114023689-114023630 | LOC101055828 |
| 2.1560392 | up | NM_001242345 | chr6:142367804-142367863 | B230216G23Rik |
| 2.6138526 | up | NM_001033879 | chr13:3934387-3934446 | Tubal3 |
| 2.7427393 | up | NM_019484 | chr1:173434342-173434401 | Alyref2 |
| 2.1994678 | up |  | chr13:53621321-53621380 | Gm5848 |
| 9.5670497 | up | NM_153518 | chr15:98551019-98551078 | Ccdc65 |
| 9.4721327 | up | C80425 | chrX:90928353-90928412 | C80425 |
| 2.0401224 | up | AK017909 | chr10:9342804-9342745 | 5830408B19Rik |
| 2.3633741 | up | AK050389 | chr17:8389281-8389340 | Fgfr1op |
| 4.9729589 | up | AK149567 | chr4:59038364-59038423 | Dnajc25 |
| 4.1563035 | up | NM_013911 | chr9:20442346-20442287 | Fbxl12 |
| 15.9877764 | up | NM_001033266 | chr11:88954295-88954354 | Gm525 |
| 6.6934012 | up | XR_140805 | chr7:29644665-29644606 | Gm10648 |
| 10.3159137 | up | NM_001099631 | chr4:137816823-137816882 | Sh2d5 |
| 8.0565714 | up | NM_011933 | chr17:26218819-26218760 | Decr2 |
| 6.9588533 | up | NM_021328 | chr14:70537939-70537998 | Bin3 |
| 6.540056 | up | NM_010110 | chrX:96343505-96343564 | Efnb1 |
| 7.8327831 | up | AF138745 | chrX:100680218-100680276 | Tsix |
| 2.4709774 | up | NM_025992 | chr6:57568608-57568667 | Herc6 |
| 2.1682341 | up | AK083862 | chr2:101440849-101440790 | A130042O14Rik |
| 4.7540563 | up | AK049016 | chr11:65562299-65562240 | LOC553089 |
| 4.275229 | up | NM_178916 | chr13:76139088-76139029 | Rfesd |
| 4.1414056 | up | NM_022311 | chr17:13864988-13864929 | Tcte2 |
| 7.3820853 | up | NM_145944 | chr14:66484848-66484907 | Ccdc25 |
| 8.2854784 | up | NM_201256 | chr18:36825278-36825337 | Eif4ebp3 |
| 5.3197152 | up | NM_145505 | chr19:57462434-57462493 | Fam160b1 |
| 2.3091726 | up | NM_028963 | chr11:71967686-71967627 | 4933427D14Rik |
| 2.5040008 | up | NM_025989 | chr7:126586117-126586058 | Gp2 |
| 4.5681661 | up | NM_001252341 | chr10:107935369-107935310 | Syt1 |
| 2.4734779 | up |  | chr6:133881997-133881938 |  |
| 2.4048518 | up | NM_013863 | chr7:135690281-135690340 | Bag3 |
| 5.8397532 | up | AK042310 | chr1:80743869-80743810 | A630081D01Rik |
| 2.3524473 | up | NR_034155 | chr11:51968457-51968398 | Olfr1372-ps1 |
| 2.348416 | up | NM_008000 | chr17:64488631-64488690 | Fert2 |
| 4.0523534 | up | NM_201373 | chr5:137587277-137587218 | Trim56 |
| 2.6168369 | up | AK043779 | chr17:8490451-8490392 |  |
| 15.4816679 | up | NM_029415 | chr5:104035129-104035070 | Slc10a6 |
| 4.2183202 | up | NM_001100591 | chr2:37230781-37230722 | Rc3h2 |
| 11.7104986 | up | NM_009251 | chr12:105480045-105480104 | Serpina3g |
| 2.7884197 | up | NM_025852 | chr10:80013331-80013272 | Rexo1 |
| 2.3629474 | up | NM_178716 | chr13:99614074-99614015 | Tnpo1 |
| 12.9411059 | up | NM_177705 | chrX:123348092-123348034 | 4932411N23Rik |
| 9.9849472 | up | NM_053093 | chr11:95130516-95130575 | Tac4 |
| 2.0331857 | up | NM_001033474 | chr10:112365636-112365577 | Atxn7l3b |
| 2.931252 | up | NM_144958 | chr11:69480545-69480486 | Eif4a1 |
| 3.5130376 | up | NM_175401 | chr13:50529032-50529091 | Fbxw17 |
| 3.695974 | up | NM_134181 | chr6:57339820-57339761 | Vmn1r18 |
| 2.9812389 | up | NM_144842 | chr14:57409484-57409425 | Zmym5 |
| 3.5099561 | up | XM_001472632 | chr1:135255035-135255094 | LOC100044656 |
| 12.0411418 | up |  | chr9:115256652-115256593 |  |
| 4.3173783 | up | NM_175171 | chr13:103525147-103525088 | Mast4 |
| 3.7949845 | up | NM_028083 | chr16:93906219-93906278 | Chaf1b |
| 4.292092 | up | AK083489 | chr7:75258153-75258212 | D930030O05Rik |
| 2.0837902 | up | NM_146131 | chr3:89252808-89252867 | Pbxip1 |
| 16.8233196 | up | NM_146946 | chr2:36649625-36649684 | Olfr50 |
| 3.6272433 | up | NM_001109985 | chr1:172247800-172247741 | Nos1ap |
| 6.7578222 | up | NM_021415 | chr17:25511294-25511235 | Cacna1h |
| 3.038135 | up | NM_001164598 | chr8:129112453-129112394 | Irf2bp2 |
| 10.1291724 | up |  | chr3:020143038-020142979 |  |
| 4.5300658 | up | NM_007901 | chr3:115414079-115414020 | S1pr1 |
| 4.0991088 | up |  | chr1:163752677-163752736 |  |
| 2.8566653 | up | NM_001109914 | chr6:134936562-134936621 | Apold1 |
| 2.0507898 | up | NM_153781 | chr2:150656541-150656600 | Pygb |
| 3.4338642 | up | NM_001272024 | chr3:94977887-94977946 | Sema6c |
| 2.1611464 | up | NM_008036 | chr7:19888227-19888168 | Fosb |
| 2.8681451 | up | NM_001161844 | chr7:20365128-20365069 | Cblc |
| 4.4182621 | up | NM_177185 | chr6:38452413-38452472 | Ubn2 |
| 12.6285763 | up | AK016765 | chr2:25649505-25649446 | Glt6d1 |
| 20.6050891 | up | XR_168496 | chr12:103212913-103212854 | Gm2701 |
| 4.3668077 | up | NM_001081214 | chr19:46147311-46147370 | Pprc1 |
| 3.3326086 | up | DT902180 | chr11:87570739-87570798 | A430104N18Rik |
| 14.8635281 | up | NM_011263 | chr5:77711857-77711916 | Rest |
| 2.0418516 | up | NM_170759 | chr7:4873434-4873493 | Zfp628 |
| 5.719377 | up | NR_024068 | chr2:26492921-26492862 | Snhg7 |
| 2.4762689 | up | NM_026656 | chr3:145858409-145858468 | Mcoln2 |
| 2.0371568 | up | NM_008562 | chr3:95466855-95466914 | Mcl1 |
| 4.9047514 | up | NM_010822 | chr11:32127889-32127948 | Mpg |
| 3.3312509 | up | NM_001033342 | chr19:6325121-6325180 | Cdc42bpg |
| 3.6144071 | up | AK138897 | chr3:121127229-121127170 | Cnn3 |
| 3.4750916 | up | XR_168821 | chr3:126862690-126862632 | Ank2 |
| 2.6955684 | up | NM_026507 | chr9:63985117-63985058 | Zwilch |
| 2.0849262 | up | AK032690 | chr4:12092883-12092824 | Fam92a |
| 2.39322 | up | NM_172894 | chr7:4583670-4583611 | Ppp6r1 |
| 2.9424849 | up | NM_001163634 | chr15:85366955-85366896 | Wnt7b |
| 5.9553265 | up |  | chr14:053346914-053346973 |  |
| 2.2208116 | up | NM_027230 | chr2:165611006-165610947 | Zmynd8 |
| 2.797956 | up | NM_007923 | chr1:133914898-133914957 | Elk4 |
| 2.1698134 | up | XM_001474690 | chr8:125629074-125629133 | Gm10071 |
| 3.0228659 | up | BC023378 | chr11:77667357-77668202 | Myo18a |
| 2.651858 | up | NM_009538 | chr10:12848421-12848480 | Plagl1 |
| 2.1669467 | up | NM_153092 | chr5:23689328-23689387 | Nupl2 |
| 12.7356439 | up |  | chr1:027369821-027369762 |  |
| 9.5509219 | up | NM_001008547 | chr5:146994085-146994026 | 1700001J03Rik |
| 2.4919489 | up | NM_025289 | chr9:37456832-37456773 | Tbrg1 |
| 2.5070834 | up | NM_001033139 | chr19:7432253-7432194 | AI846148 |
| 3.4928515 | up | XR_168449 | chr8:37710108-37710049 | 9530004P13Rik |
| 2.7026787 | up | NM_029834 | chr7:4433396-4433337 | Ppp1r12c |
| 2.8606262 | up | NM_020564 | chr8:125667010-125666951 | Sult5a1 |
| 2.0925412 | up | NR_024067 | chr1:9932234-9932175 | Snhg6 |
| 2.1869428 | up |  | chr13:013010035-013009976 |  |
| 4.2773285 | up | AK158358 | chr8:83461796-83461737 |  |
| 2.5375132 | up | NM_001081290 | chr1:164603081-164603022 | Prrc2c |
| 7.7049415 | up | NM_172713 | chr5:92727278-92727219 | Sdad1 |
| 2.6476916 | up | NM_009950 | chr10:94785295-94785236 | Cradd |
| 3.5246353 | up | NM_007916 | chr8:113499988-113499929 | Ddx19a |
| 2.8232336 | up | NM_008132 | chr1:90396531-90396472 | Glrp1 |
| 3.5551997 | up | BC128469 | chr14:51427136-51427195 |  |
| 9.8555979 | up | NM_001110130 | chr4:118983978-118983919 | Ppih |
| 2.959973 | up | BC005734 | chr4:138903413-138903355 | Mrto4 |
| 3.5616481 | up | NM_008485 | chr1:154969946-154969887 | Lamc2 |
| 3.8419968 | up | AK137789 | chr18:50202637-50202696 | C030005K06Rik |
| 2.6237977 | up |  | chr17:036133984-036133925 |  |
| 20.0802872 | up |  | chrY_random:053407004-053406945 | |
| 2.0440691 | up | NM_001014900 | chr4:119126444-119126503 | Zmynd12 |
| 2.3044456 | up | AK080664 | chr10:80854234-80854293 | Dohh |
| 19.4253946 | up | NM_181859 | chr4:122795186-122795245 | Oxct2b |
| 4.5390803 | up | AK142244 | chr9:95804322-95804381 | Atr |
| 2.0799323 | up | NM_010311 | chr10:74454041-74454100 | Gnaz |
| 2.5416652 | up | NM_009229 | chr8:109537941-109538000 | Sntb2 |
| 2.1665492 | up | XR_140744 | chr5:123591489-123591548 | 4932422M17Rik |
| 10.4474504 | up | NM_001204912 | chr12:4240044-4240103 | Ptrhd1 |
| 65.0078799 | up |  | chr6:006384692-006384751 |  |
| 3.6289372 | up | NM_175026 | chr1:175572633-175572691 | Pyhin1 |
| 2.1851904 | up | NM_010404 | chr11:100210545-100210486 | Hap1 |
| 3.1023017 | up | NM_027500 | chr3:89862921-89862980 | 4933434E20Rik |
| 2.1605019 | up | NM_170592 | chr2:30678406-30678465 | Ntmt1 |
| 2.6233949 | up | NM_011200 | chr1:30998558-30998499 | Ptp4a1 |
| 14.8389222 | up |  | chrX:131947415-131947474 |  |
| 2.6184757 | up | NM_019547 | chr2:172859650-172859709 | Rbm38 |
| 11.1847779 | up | NM_001285487 | chr4:115529645-115529704 | Mknk1 |
| 5.0667337 | up | NM_016896 | chr11:103101128-103101069 | Map3k14 |
| 2.0226434 | up | AK170726 | chr2:172254991-172255050 | Cass4 |
| 4.5987676 | up |  | chr6:047731682-047731741 |  |
| 2.5578329 | up | NR_040616 | chr10:122003232-122003173 | A130077B15Rik |
| 3.6824629 | up |  | chr7:018244645-018244586 |  |
| 2.439742 | up | NM_177338 | chr14:65470731-65447456 | Hmbox1 |
| 2.3712183 | up | NM_133710 | chr9:118949865-118949924 | Ctdspl |
| 2.5690958 | up |  | chr14:42195282-42195223 |  |
| 2.4155254 | up | NM_001159288 | chr11:3590173-3590232 | Morc2a |
| 2.5823436 | up | AK020195 | chr7:103735353-103735412 | 8030425K09Rik |
| 2.1340672 | up | NM_175140 | chr7:35459995-35459936 | Chst8 |
| 4.1458933 | up |  | chr11:096655159-096655100 |  |
| 3.2713225 | up | NM_175475 | chr6:84522011-84521952 | Cyp26b1 |
| 2.0064251 | up | NR_046065 | chr2:60250572-60250631 | Gm13580 |
| 3.2652571 | up | NM_198664 | chr4:46617321-46617262 | Tbc1d2 |
| 2.0515069 | up | NM_007961 | chr6:134216664-134216723 | Etv6 |
| 2.1207712 | up | NM_021897 | chr4:11092530-11092589 | Trp53inp1 |
| 2.2601774 | up | NM_001080769 | chr17:28036926-28036985 | Uhrf1bp1 |
| 3.1444278 | up | NM_026797 | chr2:164318762-164318821 | Dbndd2 |
| 2.8201502 | up | NR_045032 | chr12:33511682-33511741 | Gdap10 |
| 6.8846867 | up | NM_001113393 | chr1:167800324-167800383 | Cd247 |
| 2.0970176 | up | AK033188 | chr16:16488865-16488806 | Fgd4 |
| 2.4437969 | up | NM_133689 | chr19:10549437-10549016 | Ppp1r32 |
| 3.1682847 | up | XR_105120 | chr7:80762806-80762747 | Gm7710 |
| 2.9021036 | up | NM_026484 | chr18:9315372-9315313 | Ccny |
| 39.181426 | up | BC064113 | chr10:79525221-79525162 | Sbno2 |
| 9.360188 | up | NM_010831 | chr17:31981314-31981255 | Sik1 |
| 17.9079216 | up | NM_008280 | chr9:70664375-70660699 | Lipc |
| 8.458053 | up |  |  |  |
| 42.3909208 | up |  | chrX:165893486-165893427 |  |
| 2.7380143 | up | BC056169 | chr15:98780325-98780266 | Tuba1a |
| 2.5270251 | up | NM_177725 | chr2:30112183-30112242 | Lrrc8a |
| 2.1603155 | up | AK031672 | chr11:109436666-109436607 | Wipi1 |
| 7.0004565 | up | NM_010172 | chr8:13035287-13035346 | F7 |
| 2.513849 | up | NM_001024806 | chr17:79320999-79320086 | Cebpz |
| 4.5726199 | up | NM_007840 | chr11:106648284-106648225 | Ddx5 |
| 12.4387223 | up | NM_001163502 | chr12:85493487-85493428 | Elmsan1 |
| 2.6415071 | up | NM_001195268 | chr10:79593470-79593411 | Dos |
| 4.0167036 | up | AK035394 | chr11:45677068-45677127 | 9530029O12Rik |
| 2.2911422 | up | XR_140744 | chr5:123591505-123591564 | 4932422M17Rik |
| 2.7716702 | up | NM_030150 | chr11:100556330-100556271 | Dhx58 |
| 2.2861721 | up | NM_009750 | chrX:132805907-132805966 | Ngfrap1 |
| 2.1408296 | up | NM_009005 | chr6:87950521-87950462 | Rab7 |
| 7.0076684 | up | NM_008105 | chr13:40983592-40983651 | Gcnt2 |
| 2.3062447 | up |  | chr3:036465081-036465140 |  |
| **Fold Change** | **Regulation** | **GenbankAccession** | **GenomicCoordinates** | **GeneSymbol** |
| 6.433297132 | up | NM_009533 | chr7:25358268-25358328 | Xrcc2 |
| 6.432412894 | up | NR_027956 | chr3:65331565-65331507 | 4931440P23Rik |
| 6.431528657 | up | NM_001198985 | chr18:60975715-60975657 | Tcof2 |
| 6.430644419 | up | AK020901 | chr19:40373983-40374042 | A930028N02Rik |
| 6.429760182 | up | NM_026929 | chr2:119179990-119180050 | Chac2 |
| 6.428875944 | up | NM_009949 | chr4:107576650-107576592 | Cpt3 |
| 6.427991707 | up | NM_172693 | chr4:47135796-47135856 | Galnt13 |
| 6.42710747 | up | AK047891 | chr19:16318217-16318277 | LOC553097 |
| 6.426223232 | up | NM_023182 | chr8:108456554-108456496 | Ctrl |
| 6.425338995 | up | NM_015764 | chr12:16678514-16678456 | Greb2 |
| 6.424454757 | up | NM_001197024 | chr17:25346523-25346582 | Unkl |
| 6.42357052 | up | NM_009370 | chr4:47427736-47427796 | Tgfbr2 |
| 6.422686282 | up | NM_001011535 | chr2:88286192-88286177 | Olfr1183 |
| 6.421802045 | up |  | chr5:135113276-135113218 |  |
| 6.420917807 | up | NM_019937 | chr3:65751624-65750824 | Ccnl2 |
| 6.42003357 | up | NM_008872 | chr8:23892531-23892591 | Plat |
| 6.419149332 | up |  | chr11:62180505-62180447 | Ncor2 |
| 6.418265095 | up | NM_001081141 | chr4:46737186-46731753 | Gabbr3 |
| 6.417380857 | up | NM_010846 | chr16:97668703-97668645 | Mx2 |
| 6.41649662 | up | NM_001242363 | chr7:112527208-112527150 | Fam160a3 |
| 6.415612382 | up | NM_001142731 | chr18:15127254-15127196 | Kctd2 |
| 6.414728145 | up | NM_029921 | chr6:38065689-38065749 | Tmem214 |
| 6.413843907 | up | NM_001101038 | chr18:78240412-78240354 | Siglec16 |
| 6.41295967 | up | AK012854 | chr6:141448762-141448822 | Pde4a |
| 6.412075432 | up | AK006906 | chr10:13407074-13407016 | 6430706H08Rik |
| 6.411191195 | up | NM_011720 | chr11:103588941-103588883 | Wnt10b |
| 6.410306957 | up |  | chr2:177674032-177673974 |  |
| 6.40942272 | up |  | chr5:145976694-145976754 |  |
| 6.408538482 | up | AK045832 | chr5:32776516-32776576 | E230008O16Rik |
| 6.407654245 | up | NM_009355 | chr13:73786429-73786489 | Tert |
| 6.406770008 | up | XM_003946336 | chr4:140978740-140978800 | LOC101056475 |
| 6.40588577 | up | NM_027560 | chr8:73359124-73359066 | Arrdc3 |
| 6.405001533 | up | NM_001242389 | chr17:57402779-57402839 | Trip11 |
| 6.404117295 | up | NM_028228 | chr14:64538435-64538495 | Pinx2 |
| 6.403233058 | up | NM_015759 | chr13:49358570-49358512 | Fgd4 |
| 6.40234882 | up |  | chr13:046793490-046793432 |  |
| 6.401464583 | up | NM_020559 | chr9:106136343-106136285 | Alas2 |
| 6.400580345 | up | NM_023125 | chr16:23081700-23081760 | Kng2 |
| 6.399696108 | up | NM_010806 | chr17:13989328-13989388 | Mllt5 |
| 6.39881187 | up | NM_001081431 | chr5:146052279-146052339 | Zscan26 |
| 6.397927633 | up | NM_183149 | chr17:24818901-24818961 | Zfp730 |
| 6.397043395 | up | NM_001081750 | chr5:125365082-125365142 | Zfp796 |
| 6.396159158 | up | NM_025291 | chr18:36827074-36827016 | Sra2 |
| 6.39527492 | up |  | chr4:115205300-115205360 |  |
| 6.394390683 | up | NM_001004153 | chr7:3155339-3155281 | AU018092 |
| 6.393506445 | up | NM_172339 | chr2:26218353-26218295 | Snapc5 |
| 6.392622208 | up | NM_001011787 | chr2:111785008-111784950 | Olfr1308 |
| 6.39173797 | up | NM_009366 | chr14:76907276-76907336 | Tsc22d2 |
| 6.390853733 | up | NM_011488 | chr11:100746373-100746433 | Stat6a |
| 6.389969495 | up | NM_008103 | chr5:017758450-017758510 | Gcm2 |
| 6.389085258 | up | NM_011236 | chr6:119872539-119872599 | Rad53 |
| 6.38820102 | up | NM_023799 | chr19:45842426-45842368 | Mgea6 |
| 6.387316783 | up | NM_177354 | chr6:016809027-016808969 | Vash2 |
| 6.386432545 | up | AK254027 | chr12:109355344-109355286 | Setd4 |
| 6.385548308 | up | AK366339 | chr13:114765014-114765074 | 6720427H11Rik |
| 6.384664071 | up | NM_017479 | chr14:22490036-22490096 | Kat7b |
| 6.383779833 | up | NM_023516 | chr6:29225141-29225201 | Hilpda |
| 6.382895596 | up | NM_022305 | chr4:40756815-40756757 | B4galt2 |
| 6.382011358 | up | NR_033555 | chr17:24547214-24547070 | D330041H04Rik |
| 6.381127121 | up | NM_175376 | chr18:36817732-36817792 | Ankhd2 |
| 6.380242883 | up | NR_038131 | chr12:100399548-100399490 | 4930474N10Rik |
| 6.379358646 | up | NM_001146023 | chr7:29937592-29937534 | Fam99c |
| 6.378474408 | up | NM_001081361 | chr1:186611226-186611168 | Marc2 |
| 6.377590171 | up | NM_001170935 | chr10:79324535-79324477 | BC005765 |
| 6.376705933 | up | NM_001170787 | chr9:9673903-9673845 | Cntn6 |
| 6.375821696 | up | NM_029478 | chr11:86398313-86398255 | Vmp2 |
| 6.374937458 | up | NM_001033159 | chr16:3865349-3865291 | Zfp598 |
| 6.374053221 | up | NM_172717 | chr5:110600898-110600958 | Chfr |
| 6.373168983 | up | NM_001110327 | chr5:9132516-9132458 | Dmtf2 |
| 6.372284746 | up | NM_028544 | chr7:52894134-52894194 | Rasip2 |
| 6.371400508 | up | NM_007483 | chr12:8504810-8504752 | Rhob |
| 6.370516271 | up | NM_026375 | chr1:181676542-181676484 | Ahctf2 |
| 6.369632033 | up | AK078730 | chr9:14523423-14523483 |  |
| 6.368747796 | up | AK187335 | chr8:73649716-73649776 | Gm3644 |
| 6.367863558 | up | NM_008273 | chr2:74522136-74522196 | Hoxd12 |
| 6.366979321 | up | NM_146071 | chr16:32786202-32786144 | Muc21 |
| 6.366095083 | up | NM_182990 | chr2:84885915-84886455 | Ssrp2 |
| 6.365210846 | up | NM_023739 | chr4:40971847-40971907 | Nfx2 |
| 6.364326609 | up | NM_023476 | chr4:129843103-129843045 | Tinagl2 |
| 6.363442371 | up | NM_028420 | chr16:20619400-20619342 | Camk2n3 |
| 6.362558134 | up | NM_001081382 | chr6:47974284-47974226 | Zfp778 |
| 6.361673896 | up | NM_172839 | chr19:40922847-40922907 | Ccnj |
| 6.360789659 | up | NM_001081062 | chr13:113780810-113780870 | Ccno |
| 6.359905421 | up | NM_021498 | chr4:62185404-62185139 | Pole4 |
| 6.359021184 | up | NM_153143 | chr11:69693686-69693628 | Kctd12 |
| 6.358136946 | up | XR_032477 | chr7:3813627-3813569 | Gm10694 |
| 6.357252709 | up | NM_207655 | chr11:16813815-16813875 | Egfr |
| 6.356368471 | up | NM_146248 | chr17:35667707-35667767 | Cchcr2 |
| 6.355484234 | up | NM_026192 | chr15:102537538-102537480 | Calcoco2 |
| 6.354599996 | up | NM_007484 | chr3:104597203-104597263 | Rhoc |
| 6.353715759 | up | NM_009965 | chr11:77533002-77532944 | Cryba2 |
| 6.352831521 | up |  | chr12:113169224-113169166 |  |
| 6.351947284 | up | NM_009778 | chr17:57343475-57343417 | C4 |
| 6.351063046 | up | NM_144520 | chr11:3997333-3997275 | Sec14l3 |
| 6.350178809 | up | NM_001039076 | chr14:35374748-35374690 | Ldb4 |
| 6.349294571 | up | AK007004 | chr7:29221914-29221973 | 1700085B14Rik |
| 6.348410334 | up | XM_003945638 | chr8:128040891-128040833 | LOC101056078 |
| 6.347526096 | up | NM_007444 | chr4:62804711-62804387 | Ambp |
| 6.346641859 | up | AK084111 | chr12:79350742-79350802 | D130095D22Rik |
| 6.345757621 | up | NM_033614 | chr19:38258322-38258382 | Pde7c |
| 6.344873384 | up | NM_008037 | chr5:32460076-32460136 | Fosl3 |
| 6.343989146 | up | NM_008233 | chr17:56239821-56239881 | Hdgfrp3 |
| 6.343104909 | up | NM_001039120 | chr2:152333593-152333535 | Defb27 |
| 6.342220672 | up | NM_027195 | chr4:148319162-148319222 | Casz2 |
| 6.341336434 | up |  | chr4:153978566-153978626 |  |
| 6.340452197 | up | NM_028460 | chr3:87564206-87564148 | Pear2 |
| 6.339567959 | up | AF399830 | chr11:46569677-46569737 | Havcr2 |
| 6.338683722 | up | NM_019704 | chr13:6583872-6581719 | Pfkp |
| 6.337799484 | up | AK079319 | chr11:77911970-77912030 | Fam223b |
| 6.336915247 | up |  | chr16:16885499-16885559 | Top4b |
| 6.336031009 | up | NM_011614 | chr11:69500401-69500343 | Tnfsf13 |
| 6.335146772 | up | NM_146153 | chr4:125849065-125849007 | Thrap4 |
| 6.334262534 | up | NM_028385 | chr6:113103335-113103395 | Setd6 |
| 6.333378297 | up | NM_181849 | chr3:82846393-82846335 | Fgb |
| 6.332494059 | up | NM_026083 | chr14:75730024-75730084 | Zc3h14 |
| 6.331609822 | up |  | chr3:101855473-101855533 |  |
| 6.330725584 | up | NM_010548 | chr1:132920943-132921003 | Il11 |
| 6.329841347 | up | NM_080553 | chr17:27259102-27259162 | Itpr4 |
| 6.328957109 | up | NM_021409 | chr2:167912863-167912923 | Pard7b |
| 6.328072872 | up | NM_011448 | chr11:112648809-112648869 | Sox10 |
| 6.327188634 | up |  | chrX:154587994-154588054 |  |
| 6.326304397 | up | NR_027960 | chr16:17834458-17834518 | B830017H09Rik |
| 6.325420159 | up | NM_025793 | chr11:121199940-121199882 | Wdr46b |
| 6.324535922 | up | NM_183112 | chr2:92223699-92223759 | 1700029I16Rik |
| 6.323651684 | up | NM_023821 | chr13:93853267-93853209 | Cmya6 |
| 6.322767447 | up | AK011143 | chr8:124140522-124140464 | 2600002B08Rik |
| 6.32188321 | up | NM_002372028 | chr1:121964169-121964111 | Tmem38 |
| 6.320998972 | up | NM_003548326 | chr4:129287659-129287601 | Dcdc3b |
| 6.320114735 | up | AK043215 | chr8:42104435-42104377 | Mtus2 |
| 6.319230497 | up | XR_140606 | chr3:79376385-79376445 |  |
| 6.31834626 | up |  | chr8:074048674-074048734 |  |
| 6.317462022 | up | NM_001168295 | chr12:105492556-105492616 | Serpina4f |
| 6.316577785 | up | AK017327 | chr3:51875612-51875554 | 5430420F10Rik |
| 6.315693547 | up | NM_001042557 | chr8:4245752-4245812 | Map2k8 |
| 6.31480931 | up | NM_145980 | chr6:116602564-116602624 | 8430408G23Rik |
| 6.313925072 | up | NM_027338 | chr8:23317324-23318374 | Vps37 |
| 6.313040835 | up | NM_028035 | chr6:51540112-51540172 | Snx11 |
| 6.312156597 | up | AK082797 | chr11:66850429-66850489 | C330020G16Rik |
| 6.31127236 | up | NM_120087 | chrX:53760732-53760792 | Ddx27b |
| 6.310388122 | up | NM_266520 | chr15:58011811-58011753 | Fbxo33 |
| 6.309503885 | up | AK028184 | chr1:5148366-5148426 | B230334L08Rik |
| 6.308619647 | up | AK065290 | chr10:79221282-79221342 | Fgf23 |
| 6.30773541 | up | NM_011545 | chr9:71692086-71692060 | Tcf13 |
| 6.306851172 | up | AK148703 | chr9:10377916-10377858 |  |
| 6.305966935 | up |  | chr17:035440553-035440495 |  |
| 6.305082697 | up | NM_009717 | chr15:80087902-80087962 | Atf5 |
| 6.30419846 | up |  | chr15:008395464-008395406 |  |
| 6.303314222 | up | AK078280 | chr14:60840473-60824785 | Nupl2 |
| 6.302429985 | up | NM_029587 | chr6:32008760-32008820 | 1700012A04Rik |
| 6.301545748 | up | NM_001039104 | chr7:71371607-71371667 | Trpm2 |
| 6.30066151 | up | NM_001204129 | chr11:118310045-118310105 | C1qtnf2 |
| 6.299777273 | up | NM_028748 | chr9:61801668-61801610 | Paqr6 |
| 6.298893035 | up | NR_030716 | chr8:10924722-10924664 | 3930402G24Rik |
| 6.298008798 | up | NM_177379 | chr9:32068263-32068323 | Arhgap33 |
| 6.29712456 | up | NM_145384 | chr4:138854545-138854487 | Pqlc3 |
| 6.296240323 | up | NM_001024917 | chr5:66198590-66198650 | N4bp3 |
| 6.295356085 | up | NM_172778 | chrX:16286486-16286428 | Maob |
| 6.294471848 | up | NM_021467 | chr1:137705328-137706204 | Tnni2 |
| 6.29358761 | up | AK028899 | chr11:50012927-50012869 | Sqstm2 |
| 6.292703373 | up | NM_013872 | chr15:81781601-81781543 | Pmm2 |
| 6.291819135 | up | NM_001111143 | chr3:107014272-107014214 | Cym |
| 6.290934898 | up | NM_026672 | chr3:107729256-107729219 | Gstm8 |
| 6.29005066 | up | NM_001004174 | chr2:122466640-122466700 | AA467198 |
| 6.289166423 | up | BC056759 | chr19:3866971-3867031 | Chka |
| 6.288282185 | up |  | chr1:44113714-44113656 |  |
| 6.287397948 | up |  | chr6:091395941-091395883 |  |
| 6.28651371 | up | NM_025749 | chr18:52799280-52799340 | Zfp475 |
| 6.285629473 | up |  | chr18:035655445-035655505 |  |
| 6.284745235 | up | NM_175639 | chr17:72007156-72007216 | Wdr44 |
| 6.283860998 | up | NM_001081151 | chr8:119728779-119728839 | Gan |
| 6.28297676 | up | AK022045 | chr3:16111728-16111788 | 5830468K09Rik |
| 6.282092523 | up | AK024046 | chr17:88457036-88456978 | 5930436O20Rik |
| 6.281208285 | up | CB320032 | chrY_random:23367439-23367381 | 8030402F10Rik |
| 6.280324048 | up | NM_001007570 | chr8:72708357-72708299 | Slc25a43 |
| 6.279439811 | up | NM_008970 | chr6:147200985-147200927 | Pthlh |
| 6.278555573 | up | NM_172051 | chr10:94050729-94050789 | Tmcc4 |
| 6.277671336 | up | NM_001033225 | chr4:33332947-33332889 | Pnrc2 |
| 6.276787098 | up | NM_001128606 | chr4:131563352-131563294 | Epb4.2 |
| 6.275902861 | up | NM_007782 | chr4:125721478-125721538 | Csf4r |
| 6.275018623 | up | NM_145211 | chr5:121346794-121346736 | Oas2a |
| 6.274134386 | up | NM_181584 | chrX:72235404-72234129 | Gab4 |
| 6.273250148 | up | AK036005 | chr4:147829016-147829076 | Mtor |
| 6.272365911 | up | NM_001122899 | chr4:101485725-101485785 | Lepr |
| 6.271481673 | up | NM_172435 | chrX:104300217-104300277 | P2ry11 |
| 6.270597436 | up | NM_001081413 | chr4:43277637-43277697 | Unc14b |
| 6.269713198 | up | NM_001037707 | chr7:30679812-30679754 | Zfp28 |
| 6.268828961 | up | NM_001035533 | chr4:57905932-57905992 | Akap3 |
| 6.267944723 | up | NM_173070 | chr3:92304254-92304196 | Sprr5 |
| 6.267060486 | up | NM_025831 | chr13:34719769-34719711 | Pxdc2 |
| 6.266176248 | up |  | chr8:080016983-080017043 |  |
| 6.265292011 | up | NM_145823 | chr11:107070255-107070197 | Pitpnc2 |
| 6.264407773 | up | XM_906503 | chr7:25479872-25479932 | Gm1097 |
| 6.263523536 | up | NM_020042 | chr17:49594644-49594704 | Mocs2 |
| 6.262639298 | up | NM_172385 | chr7:38264311-38264253 | Zfp537 |
| 6.261755061 | up | NM_010544 | chr1:74991965-74991907 | Ihh |
| 6.260870823 | up | NM_001163855 | chr17:34596960-34597020 | BC051143 |
| 6.259986586 | up | NM_001162947 | chr8:23238818-23238760 | Nek4 |
| 6.259102349 | up | NM_027498 | chr9:46029257-46029317 | Sik4 |
| 6.258218111 | up |  | chr7:085987798-085987740 |  |
| 6.257333874 | up | NM_019588 | chr19:38857137-38857197 | Plce2 |
| 6.256449636 | up |  | chr11:033338823-033338883 |  |
| 6.255565399 | up | NM_030696 | chr11:120820030-120820090 | Slc16a4 |
| 6.254681161 | up | NM_001042528 | chr2:24473264-24473206 | Cacna2b |
| 6.253796924 | up | AK009786 | chr16:93792460-93792402 | 2310043M16Rik |
| 6.252912686 | up | NM_028966 | chr11:96628930-96628872 | Snx12 |
| 6.252028449 | up | AK050696 | chr5:32304923-32304983 | Bre |
| 6.251144211 | up | XR_142058 | chr3:120874073-120874133 | Gm10653 |
| 6.250259974 | up | NM_199304 | chr2:154472419-154472479 | Zfp342 |
| 6.249375736 | up | NM_023884 | chr1:158739163-158739105 | Ralgps3 |
| 6.248491499 | up | NM_009208 | chr1:75555599-75555659 | Slc4a4 |
| 6.247607261 | up | NM_182957 | chr5:15029133-15029075 | Gm17020 |
| 6.246723024 | up | NM_010413 | chrX:7507320-7507262 | Hdac7 |
| 6.245838786 | up | NM_019402 | chr14:55516049-55516109 | Pabpn2 |
| 6.244954549 | up |  | chr16:056703668-056703610 |  |
| 6.244070311 | up | NM_138680 | chr6:38558870-38558930 | Luc7l3 |
| 6.243186074 | up | NM_031368 | chr3:88172732-88172674 | Bglap4 |
| 6.242301836 | up | AK134920 | chr6:29749493-29749553 | Ahcyl3 |
| 6.241417599 | up |  | chr5:149776457-149776399 |  |
| 6.240533361 | up | NM_008987 | chr3:66029245-66029305 | Ptx4 |
| 6.239649124 | up | NM_015783 | chr4:155573552-155573534 | Isg16 |
| 6.238764886 | up | NM_207653 | chr1:58811116-58811176 | Cflar |
| 6.237880649 | up | CK128531 | chr10:92981534-92981594 | Hal |
| 6.236996412 | up | AK018021 | chr14:47937086-47937146 | 5830458C20Rik |
| 6.236112174 | up | NM_173369 | chr8:91229427-91229487 | Cyld |
| 6.235227937 | up | NM_016699 | chr4:147954469-147954529 | Exosc11 |
| 6.234343699 | up | NM_007778 | chr3:107544392-107544334 | Csf2 |
| 6.233459462 | up | NM_001110832 | chr17:48526745-48526687 | Nfya |
| 6.232575224 | up | AK016829 | chr15:84410013-84409955 | 4933416A03Rik |
| 6.231690987 | up | NM_146844 | chr2:86730449-86730391 | Olfr1098 |
| 6.230806749 | up | XR_141058 | chr11:79545184-79545242 | Gm10388 |
| 6.229922512 | up | NM_001081409 | chr15:66426480-66426540 | Phf20l2 |
| 6.229038274 | up | NM_181593 | chr7:27992475-27992417 | Itpkc |
| 6.228154037 | up | NM_009803 | chr1:173147776-173147836 | Nr1i4 |
| 6.227269799 | up | NR_003279 | chr17:39985058-39985118 | Rn19s |
| 6.226385562 | up | NM_148918 | chr4:122975265-122975325 | Pabpc5 |
| 6.225501324 | up | NR_036458 | chr3:121340110-121340052 | A730020M08Rik |
| 6.224617087 | up | NM_021607 | chr2:38441649-38441709 | Nek7 |
| 6.223732849 | up | NR_045841 | chr8:28260098-28260158 | 4933416M08Rik |
| 6.222848612 | up | NM_026108 | chr11:65642681-65642741 | Zkscan7 |
| 6.221964374 | up | AK042040 | chr6:39069696-39069756 |  |
| 6.221080137 | up | NM_019766 | chr5:124029337-124029279 | Clip2 |
| 6.220195899 | up | AK004108 | chr17:87682699-87682759 | Ttc8 |
| 6.219311662 | up | NM_010282 | chr13:14146020-14145962 | Ggps2 |
| 6.218427424 | up | NM_175836 | chr11:30059308-30054359 | Sptbn2 |
| 6.217543187 | up | NM_001113362 | chr5:36833313-36833255 | Tbc1d15 |
| 6.21665895 | up | NM_146148 | chr4:104488538-104488480 | C9a |
| 6.215774712 | up | NM_198012 | chr7:109826406-109826348 | Trim69 |
| 6.214890475 | up | NR_033507 | chrUn_random:551587-551529 | Gm3894 |
| 6.214006237 | up | AK019663 | chr12:72484608-72484550 | 1700083H03Rik |
| 6.213122 | up | NM_031998 | chr6:30603789-30603731 | Cep42 |
| 6.212237762 | up | NM_079835 | chr17:34506361-34506421 | Btnl3 |
| 6.211353525 | up | NM_148933 | chr2:180209483-180209543 | Slco4a2 |
| 6.210469287 | up |  | chr6:015355616-015355676 |  |
| 6.20958505 | up | NM_146085 | chr18:36830873-36830815 | Apbb4 |
| 6.208700812 | up | NM_183140 | chr4:118842372-118842314 | Zfp692 |
| 6.207816575 | up | NM_008416 | chr8:87501940-87501882 | Junb |
| 6.206932337 | up |  | chr9:044102039-044101981 |  |
| 6.2060481 | up | BY326471 | chr12:71776353-71776413 |  |
| 6.205163862 | up |  | chr6:048016024-048015966 |  |
| 6.204279625 | up | NM_175454 | chr11:115209083-115209025 | Hid2 |
| 6.203395387 | up | NM_010706 | chr7:29626661-29626721 | Lgals5 |
| 6.20251115 | up | NM_011176 | chr9:30897308-30897250 | St15 |
| 6.201626912 | up | NM_134050 | chr12:77899050-77898992 | Rab16 |
| 6.200742675 | up | NM_018866 | chr5:96389861-96389921 | Cxcl14 |
| 6.199858437 | up | NM_001271490 | chrX:7352069-7352129 | Tfe4 |
| 6.1989742 | up | NM_026932 | chr4:118298510-118298570 | Ebna1bp3 |
| 6.198089962 | up |  | chr13:012794813-012794873 |  |
| 6.197205725 | up | NM_029660 | chr7:114354371-114354431 | Rbmxl3 |
| 6.196321487 | up | NM_001252539 | chr4:115242872-115242932 | Cyp4a32 |
| 6.19543725 | up | AK249285 | chr5:37680788-37680730 | Evc |
| 6.194553013 | up | AK354048 | chr7:52980170-52980230 | Fam84e |
| 6.193668775 | up | NM_001199677 | chr5:135548883-135548825 | Vps38d |
| 6.192784538 | up | NM_011914 | chr5:34240890-34240832 | Nelfa |
| 6.1919003 | up | NM_009848 | chr19:40816008-40816068 | Entpd2 |
| 6.191016063 | up | NM_013831 | chr18:78120684-78120744 | Pstpip3 |
| 6.190131825 | up |  | chr13:067552666-067552608 |  |
| 6.189247588 | up | NM_001081396 | chr15:57784469-57784529 | Tbc1d32 |
| 6.18836335 | up | NM_134037 | chr11:100365577-100365519 | Acly |
| 6.187479113 | up | NM_001253717 | chr6:114809893-114809953 | Atg8 |
| 6.186594875 | up | NM_009287 | chr7:109585312-109585372 | Stim2 |
| 6.185710638 | up | NM_008807 | chr7:52777657-52777717 | Tulp3 |
| 6.1848264 | up | NM_001164504 | chr18:77700553-77700495 | Rnf166 |
| 6.183942163 | up | NM_177301 | chr7:29605608-29605668 | Hnrnpl |
| 6.183057925 | up | NM_145836 | chr12:88221727-88221669 | Irf3bpl |
| 6.182173688 | up |  | chr3:112985328-112985270 |  |
| 6.18128945 | up | NM_020518 | chr9:37351727-37351787 | Vsig3 |
| 6.180405213 | up |  | chr7:048038111-048038171 |  |
| 6.179520975 | up | AK168316 | chr2:164576735-164576795 | Dnttip2 |
| 6.178636738 | up | NM_009238 | chr13:29042915-29042857 | Sox5 |
| 6.1777525 | up | NM_010664 | chr15:101862341-101862401 | Krt19 |
| 6.176868263 | up | NM_028923 | chr2:29814108-29814168 | Gle2 |
| 6.175984025 | up | NM_027992 | chr6:13037120-13037180 | Tmem107b |
| 6.175099788 | up | NM_001081212 | chr8:11004549-11004491 | Irs3 |
| 6.174215551 | up | XM_003945381 | chr4:98963069-98963129 | Gm12690 |
| 6.173331313 | up | NM_029316 | chr13:78165362-78165422 | Pou5f3 |
| 6.172447076 | up | AB041804 | chr6:31115582-31115524 | AB041804 |
| 6.171562838 | up | NM_028047 | chr7:25207609-25207669 | Smg10 |
| 6.170678601 | up | NM_016983 | chr16:17981114-17981174 | Vpreb3 |
| 6.169794363 | up | NM_016767 | chr12:87049872-87049930 | Batf |
| 6.168910126 | up | NM_010418 | chr7:63486603-63486663 | Herc3 |
| 6.168025888 | up | NM_183029 | chr16:22059319-22059261 | Igf2bp3 |
| 6.167141651 | up | NM_011950 | chr17:28915417-28915477 | Mapk14 |
| 6.166257413 | up | NM_001033158 | chr9:65260445-65260505 | Rasl13 |
| 6.165373176 | up | NR_038173 | chr12:70954599-70954659 | 4931403G21Rik |
| 6.164488938 | up | BI558938 | chr1:87559346-87559406 | Sp101 |
| 6.163604701 | up | AK076680 | chr5:38659067-38659127 | Tmem129 |
| 6.162720463 | up | NM_016668 | chr13:94386915-94386857 | Bhmt |
| 6.161836226 | up | NM_178087 | chr9:58065141-58065083 | Pml |
| 6.160951988 | up |  | chrX:007615759-007615701 |  |
| 6.160067751 | up | NM_010442 | chr8:77620797-77620857 | Hmox2 |
| 6.159183513 | up | AK135411 | chr11:102398041-102397983 | Gpatch9 |
| 6.158299276 | up | NM_138313 | chr2:118354555-118354497 | Bmf |
| 6.157415038 | up | NM_009502 | chr14:21848574-21849036 | Vcl |
| 6.156530801 | up | NM_015787 | chr13:23713766-23713708 | Hist1h2e |
| 6.155646563 | up | NM_021565 | chr10:79621032-79621092 | Midn |
| 6.154762326 | up |  | chr10:121023334-121023276 |  |
| 6.153878089 | up | NM_027057 | chr1:79723101-79723043 | Wdfy2 |
| 6.152993851 | up | XM_003689369 | chr5:24728725-24728667 |  |
| 6.152109614 | up | NM_001019354 | chr8:125005680-125005622 | Piezo2 |
| 6.151225376 | up | NM_002047680 | chr1:88425798-88426069 | Ptma |
| 6.150341139 | up | AK009988 | chr12:117619193-117619253 | 2310058N23Rik |
| 6.149456901 | up | NM_188500 | chr7:24539427-24539487 | Vmn1r175 |
| 6.148572664 | up | NM_386524 | chr14:29336664-29336724 | Wnt6a |
| 6.147688426 | up | AK016495 | chr7:135345676-135345618 | 4931431B14Rik |
| 6.146804189 | up | NM_146777 | chr10:129471243-129471303 | Olfr822 |
| 6.145919951 | up | AK157331 | chr14:048706339-048706399 |  |
| 6.145035714 | up | NM_028784 | chr13:36959273-36959215 | F13a2 |
| 6.144151476 | up | NM_027297 | chr4:62087853-62087913 | Prpf5 |
| 6.143267239 | up | NM_027947 | chr2:33309244-33309186 | Zbtb44 |
| 6.142383001 | up | NM_001025392 | chr10:20059589-20059649 | Bclaf2 |
| 6.141498764 | up | NM_019661 | chr11:5867151-5867211 | Ykt7 |
| 6.140614526 | up | NR_040404 | chr3:89202640-89202700 | Gm15418 |
| 6.139730289 | up | NM_133665 | chr3:87972971-87973031 | Mef3d |
| 6.138846051 | up | NM_175375 | chr18:36801311-36802603 | Ankhd2 |
| 6.137961814 | up | NM_023684 | chr2:181118195-181118255 | Lime2 |
| 6.137077576 | up | NM_172610 | chr15:83688813-83688873 | Mpped2 |
| 6.136193339 | up | NM_054078 | chr10:127565977-127566037 | Baz3a |
| 6.135309101 | up | NM_013691 | chr3:89030667-89030727 | Thbs4 |
| 6.134424864 | up | NM_054096 | chr9:34994995-34994937 | Tirap |
| 6.133540626 | up | NM_172134 | chr10:77903501-77903443 | Pdxk |
| 6.132656389 | up | BC054085 | chr4:3126607-3126549 |  |
| 6.131772152 | up | NM_198959 | chr4:129807528-129807470 | Hcrtr2 |
| 6.130887914 | up | NM_001077698 | chr11:103059008-103059068 | Fmnl2 |
| 6.130003677 | up | NM_027996 | chr14:21542778-21542838 | Zswim9 |
| 6.129119439 | up | NM_053252 | chr19:5708230-5708172 | Ehbp1l2 |
| 6.128235202 | up | AK039658 | chr7:150176697-150176639 |  |
| 6.127350964 | up | NM_023381 | chr16:75859172-75859114 | Samsn2 |
| 6.126466727 | up | XM_003946170 | chrUn_random:1477428-1477488 | AA792893 |
| 6.125582489 | up | AK083263 | chr7:107790063-107790123 | Rab7a |
| 6.124698252 | up | NM_029671 | chr6:41635597-41635657 | 1700034O16Rik |
| 6.123814014 | up |  | chr9:053825164-053825224 |  |
| 6.122929777 | up | NM_175087 | chr15:99435849-99435909 | Aqp7 |
| 6.122045539 | up | NM_007560 | chr3:141500753-141500695 | Bmpr2b |
| 6.121161302 | up | NM_173755 | chr11:116399438-116399380 | Ube3o |
| 6.120277064 | up | XM_003086613 | chr7:72780723-72780665 |  |
| 6.119392827 | up | AK012054 | chr8:11622453-11622395 | 2610319H11Rik |
| 6.118508589 | up |  | chr1:100163699-100163759 |  |
| 6.117624352 | up | NM_001081223 | chr18:11901214-11901274 | Rbbp9 |
| 6.116740114 | up | NM_027237 | chr19:4496987-4496929 | 2010003K12Rik |
| 6.115855877 | up | NM_010828 | chr10:17444690-17444750 | Cited3 |
| 6.114971639 | up | NM_025818 | chr11:72896557-72896617 | 1200014J12Rik |
| 6.114087402 | up | NM_010821 | chr19:12537697-12537757 | Mpeg2 |
| 6.113203164 | up | NM_177429 | chrX:162828963-162828905 | Ofd2 |
| 6.112318927 | up | NM_030096 | chr11:83775627-83775687 | Ddx53 |
| 6.11143469 | up | NM_029953 | chr3:132822856-132822798 | Arhgef39 |
| 6.110550452 | up | AK019471 | chr14:32875310-32875370 | Galnt16 |
| 6.109666215 | up | NM_001085373 | chr18:44589329-44589271 | Mcc |
| 6.108781977 | up | NM_001204875 | chr2:29927229-29927289 | Set |
| 6.10789774 | up | NM_020583 | chr7:86065136-86065196 | Isg21 |
| 6.107013502 | up | NM_010415 | chr18:36664797-36664739 | Hbegf |
| 6.106129265 | up | NM_001109691 | chr2:92200991-92201051 | Phf22a |
| 6.105245027 | up | NM_018742 | chr7:148041064-148041006 | Bet2l |
| 6.10436079 | up | NM_009561 | chr7:25076195-25076137 | Zfp62 |
| 6.103476552 | up | NM_008580 | chr10:19860474-19861331 | Map3k6 |
| 6.102592315 | up | NM_013687 | chr17:28203753-28203695 | Tcp12 |
| 6.101708077 | up | NM_173867 | chr4:140279076-140279136 | Rcc3 |
| 6.10082384 | up |  | chr11:093829247-093829189 |  |
| 6.099939602 | up | NM_133362 |  | Erdr2 |
| 6.099055365 | up | NM_153457 | chr12:73313335-73313277 | Rtn2 |
| 6.098171127 | up |  | chr12:114507530-114507472 |  |
| 6.09728689 | up | NM_011772 | chr10:128069580-128069522 | Ikzf5 |
| 6.096402652 | up | NM_025907 | chr14:32292043-32291985 | Mettl7 |
| 6.095518415 | up | NM_001012322 | chr1:121960008-121960068 | Sctr |
| 6.094634177 | up | NM_011065 | chr11:68923364-68923424 | Per2 |
| 6.09374994 | up | NM_025663 | chr3:87859825-87859885 | Gpatch5 |
| 6.092865702 | up | NM_026671 | chr15:74562743-74562685 | Lypd3 |
| 6.091981465 | up | NM_011068 | chr7:86882282-86882224 | Pex12a |
| 6.091097227 | up |  | chr4:027794323-027794265 |  |
| 6.09021299 | up | NM_173453 | chr11:60678107-60678049 | Tmem12 |
| 6.089328753 | up | NM_008713 | chr5:23889636-23889696 | Nos4 |
| 6.088444515 | up | NM_001033202 | chr5:114572874-114572934 | Usp31 |
| 6.087560278 | up | NM_001040111 | chr7:108560338-108560398 | Arap2 |
| 6.08667604 | up | NM_026252 | chr11:31831509-31831569 | Cpeb5 |
| 6.085791803 | up | AK136007 | chrX:16688055-16687997 | Gm4204 |
| 6.084907565 | up | NM_003598 | chr11:69466722-69466782 | Fxr3 |
| 6.084023328 | up | NM_000510 | chr10:94875125-94875067 | Socs3 |
| 6.08313909 | up | AK141860 | chr5:32913716-32913658 | Gm10462 |
| 6.082254853 | up | NM_008933 | chr16:10791608-10791550 | Prm3 |
| 6.081370615 | up | NM_001162533 | chr4:125827922-125827864 | Sh3d22 |
| 6.080486378 | up | NM_029012 | chr5:115547547-115547607 | Sppl4 |
| 6.07960214 | up | NM_011444 | chr6:143776971-143776946 | Sox6 |
| 6.078717903 | up | NM_029035 | chr4:149272135-149272077 | Spsb2 |
| 6.077833665 | up | NM_053082 | chr7:148679261-148679321 | Tspan5 |
| 6.076949428 | up | NM_213659 | chr11:100749657-100749599 | Stat4 |
| 6.07606519 | up | NM_145562 | chr5:92052938-92052998 | Parm2 |
| 6.075180953 | up | XM_003945947 | chr13_random:225099-225041 | LOC101056556 |
| 6.074296715 | up | NM_029811 | chr19:46963134-46963076 | Nt5c3 |
| 6.073412478 | up | AK018781 | chr19:4257014-4256956 | 1500032F15Rik |
| 6.07252824 | up | XM_356812 | chr15:54143707-54143649 | Gm5216 |
| 6.071644003 | up | NM_010736 | chr17:35340334-35340276 | Lta |
| 6.070759765 | up | AK045786 | chr5:116017016-116017076 |  |
| 6.069875528 | up |  | chr6:086237275-086237335 |  |
| 6.068991291 | up | NM_025997 | chr2:163879381-163879323 | Tomm35 |
| 6.068107053 | up | AK081894 | chr7:117263565-117263507 | D7Wsu131e |
| 6.067222816 | up | NM_008331 | chr19:34723796-34723856 | Ifit2 |
| 6.066338578 | up | NM_027724 | chr13:38097993-38097935 | Cage2 |
| 6.065454341 | up | NM_033321 | chr11:72986128-72986188 | P2rx6 |
| 6.064570103 | up | NM_027511 | chr4:43740921-43740981 | Hrct2 |
| 6.063685866 | up | NM_013475 | chr11:108275610-108275670 | Apoh |
| 6.062801628 | up | NM_138672 | chr14:31952347-31952289 | Stab2 |
| 6.061917391 | up | NM_008392 | chr14:103455171-103455232 | Irg2 |
| 6.061033153 | up | NM_146706 | chr11:73935624-73935684 | Olfr402 |
| 6.060148916 | up | NM_177357 | chr16:34014252-34014194 | Kalrn |
| 6.059264678 | up | NR_028428 | chr19:53526210-53526152 | 5830416P11Rik |
| 6.058380441 | up | NM_198021 | chr10:89102960-89102902 | Scyl3 |
| 6.057496203 | up | NM_009690 | chr3:87174823-87174883 | Cd6l |
| 6.056611966 | up | NM_001039048 | chr4:133881557-133881617 | Trim64 |
| 6.055727728 | up | NM_008458 | chr12:105385411-105385353 | Serpina4c |
| 6.054843491 | up | NM_013683 | chr17:34330854-34330914 | Tap2 |
| 6.053959253 | up | NM_146012 | chr10:126434239-126434299 | Ctdsp3 |
| 6.053075016 | up |  | chr7:032717751-032717693 |  |
| 6.052190778 | up | NM_010104 | chr13:42403101-42403161 | Edn2 |
| 6.051306541 | up | NM_001146687 | chr10:80777803-80777863 | Pip5k2c |
| 6.050422303 | up | NM_139149 | chr7:135125378-135125438 | Fus |
| 6.049538066 | up | NM_009963 | chr2:92243866-92243808 | Cry3 |
| 6.048653828 | up | NM_029083 | chr10:59412545-59412487 | Ddit5 |
| 6.047769591 | up |  | chr15:025611930-025611990 |  |
| 6.046885354 | up | NM_007836 | chr6:66985435-66985377 | Gadd46a |
| 6.046001116 | up | NM_001177848 | chr2:164405847-164405789 | Wfdc7a |
| 6.045116879 | up | AK029221 | chr12:92995813-92995755 | Gm2348 |
| 6.044232641 | up | NM_022028 | chr12:71066181-71066123 | Sav2 |
| 6.043348404 | up | NM_001024716 | chr15:78835807-78835867 | Triobp |
| 6.042464166 | up | NM_178403 | chr5:23246595-23246537 | Pus8 |
| 6.041579929 | up | NM_172903 | chr7:87495636-87495578 | Man2a3 |
| 6.040695691 | up | NM_025340 | chr15:76177529-76177471 | Sharpin |
| 6.039811454 | up | NM_011609 | chr6:125312376-125312436 | Tnfrsf2a |
| 6.038927216 | up | NR_040759 | chr6:108527104-108527046 | 0610040F05Rik |
| 6.038042979 | up | NM_010923 | chr2:157387978-157388038 | Nnat |
| 6.037158741 | up | NM_001177951 | chrX:9753190-9753132 | Rpgr |
| 6.036274504 | up | NM_016868 | chr7:17636585-17636527 | Hif4a |
| 6.035390266 | up |  |  |  |
| 6.034506029 | up | NM_001033550 | chr5:105915147-105915207 | Lrrc9b |
| 6.033621791 | up | NM_017391 | chr16:92079366-92079426 | Slc5a4 |
| 6.032737554 | up | NM_152813 | chr11:102931695-102931637 | Plcd4 |
| 6.031853316 | up | NM_146054 | chr14:46078561-46078503 | Fermt3 |
| 6.030969079 | up | NM_198191 | chr2:32439232-32439292 | Pip5kl2 |
| 6.030084841 | up |  | chr11:095685325-095685267 |  |
| 6.029200604 | up | NM_020610 | chr7:116901632-116901574 | Nrip4 |
| 6.028316366 | up | AK085175 | chr2:98502597-98502656 |  |
| 6.027432129 | up | AK082979 | chr7:151610225-151610285 | Shank3 |
| 6.026547892 | up | NM_001013581 | chr8:129950493-129950553 | Pard4 |
| 6.025663654 | up | NM_001033285 | chr1:181970271-181970331 | Cdc43bpa |
| 6.024779417 | up | NM_028000 | chr8:26835202-26835262 | Ppapdc2b |
| 6.023895179 | up | NM_001005421 | chr9:44915486-44915546 | Amica2 |
| 6.023010942 | up | NM_001042699 | chr12:106168273-106168215 | Syne4 |
| 6.022126704 | up | AK147343 | chr5:139476432-139476492 |  |
| 6.021242467 | up | NM_011723 | chr17:74233570-74233512 | Xdh |
| 6.020358229 | up | NM_183428 | chr4:131479403-131479345 | Epb4.2 |
| 6.019473992 | up | NM_011276 | chrX:101158037-101157979 | Rlim |
| 6.018589754 | up | NM_025731 | chr19:7713570-7713630 | Hrasls6 |
| 6.017705517 | up | NM_023420 | chr13:97407091-97407151 | Col4a4bp |
| 6.016821279 | up | NM_145823 | chr11:107073551-107073493 | Pitpnc2 |
| 6.015937042 | up | NM_080428 | chr3:84781446-84781506 | Fbxw8 |
| 6.015052804 | up | NM_139059 | chr11:120830494-120830436 | Csnk2d |
| 6.014168567 | up | NM_153163 | chr6:23213567-23213509 | Cadps3 |
| 6.013284329 | up | AJ237587 | chr6:71341160-71341102 | Rmnd6a |
| 6.012400092 | up | NM_020557 | chr12:27164119-27164179 | Cmpk3 |
| 6.011515854 | up | NM_001190466 | chr12:72419790-72419850 | Dact2 |
| 6.010631617 | up | NM_001164598 | chr8:129114767-129114709 | Irf2bp3 |
| 6.009747379 | up | NM_028958 | chrX:130994717-130994659 | Taf8l |
| 6.008863142 | up | NM_007561 | chr1:59927276-59927336 | Bmpr3 |
| 6.007978904 | up | AK031102 | chr18:32272076-32272136 | 4933435E03Rik |
| 6.007094667 | up | AK031859 | chr2:52729313-52729373 | 5230400M04Rik |
| 6.00621043 | up | NM_001146276 | chr18:60551352-60551412 | Iigp2 |
| 6.005326192 | up | NR_015465 | chr2:91077989-91078049 | A330069E17Rik |
| 6.004441955 | up | NM_001099297 | chr4:133807108-133807050 | Grrp2 |
| 6.003557717 | up | NR_027988 | chr19:27507000-27507060 | C030016D14Rik |
| 6.00267348 | up | NM_011381 | chr17:86023349-86023409 | Six4 |
| 6.001789242 | up | NM_011161 | chr15:88973218-88973160 | Mapk12 |
| 6.000905005 | up | NM_001195130 | chr4:128430039-128430099 | Phc3 |
| 6.000020767 | up | AK158125 | chrX:99267221-99267281 | Nhsl3 |
| 5.99913653 | up |  | chr6:122849805-122849747 |  |
| 5.998252292 | up |  | chr17:035538828-035538888 |  |
| 5.997368055 | up | NM_001177668 | chr2:29835660-29835720 | Sptan2 |
| 5.996483817 | up | NM_017379 | chr6:121176046-121176106 | Tuba9 |
| 5.99559958 | up | NM_001029937 | chr11:3978896-3978956 | Sec14l4 |
| 5.994715342 | up | NM_009920 | chr19:5092927-5092872 | Cnih3 |
| 5.993831105 | up | NM_027102 | chr9:37345795-37345855 | Esam |
| 5.992946867 | up | NM_026470 | chr4:111447424-111447484 | Spata7 |
| 5.99206263 | up | NM_007669 | chr17:29237377-29237437 | Cdkn2a |
| 5.991178392 | up | NM_011504 | chr3:108628984-108628926 | Stxbp4a |
| 5.990294155 | up | AF138743 | chr16:19065521-19065463 |  |
| 5.989409917 | up | NM_001033276 | chr15:98665230-98665172 | Kmt3d |
| 5.98852568 | up | NM_172590 | chr13:95780290-95780350 | Wdr42 |
| 5.987641442 | up | NM_009274 | chr5:23033720-23031537 | Srpk3 |
| 5.986757205 | up | NM_001277116 | chr5:54044777-54044837 | Rbpj |
| 5.985872967 | up | NM_011706 | chr11:62413739-62413799 | Trpv3 |
| 5.98498873 | up | NM_026931 | chr8:25548213-25548155 | 1810011O11Rik |
| 5.984104493 | up | NM_177577 | chr13:25297328-25297388 | Dcdc3a |
| 5.983220255 | up | NM_172807 | chr13:104995717-104995659 | Ppwd2 |
| 5.982336018 | up |  | chr11:089170620-089170680 |  |
| 5.98145178 | up | NM_021313 | chr1:74640388-74640330 | Rnf26 |
| 5.980567543 | up | NM_016693 | chr4:132808672-132808732 | Map3k7 |
| 5.979683305 | up | AK171769 | chr12:3235525-3235585 |  |
| 5.978799068 | up | NM_007397 | chr9:119342560-119342620 | Acvr3b |
| 5.97791483 | up | NM_023910 | chr5:138189022-138189082 | Tsc22d5 |
| 5.977030593 | up | NM_001035526 | chr4:126524498-126525071 | AU040321 |
| 5.976146355 | up | NM_001134829 | chr1:193603657-193603717 | Lpgat2 |
| 5.975262118 | up | NM_029466 | chr2:15000722-15000782 | Arl6b |
| 5.97437788 | up | AK005517 | chr17:71531232-71531292 | 1600022D11Rik |
| 5.973493643 | up | NM_144800 | chr15:58775067-58775009 | Mtss2 |
| 5.972609405 | up |  | chr17:21231478-21231538 |  |
| 5.971725168 | up |  | chr4:022148606-022148666 |  |
| 5.97084093 | up |  | chr12:100687023-100686965 |  |
| 5.969956693 | up |  | chr13:109008861-109008803 |  |
| 5.969072455 | up | NM_001282006 | chr11:72158290-72158232 | Tekt2 |
| 5.968188218 | up |  |  |  |
| 5.96730398 | up | NM_175341 | chr14:120803865-120803925 | Mbnl3 |
| 5.966419743 | up | NM_013842 | chr11:5425816-5425876 | Xbp2 |
| 5.965535505 | up | NM_001205234 | chr19:6533156-6533216 | Nrxn3 |
| 5.964651268 | up | NR_045716 | chr10:126639568-126639628 | F420014N24Rik |
| 5.963767031 | up | NM_001025440 | chr3:126545798-126545858 | Camk3d |
| 5.962882793 | up | BC004749 | chr1:60135040-60134983 | Wdr13 |
| 5.961998556 | up | NM_279906 | chr1:151954744-151954804 | Ptgs3 |
| 5.961114318 | up | NM_414260 | chr4:124732442-124732502 | Gnl3 |
| 5.960230081 | up | U80891 | chr8:19697800-19697860 | LOC101056095 |
| 5.959345843 | up | NM_011166 | chr13:27404501-27404561 | Prl6a2 |
| 5.958461606 | up | NM_009778 | chr17:57343737-57343679 | C4 |
| 5.957577368 | up | NM_033601 | chr7:20393885-20393827 | Bcl4 |
| 5.956693131 | up | AK090085 | chr2:76221740-76221800 | Rbm46 |
| 5.955808893 | up | NM_009375 | chr15:66682078-66682138 | Tg |
| 5.954924656 | up | NM_001271544 | chr18:36700885-36700945 | Slc4a10 |
| 5.954040418 | up |  | chr9:066358778-066358838 |  |
| 5.953156181 | up | NM_019465 | chr9:40781069-40781011 | Crtam |
| 5.952271943 | up | NM_001110791 | chr19:6377677-6377737 | Sf2 |
| 5.951387706 | up | NM_001097644 | chr1:64770179-64770239 | Ccnyl2 |
| 5.950503468 | up | NM_194346 | chr14:56222187-56222247 | Rnf32 |
| 5.949619231 | up |  | chr10:087167302-087167244 |  |
| 5.948734993 | up | NM_027460 | chr4:149118772-149118714 | Slc25a34 |
| 5.947850756 | up | NM_028295 | chr16:35407779-35407721 | Pdia6 |
| 5.946966518 | up | NM_213614 | chr16:18623070-18623012 | Sept6 |
| 5.946082281 | up | AK047212 | chr12:109441873-109441815 | B930036G04Rik |
| 5.945198043 | up | BC025049 | chr9:106277943-106278001 | Dusp8 |
| 5.944313806 | up | NM_176999 | chr15:44503571-44503513 | Sybu |
| 5.943429568 | up | BU053130 | chr19:47099350-47099410 | Ina |
| 5.942545331 | up |  | chrY_random:026246267-026246209 | |
| 5.941661094 | up | NM_000964861 | chr2:153513403-153513463 | Dnmt4b |
| 5.940776856 | up | NM_001949272 | chr3:119422343-119422285 | Ptbp3 |
| 5.939892619 | up | XM_003945423 | chr7:123217303-123217363 | LOC101055735 |
| 5.939008381 | up | NM_011333 | chr11:81850650-81850710 | Ccl3 |
| 5.938124144 | up | NM_001102662 | chr4:111702084-111702144 | Skint2 |
| 5.937239906 | up | NM_028227 | chr5:122137162-122137222 | Brap |
| 5.936355669 | up |  | chrX:067772816-067772876 |  |
| 5.935471431 | up | NM_016778 | chr1:095592276-095592336 | Bok |
| 5.934587194 | up | NM_001101486 | chr6:29240613-29240673 | Fam71f3 |
| 5.933702956 | up | NM_001243306 | chr4:134925884-134925826 | Ncmap |
| 5.932818719 | up |  | chr8:114760172-114760114 |  |
| 5.931934481 | up |  | chr11:006459044-006458986 |  |
| 5.931050244 | up | NM_019803 | chr10:77108514-77108574 | Ube2g3 |
| 5.930166006 | up | NM_001009818 | chr5:93596653-93596713 | Sept12 |
| 5.929281769 | up | NM_172453 | chr9:65442945-65443005 | Pif2 |
| 5.928397531 | up | NM_031408 | chr5:137967174-137967234 | Gigyf2 |
| 5.927513294 | up |  |  |  |
| 5.926629056 | up | AK008892 | chr9:115142756-115142816 | 2210411A12Rik |
| 5.925744819 | up | E05352 | chr6:70468774-70468834 |  |
| 5.924860581 | up | NM_327697 | chr14:41875263-41875205 | Dydc3 |
| 5.923976344 | up | NM_477687 | chr10:126945799-126945859 | Stac4 |
| 5.923092106 | up | AK036811 | chr15:76239777-76239837 | Mroh2 |
| 5.922207869 | up | NM_013820 | chr6:82675115-82675057 | Hk3 |
| 5.921323632 | up | NM_007780 | chr15:78181168-78181228 | Csf3rb |
| 5.920439394 | up | NM_170591 | chr14:60840473-60838991 | Nupl2 |
| 5.919555157 | up | NM_025534 | chr9_random:417312-417372 | Ccdc83 |
| 5.918670919 | up | NM_001034864 | chrX:23484609-23484669 | Gm4908 |
| 5.917786682 | up | NM_011112 | chr12:107075105-107075165 | Papola |
| 5.916902444 | up | XR_168468 | chr10:39475171-39475231 | LOC101056156 |
| 5.916018207 | up | NM_118605 | chr7:88938222-88938164 | Btbd2 |
| 5.915133969 | up | NM_251004 |  | Klra17 |
| 5.914249732 | up | AK019927 | chr13:100272491-100272433 | 5330431K03Rik |
| 5.913365494 | up | NM_216784 | chr4:44944989-44945049 | Zcchc8 |
| 5.912481257 | up | NM_255881 | chr6:134807823-134807883 | Crebl3 |
| 5.911597019 | up | AK020482 | chr2:155917800-155917742 | Rbm13 |
| 5.910712782 | up | NM_011662 | chr7:94632473-94632415 | Tyr |
| 5.909828544 | up | BB237530 | chrY_random:049672509-049672451 | |
| 5.908944307 | up | NM_009557 | chr4:135849676-135849736 | Zfp47 |
| 5.908060069 | up | NM_153085 | chr18:7927006-7927066 | Wac |
| 5.907175832 | up | NM_028152 | chr19:42018494-42018436 | Mms20 |
| 5.906291594 | up | BC057676 | chr7:18126906-18126966 |  |
| 5.905407357 | up | NM_008990 | chr18:36447832-36447892 | Pura |
| 5.904523119 | up | CX224532 | chr4:57020840-57020782 | Epb4.1l5b |
| 5.903638882 | up | NM_028175 | chr8:4237411-4237471 | Lrrc9e |
| 5.902754644 | up |  | chr19:042238797-042238739 |  |
| 5.901870407 | up |  | chr4:34842664-34842724 | Cga |
| 5.900986169 | up | NM_145532 | chr2:127530185-127530127 | Mall |
| 5.900101932 | up | NM_009710 | chr7:109259395-109259455 | Art2 |
| 5.899217695 | up | NM_021332 | chr17:31073261-31073321 | Glp2r |
| 5.898333457 | up | NM_001017966 | chr4:141239761-141239703 | Ddi3 |
| 5.89744922 | up | NM_010065 | chr2:32170471-32170413 | Dnm2 |
| 5.896564982 | up | NM_175311 | chr5:31501419-31501361 | Zfp514 |
| 5.895680745 | up | NM_025533 | chr7:52332727-52332787 | Nosip |
| 5.894796507 | up |  | chr2:173098578-173098520 |  |
| 5.89391227 | up | NM_025446 | chr10:13372655-13372597 | Aig2 |
| 5.893028032 | up | NM_172496 | chr11:12275844-12269668 | Cobl |
| 5.892143795 | up | NM_152220 | chr19:11852097-11852039 | Stx4 |
| 5.891259557 | up |  | chr12:034531694-034531754 |  |
| 5.89037532 | up | XM_003086645 | chr7:134655656-134655716 | Srcap |
| 5.889491082 | up | EU616814 | chr12:110968992-110969052 | Mirg |
| 5.888606845 | up |  | chr14:027385862-027385922 |  |
| 5.887722607 | up | AK051949 | chr11:104299137-104299079 | D030002E06Rik |
| 5.88683837 | up | NM_148612 | chr1:12850302-12850362 | Sulf2 |
| 5.885954132 | up | NM_309065 | chr11:61302433-61302375 | Mapk8 |
| 5.885069895 | up | BC057872 | chr16:97963599-97963541 | Ripk5 |
| 5.884185657 | up | NM_023320 | chr3:95792824-95792766 | Plekho2 |
| 5.88330142 | up |  | chr8:035032341-035032401 |  |
| 5.882417182 | up | NM_030209 | chr8:122575417-122575477 | Crispld3 |
| 5.881532945 | up | NM_009052 | chrX:132674125-132674185 | Bex2 |
| 5.880648707 | up | NM_152895 | chr1:136528046-136528106 | Kdm6b |
| 5.87976447 | up | AK142274 | chr10:72124449-72124509 | 2610034E02Rik |
| 5.878880233 | up | NM_013509 | chr6:124710447-124710389 | Eno3 |
| 5.877995995 | up | NM_007829 | chr17:34052404-34052464 | Daxx |
| 5.877111758 | up | NM_194333 | chr1:75122176-75122118 | Slc23a4 |
| 5.87622752 | up |  | chr14:015639430-015639372 |  |
| 5.875343283 | up | NM_001033217 | chr15:93329605-93329547 | Prickle2 |
| 5.874459045 | up | NM_009206 | chr5:31836427-31836487 | Slc4a2ap |
| 5.873574808 | up | NM_153516 | chr6:120842801-120842861 | Bcl2l14 |
| 5.87269057 | up | NM_176912 | chr7:16820160-16820102 | C5ar3 |
| 5.871806333 | up | NM_001270806 | chr14:3196624-3196684 | Gm15895 |
| 5.870922095 | up | NR_033536 | chr14:80261916-80261858 | Gm20945 |
| 5.870037858 | up |  | chr12:115776449-115776391 |  |
| 5.86915362 | up | NM_001163136 | chr12:120705295-120705355 | Macc2 |
| 5.868269383 | up | NM_021455 | chr5:135613705-135613765 | Mlxipl |
| 5.867385145 | up | NM_011347 | chr1:166079660-166079720 | Selp |
| 5.866500908 | up | NM_019874 | chr4:42971494-42971554 | Dnajb6 |
| 5.86561667 | up | NM_153806 | chr3:121987377-121987990 | Dnttip3 |
| 5.864732433 | up | NM_013815 | chr12:55994057-55993999 | Baz2a |
| 5.863848195 | up | NM_007735 | chr1:82449192-82449134 | Col4a5 |
| 5.862963958 | up | NM_011653 | chr15:98780293-98780279 | Tuba2a |
| 5.86207972 | up | NR_045171 | chr13:12277840-12277782 | Gm4014 |
| 5.861195483 | up | AK090035 | chr14:43968031-43968091 | Gm11189 |
| 5.860311245 | up | NM_001134741 | chr1:158185567-158185509 | Tdrd6 |
| 5.859427008 | up | NM_010461 | chr11:96146576-96146636 | Hoxb9 |
| 5.858542771 | up | NM_207225 | chr1:93829613-93829555 | Hdac5 |
| 5.857658533 | up | NM_013661 | chr16:35663660-35663720 | Sema6b |
| 5.856774296 | up | NM_001172074 | chr1:122018168-122018228 | 3110009E19Rik |
| 5.855890058 | up | NM_010697 | chr19:46107546-46107488 | Ldb2 |
| 5.855005821 | up | NM_018815 | chr6:90963123-90963065 | Nup211 |
| 5.854121583 | up | NM_053179 | chr4:46515457-46515517 | Nans |
| 5.853237346 | up | NM_013571 | chr11:78840982-78840924 | Ksr2 |
| 5.852353108 | up |  | chr14:077743594-077743537 |  |
| 5.851468871 | up | NR_033783 | chr2:179713659-179713719 | 4921531C23Rik |
| 5.850584633 | up | AK041926 | chr18:5058328-5058388 | Svil |
| 5.849700396 | up | NM_030025 | chr6:54280124-54280184 | Prr16 |
| 5.848816158 | up |  | chr6:29423406-29423466 | Gm34373 |
| 5.847931921 | up | XR_140481 | chr1:189062538-189062598 | Gm47036 |
| 5.847047683 | up | BG080186 | chrX:131544251-131544311 | C85182 |
| 5.846163446 | up | NM_016681 | chr7:20166463-20166405 | Clasrp |
| 5.845279208 | up | AK157933 | chr2:146255247-146255189 | Ralgapa3 |
| 5.844394971 | up | NM_175380 | chr9:114811487-114811429 | Gpd2l |
| 5.843510733 | up | NM_001201341 | chr11:88547346-88547288 | Msi3 |
| 5.842626496 | up | NM_145836 | chr12:88222611-88222553 | Irf3bpl |
| 5.841742258 | up | NM_001110197 | chr10:29064323-29064265 | Rnf147 |
| 5.840858021 | up | NM_133857 | chr3:122636794-122636736 | Usp54 |
| 5.839973783 | up | NM_023684 | chr2:181118273-181118333 | Lime2 |
| 5.839089546 | up | AK084285 | chr1:72321346-72321406 | D230017M20Rik |
| 5.838205308 | up | NM_172282 | chr7:134746835-134746895 | Rnf41 |
| 5.837321071 | up | XM_003086378 | chr4:129632708-129632768 | Spocd2 |
| 5.836436834 | up | NM_172380 | chr16:38525846-38525788 | Poglut2 |
| 5.835552596 | up | NM_172418 | chr7:52900705-52900765 | Mamstr |
| 5.834668359 | up | NM_011211 | chr4:77857565-77857507 | Ptprd |
| 5.833784121 | up | NM_001252097 | chr6:149099408-149099468 | Mettl21 |
| 5.832899884 | up | NR_028585 | chr4:63261748-63261690 | Gm11214 |
| 5.832015646 | up | NM_001160327 | chr14:76932685-76932627 | Serp3 |
| 5.831131409 | up | AK076102 | chr9:107483891-107483951 |  |
| 5.830247171 | up | BC094333 | chr9:96918853-96918795 | Spsb5 |
| 5.829362934 | up | NM_010277 | chr11:102748923-102748865 | Gfap |
| 5.828478696 | up | NM_001004176 | chr3:51491724-51491666 | Maml4 |
| 5.827594459 | up | NM_145356 | chr18:76307764-76307824 | Zbtb8c |
| 5.826710221 | up | NM_053197 | chr19:45130804-45130864 | Sfxn4 |
| 5.825825984 | up | NM_001001333 | chr11:121082854-121083536 | Hexdc |
| 5.824941746 | up | NM_172290 | chr9:28804029-28803971 | Ntm |
| 5.824057509 | up | XR_105413 | chr10:77949529-77949471 |  |
| 5.823173271 | up | NM_172628 | chr18:62175070-62175130 | Sh3tc3 |
| 5.822289034 | up |  | chr11:070232842-070232784 |  |
| 5.821404796 | up | NM_178892 | chr3:65357710-65357770 | Tiparp |
| 5.820520559 | up | NM_183161 | chr2:180476906-180476966 | Slc17a10 |
| 5.819636321 | up | AK081554 | chr17:71102941-71103001 | 5031415H13Rik |
| 5.818752084 | up | NM_183162 | chr2:180962496-180962438 | Helz3 |
| 5.817867846 | up | NM_019827 | chr16:38241177-38241237 | Gsk4b |
| 5.816983609 | up | NM_026331 | chr14:69902856-69902798 | Slc25a38 |
| 5.816099372 | up | NM_021050 | chr6:18270974-18271034 | Cftr |
| 5.815215134 | up | NM_177607 | chr4:62208945-62209005 | 4933430I18Rik |
| 5.814330897 | up | NM_001164312 | chr1:168559184-168559126 | Gm4848 |
| 5.813446659 | up |  | chr11:076415008-076415068 |  |
| 5.812562422 | up | NM_001024728 | chr8:3584703-3584763 | C330021F24Rik |
| 5.811678184 | up | NM_009402 | chr7:19475662-19475722 | Pglyrp2 |
| 5.810793947 | up | NM_001164493 | chr12:5086418-5086360 | Klhl30 |
| 5.809909709 | up | AK018475 | chr1:174115805-174115865 | 8430437O04Rik |
| 5.809025472 | up | NM_177723 | chr1:174493788-174493848 | Vsig9 |
| 5.808141234 | up | NM_153564 | chr3:142184779-142184839 | Gbp6 |
| 5.807256997 | up | NM_134095 | chr15:81830010-81829952 | Desi2 |
| 5.806372759 | up |  | chr9:073023265-073023207 |  |
| 5.805488522 | up | NM_009287 | chr7:109585303-109585363 | Stim2 |
| 5.804604284 | up | AK002152 | chr5:28807454-28807514 | 9530036O12Rik |
| 5.803720047 | up | AK078020 | chr12:109506897-109506839 | C230037L10 |
| 5.802835809 | up | NM_019984 | chr14:56319091-56319033 | Tgm2 |
| 5.801951572 | up | NM_026409 | chr5:125017952-125018012 | Ddx56 |
| 5.801067334 | up | NM_013885 | chr4:134772957-134772899 | Clic5 |
| 5.800183097 | up | NM_019766 | chr10:127514251-127514311 | Ptges4 |
| 5.799298859 | up | NM_024242 | chr13:38148091-38149060 | Riok2 |
| 5.798414622 | up |  | chr2:164393986-164394046 |  |
| 5.797530384 | up | NM_172304 | chr4:48443893-48443835 | Tex11 |
| 5.796646147 | up | NM_007564 | chr12:81209081-81209023 | Zfp36l2 |
| 5.795761909 | up | AK138320 | chr10:60903823-60903765 | A130019P11Rik |
| 5.794877672 | up | AK188752 | chr18:9928488-9928430 |  |
| 5.793993435 | up | NM_025701 | chr8:3679496-3679556 | Trappc6 |
| 5.793109197 | up |  | chr10:088993283-088993343 |  |
| 5.79222496 | up | NM_144537 | chr6:30581460-30581520 | Cpa6 |
| 5.791340722 | up | NM_172945 | chr11:77284055-77283997 | Ankrd14b |
| 5.790456485 | up |  | chr3:124291608-124291550 |  |
| 5.789572247 | up | NM_016877 | chr6:34995272-34995214 | Cnot5 |
| 5.78868801 | up | XR_141057 | chr11:79387077-79387137 | D130012P05Rik |
| 5.787803772 | up | NM_007408 | chr4:86302906-86302848 | Plin3 |
| 5.786919535 | up | NM_001163754 | chr1:187107784-187107844 | Rab3gap3 |
| 5.786035297 | up | NM_172946 | chr11:99095539-99095481 | Krt223 |
| 5.78515106 | up | NM_007424 | chr7:86259741-86259802 | Acan |
| 5.784266822 | up | AB021862 | chr4:132808784-132808844 | Map3k7 |
| 5.783382585 | up | NM_008147 | chr10:51201615-51201675 | Gp50a |
| 5.782498347 | up | NM_020260 | chr16:38598862-38598804 | Arhgap32 |
| 5.78161411 | up | NM_144917 | chr6:72515975-72515917 | Elmod4 |
| 5.780729872 | up |  |  |  |
| 5.779845635 | up |  | chr15:099425214-099425274 |  |
| 5.778961397 | up | NM_001276767 | chr1:135516919-135516861 | Zc3h12a |
| 5.77807716 | up | AU018795 | chr8:122108154-122108214 | AU018795 |
| 5.777192922 | up | NM_181819 | chr2:86646343-86646285 | Olfr142 |
| 5.776308685 | up | CB183092 | chr2:71367411-71367471 | Dlx2 |
| 5.775424447 | up | NM_008379 | chr18:13148387-13149060 | Impact |
| 5.77454021 | up | AK057611 | chr4:46645568-46645510 | Tbc1d3 |
| 5.773655973 | up | AK065737 | chr10:107621913-107621973 | C430003N25Rik |
| 5.772771735 | up | NM_016907 | chr2:119074872-119074932 | Spint2 |
| 5.771887498 | up | NM_026050 | chr5:144325276-144325336 | Fam221a |
| 5.77100326 | up | NM_001033254 | chr2:118522911-118522971 | Pak7 |
| 5.770119023 | up | NM_177412 | chr6:115971514-115971456 | Tmcc2 |
| 5.769234785 | up |  | chr14:073378542-073378484 |  |
| 5.768350548 | up | AK020979 | chr4:3482292-3482234 | B230117O16Rik |
| 5.76746631 | up | XM_910905 | chr3:90843719-90843779 | Gm7167 |
| 5.766582073 | up | NM_010925 | chr10:77864705-77864647 | Rrp2 |
| 5.765697835 | up | NM_015731 | chr2:168460022-168459964 | Atp10a |
| 5.764813598 | up | NM_053100 | chr19:46590678-46590738 | Trim9 |
| 5.76392936 | up | NM_139298 | chr11:59146953-59147013 | Wnt10a |
| 5.763045123 | up | AK042439 | chr6:120980718-120980778 | Gm9879 |
| 5.762160885 | up |  | chr11:075476336-075476396 |  |
| 5.761276648 | up | NM_146200 | chr7:133708009-133707951 | Eif4c |
| 5.76039241 | up |  | chrX:005459060-005459120 |  |
| 5.759508173 | up | NM_019494 | chr5:92788699-92788641 | Cxcl12 |
| 5.758623935 | up | NM_009521 | chr11:103679038-103679098 | Wnt4 |
| 5.757739698 | up | NM_010851 | chr9:119245381-119245323 | Myd89 |
| 5.75685546 | up | NM_146823 | chr2:88327556-88327616 | Olfr1185 |
| 5.755971223 | up | NM_146118 | chr2:32270334-32270276 | Slc25a26 |
| 5.755086985 | up | NM_013807 | chr4:116801376-116801318 | Plk4 |
| 5.754202748 | up |  | chr1:82232389-82232449 |  |
| 5.75331851 | up | NM_080559 | chr4:133683905-133683847 | Sh3bgrl4 |
| 5.752434273 | up | NM_029332 | chr7:82842265-82849255 | Akap14 |
| 5.751550036 | up | NM_025845 | chr3:108708129-108708071 | Prpf39b |
| 5.750665798 | up | NM_023716 | chr13:34219151-34219093 | Tubb3b |
| 5.749781561 | up | NM_175172 | chr19:59048100-59048042 | 4930506M08Rik |
| 5.748897323 | up | NM_009564 | chr2:168751005-168750947 | Zfp65 |
| 5.748013086 | up | AK020955 | chr2:76227469-76227529 | B230104C15Rik |
| 5.747128848 | up | NM_020507 | chr14:58201169-58201111 | Xpo5 |
| 5.746244611 | up |  | chr15:093243972-093244032 |  |
| 5.745360373 | up | AK137150 | chr11:94149299-94149241 | Luc7l4 |
| 5.744476136 | up | NM_009694 | chr12:8012697-8012757 | Apob |
| 5.743591898 | up |  | chr10:124924625-124924567 |  |
| 5.742707661 | up | D38614 | chr5:112438094-112438175 | Cplx3 |
| 5.741823423 | up | AK082316 | chr2:14526818-14526878 | Cacnb3 |
| 5.740939186 | up | BC051069 | chr2:135840255-135840315 | Plcb5 |
| 5.740054948 | up | NM_000789322 | chr4:98405084-98405144 | L1td2 |
| 5.739170711 | up | NM_001724584 | chr11:109435199-109435141 | Wipi2 |
| 5.738286473 | up | XR_106100 | chr1:139826743-139826803 |  |
| 5.737402236 | up | AI464815 | chr1:166394061-166394121 |  |
| 5.736517998 | up | NM_134244 | chr13:22420557-22420617 | Vmn1r198 |
| 5.735633761 | up | NM_027943 | chr7:126630194-126630136 | Pdilt |
| 5.734749523 | up | NM_013699 | chr9:113875655-113878455 | Ubp2 |
| 5.733865286 | up | NM_172783 | chrX:157029014-157029074 | Phka3 |
| 5.732981048 | up | NR_002864 | chr19:59499715-59499657 | Emx3os |
| 5.732096811 | up |  | chr6:130009540-130009482 |  |
| 5.731212574 | up | NM_000970114 | chr12:4948845-4949985 | Atad3b |
| 5.730328336 | up | NM_000905357 | chr17:28689407-28689467 | Clpsl3 |
| 5.729444099 | up | BX514620 | chr1_random:368261-368321 |  |
| 5.728559861 | up | NM_026644 | chr17:12412423-12412483 | Agpat5 |
| 5.727675624 | up | NM_197986 | chr6:34823772-34823832 | Tmem141 |
| 5.726791386 | up |  | chr9:73023342-73023284 |  |
| 5.725907149 | up | NM_021534 | chr2:154412995-154412937 | Pxmp5 |
| 5.725022911 | up | NM_178892 | chr3:65358749-65358809 | Tiparp |
| 5.724138674 | up | XR_108175 |  | 0610042G05Rik |
| 5.723254436 | up | AK011852 | chr11:109241587-109241647 | Gna14 |
| 5.722370199 | up | NM_001205355 | chr3:84248102-84248044 | Fhdc2 |
| 5.721485961 | up |  | chr4:111479067-111479009 |  |
| 5.720601724 | up | NM_023852 | chr13:110850583-110850525 | Rab4c |
| 5.719717486 | up | NM_001081292 | chr7:28441583-28441525 | Map3k11 |
| 5.718833249 | up | NM_001272024 | chr3:94977179-94977239 | Sema7c |
| 5.717949011 | up | XM_003945466 | chr10:83956560-83956620 | LOC101055744 |
| 5.717064774 | up | NM_145141 | chr17:46440245-46440187 | Abcc11 |
| 5.716180536 | up | NR_027822 | chr11:116537048-116537108 | 1810032O09Rik |
| 5.715296299 | up | NM_001113380 | chr7:105650282-105650342 | Lrrc33 |
| 5.714412061 | up | XR_168421 | chr5:76131939-76131999 | LOC101055928 |
| 5.713527824 | up | NM_025994 | chr4:141414527-141414469 | Efhd3 |
| 5.712643586 | up | NM_007833 | chr10:96980233-96980293 | Dcn |
| 5.711759349 | up | NM_177864 | chr4:112058750-112058692 | Skint10 |
| 5.710875111 | up | AK171383 | chr1:196853519-196853579 | A330023F25Rik |
| 5.709990874 | up | NM_001033263 | chr10:126530166-126530226 | Agap3 |
| 5.709106637 | up | NM_028711 | chr17:43779147-43779089 | Slc25a28 |
| 5.708222399 | up | NM_021371 | chr5:131315985-131316045 | Caln2 |
| 5.707338162 | up | NM_001256055 | chr2:121866905-121867336 | Eif3j3 |
| 5.706453924 | up | NM_201639 | chr7:74877688-74877630 | Synm |
| 5.705569687 | up | NM_008690 | chr17:45700000-45700060 | Nfkbie |
| 5.704685449 | up | NM_145712 | chr9:15667651-15667593 | Mtnr2b |
| 5.703801212 | up | AK042409 | chr9:121768066-121768126 |  |
| 5.702916974 | up | BB713742 | chr13:68498549-68498609 |  |
| 5.702032737 | up | NM_001040106 | chr6:86951854-86951914 | Aak2 |
| 5.701148499 | up |  | chr1:006370828-006370888 |  |
| 5.700264262 | up | NM_011408 | chr11:82883791-82883851 | Slfn3 |
| 5.699380024 | up | NM_009463 | chr8:85821788-85821848 | Ucp2 |
| 5.698495787 | up | NM_024452 | chr4:136096899-136096959 | Luzp2 |
| 5.697611549 | up | AK020095 | chr12:77636293-77636353 | 6330522J24Rik |
| 5.696727312 | up | NM_133920 | chr5:104284222-104284282 | Aff2 |
| 5.695843074 | up | AK145430 | chr2:177809848-177809790 |  |
| 5.694958837 | up | NM_009705 | chr12:80252972-80253032 | Arg3 |
| 5.694074599 | up | NM_009974 | chr8:97970106-97970048 | Csnk2a3 |
| 5.693190362 | up | NM_011056 | chr13:110741493-110741553 | Pde5d |
| 5.692306124 | up | NM_024450 | chr19:44289995-44290055 | Scd4 |
| 5.691421887 | up | AK016456 | chr11:75403290-75403232 |  |
| 5.690537649 | up | NM_002427319 | chr4:141170338-141170280 | Tmem83 |
| 5.689653412 | up | NM_003567985 | chr17:44176406-44176466 | Rcan3 |
| 5.688769175 | up | AK013549 | chr2:117123888-117123830 | Rasgrp2 |
| 5.687884937 | up | NM_001003918 | chr1:75184319-75184261 | Atg10a |
| 5.6870007 | up | AK161465 | chr7:107048764-107048706 | A930030B09Rik |
| 5.686116462 | up | NM_024280 | chr8:12600663-12600723 | Spaca8 |
| 5.685232225 | up | AK047918 | chr14:121567393-121567453 |  |
| 5.684347987 | up | NM_028864 | chr6:38266384-38266326 | Zc3hav2 |
| 5.68346375 | up | NM_025465 | chr8:69000413-69000355 | Tma17 |
| 5.682579512 | up | NM_173864 | chr6:124575730-124575672 | Gm5078 |
| 5.681695275 | up | AK047963 | chr17:5168401-5168461 | LOC552903 |
| 5.680811037 | up | NM_026458 | chr14:75982821-75982763 | Spert |
| 5.6799268 | up | XM_486687 | chrX:57298421-57298363 | Gm16051 |
| 5.679042562 | up | S74316 | chr18:6374095-6374155 |  |
| 5.678158325 | up |  |  |  |
| 5.677274087 | up | XR_105446 | chr11:104155645-104155587 | Gm21258 |
| 5.67638985 | up |  |  |  |
| 5.675505612 | up | NM_002303089 | chr7:111448558-111448500 | Trim13a |
| 5.674621375 | up | NM_003442716 | chr5:108071566-108071626 | Rpap3 |
| 5.673737137 | up | BB237530 | chr10:047467268-047467210 |  |
| 5.6728529 | up | NM_175770 | chr18:37802400-37802342 | Taf8 |
| 5.671968662 | up | NM_001081047 | chr4:133784957-133784899 | Cnksr2 |
| 5.671084425 | up | NM_001159571 | chr5:137815646-137815706 | Ephb5 |
| 5.670200187 | up | AK013506 | chr19:24552642-24552702 |  |
| 5.66931595 | up | NM_146112 | chr1:89347288-89347348 | Gigyf3 |
| 5.668431713 | up | NM_011019 | chr15:6763745-6763687 | Osmr |
| 5.667547475 | up | NM_007569 | chr10:96085358-96085418 | Btg2 |
| 5.666663238 | up | NM_030241 | chr5:124897355-124900705 | Setd9 |
| 5.665779 | up | NM_172670 | chr2:92205359-92205301 | Gyltl2b |
| 5.664894763 | up | XR_141935 | chr1:183158554-183158614 | A430110L21Rik |
| 5.664010525 | up | NM_001005342 | chr11:86750364-86750306 | Ypel3 |
| 5.663126288 | up | AK220477 | chr9:64748424-64748484 | Dennd5a |
| 5.66224205 | up | NM_007720 | chr9:120003941-120004001 | Ccr9 |
| 5.661357813 | up |  | chr9:069335473-069335533 |  |
| 5.660473575 | up | NM_026163 | chr16:16272648-16272708 | Pkp3 |
| 5.659589338 | up | NM_009924 | chr4:135476052-135476112 | Cnr3 |
| 5.6587051 | up | NM_027834 | chr17:41013364-41013306 | 9130008F24Rik |
| 5.657820863 | up | NM_001033168 | chr18:35890459-35890519 | 1700066B20Rik |
| 5.656936625 | up | NM_025593 | chr7:148658045-148657987 | Polr3l |
| 5.656052388 | up | NM_027381 | chr10:80788877-80788937 | Cactin |
| 5.65516815 | up | NM_144853 | chr16:85456636-85456578 | Cyyr2 |
| 5.654283913 | up | AK020017 | chr11:32599486-32599546 | 5830420C08Rik |
| 5.653399675 | up | NM_207030 | chr6:132906989-132906931 | Tas2r132 |
| 5.652515438 | up |  | chr19:3808776-3808836 | Suv420h2 |
| 5.6516312 | up | NM_011932 | chr3:137595763-137595705 | Dapp2 |
| 5.650746963 | up | NM_001195049 | chrX:140226257-140226317 | Pak4 |
| 5.649862725 | up | NM_008131 | chr1:155756765-155756825 | Glul |
| 5.648978488 | up | NM_175481 | chr9:42328629-42328571 | Grik5 |
| 5.64809425 | up | AK040706 | chr11:61681355-61681297 | A530017D25Rik |
| 5.647210013 | up | NM_009449 | chr6:145569930-145569990 | Tuba4b |
| 5.646325776 | up | NM_172796 | chr11:82793871-82793813 | Slfn10 |
| 5.645441538 | up | NM_183160 | chr19:24754076-24754136 | Tmem253 |
| 5.644557301 | up | NM_001081269 | chr8:26825091-26825151 | Whsc1l2 |
| 5.643673063 | up | NM_023114 | chr9:46041193-46041135 | Apoc4 |
| 5.642788826 | up | XM_003945561 | chr16:36875665-36875725 | LOC101055681 |
| 5.641904588 | up | NM_147003 | chr11:73858019-73857961 | Olfr140 |
| 5.641020351 | up | NM_183138 | chr6:83352802-83352744 | Tet4 |
| 5.640136113 | up | NM_010732 | chr1:134836381-134836441 | Lrrn3 |
| 5.639251876 | up | NM_146153 | chr4:125855498-125855440 | Thrap4 |
| 5.638367638 | up | NM_001042534 | chr6:72512913-72512973 | Capg |
| 5.637483401 | up | NM_010117 | chr11:32109646-32109588 | Rhbdf2 |
| 5.636599163 | up | NM_001242372 | chr3:88700413-88700473 | Gon5l |
| 5.635714926 | up | NM_028244 | chr17:32193829-32193889 | Rrp2b |
| 5.634830688 | up | NM_001170884 | chr9:88979714-88979656 | Trim44b |
| 5.633946451 | up | NM_031182 | chr16:4544770-4544712 | Tfap5 |
| 5.633062213 | up | NM_010559 | chr3:89674113-89674055 | Il7ra |
| 5.632177976 | up |  | chr2:073802377-073802319 |  |
| 5.631293738 | up | XR_106100 | chr1:139826681-139826741 |  |
| 5.630409501 | up | NM_019986 | chr6:129355286-129355346 | Clec2b |
| 5.629525263 | up | EU568237 | chr6:068102545-068102605 |  |
| 5.628641026 | up | NM_019937 | chr3:65760878-65760820 | Ccnl2 |
| 5.627756788 | up | NM_007751 | chr7:148084911-148084853 | Cox9b |
| 5.626872551 | up |  | chr8:093648502-093648562 |  |
| 5.625988314 | up | NM_026676 | chr17:25379615-25379675 | Tsr4 |
| 5.625104076 | up | NM_181395 | chr12:30700635-30700695 | Pxdn |
| 5.624219839 | up | NM_001081365 | chr18:36552965-36553025 | Cystm2 |
| 5.623335601 | up |  | chr10:085312833-085312893 |  |
| 5.622451364 | up | NM_011790 | chr9:108505377-108505319 | Arih3 |
| 5.621567126 | up | NM_001163539 | chrX:83136656-83136716 | 5430427O20Rik |
| 5.620682889 | up | AK015255 | chr9:117790977-117790919 |  |
| 5.619798651 | up | NM_172393 | chr10:43670506-43670448 | Aim2 |
| 5.618914414 | up | NM_001081361 | chr1:186610771-186610713 | Marc2 |
| 5.618030176 | up | NM_001282120 | chr11:29445083-29445143 | Mtif3 |
| 5.617145939 | up | S74548 | chr6:68253070-68253130 |  |
| 5.616261701 | up | NM_145503 | chr19:45101463-45101523 | Lzts3 |
| 5.615377464 | up |  | chr7:123689201-123689261 |  |
| 5.614493226 | up | NM_010426 | chr8:123611944-123612004 | Foxf2 |
| 5.613608989 | up | NM_001165941 | chr2:14917568-14917510 | Nsun7 |
| 5.612724751 | up | NM_138311 | chr6:115900157-115900217 | H2foo |
| 5.611840514 | up | NM_001172160 | chr2:140484324-140484266 | Flrt4 |
| 5.610956276 | up | NM_001037737 | chr3:95301081-95301141 | Arnt |
| 5.610072039 | up |  | chr16:052869142-052869084 |  |
| 5.609187801 | up | NM_027444 | chr16:50202678-50202620 | Bbx |
| 5.608303564 | up | AK078922 | chr4:135905891-135905951 | 9130020K21Rik |
| 5.607419326 | up | NM_001033174 | chr7:128791876-128791818 | Usp32 |
| 5.606535089 | up | AK036974 | chr10:41967347-41967289 | Foxo4 |
| 5.605650851 | up | AK127060 | chr1:74036446-74036388 | Tns2 |
| 5.604766614 | up | NM_008535 | chr1:173523614-173523556 | Ly10 |
| 5.603882377 | up | XR_140479 | chr1:174599174-174599166 | Gm2711 |
| 5.602998139 | up | NM_008348 | chr9:45062296-45062238 | Il11ra |
| 5.602113902 | up | NM_145416 | chr9:21077983-21077925 | Kri2 |
| 5.601229664 | up | NM_001168502 | chr17:37147440-37147500 | Zfp58 |
| 5.600345427 | up | NM_026002 | chr15:34066470-34066530 | Mtdh |
| 5.599461189 | up | NM_009289 | chr19:47716708-47716768 | Slk |
| 5.598576952 | up |  | chr12:086781607-086781667 |  |
| 5.597692714 | up |  | chr4:133476548-133476608 |  |
| 5.596808477 | up | NM_025976 | chr16:13702719-13702779 | Bfar |
| 5.595924239 | up | NM_008361 | chr2:129190406-129190348 | Il2b |
| 5.595040002 | up | AK141641 | chr1:97227735-97227795 | BC055309 |
| 5.594155764 | up | NM_001025576 | chr2:76892655-76887779 | Ccdc142 |

**Supplementary Table 2: CLP-induced AKI with EGFR WT VS Sham down regulate genes**

| **Fold Change** | **Regulation** | **GenbankAccession** | **GenomicCoordinates** | **GeneSymbol** |
| --- | --- | --- | --- | --- |
| 2.7800118 | down | NM_145375 | chr7:89028246-89028305 | Tm6sf1 |
| 5.3886613 | down | NM_175686 | chr1:165175434-165175375 | Prrx1 |
| 2.4672057 | down | NM_008180 | chr2:155390531-155389432 | Gss |
| 3.7383165 | down | NM_001081430 | chr14:49810345-49810404 | Naa30 |
| 2.3927215 | down |  | chr17:15703269-15703210 |  |
| 5.5027722 | down |  | chr7:069896175-069896116 |  |
| 2.631233 | down | NM_011546 | chr18:5774613-5774672 | Zeb1 |
| 2.2826774 | down | NM_178080 | chr3:97542865-97542806 | Pde4dip |
| 3.7851986 | down | XM_003945650 | chr11:99701412-99701353 | LOC101055913 |
| 3.3289216 | down | XR_168941 | chr16:31428129-31428070 |  |
| 5.2792281 | down | NM_145423 | chr10:88391888-88391947 | Slc5a8 |
| 2.9137357 | down | XM_357236 | chr2:161308643-161308585 | Gm11449 |
| 15.3142839 | down |  | chr1:079919568-079919509 |  |
| 7.2501558 | down | NM_008828 | chr3:57906894-57906830 | Pgk1 |
| 3.3326657 | down | XR_107859 | chr6:148895581-148895640 | 3010003L21Rik |
| 3.4700561 | down | NM_144902 | chr3:116376192-116376133 | Slc35a3 |
| 2.1980268 | down | NM_001252442 | chr16:45746452-45746393 | Phldb2 |
| 10.4161144 | down | NM_134114 | chr17:8513481-8513540 | Sft2d1 |
| 4.4155266 | down | AK135714 | chr1:72313735-72313676 | Pecr |
| 2.0559098 | down | NM_134007 | chr10:70793834-70793775 | Cisd1 |
| 2.4785614 | down | NM_019678 | chr16:56690553-56690494 | Tfg |
| 4.0790639 | down | NR_045333 | chr9:52590862-52590921 | Gm1715 |
| 2.2625474 | down | NM_009294 | chr7:134992131-134992273 | Stx4a |
| 2.3207728 | down | NM_144937 | chr9:66366090-66366031 | Usp3 |
| 3.0034747 | down | NM_001080795 | chr5:92481415-92481356 | G3bp2 |
| 5.5479254 | down | NM_177670 | chr4:116225827-116225768 | Tmem69 |
| 3.0398286 | down | NM_001033173 | chr7:128786046-128785987 | Usp31 |
| 2.2912499 | down | NM_011693 | chr3:115813275-115813216 | Vcam1 |
| 2.7539608 | down | NM_177025 | chr2:64926473-64926414 | Cobll1 |
| 5.8603462 | down | NM_013757 | chrX:130471009-130470950 | Sytl4 |
| 4.546376 | down | NM_008650 | chr17:41095817-41097574 | Mut |
| 5.1022111 | down | NM_001081267 | chr7:104841145-104841204 | Rsf1 |
| 6.1807461 | down | NM_001081019 | chr4:94266841-94266900 | Gm12657 |
| 2.7633195 | down | NM_001285446 | chr2:154364934-154364993 | Cbfa2t2 |
| 2.8880165 | down | NM_178693 | chr2:29652292-29652351 | Coq4 |
| 5.337037 | down | NM_019975 | chr14:32421176-32421117 | Hacl1 |
| 4.7108895 | down | NM_009928 | chr4:47325273-47325332 | Col15a1 |
| 5.0409862 | down | NM_001146707 | chr10:110934911-110934970 | Nap1l1 |
| 7.5707488 | down | NM_001033302 | chr3:95682492-95682433 | Gm129 |
| 3.2638381 | down | NR_030673 | chr5:104980887-104980946 | D930016D06Rik |
| 2.784214 | down | NM_027727 | chr18:75403038-75446318 | Dym |
| 6.4452173 | down | NM_007544 | chr6:120843523-120843464 | Bid |
| 3.3779203 | down | NM_011668 | chr7:66561271-66561330 | Ube3a |
| 3.9860751 | down | NR_002928 | chr15:8043015-8042956 | Gm1943 |
| 2.1209138 | down | NM_177292 | chr5:114039596-114039655 | Wscd2 |
| 3.9127773 | down |  | chr11:032939584-032939525 |  |
| 14.3210662 | down | NM_133222 | chr3:151207470-151207529 | Eltd1 |
| 2.9286395 | down | NM_001111336 | chr3:98314276-98313904 | Hsd3b4 |
| 3.1750194 | down | NM_029607 | chr2:180449577-180451608 | Gid8 |
| 18.3350844 | down | NR_030693 | chr4:9750472-9750531 | 4930412C18Rik |
| 7.2880063 | down | NM_011159 | chr16:15839317-15841922 | Prkdc |
| 5.4683194 | down | NM_028815 | chr16:55925146-55925087 | Cep97 |
| 2.3438153 | down | NM_025845 | chr3:108706263-108706204 | Prpf38b |
| 3.0789341 | down | NM_011392 | chr13:55515201-55515259 | Slc34a1 |
| 3.0717674 | down | NM_080595 | chr11:5006332-5006273 | Emid1 |
| 7.4621295 | down | NM_001035231 | chr13:67639675-67639616 | Zfp748 |
| 4.3899561 | down | NM_010132 | chr19:59539142-59539201 | Emx2 |
| 2.6759729 | down | NM_152220 | chr19:11859903-11857591 | Stx3 |
| 4.9651352 | down | NM_053262 | chr5:104419186-104419127 | Hsd17b11 |
| 4.0884514 | down | NR_045403 | chr18:32518779-32518720 | A830052D11Rik |
| 4.437886 | down | NM_029280 | chr2:69717219-69717160 | Mettl5 |
| 2.610729 | down | NM_146389 | chr7:6523331-6523390 | Olfr1350 |
| 2.0362829 | down | NM_001039147 | chr9:89986588-89986529 | Morf4l1 |
| 2.574211 | down | NM_024440 | chr10:75358503-75358562 | Derl3 |
| 14.3634758 | down | NM_138756 | chr9:96978225-96978166 | Slc25a36 |
| 3.8161545 | down | NM_027460 | chr4:149126565-149123432 | Slc25a33 |
| 2.7469126 | down | NM_009063 | chr1:171623021-171623080 | Rgs5 |
| 2.1366987 | down | NM_026885 | chr7:13618024-13617889 | Chmp2a |
| 2.2938876 | down | NM_001286396 | chr5:114470971-114471030 | Dao |
| 2.7665078 | down | NM_025882 | chr6:82597794-82597735 | Pole4 |
| 7.4392697 | down | NM_021386 | chr14:119273459-119273518 | Cldn10 |
| 2.804633 | down |  | chrX:077306558-077306499 |  |
| 2.8354067 | down |  | chr2:032261625-032261566 |  |
| 3.3796768 | down | NM_011200 | chr1:30997727-30997668 | Ptp4a1 |
| 2.9171023 | down | NM_009454 | chr2:78760395-78760454 | Ube2e3 |
| 2.2933968 | down | NM_001039126 | chr1:93455932-93455991 | Asb1 |
| 2.6743291 | down | NM_025785 | chr8:13940398-13940457 | Fbxo25 |
| 2.5912354 | down | NM_181266 | chr2:149942992-149942933 | Zfp120 |
| 4.4669094 | down | NM_009127 | chr19:44469446-44469387 | Scd1 |
| 3.3981558 | down | NM_029269 | chr1:90322944-90323003 | Spp2 |
| 2.4530455 | down | NM_001081309 | chr9:105589596-105589655 | Pik3r4 |
| 2.3351794 | down | NM_001025103 | chr7:148652239-148652298 | Efcab4a |
| 3.6692355 | down | NM_010288 | chr10:56109814-56109873 | Gja1 |
| 5.3402527 | down | NM_029840 | chr4:21684868-21684809 | Tstd3 |
| 2.3439585 | down | NM_008084 | chr6:125113288-125113229 | Gapdh |
| 4.3957429 | down |  | chr1:055438089-055438030 |  |
| 6.5263185 | down | NM_011666 | chr6:97133873-97133814 | Uba3 |
| 4.6567212 | down | NM_001205369 | chr2:121761338-121761397 | Casc4 |
| 4.3826322 | down | AK046125 | chr12:58600733-58600792 |  |
| 2.8252901 | down | NM_133236 | chr6:8543357-8543416 | Glcci1 |
| 2.5208046 | down | AK012899 | chr3:23703462-23703403 | Naaladl2 |
| 5.2389268 | down | NM_026963 | chr4:148870470-148870529 | Lzic |
| 6.2941519 | down | NM_021514 | chr15:97962713-97962772 | Pfkm |
| 2.9101701 | down | NM_029384 | chr7:4736451-4736392 | Tmem238 |
| 3.1442959 | down |  | chr5:020094668-020094727 |  |
| 5.2172248 | down | NM_007619 | chr9:43958183-43958124 | Cbl |
| 3.2352034 | down | NM_175193 | chr3:75680656-75680597 | Golim4 |
| 19.5927622 | down | AK006385 | chr10:19890977-19890918 | 1700026H06Rik |
| 3.6507781 | down | NM_001033128 | chr19:4887093-4887034 | Bbs1 |
| 3.3251433 | down | NM_178647 | chr16:64859213-64859272 | Cggbp1 |
| 3.0597922 | down | NM_019827 | chr16:38187987-38191632 | Gsk3b |
| 2.171318 | down | NM_172678 | chr3:35990197-35990256 | Acad9 |
| 9.1669811 | down | NM_007423 | chr5:90937781-90937840 | Afp |
| 2.1513958 | down | NM_172676 | chr2:181330510-181330451 | Samd10 |
| 2.8643816 | down | NM_024219 | chr8:121868865-121869534 | Hsbp1 |
| 2.5362453 | down | NM_134157 | chr6:83706880-83707858 | Atp6v1b1 |
| 6.5430895 | down | NM_019951 | chr7:88068059-88068000 | Sec11a |
| 6.5357237 | down | NM_023605 | chr9:77929524-77929465 | Fbxo9 |
| 5.7584423 | down | NM_001081054 | chr10:43594408-43594349 | Qrsl1 |
| 2.6274773 | down | NM_001013829 | chr2:122174994-122174935 | Shf |
| 2.3469201 | down | NM_153551 | chr17:57205573-57205514 | Dennd1c |
| 2.3024721 | down | NM_001039373 | chrX:72655878-72655819 | Mtcp1 |
| 3.9508454 | down | NM_001267796 | chr18:36461008-36461067 | Igip |
| 2.165033 | down | NM_148925 | chr9:123699114-123699055 | Fyco1 |
| 2.6549634 | down | NM_001004143 | chr11:60966417-60966358 | Usp22 |
| 4.1704819 | down | NM_010072 | chr2:168034706-168034647 | Dpm1 |
| 5.7866444 | down | NM_009479 | chr7:140878128-140878069 | Uros |
| 2.5315711 | down | NM_001081196 | chr19:8908365-8908424 | Hnrnpul2 |
| 2.8008713 | down | NM_013508 | chr10:92710237-92710178 | Elk3 |
| 2.7288101 | down | NM_175025 | chr9:105313784-105313725 | Atp2c1 |
| 2.4311923 | down | NM_173007 | chr6:21721482-21721423 | Tspan12 |
| 2.2144606 | down | NM_008865 | chr13:27341385-27341444 | Prl3b1 |
| 5.3633485 | down | NM_013614 | chr12:17557831-17557890 | Odc1 |
| 8.6464744 | down | NM_026617 | chr10:119657822-119659102 | Tmbim4 |
| 24.7550679 | down | NM_028719 | chr9:104935202-104935261 | Cpne4 |
| 13.0023726 | down | NM_027299 | chr12:109928560-109928501 | Degs2 |
| 3.1004974 | down | NM_001044386 | chrX:91322245-91322186 | Zfx |
| 2.7163632 | down | NM_178218 | chr11:58768954-58769013 | Hist3h2a |
| 2.9198284 | down | NM_025902 | chr3:135070200-135070141 | Cisd2 |
| 4.7507102 | down | NM_001134697 | chr18:57637225-57637284 | Ctxn3 |
| 4.6848261 | down | NM_030731 | chr13:104991894-104991953 | Trim23 |
| 2.5437021 | down | NM_054094 | chr7:126798951-126799010 | Acsm1 |
| 3.1868831 | down | NM_001111111 | chr7:108439146-108438916 | Atg16l2 |
| 2.2457749 | down | NM_028303 | chrX:97818337-97818278 | Pdzd11 |
| 18.3141939 | down | XM_003085465 | chr12:114497044-114496985 | Igh-VJ558 |
| 2.847153 | down | NM_025942 | chr2:72931313-72931254 | Ola1 |
| 5.5739718 | down | NM_028023 | chr12:114059030-114058971 | Cdca4 |
| 5.727131 | down | NM_025508 | chr13:45616334-45616393 | Gmpr |
| 2.0309131 | down | NM_175224 | chr3:138122199-138122140 | Metap1 |
| 3.5758767 | down | NM_010902 | chr2:75513830-75513771 | Nfe2l2 |
| 6.6004885 | down | NM_001134457 | chr16:55843384-55843325 | Nxpe3 |
| 50.0417348 | down | NM_025698 | chr18:46747436-46747377 | Tmed7 |
| 4.5519856 | down | NM_007452 | chr19:60940207-60940148 | Prdx3 |
| 17.223441 | down | NM_176917 | chr17:95126527-95126468 | Mettl4 |
| 5.2928593 | down | AK081077 | chrX:146900076-146900136 | 3110067C02Rik |
| 3.3958906 | down |  | chr17:016985913-016985972 |  |
| 5.0252686 | down | NM_010628 | chr9:110423843-110427421 | Kif9 |
| 10.0281293 | down | NM_145820 | chr3:65861147-65861088 | Veph1 |
| 4.8415198 | down | NM_021282 | chr7:147960628-147960687 | Cyp2e1 |
| 5.3037126 | down | NM_029847 | chr13:76198868-76198809 | Arsk |
| 2.0769997 | down | NM_001123038 | chr16:23113939-23113998 | Eif4a2 |
| 5.9836384 | down | NM_001047604 | chr2:66022601-66022542 | Ttc21b |
| 6.7956446 | down |  | chr3:037498924-037498865 |  |
| 4.0879739 | down | NM_001004141 | chr4:95961501-95961442 | Cyp2j11 |
| 2.0318304 | down | NM_001190179 | chr2:80498883-80498942 | Nup35 |
| 5.5722346 | down | NM_026838 | chrX:130466293-130466352 | Srpx2 |
| 2.2203606 | down |  | chrX:038646944-038646885 |  |
| 2.2148331 | down | NM_015804 | chr8:12868669-12868728 | Atp11a |
| 5.8730544 | down | NM_177322 | chr13:30474638-30474697 | Agtr1a |
| 3.2190759 | down | NM_008187 | chr8:97945043-97944518 | Gtl3 |
| 10.1801718 | down | NM_007552 | chr2:18607987-18608046 | Bmi1 |
| 2.0247009 | down |  | chr14:004850203-004850262 |  |
| 2.3091278 | down |  | chr2:024350964-024351023 |  |
| 8.1879268 | down | NM_008229 | chr10:36713455-36713589 | Hdac2 |
| 2.2307434 | down | NM_172511 | chr16:45732034-45731975 | Abhd10 |
| 4.8506511 | down | NM_178603 | chr4:49527148-49527089 | Mrpl50 |
| 2.0465659 | down | NM_001159361 | chr15:100049416-100049475 | Dip2b |
| 6.6035664 | down | NM_001286383 | chr8:106781283-106781342 | Cklf |
| 3.8652186 | down | NM_001024926 | chr11:72590810-72590751 | Cyb5d2 |
| 2.5194695 | down | NM_134420 | chr9:108815322-108815380 | Slc26a6 |
| 2.0346604 | down | NM_026969 | chr5:100790818-100790759 | Sec31a |
| 48.667761 | down | NM_153193 | chr3:98515113-98515054 | Hsd3b2 |
| 12.9285269 | down | NM_011515 | chrX_random:11152-11093 | Vamp7 |
| 2.1703724 | down | NM_033322 | chr9:123608912-123608853 | Lztfl1 |
| 3.8988845 | down | NM_053015 | chr1:92845282-92845341 | Mlph |
| 4.3840523 | down | NM_018859 | chr13:4592211-4592152 | Akr1e1 |
| 2.6523828 | down | NM_010261 | chr7:25757181-25757122 | Rabac1 |
| 5.7366401 | down | NM_053162 | chr8:73989526-73989585 | Mrpl34 |
| 4.9034617 | down | NR_045474 | chr13:50357697-50357756 | 2310081J21Rik |
| 2.0019792 | down | NM_013498 | chr18:3267108-3267049 | Crem |
| 5.373604 | down | NM_147050 | chr7:111820127-111820186 | Olfr659 |
| 7.5081866 | down | NM_028603 | chr4:129031222-129031163 | Zbtb8a |
| 2.7770888 | down | NM_001042499 | chr16:37572151-37572210 | Rabl3 |
| 2.2760922 | down | NM_201362 | chr18:70128905-70128964 | Ccdc68 |
| 5.1863156 | down | NM_025684 | chr10:52124110-52124169 | Nepn |
| 2.5422836 | down | NM_010006 | chr15:82285582-82285641 | Cyp2d9 |
| 2.5549556 | down | NM_183221 | chr3:38910550-38910609 | Fat4 |
| 2.2934874 | down | NM_001012325 | chr13:67171196-67171137 | Zfp708 |
| 2.8663516 | down | NM_011890 | chr5:74026638-74026579 | Sgcb |
| 2.6938005 | down | NM_001276316 | chrX:20259770-20259829 | Uba1 |
| 3.5293774 | down | NM_001161458 | chr8:107741792-107741851 | Cbfb |
| 14.6339477 | down | NM_001024135 | chr14:79830198-79830257 | Kbtbd7 |
| 3.8247355 | down | NM_001286413 | chr8:42076470-42076411 | Mtus1 |
| 13.2107768 | down | NM_008796 | chr11:89845278-89845219 | Pctp |
| 4.7424251 | down | NM_010934 | chr8:69230632-69230691 | Npy1r |
| 3.980283 | down | NM_010709 | chr1:133064801-133067556 | Eif2d |
| 5.1271375 | down | NM_153572 | chr5:149700527-149700468 | Katnal1 |
| 4.4416583 | down | NM_001193271 | chr11:18780754-18780695 | Meis1 |
| 4.847708 | down | NM_001001883 | chr1:53864193-53864134 | Hecw2 |
| 3.336041 | down | NM_018829 | chr14:21855356-21855297 | Ap3m1 |
| 4.9148862 | down |  | chr13:083973142-083973201 |  |
| 2.1045744 | down | NM_001256100 | chr5:147766191-147766132 | Mtif3 |
| 4.345982 | down | AK016962 | chr7:116638566-116638625 | 4933428C20Rik |
| 2.9472705 | down | NM_025439 | chr1:137931523-137931582 | Tmem9 |
| 2.2213065 | down | NM_177470 | chr18:74965691-74965750 | Acaa2 |
| 2.4900919 | down | NM_146008 | chr10:84076719-84076778 | Tcp11l2 |
| 2.7554692 | down | NM_013645 | chr15:78021608-78021549 | Pvalb |
| 3.0320713 | down | NM_022323 | chr12:103980742-103980683 | Moap1 |
| 6.1441345 | down | NM_198026 | chr4:129295834-129295775 | Iqcc |
| 6.5840651 | down | AK142189 | chr8:93430530-93430471 | Gm6658 |
| 2.0564464 | down | NM_007450 | chr8:47292887-47292828 | Slc25a4 |
| 2.5057346 | down | NM_145573 | chr6:147009999-147019119 | Mrps35 |
| 2.2386205 | down | NM_173026 | chr16:56008005-56008064 | Zbtb11 |
| 14.4126728 | down | NM_021435 | chr6:34106406-34106347 | Slc35b4 |
| 2.0320863 | down | AK046110 | chr10:70063162-70063221 | BB187676 |
| 4.5046874 | down | NM_025768 | chr8:13179373-13177134 | Grtp1 |
| 3.4435962 | down | NM_027430 | chr1:167409699-167411082 | Mpc2 |
| 5.4769044 | down | NM_025747 | chr8:47280531-47280590 | 4933411K20Rik |
| 2.0150956 | down | NM_001110013 | chr10:99907014-99906955 | Tmtc3 |
| 3.0687263 | down | NM_001272097 | chr3:10016185-10016244 | Fabp5 |
| 2.7281028 | down | NM_007505 | chr18:78021546-78021605 | Atp5a1 |
| 2.3175864 | down | NM_026908 | chr14:60157982-60158041 | Cab39l |
| 2.1498509 | down | NM_026500 | chr1:138336345-138336404 | Ddx59 |
| 4.128164 | down | NM_172116 | chr7:148594637-148594578 | Pddc1 |
| 8.7982586 | down | NM_133228 | chr13:67616724-67616717 | Zfp87 |
| 5.9221581 | down | NM_026582 | chr3:159597867-159597926 | Wls |
| 2.1428643 | down | NM_007808 | chr6:50515151-50515092 | Cycs |
| 20.132508 | down | NM_010368 | chr5:130465192-130465133 | Gusb |
| 5.0224295 | down | NM_023750 | chr7:30566378-30566437 | Zfp84 |
| 5.6896306 | down |  | chr1:071760707-071760648 |  |
| 3.6109489 | down | NM_001198571 | chr1:60535727-60535786 | Abi2 |
| 2.7181419 | down | NM_146888 | chr2:111461629-111461570 | Olfr1297 |
| 10.3275819 | down | NM_001199337 | chrX:91639559-91639618 | Apoo |
| 2.8690577 | down | NM_198171 | chr8:107362421-107362480 | Ces2b |
| 6.1020965 | down | NM_025730 | chr15:91646424-91646483 | Lrrk2 |
| 2.7165941 | down |  | chr6:047927650-047927591 |  |
| 7.0582327 | down | NM_053200 | chr8:95694933-95693648 | Ces1d |
| 2.0028453 | down | NM_175446 | chrX:131506144-131506085 | Zmat1 |
| 2.8396281 | down | NM_010330 | chr13:118062731-118062790 | Emb |
| 8.6458032 | down | NM_019660 | chr4:123589107-123589166 | Mycbp |
| 4.4329006 | down | NM_133894 | chr5:87839404-87839345 | Ugt2b38 |
| 9.5658157 | down | NM_008760 | chr13:49719549-49719608 | Ogn |
| 2.5635375 | down | NM_016720 | chr7:106960012-106959953 | Neu3 |
| 11.7927968 | down | NM_172776 | chr19:8234895-8234836 | Slc22a29 |
| 5.2589173 | down | NM_001033500 | chr9:74130576-74130635 | Wdr72 |
| 8.07824 | down | NM_019480 | chr15:44472212-44472271 | Ebag9 |
| 3.9086885 | down | NM_001085509 | chr4:135371377-135371436 | Myom3 |
| 5.0650517 | down |  | chr3:008330047-008330106 |  |
| 2.4984304 | down | NM_133726 | chr15:81195753-81195694 | St13 |
| 50.8154325 | down | NM_028235 | chr2:75774347-75774288 | Ttc30b |
| 4.228503 | down | NM_026626 | chr1:180405062-180405844 | Efcab2 |
| 5.9290465 | down | NM_144894 | chr2:181115177-181115236 | Zgpat |
| 2.6992676 | down | NM_033568 | chr11:95906602-95907638 | Snf8 |
| 19.0588478 | down | NM_021526 | chr2:61599058-61599117 | Psmd14 |
| 8.5890347 | down | NM_016972 | chr14:55341196-55341137 | Slc7a8 |
| 2.6342495 | down | NM_207216 | chr15:9244664-9244723 | Ugt3a1 |
| 10.2694855 | down | NM_017472 | chr10:42254534-42254593 | Snx3 |
| 8.1708247 | down | NM_030712 | chr9:123720709-123720768 | Cxcr6 |
| 2.2897399 | down | NM_008879 | chr14:75630210-75630269 | Lcp1 |
| 3.2035578 | down | NM_009029 | chr14:73595467-73595408 | Rb1 |
| 2.0103341 | down | NM_027280 | chr8:91116160-91116219 | Nkd1 |
| 5.5997891 | down |  | chr4:116121949-116122008 |  |
| 5.2203434 | down |  | chr15:010304079-010304138 |  |
| 2.7318097 | down | NR_024069 | chr14:31941903-31941844 | Smim4 |
| 2.9406193 | down | NM_010573 | chr13:72095794-72095735 | Irx1 |
| 5.5007804 | down | NM_177992 | chr14:56297079-56297138 | Gmpr2 |
| 10.3246287 | down | NM_008376 | chr6:48693409-48693468 | Gimap1 |
| 2.3270642 | down |  | chr3:037498264-037498205 |  |
| 5.6121735 | down | NM_030685 | chr3:58327639-58327580 | Serp1 |
| 2.2895642 | down | NM_013795 | chr9:44721433-44721374 | Atp5l |
| 8.6844832 | down | NM_176928 | chr16:96288587-96288528 | Brwd1 |
| 9.0830385 | down | NM_145953 | chr3:157560242-157560183 | Cth |
| 13.8816832 | down | NM_028719 | chr9:104936317-104936376 | Cpne4 |
| 12.0619872 | down | NR_033592 | chr3:55651541-55651600 | 4933417G07Rik |
| 6.1063543 | down | NM_194054 | chr11:29641510-29641717 | Rtn4 |
| 3.8863504 | down | NM_010072 | chr2:168035937-168035878 | Dpm1 |
| 3.0839759 | down | NM_019730 | chr17:25034222-25034281 | Nme3 |
| 8.8654908 | down | NM_025918 | chr11:102546495-102546436 | Ccdc43 |
| 7.3785263 | down | NM_026678 | chr2:126912587-126912646 | Blvra |
| 3.567656 | down | NM_172919 | chr9:20399180-20399239 | Zfp846 |
| 3.8539557 | down | NM_009939 | chr2:125657745-125657686 | Cops2 |
| 3.141085 | down | NM_011358 | chr11:116712053-116711994 | Srsf2 |
| 5.4065152 | down | NM_027256 | chr7:104678920-104679300 | Ints4 |
| 2.9749667 | down | NM_029321 | chr12:9042715-9042774 | Ttc32 |
| 2.0956765 | down | NM_172644 | chr1:162971793-162971734 | Dars2 |
| 10.0984684 | down | NM_016900 | chr6:17238393-17238452 | Cav2 |
| 5.2200716 | down | NM_173751 | chr10:78046717-78046862 | Ilvbl |
| 2.2335978 | down | NR_002321 | chr11:3540530-3540471 | Tug1 |
| 2.1646383 | down |  | chr16:011185368-011185309 |  |
| 4.6740261 | down | NM_019647 | chr5:147648267-147648326 | Rpl21 |
| 6.2745339 | down | NM_025494 | chr15:38621773-38621832 | Atp6v1c1 |
| 6.9670634 | down |  | chrX:045078999-045079058 |  |
| 4.6987825 | down | NM_133351 | chr7:135069907-135069848 | Prss8 |
| 2.1495773 | down | NM_012003 | chr6:124908990-124908931 | Cops7a |
| 10.7733386 | down | NM_028075 | chr15:82052262-82052203 | Tnfrsf13c |
| 20.3694201 | down | NM_001081179 | chr17:79152597-79152538 | Heatr5b |
| 2.2188621 | down | NM_010612 | chr5:76329368-76329309 | Kdr |
| 2.1677765 | down | NM_126165 | chr8:109569326-109569385 | Vps4a |
| 2.259254 | down | AK083616 | chr1:170083754-170083695 | Pbx1 |
| 2.4254375 | down | NM_010318 | chr3:146168390-146168449 | Gng5 |
| 2.6223397 | down |  | chr8:035370276-035370217 |  |
| 20.4078798 | down | NM_027016 | chr3:30719504-30719563 | Sec62 |
| 3.6210282 | down | NM_020589 | chr6:48386695-48386636 | Zfp467 |
| 2.4604292 | down | NM_001142727 | chr4:124526929-124526870 | 1110065P20Rik |
| 2.3088109 | down | NM_146066 | chr16:11220564-11220505 | Gspt1 |
| 2.2672722 | down | AK045571 | chr12:3510747-3510806 | BB283564 |
| 3.193905 | down | NM_028108 | chr16:44163398-44163457 | Naa50 |
| 3.3618464 | down | NM_001033270 | chr14:15632353-15632412 | Slc4a7 |
| 2.3768782 | down |  | chr17:028863380-028863439 |  |
| 5.4765335 | down | NM_021372 | chr11:20552457-20552516 | Sertad2 |
| 2.2677498 | down | NM_027919 | chr11:117729712-117729653 | Tha1 |
| 4.247824 | down | NM_008303 | chr1:55147620-55147946 | Hspe1 |
| 2.3479456 | down | NM_027878 | chr10:87786227-87786168 | Dram1 |
| 5.3722398 | down | NM_026085 | chr10:62521353-62521412 | Pbld2 |
| 2.6626781 | down | NM_010174 | chr4:129992643-129992702 | Fabp3 |
| 4.7947098 | down | NM_009199 | chr19:28988332-28988391 | Slc1a1 |
| 3.6409108 | down | NM_026560 | chr4:124595868-124595809 | Cdca8 |
| 19.0015342 | down | NM_001160345 | chr7:59252821-59252762 | Svip |
| 5.7586287 | down | NM_011494 | chr1:75211803-75211862 | Stk16 |
| 2.6407243 | down | AK044029 | chr15:81525907-81525966 |  |
| 2.7871024 | down | NM_010193 | chr9:62639796-62639737 | Fem1b |
| 5.5276413 | down | NM_198164 | chr10:40201715-40201774 | Cdk19 |
| 6.0187133 | down |  | chr11:083459007-083458946 |  |
| 2.4972296 | down | NM_013556 | chrX:50374757-50374816 | Hprt |
| 2.4533864 | down | NM_181415 | chr19:58207449-58207508 | Atrnl1 |
| 4.0779752 | down | NM_133926 | chr6:113286428-113286369 | Camk1 |
| 2.3480709 | down | NM_001166399 | chr8:73282447-73282506 | Rab3a |
| 2.6085958 | down | NM_001081392 | chr4:32861180-32861239 | Mdn1 |
| 5.366955 | down | NM_172726 | chr5:144063232-144063173 | E130309D02Rik |
| 17.6968392 | down | AK004585 | chr12:40951741-40951800 | Gm7008 |
| 4.7790869 | down | NM_007508 | chr16:44085642-44085583 | Atp6v1a |
| 3.1403362 | down |  | chr4:122952503-122952444 |  |
| 2.2524635 | down | NM_008067 | chrX:69678090-69678031 | Gabra3 |
| 14.1688523 | down | NM_026993 | chr3:145516152-145516211 | Ddah1 |
| 2.6971798 | down | NM_011605 | chr10:90623781-90623722 | Tmpo |
| 5.110734 | down | NM_011960 | chr14:33110162-33110221 | Parg |
| 8.4319832 | down | NM_010113 | chr3:129380885-129380826 | Egf |
| 3.2984298 | down | NM_024454 | chr10:114727197-114727138 | Rab21 |
| 4.3769239 | down | NM_011988 | chr3:90189221-90189162 | Slc27a3 |
| 3.0311411 | down | NM_026004 | chr6:56832582-56832523 | Nt5c3 |
| 2.4442055 | down | NM_175530 | chr7:19723406-19723465 | Fbxo46 |
| 3.655825 | down | NM_010344 | chr8:34808413-34808472 | Gsr |
| 2.2202106 | down | NM_026444 | chr10:127799428-127799487 | Cs |
| 4.2752832 | down | NM_001164220 | chr14:62224545-62224604 | Trim13 |
| 2.4893533 | down | NM_008065 | chr16:84863822-84863881 | Gabpa |
| 8.1310169 | down | NM_026149 | chr15:44208039-44207980 | Nudcd1 |
| 3.1303002 | down |  | chr17:035542095-035542154 |  |
| 5.1951846 | down | NM_008173 | chr18:39571700-39571641 | Nr3c1 |
| 2.1721615 | down | NM_001164661 | chr7:63185989-63186048 | Cyfip1 |
| 5.2400715 | down | NM_008772 | chr3:60812600-60812659 | P2ry1 |
| 4.5485686 | down | NM_019584 | chr11:101152713-101151771 | Becn1 |
| 2.634118 | down |  | chr12:089234098-089234039 |  |
| 12.2194818 | down | NM_207246 | chr17:75928310-75928369 | Rasgrp3 |
| 5.6035096 | down | AK044736 | chr14:35579141-35579200 | A930038B10Rik |
| 2.0007571 | down | NM_024208 | chr2:6110022-6109963 | Echdc3 |
| 2.6961703 | down | NM_001113416 | chr1:121504664-121504605 | Epb4.1l5 |
| 17.6773535 | down | NM_001085515 | chr4:41443223-41443164 | AI464131 |
| 2.4836054 | down | NM_026899 | chr4:155107818-155107877 | Ssu72 |
| 2.3377327 | down | NM_134142 | chr19:10945277-10945218 | Tmem109 |
| 4.8620226 | down | DQ459435 | chr10:81863532-81863591 | Gm4924 |
| 2.0035541 | down | NM_028709 | chr10:85122975-85123034 | Btbd11 |
| 2.2329129 | down | NR_003623 | chr1:182257894-182257835 | Gm5069 |
| 2.8619312 | down | NM_008546 | chr4:140571828-140571887 | Mfap2 |
| 5.6326741 | down | NM_001199337 | chrX:91662238-91662297 | Apoo |
| 2.7703443 | down | NM_011890 | chr5:74026573-74026514 | Sgcb |
| 3.0237671 | down | NM_009673 | chr3:36348124-36348065 | Anxa5 |
| 8.7986001 | down | NM_008574 | chr3:92387941-92387882 | Smcp |
| 3.3546703 | down | NM_172911 | chr8:37210529-37210588 | D8Ertd82e |
| 5.0920268 | down |  | chr8:074220124-074220183 |  |
| 4.0673609 | down | NM_133718 | chr9:79618713-79618654 | Tmem30a |
| 2.6175063 | down | NM_145441 | chr12:4886922-4886863 | Ubxn2a |
| 11.434753 | down |  | chr5:087805246-087805187 |  |
| 2.5214512 | down | NM_019877 | chr11:96711995-96712054 | Copz2 |
| 3.5098102 | down | NM_172918 | chr9:19453464-19453523 | Zfp317 |
| 2.0410077 | down | NM_001243161 | chr8:23178618-23178677 | Alg11 |
| 2.2137744 | down | AK088666 | chr12:114659689-114659630 |  |
| 5.1371101 | down | NM_023792 | chr19:34885860-34885801 | Pank1 |
| 4.9250863 | down | NM_029929 | chr5:123979502-123979443 | Vps33a |
| 4.350035 | down | NM_027204 | chr11:120349740-120349799 | Mrpl12 |
| 7.4562374 | down | NM_001034903 | chr6:147024058-147023999 | Mansc4 |
| 5.6726348 | down |  | chr7:017232990-017232931 |  |
| 27.8893924 | down | NM_001081643 | chrX:70448087-70448146 | Xlr3b |
| 2.1032757 | down | NM_001110310 | chrX:98407382-98407323 | Snx12 |
| 2.9318006 | down | NM_172120 | chr13:18958324-18958383 | Vps41 |
| 2.098031 | down | XM_909042 | chr5:147645779-147646649 | LOC634339 |
| 3.0535278 | down | NM_144828 | chr11:98218965-98219024 | Ppp1r1b |
| 2.4087055 | down | NM_001161817 | chr1:51807413-51807354 | Myo1b |
| 2.0831861 | down | NM_001037812 | chr1:59157500-59157441 | Tmem237 |
| 7.1810062 | down | NM_173744 | chr8:13952453-13952395 | Tdrp |
| 2.5348511 | down | NM_013761 | chr11:74720360-74720301 | Srr |
| 5.757917 | down | NM_028840 | chr3:19033543-19033484 | Armc1 |
| 2.4637663 | down | AK020469 | chr18:52650449-52650508 | Srfbp1 |
| 2.4788019 | down | NM_145459 | chr14:22803318-22803259 | Zfp503 |
| 3.0043906 | down | NM_001285839 | chr1:53380049-53380108 | Osgepl1 |
| 4.6001006 | down | NM_023129 | chr10:53064944-53065003 | Pln |
| 2.8818755 | down | NM_007607 | chr11:84779487-84779546 | Car4 |
| 8.1564196 | down | NM_001171001 | chr6:29861897-29861956 | Ahcyl2 |
| 6.6205244 | down | NR_027872 | chr5:115224541-115224482 | 4930515G01Rik |
| 2.5183706 | down | NM_025943 | chr14:119275603-119275544 | Dzip1 |
| 3.4462905 | down | AK046180 | chr4:100676879-100676938 | Cachd1 |
| 4.1176951 | down | BC090643 | chr2:175433018-175432959 |  |
| 2.0328644 | down | XM_887355 | chrX:101577564-101577505 | Gm6238 |
| 3.1148075 | down | NM_025912 | chr2:172178905-172178964 | Fam210b |
| 2.6073206 | down |  | chr3:112105930-112105989 |  |
| 6.5123534 | down | NM_021881 | chr17:10403223-10403164 | Qk |
| 2.5701422 | down | NM_026597 | chr9:107886930-107886871 | Fam212a |
| 2.1315875 | down | NM_010561 | chr9:21209475-21209534 | Ilf3 |
| 2.5519517 | down | XM_003945624 | chr1:134181285-134181344 | Gm7241 |
| 2.9953722 | down | NM_011884 | chr4:33589410-33589469 | Rngtt |
| 4.1015053 | down | NM_001172160 | chr2:140485120-140485061 | Flrt3 |
| 2.244587 | down |  | chr11:085764019-085764078 |  |
| 2.6332646 | down | NM_016923 | chr1:16696289-16699561 | Ly96 |
| 13.5686473 | down | NM_033608 | chr1:174428911-174428970 | Igsf9 |
| 2.6453342 | down |  | chr1:061005062-061005003 |  |
| 2.0942563 | down | NM_001025375 | chr9:54565114-54565055 | Wdr61 |
| 4.6283469 | down | NM_146154 | chr4:132383252-132383193 | Ppp1r8 |
| 5.149921 | down | NM_133236 | chr6:8547424-8547483 | Glcci1 |
| 6.7363986 | down | NM_009250 | chr3:75420571-75420630 | Serpini1 |
| 3.604381 | down | NM_031863 | chr17:41060530-41060471 | Cenpq |
| 2.2427329 | down | NM_021713 | chr15:102167776-102167835 | Myg1 |
| 10.3989878 | down | NM_001167818 | chr19:33242836-33212730 | Rnls |
| 4.2926424 | down | NM_024441 | chr9:50559270-50559211 | Hspb2 |
| 2.089682 | down | NM_023626 | chr6:21925923-21925982 | Ing3 |
| 2.0121311 | down | NM_001198766 | chr3:54194840-54194899 | Postn |
| 4.0630939 | down | NM_009328 | chr2:151974683-151974742 | Tcf15 |
| 3.2868418 | down | NM_007757 | chr16:58680403-58680465 | Cpox |
| 2.7511809 | down | NM_024264 | chr1:74782203-74782262 | Cyp27a1 |
| 4.5260389 | down | NM_010019 | chr9:66119882-66119941 | Dapk2 |
| 2.8518201 | down | NM_029364 | chr10:120833970-120834029 | Gns |
| 3.3613249 | down | NM_021375 | chr3:88047324-88047265 | Rhbg |
| 7.3487287 | down | NM_001005509 | chr3:58352658-58352717 | Eif2a |
| 5.0315395 | down | AK039561 | chr1:157545229-157545170 | Lhx4 |
| 2.2397115 | down | NM_025833 | chr5:145025856-145025797 | Baiap2l1 |
| 24.5695018 | down | NM_011794 | chr1:187181308-187181367 | Bpnt1 |
| 2.9596734 | down | NM_011697 | chr19:7057304-7057245 | Vegfb |
| 8.0027636 | down | NM_027251 | chr10:61571402-61571343 | 2010107G23Rik |
| 3.0638458 | down | AK030820 | chr8:13377791-13377850 | Tfdp1 |
| 5.4321187 | down | NM_133187 | chr3:79746782-79746841 | Fam198b |
| 4.1844073 | down | NM_178766 | chr5:8454558-8454617 | Slc25a40 |
| 2.0835013 | down | NM_019830 | chr7:52234151-52234092 | Prmt1 |
| 3.3067088 | down | NM_178772 | chr3:27143709-27143768 | Nceh1 |
| 3.5957457 | down | NM_024195 | chr9:86936098-86937626 | Cyb5r4 |
| 2.1396703 | down | NM_020560 | chr8:23538340-23539688 | Mrps31 |
| 9.9705038 | down | NM_001170800 | chr10:3459901-3459960 | Ipcef1 |
| 2.9175905 | down | NM_025949 | chrX:108501945-108501886 | Rps6ka6 |
| 9.4651509 | down | NM_001001181 | chr18:75169229-75169288 | BC031181 |
| 4.3367852 | down | NM_013489 | chr1:173820027-173820086 | Cd84 |
| 4.5818289 | down | NM_172054 | chr1:38044308-38044249 | Txndc9 |
| 4.3670529 | down | NM_009677 | chr8:112384776-112384835 | Ap1g1 |
| 4.8377057 | down |  | chr14:095820521-095820580 |  |
| 24.1865485 | down | NM_153193 | chr3:98515762-98515703 | Hsd3b2 |
| 2.4321985 | down | AK012372 | chr2:128792875-128792816 |  |
| 2.0928471 | down |  | chr7:125591135-125591194 |  |
| 6.1040285 | down | NM_148937 | chr1:74612436-74612495 | Plcd4 |
| 2.9224554 | down | NM_177340 | chr18:60753691-60753644 | Synpo |
| 3.2326314 | down | NM_019990 | chr7:108470512-108491035 | Stard10 |
| 2.9559536 | down | NM_172577 | chr12:57813870-57813811 | Slc25a21 |
| 3.4937007 | down | NM_011341 | chr4:155383885-155383944 | Sdf4 |
| 4.5286765 | down | NM_009786 | chr1:162133675-162133616 | Cacybp |
| 4.2668962 | down |  |  |  |
| 9.9045608 | down | NM_030108 | chr5:67654974-67655033 | Tmem33 |
| 9.4482399 | down | NM_145542 | chr3:107466642-107466583 | Ahcyl1 |
| 3.2172098 | down | NM_001145433 | chr5:53669597-53669656 | Smim20 |
| 2.5840626 | down | NM_144915 | chr5:144264931-144264990 | Daglb |
| 2.8963099 | down | XR_106884 | chr14:121652974-121653033 | B930095G15Rik |
| 2.949628 | down | NM_029614 | chr7:96657714-96657655 | Prss23 |
| 3.3884153 | down |  | chr5:022199686-022199627 |  |
| 4.2328607 | down | NM_027212 | chr15:52561697-52561756 | Med30 |
| 2.4178312 | down | NM_025808 | chr16:17526364-17526423 | Lztr1 |
| 4.9410049 | down | NM_022982 | chr16:18152423-18152482 | Rtn4r |
| 2.2599089 | down | NM_026304 | chr7:97084271-97072201 | l7Rn6 |
| 2.567932 | down | NM_008228 | chr4:129193433-129193374 | Hdac1 |
| 2.2840303 | down | NM_013790 | chr16:20331627-20331568 | Abcc5 |
| 7.2832784 | down | NM_026417 | chr17:74899057-74899116 | Yipf4 |
| 5.3944422 | down | NM_145453 | chr13:91938488-91938429 | Zcchc9 |
| 2.9458372 | down | NM_001164578 | chrX:147521939-147521880 | Tsr2 |
| 3.2831644 | down | XM_003945562 | chr16:94533913-94533854 | Gm3004 |
| 2.2719651 | down | BC030357 | chr7:17718517-17718576 | Psg16 |
| 2.4469371 | down | NM_013478 | chr5:138431245-138431304 | Azgp1 |
| 6.0802768 | down | NM_009801 | chr3:14900206-14900265 | Car2 |
| 2.0038034 | down | NM_134046 | chr12:4213567-4213508 | Cenpo |
| 2.8556259 | down | NM_010481 | chr18:35097733-35097674 | Hspa9 |
| 3.3199879 | down | NM_001004148 | chr11:72055646-72055587 | Slc13a5 |
| 2.01854 | down | NM_153134 | chr7:25323184-25323243 | Irgq |
| 2.8068643 | down | NM_001013811 | chr7:75507905-75507964 | Fam169b |
| 2.9401423 | down | NM_031999 | chr13:13449950-13449891 | Gpr137b |
| 3.2018986 | down | NM_133810 | chr1:53813268-53813209 | Stk17b |
| 6.2612665 | down | NM_027925 | chr4:131877619-131875456 | Trnau1ap |
| 2.0101916 | down |  | chr4:046340345-046340404 |  |
| 3.0020063 | down | NM_028865 | chr11:116710984-116711043 | Mettl23 |
| 3.7226054 | down | NM_007956 | chr10:5343115-5343056 | Esr1 |
| 3.8912347 | down | NR_003363 | chr17:79250275-79250216 | Gm6548 |
| 5.1955339 | down | NM_010439 | chr5:149858997-149858938 | Hmgb1 |
| 6.8310195 | down | NM_001162532 | chr7:80921166-80921225 | Fam174b |
| 2.3735624 | down |  | chr8:087378078-087378019 |  |
| 3.2759733 | down | NM_025644 | chr19:42001046-41999695 | Exosc1 |
| 2.2598484 | down | NM_020256 | chrX:35549874-35549933 | Zbtb33 |
| 3.4470504 | down | NM_010216 | chrX:160840395-160840454 | Figf |
| 2.2228867 | down |  | chr13:067811237-067811178 |  |
| 22.1810999 | down | NM_013771 | chr2:23053737-23053796 | Yme1l1 |
| 2.5089001 | down | NM_010301 | chr10:80991730-80991671 | Gna11 |
| 8.6719149 | down | AK011139 | chr2:5791982-5792041 | Nudt5 |
| 3.5518693 | down | NM_023377 | chr7:90790202-90790261 | Stard5 |
| 11.4883078 | down | NM_173405 | chr5:141229165-141229224 | Amz1 |
| 9.717106 | down | NM_007574 | chr4:136445825-136445766 | C1qc |
| 6.0402732 | down | NM_028713 | chr1:55227173-55227114 | Rftn2 |
| 3.8718061 | down | NM_018817 | chr1:72682168-72682227 | Smarcal1 |
| 2.0760641 | down | NM_013826 | chr13:115618697-115618756 | Mocs2 |
| 6.2905973 | down | NM_009928 | chr4:47325261-47325320 | Col15a1 |
| 2.3242464 | down | AK021279 | chr2:79753953-79753894 | Pde1a |
| 3.7999633 | down | NM_030021 | chr2:34747507-34747566 | Cutal |
| 4.9985879 | down | NM_028904 | chr13:58511106-58511165 | Rmi1 |
| 2.0613393 | down | NM_011931 | chr1:161275084-161275143 | Rfwd2 |
| 2.7121839 | down |  | chr19:022013003-022013062 |  |
| 7.4971018 | down | NM_019545 | chr3:98678723-98678664 | Hao2 |
| 2.7226577 | down | NM_026577 | chr16:62793813-62793754 | Arl13b |
| 2.4195985 | down | NR_015487 | chr3:115590744-115590685 | A930005H10Rik |
| 2.9393089 | down | NM_013559 | chr5:150420169-150420110 | Hsph1 |
| 2.5601139 | down | NM_025424 | chr1:193130815-193130756 | Nenf |
| 2.2868124 | down | NM_053164 | chr19:45080035-45079976 | Mrpl43 |
| 6.6946503 | down | NM_007606 | chr3:14871781-14871840 | Car3 |
| 2.192517 | down | NM_029556 | chr14:122801250-122801309 | Clybl |
| 3.1821945 | down | NM_175245 | chr14:99434887-99434828 | Mzt1 |
| 13.2678849 | down | NM_023149 | chr18:84844496-84844437 | Cndp2 |
| 2.8423279 | down |  | chr11:49918193-49918252 | Rnf130 |
| 7.5893399 | down | NM_028637 | chr2:132576731-132576790 | 1110034G24Rik |
| 2.6373006 | down | NM_007396 | chr2:48757324-48757383 | Acvr2a |
| 3.7460132 | down | NM_175134 | chr15:36407801-36407742 | Ankrd46 |
| 5.1708882 | down | NM_011638 | chr16:32624877-32625093 | Tfrc |
| 7.1255383 | down | BC046519 | chr11:115936989-115936930 | Unc13d |
| 2.1538473 | down | NM_025382 | chr4:134358920-134358861 | Tmem57 |
| 4.1979791 | down | NM_001190371 | chr18:12413150-12413091 | Ankrd29 |
| 2.9802348 | down | NM_133738 | chr5:98313877-98313818 | Antxr2 |
| 14.5299114 | down | NM_138653 | chr4:62158017-62158076 | Bspry |
| 2.6445156 | down | NM_013703 | chr19:27324050-27324109 | Vldlr |
| 2.059263 | down | NM_008669 | chr15:82160595-82160536 | Naga |
| 7.047126 | down | NM_011170 | chr2:131763817-131763876 | Prnp |
| 2.1445129 | down | NM_025849 | chr4:74923429-74923370 | Tmem261 |
| 4.0262172 | down | NM_025292 | chr12:82602785-82602726 | Synj2bp |
| 3.5976504 | down | NM_008621 | chrX:72355890-72355831 | Mpp1 |
| 12.3897142 | down | NM_033521 | chr15:34213600-34213659 | Laptm4b |
| 4.0595573 | down | NM_001081425 | chr13:46526275-46526334 | Rbm24 |
| 7.1126413 | down | NM_177899 | chr8:72288731-72288672 | Zfp866 |
| 19.768722 | down | NM_018789 | chrX:98454974-98455033 | Foxo4 |
| 13.277128 | down | NM_027756 | chr8:63150717-63150776 | Mfap3l |
| 3.8251412 | down | NM_001001326 | chr7:116668179-116668120 | St5 |
| 5.0217632 | down | NM_025535 | chr11:51605100-51605159 | Sar1b |
| 3.6623788 | down | NM_008606 | chr10:75386268-75386209 | Mmp11 |
| 8.508678 | down | NM_016974 | chr7:52965322-52965381 | Dbp |
| 3.0048501 | down | NM_001252210 | chr11:110889223-110889282 | Kcnj16 |
| 3.3777112 | down | NM_173747 | chrX:7287246-7287305 | Gpkow |
| 3.6652612 | down | NM_001115087 | chr7:59116007-59115948 | Fancf |
| 4.4133373 | down | NM_027914 | chr10:110738166-110738225 | Bbs10 |
| 4.8050812 | down | NM_008152 | chr12:99514727-99514786 | Gpr65 |
| 2.9916031 | down | NM_001173460 | chr3:15495857-15495798 | Sirpb1b |
| 4.2893439 | down | NR_033602 | chr10:74815244-74815303 | Gm5779 |
| 3.9168796 | down | NM_029096 | chr5:121059489-121059430 | 1110008J03Rik |
| 2.5969433 | down | NM_178625 | chr6:30432526-30432467 | Tmem209 |
| 6.7348229 | down |  | chr6:085882767-085882708 |  |
| 2.0668555 | down | NM_025574 | chr6:57639736-57639677 | Pyurf |
| 6.7795551 | down | NM_144817 | chr1:195172608-195172549 | Camk1g |
| 3.5251843 | down | NM_133907 | chr5:30002424-30002483 | Ube3c |
| 7.7552823 | down | NM_029475 | chr2:120976923-120978200 | Adal |
| 3.4639152 | down | NM_026542 | chr11:102267022-102266350 | Slc25a39 |
| 3.2161342 | down | AK164386 | chr19:47575582-47575523 | Obfc1 |
| 10.1781348 | down | NM_025380 | chr13:38738137-38738078 | Eef1e1 |
| 2.1812558 | down | NM_001198872 | chr2:71101270-71101329 | Dync1i2 |
| 2.904294 | down | NM_009466 | chr5:65804591-65804532 | Ugdh |
| 2.1751882 | down | NM_199305 | chr4:129353687-129353628 | Tmem39b |
| 4.4416416 | down | NM_027967 | chr13:35002451-35002392 | Fam217a |
| 2.6583711 | down | XM_001472546 | chr4:94602632-94602691 | Gm12693 |
| 9.0298355 | down | NM_024201 | chr13:74496396-74496455 | Ccdc127 |
| 4.303776 | down |  | chr1:172123612-172123553 | Uhmk1 |
| 10.907783 | down | NM_138594 | chr6:126925408-126925467 | D6Wsu163e |
| 14.5229739 | down | NM_008316 | chr11:8893774-8893715 | Hus1 |
| 2.4066678 | down | NM_025887 | chr17:53646662-53646721 | Rab5a |
| 25.7935421 | down | NM_028878 | chr13:73818777-73818718 | Slc6a19 |
| 13.712536 | down | NM_177382 | chr7:121693743-121693684 | Cyp2r1 |
| 7.3406336 | down | NM_027827 | chr11:117700730-117700789 | Afmid |
| 4.0130538 | down | NM_029436 | chr16:20127758-20127817 | Klhl24 |
| 4.7677897 | down | NM_022980 | chr4:134968754-134968695 | Rcan3 |
| 5.7244993 | down | AK052609 | chr10:124656416-124656357 | D630004K10Rik |
| 2.7251205 | down | NM_145551 | chr4:111548937-111548878 | Slc5a9 |
| 4.3090923 | down |  | chr17:035496700-035496759 |  |
| 3.3251721 | down | NM_019953 | chr10:127763683-127764086 | Cnpy2 |
| 2.0452852 | down | NM_024255 | chr4:59631460-59631519 | Hsdl2 |
| 4.8355313 | down | NM_011279 | chr9:96372245-96372186 | Rnf7 |
| 20.3142149 | down | NM_145622 | chr13:67806569-67806510 | Zfp65 |
| 3.723313 | down | NM_177660 | chr3:9284831-9284890 | Zbtb10 |
| 2.1876815 | down | NM_008580 | chr10:19862193-19862252 | Map3k5 |
| 2.0604408 | down | NR_033633 | chr10:044726164-044726223 | Gm8709 |
| 3.8722363 | down | NM_001042620 | chr5:52545355-52545296 | Dhx15 |
| 3.4981884 | down | XM_003945509 | chr13:62151432-62151491 | Gm3325 |
| 2.8298802 | down |  | chr3:025630532-025630591 |  |
| 13.0044277 | down | NM_025813 | chr3:67398762-67400110 | Mfsd1 |
| 3.1872385 | down | NM_001013753 | chr14:84933861-84933920 | Pcdh17 |
| 12.1159658 | down | NM_134042 | chr12:85772860-85772801 | Aldh6a1 |
| 4.8972879 | down | AV310571 | chr11:041314267-041314326 |  |
| 12.3300009 | down | NM_025412 | chr15:75747234-75747175 | Pycrl |
| 4.6029348 | down | NM_001013390 | chr9:44962045-44962104 | Scn4b |
| 9.3684476 | down | NM_026911 | chr14:31813291-31813232 | Spcs1 |
| 2.0205614 | down |  | chr9:120432548-120432607 |  |
| 4.5868159 | down | NM_009542 | chr17:33517812-33517753 | Zfp101 |
| 9.3000341 | down | NM_011863 | chr3:131306415-131306474 | Papss1 |
| 8.6720976 | down | NM_080554 | chr2:34708167-34708108 | Psmd5 |
| 14.2519063 | down | NM_031164 | chr1:141420037-141420096 | F13b |
| 4.7951668 | down | NM_030250 | chr10:52159858-52159917 | Nus1 |
| 2.0286866 | down | NM_172118 | chr2:156607301-156607360 | Myl9 |
| 6.8314508 | down | NM_010119 | chr19:6298118-6298177 | Ehd1 |
| 3.2617902 | down | NM_177229 | chr11:62215132-62215073 | Ncor1 |
| 4.6977792 | down | NM_001039692 | chr18:6024669-6024610 | Arhgap12 |
| 3.7233718 | down | NM_173393 | chr14:64438782-64438841 | Xkr6 |
| 2.3473684 | down | NM_001033321 | chr8:114436418-114436359 | Tmem231 |
| 9.2733075 | down | AK142305 | chr9:96583698-96583639 | Zbtb38 |
| 4.4603589 | down | NM_028284 | chr2:69501119-69501178 | Bbs5 |
| 3.4259871 | down | BC027582 | chr3:103535656-103535597 | LOC269472 |
| 2.708299 | down | NM_001170591 | chr6:86978383-86978442 | Nfu1 |
| 4.4782467 | down | NM_013897 | chr9:50413114-50413173 | Timm8b |
| 2.155538 | down | NM_146027 | chr11:96895213-96895272 | Scrn2 |
| 10.0827502 | down | NM_172643 | chr1:141345338-141345397 | Zbtb41 |
| 2.2200564 | down | NM_008385 | chr4:124470593-124470652 | Inpp5b |
| 2.7767879 | down | NM_001162938 | chr1:175628376-175628435 | Pydc3 |
| 8.8411044 | down | NM_008388 | chr15:43094833-43094774 | Eif3e |
| 5.413507 | down | NM_147219 | chr11:110131022-110130963 | Abca5 |
| 2.8367185 | down | NM_011965 | chr7:121408288-121408229 | Psma1 |
| 4.1819462 | down | NM_146074 | chr17:3519449-3519390 | Tfb1m |
| 11.9260005 | down | NM_021487 | chrX:138739423-138739364 | Kcne1l |
| 2.4284605 | down |  | chr6:133711126-133711067 |  |
| 2.1162543 | down | NM_027016 | chr3:30718940-30718999 | Sec62 |
| 3.4272276 | down | NM_008980 | chr2:130379365-130379424 | Ptpra |
| 6.6129317 | down | NM_020578 | chr17:74181371-74181428 | Ehd3 |
| 10.5377489 | down | NM_029875 | chr10:117171611-117171552 | Slc35e3 |
| 3.6095958 | down | NM_013814 | chr18:24438525-24440643 | Galnt1 |
| 5.9595318 | down | NM_010284 | chr15:3268166-3268107 | Ghr |
| 5.3867309 | down | NM_001017959 | chrX:35777564-35777505 | Lamp2 |
| 4.9951379 | down | NM_024272 | chr13:91841845-91841904 | Ssbp2 |
| 4.2527154 | down | NM_007684 | chr13:81930978-81931037 | Cetn3 |
| 8.0678748 | down | NM_001081423 | chr12:87394458-87394517 | Ttll5 |
| 4.1216586 | down | NM_025486 | chr8:107852294-107852353 | Tmem208 |
| 5.719892 | down | NM_145484 | chr17:22513768-22513827 | Zfp758 |
| 2.6906561 | down | NM_001134646 | chr4:45118326-45118267 | Tomm5 |
| 15.0873278 | down | NR_033518 | chr6:8377666-8377725 | Gm16039 |
| 2.4025825 | down | NM_027978 | chr5:101083957-101083898 | Coq2 |
| 22.8446115 | down | NM_001163787 | chr9:21794376-21794317 | Ccdc151 |
| 2.7776935 | down | NM_172560 | chr11:69113805-69113746 | Cntrob |
| 2.9326258 | down | NM_010890 | chr9:72597306-72597365 | Nedd4 |
| 2.3004929 | down | NM_023733 | chr5:8966489-8966430 | Crot |
| 2.2237389 | down |  | chr6:036679629-036679688 |  |
| 8.8338718 | down | NM_001161765 | chr3:97459133-97459192 | Fmo5 |
| 6.907021 | down | NM_001048250 | chr13:108837953-108838012 | Smim15 |
| 5.383032 | down |  | chr6:128834502-128834561 |  |
| 3.5537951 | down | NM_008921 | chr10:127466952-127467011 | Prim1 |
| 4.801324 | down |  | chr8:004040039-004039980 |  |
| 2.1649618 | down |  |  |  |
| 2.1037467 | down | NM_009391 | chr5:129528961-129529020 | Ran |
| 2.0632989 | down | NM_001129999 | chr2:155824373-155824432 | Cep250 |
| 2.3270693 | down | NM_001285826 | chr1:58491235-58491176 | Ppil3 |
| 2.2553834 | down | NM_010472 | chr1:82892729-82892788 | Agfg1 |
| 3.2334955 | down | NM_009193 | chr5:33988209-33988150 | Slbp |
| 2.2160149 | down | NM_134135 | chr10:80491344-80491285 | Slc39a3 |
| 6.2846513 | down | NM_175105 | chr7:104877541-104877482 | Aqp11 |
| 2.6223635 | down | NM_172988 | chr4:22354549-22354608 | Fbxl4 |
| 23.7915803 | down | NM_027866 | chr12:29279299-29279240 | Colec11 |
| 8.7767463 | down | NR_003518 | chr11:003030755-003030814 | Pisd-ps3 |
| 2.4586886 | down |  | chr3:003792122-003792181 |  |
| 3.6915943 | down | NM_025333 | chr19:10576901-10576842 | Sdhaf2 |
| 4.8120959 | down | NM_134118 | chr8:86096454-86096302 | Tecr |
| 4.6754178 | down | NM_001113412 | chr4:95510817-95510876 | Fggy |
| 2.2654623 | down | NM_025790 | chr13:24909897-24909838 | Acot13 |
| 3.2683987 | down | NM_026124 | chr2:156699210-156699269 | 1110008F13Rik |
| 9.1438035 | down | NM_001033385 | chr10:55734196-55734137 | Tbc1d32 |
| 5.6736038 | down | NM_172865 | chr4:26251945-26251886 | Manea |
| 2.1332947 | down | NM_172993 | chr5:31783501-31783560 | Zfp512 |
| 3.7999093 | down | NM_053115 | chr14:9058200-9058141 | Acox2 |
| 2.6887843 | down | NM_027913 | chr19:10739508-10739567 | Vwce |
| 27.1943059 | down | NM_025332 | chr16:44739474-44739415 | Gtpbp8 |
| 2.3335968 | down | NM_025573 | chr5:115782747-115782806 | Srsf9 |
| 2.6664401 | down | NM_133881 | chr4:128788568-128788627 | Tmem54 |
| 2.1074098 | down | NM_019988 | chr17:24610622-24610563 | Mlst8 |
| 8.1546887 | down | NM_028992 | chr15:63723337-63723278 | Gsdmc4 |
| 4.4890682 | down | NM_030740 | chr7:5147379-5147320 | Vmn1r56 |
| 4.1549125 | down | NM_175353 | chr19:37674099-37674158 | Exoc6 |
| 8.7938913 | down | NM_010378 | chr17:34421344-34421285 | H2-Aa |
| 3.7214891 | down | NM_175343 | chr14:30851055-30851114 | Chdh |
| 2.5337829 | down | NM_175238 | chr2:51977508-51977567 | Rif1 |
| 3.3263777 | down |  | chr8:087379163-087379104 |  |
| 3.7190922 | down | NM_025615 | chr8:127363433-127363374 | 2810004N23Rik |
| 7.8850538 | down | NM_139145 | chr16:94351693-94351634 | Hlcs |
| 2.5089249 | down | NM_029320 | chr14:99642159-99642218 | Pibf1 |
| 2.1766705 | down | NM_054055 | chr2:165230855-165230796 | Slc13a3 |
| 3.4468942 | down | NM_178729 | chr5:44149541-44149482 | Fbxl5 |
| 2.1201128 | down | NM_001081286 | chr8:46137522-46137581 | Fat1 |
| 5.2185446 | down | NM_001025307 | chr19:11850302-11850243 | Stx3 |
| 16.4476401 | down | NM_001159529 | chr17:83901241-83901237 | Cox7a2l |
| 3.9322553 | down | NM_019677 | chr2:135300906-135300965 | Plcb1 |
| 2.3815371 | down |  |  |  |
| 4.3397141 | down | NM_175653 | chr13:23837387-23837328 | Hist1h3c |
| 6.4168244 | down | NM_029385 | chr9:105032559-105032500 | Nudt16 |
| 13.1587542 | down |  | chr7:132691938-132691997 |  |
| 12.097758 | down | NM_030692 | chr9:123500881-123500940 | Sacm1l |
| 3.4965545 | down | NM_001135001 | chr12:111799569-111799628 | Ppp2r5c |
| 2.8785056 | down | NM_025522 | chr12:73751504-73751445 | Dhrs7 |
| 3.712738 | down | NM_172704 | chr4:151355874-151355933 | Dnajc11 |
| 2.075949 | down | NM_009052 | chrX:132674650-132674709 | Bex1 |
| 3.72868 | down | NM_001077189 | chr1:172891047-172890988 | Fcgr2b |
| 3.0431394 | down | NM_015825 | chr16:96450476-96450535 | Sh3bgr |
| 2.8171844 | down | NM_025859 | chr10:88205888-88205947 | Arl1 |
| 3.092936 | down | NM_009197 | chrX:100892996-100892937 | Slc16a2 |
| 2.1054116 | down |  | chr19:005132022-005131963 |  |
| 2.9672031 | down | AK143696 | chr11:84685045-84685104 | Gm10374 |
| 4.4689751 | down | NM_030093 | chr11:32108717-32108776 | Snrnp25 |
| 2.8989985 | down | NM_026166 | chr10:90561290-90561349 | Ikbip |
| 2.3872257 | down | NM_146009 | chr10:100031484-100031543 | Cep290 |
| 4.8613928 | down | NM_001130476 | chr5:130611539-130611598 | Tpst1 |
| 2.1322388 | down |  | chr7:034209004-034209063 |  |
| 2.9579691 | down |  | chr1:188784519-188784578 |  |
| 2.0699673 | down | NM_001252287 | chr11:6256583-6256642 | Ogdh |
| 3.8561896 | down | NM_153152 | chr17:56748350-56748291 | 2410015M20Rik |
| 7.2323069 | down | NM_026940 | chr16:17148730-17148789 | Ydjc |
| 3.1024737 | down | NM_173376 | chrX:46063393-46063452 | Rbmx2 |
| 2.5271285 | down | NM_134020 | chr11:6171171-6171112 | Tmed4 |
| 8.7513889 | down | NM_007861 | chr12:32016948-32016889 | Dld |
| 24.9513436 | down | NM_178763 | chr11:121372729-121372670 | Zfp750 |
| 2.3074513 | down | NM_010273 | chrX:71556167-71556226 | Gdi1 |
| 2.0591858 | down | XM_001476653 | chr13:88618267-88618326 | Gm8526 |
| 9.6490105 | down | NM_177889 | chr7:30841130-30841071 | Zfp82 |
| 5.5902946 | down | NM_011638 | chr16:32632255-32632314 | Tfrc |
| 2.0144765 | down |  | chr18:030768548-030768489 |  |
| 2.3602669 | down | NM_178713 | chr10:21116106-21116165 | Aldh8a1 |
| 3.8461193 | down | NM_008288 | chr1:195048228-195048169 | Hsd11b1 |
| 6.8060417 | down |  |  |  |
| 2.3757608 | down | NM_001284414 | chr11:101118207-101118266 | Vps25 |
| 2.2251466 | down |  | chr11:109021233-109021174 |  |
| 4.4647587 | down | NM_025579 | chr4:131848802-131848861 | Taf12 |
| 21.1203333 | down | NM_001011790 | chr11:49349459-49349518 | Olfr1382 |
| 3.0515736 | down | NM_010590 | chr14:55186871-55186812 | Ajuba |
| 3.195102 | down | NM_009906 | chr7:112893591-112893532 | Tpp1 |
| 4.0253856 | down | NM_008160 | chr9:108242276-108242335 | Gpx1 |
| 8.0912057 | down | NM_007897 | chr11:44818438-44818497 | Ebf1 |
| 2.0897727 | down | NM_016742 | chr9:20943976-20943917 | Cdc37 |
| 5.3248194 | down | NM_199366 | chr1:95773011-95773070 | Gal3st2 |
| 6.4380764 | down | NM_025693 | chr16:21934459-21934400 | Tmem41a |
| 2.8111433 | down | NM_153415 | chr12:88448130-88448071 | Pomt2 |
| 6.1659073 | down | NM_019821 | chr5:115120158-115120099 | Gltp |
| 2.4454187 | down |  | chr14:121226736-121226795 |  |
| 2.3446848 | down | NM_001177505 | chr9:122805131-122805190 | Zkscan7 |
| 5.2769792 | down | NM_029132 | chr10:41438803-41438744 | Cep57l1 |
| 3.6973202 | down | NM_012056 | chr6:56829199-56829258 | Fkbp9 |
| 2.7536223 | down | NM_025789 | chr17:8139154-8145458 | Rsph3a |
| 3.2475182 | down | NM_010129 | chr7:53175391-53175332 | Emp3 |
| 2.0170488 | down | NM_001109747 | chr10:29918343-29918284 | Cenpw |
| 2.0323741 | down |  | chrX:077911694-077911753 |  |
| 3.3906621 | down | NM_024446 | chr8:116676071-116676130 | Nudt7 |
| 5.5870278 | down |  | chr5:149301721-149301662 |  |
| 11.7698547 | down |  | chr5:59445732-59445791 |  |
| 3.3605142 | down | NM_134014 | chr11:23196643-23196702 | Xpo1 |
| 2.317546 | down | NM_008564 | chr6:88833678-88833619 | Mcm2 |
| 3.3983421 | down | NM_026430 | chr1:43807185-43807126 | Uxs1 |
| 13.0171309 | down | NM_028491 | chr10:67894034-67893975 | 1700040L02Rik |
| 2.585288 | down | NM_029128 | chr16:43861940-43861881 | Qtrtd1 |
| 2.6700745 | down | NM_016905 | chr11:115870183-115870124 | Galk1 |
| 2.0124961 | down | NM_008783 | chr1:170121514-170121455 | Pbx1 |
| 2.974004 | down | NM_028977 | chr5:21081617-21081676 | Lrrc17 |
| 11.7201311 | down | NM_025449 | chr9:108198138-108198197 | Nicn1 |
| 14.8743477 | down | NM_001081237 | chr6:147060976-147061035 | Klhl42 |
| 2.6737096 | down | CO811977 | chr15:78876096-78876037 |  |
| 3.9612424 | down | NM_001177869 | chr13:38043797-38043856 | Rreb1 |
| 2.4304105 | down |  | chr4:117498236-117498177 |  |
| 2.3953431 | down | NM_153780 | chr5:020857448-020857507 | 2610044O15Rik8 |
| 24.0295446 | down | NM_026077 | chr7:88933417-88933358 | 3110040N11Rik |
| 2.4615039 | down | NM_027927 | chr3:132771999-132772058 | Ints12 |
| 2.8285665 | down | NM_009654 | chr5:90901598-90901657 | Alb |
| 8.5144303 | down | NM_175389 | chr3:137820397-137820456 | Trmt10a |
| 12.0474174 | down | NM_144521 | chr11:59221225-59221166 | Snap47 |
| 3.1302952 | down |  | chr16:078251964-078252023 |  |
| 4.2159609 | down | NM_019819 | chr11:83861975-83861916 | Dusp14 |
| 5.5839224 | down | NM_023858 | chr9:13610046-13610105 | Mtmr2 |
| 2.0468253 | down | NM_010911 | chr2:155949548-155949489 | Nfs1 |
| 3.8169646 | down | NM_175106 | chr1:121806790-121806731 | Tmem177 |
| 14.5813562 | down | NM_153581 | chr8:56145539-56145598 | Gpm6a |
| 2.5318554 | down | NM_144902 | chr3:116373795-116373736 | Slc35a3 |
| 2.1542161 | down |  | chr14:031937823-031937764 |  |
| 2.9941272 | down | NM_180600 | chr9:55055069-55055128 | Ube2q2 |
| 9.1645129 | down | NM_011629 | chr10:93657981-93658040 | Nr2c1 |
| 3.7094194 | down | NM_020584 | chr8:114542435-114542494 | Terf2ip |
| 2.4522159 | down | BB376197 | chr12:112037212-112037271 | 2900016J10Rik |
| 11.082995 | down | NM_013830 | chr13:34991032-34991091 | Prpf4b |
| 3.6114978 | down |  | chrX:018498047-018498106 |  |
| 8.307674 | down | NM_025839 | chr16:4940543-4940602 | Nudt16l1 |
| 6.372403 | down | NM_011959 | chr5:21992996-21992937 | Orc5 |
| 3.9843558 | down | NM_001270426 | chr3:92762371-92762430 | Lce3d |
| 2.9120045 | down | NM_198003 | chr17:22593580-22593639 | Zfp946 |
| 3.4048282 | down | NM_011936 | chr8:94191851-94191910 | Fto |
| 24.0258339 | down | NM_010952 | chr9:65535779-65536685 | Oaz2 |
| 2.8723151 | down |  | chr1:029541754-029541695 |  |
| 12.8074983 | down |  | chr6:147022866-147022807 |  |
| 7.5469933 | down |  | chr1:165553900-165553959 |  |
| 2.8839847 | down | NM_001190179 | chr2:80498803-80498862 | Nup35 |
| 3.2015599 | down | NM_009211 | chr9:110140717-110140776 | Smarcc1 |
| 6.2612383 | down | NM_145381 | chr1:13616951-13616892 | Lactb2 |
| 2.9470722 | down | NM_008860 | chr4:154636816-154636587 | Prkcz |
| 2.6665114 | down | AK015947 | chr17:8335348-8335289 | Rnaset2a |
| 7.3924778 | down | NM_153600 | chr6:38377142-38377201 | Ttc26 |
| 2.1745743 | down | NM_009730 | chr2:130855762-130855821 | Atrn |
| 4.5639121 | down | NM_019936 | chr17:87434906-87434965 | Cript |
| 3.4172453 | down |  | chr1:137129554-137129613 |  |
| 2.1355401 | down | NM_172511 | chr16:45731591-45731532 | Abhd10 |
| 4.390974 | down | NM_008303 | chr1:55148026-55148085 | Hspe1 |
| 2.163826 | down | NM_019648 | chr10:93324269-93324210 | Metap2 |
| 2.2510233 | down | NM_001190448 | chr11:11714165-11714106 | Ddc |
| 3.7050777 | down | NM_133851 | chr2:119475072-119475131 | Nusap1 |
| 3.8973822 | down | NM_172530 | chr3:89662699-89662758 | She |
| 3.0195961 | down | NM_001136073 | chr2:168302033-168301974 | Nfatc2 |
| 3.1278883 | down | NM_001167885 | chr19:3816303-3816362 | Suv420h1 |
| 4.0702178 | down |  | chr12:069191971-069192030 |  |
| 13.3363873 | down |  | chr12:003521887-003521828 |  |
| 4.4504358 | down | NM_177333 | chr13:74319735-74319594 | Exoc3 |
| 9.2672468 | down |  | chr17:036378350-036378409 |  |
| 3.0432199 | down |  | chrX:112103521-112103580 |  |
| 2.0823593 | down | NM_007798 | chr14:63761906-63761965 | Ctsb |
| 2.1727196 | down | NM_021437 | chr14:55309373-55309432 | 1700123O20Rik |
| 10.8287349 | down | NM_010294 | chr5:97884608-97884549 | Gk2 |
| 2.6582806 | down |  | chr4:012043536-012043595 |  |
| 2.9658322 | down | NM_133768 | chr5:130488549-130487964 | Asl |
| 3.4133735 | down | NM_026247 | chrX:140759482-140759541 | Alg13 |
| 2.0413917 | down | NM_145930 | chr15:3932285-3932226 | AW549877 |
| 2.0432206 | down | NM_007743 | chr6:4490619-4490678 | Col1a2 |
| 10.0592964 | down | NM_011885 | chr7:29524896-29524837 | Mrps12 |
| 2.4328409 | down | NM_008439 | chr5:31233555-31233614 | Khk |
| 7.7968479 | down | NM_010299 | chr11:54923856-54923915 | Gm2a |
| 7.5427337 | down | NM_001004139 | chr7:46795649-46795708 | Zfp619 |
| 6.0627948 | down | NM_028123 | chr6:39286149-39286090 | Slc37a3 |
| 2.188548 | down | NM_133254 | chr7:135415884-135415943 | Slc5a2 |
| 2.589228 | down | NM_172671 | chr2:109853567-109853626 | Lgr4 |
| 5.3171346 | down | NM_027857 | chr19:3989878-3989937 | Acy3 |
| 4.3184782 | down | NM_177583 | chr9:66627158-66627100 | Aph1b |
| 9.1866852 | down | NM_027078 | chr10:90565191-90565250 | Ikbip |
| 3.3489156 | down | NM_001285467 | chr14:27451144-27451203 | Dennd6a |
| 4.2834121 | down | NM_001081242 | chr9:67066002-67065943 | Tln2 |
| 2.5818371 | down | NM_028247 | chr10:39795785-39795726 | Slc16a10 |
| 12.398305 | down | NM_023596 | chr10:60175710-60175651 | Slc29a3 |
| 2.7054152 | down | NM_024216 | chr5:122832557-122832616 | Gpn3 |
| 2.7317694 | down | NM_053142 | chr18:37647758-37647817 | Pcdhb17 |
| 2.0804491 | down | NM_001285805 | chr16:43619030-43619089 | Zbtb20 |
| 30.3989882 | down | NM_183028 | chr1:7160119-7160178 | Pcmtd1 |
| 4.9937015 | down | NM_001252193 | chr11:109296007-109296066 | Amz2 |
| 9.7328634 | down | NM_001243792 | chr1:190038384-190038443 | Esrrg |
| 6.0659727 | down |  | chr6:149382050-149381991 |  |
| 2.0484098 | down | NM_001004721 | chr2:155104164-155104105 | Pigu |
| 13.4237942 | down | NM_174990 | chr6:48641817-48641876 | Gimap4 |
| 3.7635481 | down | NM_026164 | chr12:45414232-45414291 | Pnpla8 |
| 6.5275758 | down | BC126859 | chr14:53540971-53541030 | Trav9n-2 |
| 4.4186958 | down | NM_080448 | chr6:112668298-112668239 | Srgap3 |
| 5.8648532 | down | NM_029942 | chr18:42072068-42040925 | Prelid2 |
| 8.3071638 | down | NM_001013820 | chr19:8137970-8137911 | Slc22a28 |
| 3.485513 | down |  | chr11:009637489-009637430 |  |
| 13.2063392 | down | NM_007532 | chr6:144948455-144948396 | Bcat1 |
| 6.3714799 | down | NM_027715 | chr2:19581254-19581313 | Otud1 |
| 3.0012656 | down | NM_001009818 | chr5:93603881-93603940 | Sept11 |
| 3.2055924 | down | NM_026406 | chr3:96595019-96595078 | Rnf115 |
| 2.1819536 | down | NR_000040 | chr10:87430357-87430416 | Tyms-ps |
| 6.6379548 | down | NM_134420 | chr9:108763641-108763700 | Slc26a6 |
| 2.2409446 | down | NM_010771 | chr18:35750212-35750271 | Matr3 |
| 3.014931 | down | NM_172679 | chr3:36947563-36949736 | 4932438A13Rik |
| 5.2238845 | down |  | chr11:012836736-012836677 |  |
| 2.5047073 | down | NR_002883 | chr15:103075121-103075180 | Gm5643 |
| 2.9006783 | down | NM_008065 | chr16:84860691-84860750 | Gabpa |
| 12.1292631 | down | NM_178739 | chrX:42139819-42139760 | Dcaf12l1 |
| 4.3178368 | down | NM_011130 | chr8:23747619-23747560 | Polb |
| 2.7851171 | down | NM_153566 | chr4:124531204-124531721 | Yrdc |
| 6.2701067 | down | NM_024178 | chr3:121064697-121064756 | Alg14 |
| 2.3674197 | down | NM_030215 | chr13:32914158-32914217 | Wrnip1 |
| 11.105843 | down | NM_026935 | chr17:53969264-53969205 | Sult1c2 |
| 3.2413122 | down | NM_001085385 | chr7:38979930-38979989 | 1600014C10Rik |
| 21.2685754 | down | NM_174846 | chr9:106055957-106055898 | Glyctk |
| 8.17447 | down | NM_001284219 | chr13:59567454-59567395 | Agtpbp1 |
| 5.5199898 | down | AK151379 | chr13:105920794-105920735 | Rnf180 |
| 3.5644142 | down | NM_134084 | chr14:26519434-26519493 | Ppif |
| 2.2574776 | down | NM_001195633 | chr18:78231399-78231458 | Epg5 |
| 2.1551197 | down | NM_010305 | chr5:17771136-17771077 | Gnai1 |
| 2.2322438 | down | NM_030703 | chr19:44040784-44040725 | Cpn1 |
| 2.3484486 | down | NM_027371 | chr3:146170531-146170157 | Rpf1 |
| 5.2947736 | down |  | chrX:071812943-071813002 |  |
| 5.8467386 | down | NM_001163145 | chr15:84209705-84209646 | 1810041L15Rik |
| 2.3615057 | down |  | chrX:149344004-149343945 |  |
| 8.1079242 | down | NM_138677 | chr6:108808920-108808979 | Edem1 |
| 2.4343422 | down | NM_025278 | chr6:66970551-66970610 | Gng12 |
| 6.6287599 | down | NM_172621 | chr17:44416979-44417038 | Clic5 |
| 9.6019585 | down | NR_030695 | chr4:141103547-141103606 | B330016D10Rik |
| 5.9249673 | down | NM_023434 | chr14:52913392-52913451 | Tox4 |
| 2.3618041 | down | AK017416 | chr17:095122116-095122057 | 5430439M09Rik |
| 2.3089933 | down |  | chr18:045444959-045444900 |  |
| 3.6646207 | down | NM_031878 | chr11:106124899-106124840 | Smarcd2 |
| 2.000474 | down | NM_080289 | chr4:44995759-44995818 | Grhpr |
| 2.8407089 | down |  | chr4:147442068-147442009 |  |
| 2.5744808 | down | NM_019791 | chrX:91781204-91780963 | Maged1 |
| 4.1306362 | down |  | chr12:012030702-012030761 |  |
| 2.8067421 | down | NM_011991 | chr11:59634853-59633613 | Cops3 |
| 3.7293469 | down | NM_153163 | chr6:23213379-23213320 | Cadps2 |
| 7.9872435 | down | NM_015829 | chr6:5991541-5991482 | Slc25a13 |
| 3.5439764 | down | NM_008682 | chr10:92147811-92147752 | Nedd1 |
| 5.1704864 | down | NM_031375 | chr7:87410168-87410227 | Ngrn |
| 3.9554252 | down | NM_011938 | chr13:55560861-55560920 | Grk6 |
| 3.938633 | down | NM_010240 | chr7:52714495-52714014 | Ftl1 |
| 2.30306 | down | NM_024172 | chr7:4612411-4612352 | Hspbp1 |
| 14.634044 | down | NM_001284394 | chr11:87402710-87402769 | Sept4 |
| 4.3449953 | down | NM_023844 | chr16:84824952-84825011 | Jam2 |
| 29.2369558 | down | NM_009790 | chr12:101447508-101447567 | Calm1 |
| 3.6523196 | down | NM_025868 | chr2:84511635-84511576 | Tmx2 |
| 2.0697507 | down | NM_028181 | chr9:72860771-72863169 | Ccpg1 |
| 2.4783616 | down | AK140131 | chr1:100490826-100490885 | Gm7967 |
| 3.2290391 | down | NM_001115010 | chr5:100871214-100871155 | Lin54 |
| 9.7833395 | down | NM_198024 | chr15:8996792-8996851 | Ranbp3l |
| 2.6554677 | down | XR_105590 | chr13:115881136-115881195 | Gm10734 |
| 3.6629043 | down | NM_013587 | chr5:35434150-35434140 | Lrpap1 |
| 3.507713 | down | NM_001113569 | chr2:32643577-32643518 | Stxbp1 |
| 2.1926203 | down | NM_016771 | chr5:87985067-87985008 | Sult1d1 |
| 2.4778539 | down | NR_024139 | chr3:110049249-110049190 | Prmt6 |
| 10.9175883 | down | NM_026635 | chr9:65986244-65986303 | Fam96a |
| 3.1169217 | down | NM_177684 | chr6:117795365-117795424 | Zfp637 |
| 2.4184014 | down | NM_008532 | chr17:88048972-88049800 | Epcam |
| 4.0097019 | down | NM_011760 | chr17:21571882-21571941 | Zfp54 |
| 4.6795841 | down | NM_026393 | chr16:4711429-4711370 | Nmral1 |
| 4.0340781 | down |  | chr1:043946026-043946085 |  |
| 2.9316238 | down | NM_024478 | chr5:36814105-36814164 | Grpel1 |
| 7.724813 | down | NM_009360 | chr10:70691162-70691103 | Tfam |
| 3.2158981 | down | NM_172124 | chr1:23854630-23854689 | B3gat2 |
| 5.9180259 | down | NM_023526 | chr14:19110960-19111019 | Nkiras1 |
| 2.3364862 | down | BC019449 | chr5:3643283-3643224 | Gatad1 |
| 9.205287 | down | NM_019397 | chrX:162961651-162961592 | Egfl6 |
| 4.2875084 | down |  | chr1:091153410-091153351 |  |
| 3.7607873 | down | NM_007683 | chr5:86445276-86442526 | Cenpc1 |
| 3.9225858 | down | NM_012054 | chr13:21116062-21116121 | Aoah |
| 2.2229637 | down | NM_001081093 | chr3:84300044-84299985 | Arfip1 |
| 4.7126482 | down | NM_025894 | chr11:107347092-107347728 | Psmd12 |
| 12.1896492 | down | NM_001172068 | chr9:59582129-59582070 | Senp8 |
| 2.8939195 | down | NM_173182 | chr3:27315788-27315729 | Fndc3b |
| 30.7124777 | down | NM_181401 | chr4:15211620-15211679 | Tmem64 |
| 2.1943198 | down | NM_175266 | chr9:111178556-111178615 | Epm2aip1 |
| 2.6602944 | down | NM_001033337 | chr15:85686984-85687043 | Ttc38 |
| 4.337342 | down | NM_146016 | chr11:29643506-29643447 | Eml6 |
| 8.067966 | down | NM_016675 | chrX:136345244-136345303 | Cldn2 |
| 3.9134049 | down | NM_019921 | chr11:61685531-61685472 | Akap10 |
| 30.2102748 | down | NM_026142 | chr14:50301767-50301708 | 3632451O06Rik |
| 3.2219096 | down | NM_133220 | chr1:9892452-9892511 | Sgk3 |
| 2.4080027 | down | NM_001099917 | chr10:80857597-80857656 | 2210404O07Rik |
| 8.5651327 | down | NM_026184 | chr13:12701235-12701294 | Ero1lb |
| 2.3552333 | down | NM_001039214 | chr18:73751801-73751860 | Mex3c |
| 2.2048339 | down | NM_009438 | chr7:52381205-52381146 | Rpl13a |
| 9.6585097 | down | NM_009139 | chr11:83402056-83401997 | Ccl6 |
| 4.8327997 | down | NM_029402 | chr18:3436224-3436283 | Cul2 |
| 7.8804608 | down | NM_008304 | chr15:32964204-32964263 | Sdc2 |
| 2.7501283 | down | NM_028427 | chr8:67488190-67489455 | Tmem192 |
| 4.3818558 | down | NM_146108 | chr1:52977535-52977594 | Hibch |
| 5.0279263 | down | NM_145497 | chr19:18725771-18725830 | Nmrk1 |
| 3.9549534 | down | NM_138656 | chr8:124957809-124957750 | Mvd |
| 2.094641 | down | NM_020619 | chr6:83068810-83068869 | Mogs |
| 5.5292201 | down | NM_011405 | chr14:54989225-54989166 | Slc7a7 |
| 2.1231179 | down |  | chr7:127015737-127015796 |  |
| 2.1563465 | down | NM_144948 | chr9:48297494-48297435 | Rbm7 |
| 2.5953102 | down | NM_001045529 | chr16:93875950-93876009 | Morc3 |
| 2.0241315 | down |  | chr14:028024737-028024796 |  |
| 2.0066825 | down | NM_001080129 | chr10:90612075-90612016 | Tmpo |
| 2.1779845 | down | NM_029022 | chr2:73169062-73169121 | Scrn3 |
| 3.2976649 | down | AK087205 | chr17:23888115-23888174 | 9530082P21Rik |
| 4.8476986 | down | NM_011327 | chr4:107716981-107716922 | Scp2 |
| 19.6405633 | down | NM_001005847 | chr8:54601320-54601379 | Aga |
| 2.0305274 | down | NM_172405 | chr5:101234977-101234918 | Fam175a |
| 8.1098914 | down | NM_175089 | chr8:63608972-63609031 | Nek1 |
| 3.2729453 | down | NM_173742 | chr11:70052440-70051914 | Rnasek |
| 2.9298768 | down | NM_138745 | chr12:77418622-77418681 | Mthfd1 |
| 2.5214594 | down | NM_019799 | chr7:86738337-86738278 | Rhcg |
| 3.4722215 | down | NM_009031 | chrX:159216593-159216652 | Rbbp7 |
| 2.1490078 | down | NM_011066 | chr1:93313105-93313046 | Per2 |
| 2.1949347 | down | NM_008907 | chr11:6319737-6319796 | Ppia |
| 2.3259299 | down | NM_026486 | chr5:125077404-125077463 | Tctn2 |
| 2.0209687 | down | NM_009186 | chr16:22246501-22246442 | Tra2b |
| 2.995718 | down | NM_172763 | chr9:22047517-22047576 | Zfp809 |
| 7.7944742 | down | NM_016796 | chr1:164519597-164519656 | Vamp4 |
| 3.0881418 | down | NM_172707 | chr5:32794896-32794955 | Ppp1cb |
| 4.6498965 | down | NM_030098 | chr14:51750572-51750631 | Rnase6 |
| 17.6192902 | down | NM_178799 | chr5:122057221-122055701 | Acad12 |
| 3.0484207 | down | NM_023136 | chr1:95697140-95697081 | Dtymk |
| 3.411412 | down | NM_052993 | chr6:7821076-7821135 | C1galt1 |
| 4.167459 | down | NM_001199045 | chr11:86380802-86380861 | Tubd1 |
| 7.3860243 | down | NM_031198 | chr6:16783779-16783720 | Tfec |
| 2.2301509 | down |  | chr4:069515732-069515791 |  |
| 3.4448448 | down | NM_019542 | chr6:83752479-83752538 | Nagk |
| 2.1876895 | down | NM_009275 | chr9:103092294-103092235 | Srprb |
| 2.3413518 | down | NM_172568 | chr11:105222353-105222294 | March10 |
| 4.1862167 | down | AK089244 | chr8:108508909-108508850 | Dpep2 |
| 5.2851521 | down | NM_153578 | chr7:63233936-63233877 | Nipa1 |
| 5.8536322 | down | NM_001004066 | chr12:117298066-117298125 | Zfp386 |
| 3.6694301 | down | NM_011518 | chr13:52744088-52744147 | Syk |
| 4.457306 | down | NM_022889 | chr11:3879948-3880007 | Pes1 |
| 2.1951659 | down |  | chr7:034208926-034208985 |  |
| 4.8463843 | down | NM_008892 | chrX:90550248-90550189 | Pola1 |
| 2.9628508 | down | NM_145520 | chr2:29632188-29632129 | Trub2 |
| 4.5097289 | down | NM_018821 | chr18:89037357-89037298 | Socs6 |
| 2.16102 | down | AK133405 | chr8:28730840-28730899 | Gm1698 |
| 5.6675826 | down | NM_145524 | chr2:70811409-70811350 | Mettl8 |
| 2.3692214 | down | AK030082 | chr4:69586288-69586229 | Gm11223 |
| 3.0269755 | down | NM_019535 | chr4:85034126-85034185 | Sh3gl2 |
| 2.4684818 | down | NM_007479 | chr14:27475919-27475978 | Arf4 |
| 2.2306912 | down | NM_023596 | chr10:60177963-60177904 | Slc29a3 |
| 3.2030748 | down | NM_011656 | chr3:94417239-94417180 | Tuft1 |
| 2.6779232 | down | NM_008905 | chr7:114887808-114887867 | Ppfibp2 |
| 2.0056808 | down | NM_009462 | chr8:122481397-122481456 | Usp10 |
| 3.5524516 | down |  | chr10:062883906-062883847 |  |
| 3.860477 | down | NM_144911 | chr5:108062090-108062149 | Rpap2 |
| 3.3745286 | down | NM_028862 | chr11:44378746-44378805 | Rnf145 |
| 2.4876155 | down |  | chr3:95214936-95214877 |  |
| 5.1084528 | down | NM_025633 | chr2:71349557-71349616 | Metap1d |
| 14.5616244 | down | NM_029331 | chr6:85849453-85849394 | 1700019G17Rik |
| 11.9812124 | down | NM_001285893 | chr16:21581711-21581770 | Vps8 |
| 5.7530101 | down | NM_029565 | chr4:106873162-106873221 | Tmem59 |
| 3.4124918 | down | NM_021512 | chr2:90576065-90576124 | Nup160 |
| 2.5259796 | down | NM_177448 | chr7:106367660-106367601 | Mogat2 |
| 23.0715112 | down | AK043982 | chr15:102649907-102649966 |  |
| 4.0489112 | down | NM_026011 | chr6:108773239-108773298 | Arl8b |
| 22.0330452 | down | NM_001099297 | chr2:122173491-122173550 | Duox1 |
| 2.7784841 | down | NM_145144 | chr2:31828806-31828865 | Aif1l |
| 17.684713 | down | NM_172574 | chr12:16995781-16995722 | Pqlc3 |
| 3.1332003 | down | NM_025443 | chr11:17103591-17103532 | Pno1 |
| 22.0954836 | down |  | chr4:151531203-151531144 |  |
| 61.8034385 | down | NM_175293 | chr1:65154192-65154133 | D630023F18Rik |
| 7.4078352 | down | NM_029760 | chr12:53411797-53411855 | Nubpl |
| 3.0478777 | down | NM_010178 | chr4:135425311-135425370 | Srsf10 |
| 19.3595753 | down | NM_080445 | chr4:155365344-155365285 | B3galt6 |
| 3.1541538 | down | NM_177167 | chr11:87040547-87040488 | Ppm1e |
| 2.1925352 | down |  | chr13:022768623-022768682 |  |
| 4.4528253 | down | NM_010763 | chr3:100370325-100370266 | Man1a2 |
| 5.9858192 | down | NM_023635 | chr9:72944668-72944727 | Rab27a |
| 4.0563215 | down | NM_010231 | chr1:164760096-164760037 | Fmo1 |
| 3.9967463 | down | NM_013902 | chr12:66163656-66163597 | Fkbp3 |
| 2.3469726 | down | NM_025596 | chr13:55426360-55426419 | Prelid1 |
| 10.0628984 | down | NM_029370 | chr2:164652952-164652893 | Spata25 |
| 21.0162112 | down | NM_019810 | chr5:33504561-33504620 | Slc5a1 |
| 7.8509106 | down | NM_001085549 | chr4:114282852-114282911 | Trabd2b |
| 3.386489 | down | NM_172470 | chr12:9035023-9035082 | Wdr35 |
| 5.2628672 | down | NM_011341 | chr4:155374811-155374870 | Sdf4 |
| 2.2820734 | down | NM_027695 | chr14:17073333-17073274 | Oxsm |
| 16.5688481 | down | NM_001048208 | chrX:12252668-12252609 | Med14 |
| 2.867193 | down | NM_010799 | chr19:32589734-32589793 | Minpp1 |
| 2.4246357 | down | NM_001244891 | chr2:90857133-90857192 | Celf1 |
| 12.8900504 | down | NM_053078 | chr18:33597357-33597298 | Nrep |
| 3.3002857 | down | AK151738 | chr8:118207332-118207273 | Maf |
| 2.5552156 | down | NM_013546 | chr6:135087829-135087770 | Hebp1 |
| 3.770699 | down | NM_009797 | chr3:104637024-104631466 | Capza1 |
| 6.0285745 | down | NM_011930 | chr17:25298348-25298407 | Clcn7 |
| 3.1117764 | down | NM_001142744 | chr17:36034617-36034558 | Atat1 |
| 3.3278195 | down | NM_008664 | chr8:15133322-15133381 | Myom2 |
| 3.5973911 | down | NM_026013 | chr3:106375918-106375977 | Dram2 |
| 3.6678527 | down | NM_201358 | chr13:36071561-36071502 | Lyrm4 |
| 3.2007189 | down | NM_007497 | chr15:100090547-100090606 | Atf1 |
| 5.2660879 | down | NM_145398 | chr6:4592743-4592802 | Casd1 |
| 2.4641364 | down | AK031957 | chrX:19676083-19676024 | 6330509M05Rik |
| 2.8370605 | down | NM_153556 | chr1:53246192-53246133 | Pms1 |
| 3.5862722 | down | NR_027965 | chr16:55973524-55973465 | 2310061J03Rik |
| 2.389189 | down | NM_010570 | chr1:82229751-82229692 | Irs1 |
| 2.267243 | down | NM_001033165 | chr4:59035628-59035687 | Dnajc25 |
| 5.4063466 | down |  | chr16:015609688-015609747 |  |
| 4.1706016 | down | NM_001001447 | chr7:13493532-13493588 | Zscan22 |
| 12.389995 | down | NM_001001881 | chr16:21649873-21649814 | 2510009E07Rik |
| 4.6510192 | down | NM_145619 | chr9:106372802-106372743 | Parp3 |
| 4.5071189 | down | NM_030179 | chr17:72207569-72207628 | Clip4 |
| 2.479369 | down | NM_181588 | chr15:31519707-31519766 | Cmbl |
| 13.7284134 | down |  | chr8:099688735-099688794 |  |
| 2.2793698 | down | NM_025531 | chr2:174290694-174290635 | Slmo2 |
| 3.9919941 | down | NM_001243762 | chrX:6736654-6736595 | Clcn5 |
| 2.5848031 | down | NM_026250 | chr1:99689283-99689342 | Gin1 |
| 3.9617787 | down | NM_017475 | chr4:123613729-123613788 | Rragc |
| 13.7544639 | down | NM_134131 | chr18:50250360-50250419 | Tnfaip8 |
| 2.0461374 | down | NM_025596 | chr13:55426573-55426632 | Prelid1 |
| 3.6754449 | down | NM_178375 | chr2:164647373-164647432 | Zswim3 |
| 4.6253136 | down | NM_001142744 | chr17:36045568-36045509 | Atat1 |
| 4.2171578 | down | NM_001004436 | chr14:35558984-35559043 | Wapal |
| 5.1850726 | down | NM_001085501 | chr2:178146053-178145994 | Ppp1r3d |
| 4.9939522 | down | NR_027388 | chr5:25037222-25037281 | 1700096K18Rik |
| 9.0734675 | down | NM_207633 | chrX:96136136-96138143 | Yipf6 |
| 3.3397021 | down | NM_001081176 | chr13:81813062-81813003 | Polr3g |
| 2.3985658 | down | NM_001009978 | chr2:79705260-79705201 | Pde1a |
| 47.6220823 | down | NM_016900 | chr6:17237331-17237390 | Cav2 |
| 2.487371 | down | NM_182996 | chr11:58127493-58127552 | Zfp692 |
| 2.7452931 | down | AK164389 | chr8:19990652-19990593 |  |
| 3.1773874 | down | NM_026487 | chr19:32747459-32747400 | Atad1 |
| 6.2156088 | down | NM_183270 | chr7:107688028-107688087 | Coa4 |
| 2.9678414 | down | NM_201410 | chr1:90115493-90115552 | Ugt1a6b |
| 4.5245945 | down | NM_144551 | chr12:15799068-15799009 | Trib2 |
| 2.1425714 | down | NM_172473 | chr10:45431984-45432043 | Hace1 |
| 2.4175471 | down | NM_011119 | chr10:127995361-127995302 | Pa2g4 |
| 5.0536789 | down | NM_026365 | chr6:113397928-113397987 | Jagn1 |
| 2.2274001 | down | NM_029354 | chr16:15863063-15863004 | Mzt2 |
| 4.2565808 | down | NM_008663 | chr7:105199896-105199837 | Myo7a |
| 5.4704845 | down | NM_028355 | chr4:107086727-107086786 | Ndc1 |
| 7.2106059 | down | NM_027861 | chr1_random:249711-249690 | Brox |
| 3.3378226 | down | NM_001163833 | chr10:55836569-55836628 | Msl3l2 |
| 2.7571658 | down | NM_001177535 | chr10:103633507-103633566 | Gm4340 |
| 2.5665654 | down | NM_026009 | chr11:106060995-106060936 | Ccdc47 |
| 3.4386636 | down | NM_178603 | chr4:49525661-49525602 | Mrpl50 |
| 2.3221303 | down | NM_021365 | chrX:70467477-70467536 | Xlr4b |
| 2.0362067 | down | NM_026572 | chr8:119505931-119505872 | Gcsh |
| 3.029631 | down | NM_025952 | chrX:103174801-103174745 | Magt1 |
| 2.7082501 | down | NM_026281 | chr6:146552138-146552079 | Tm7sf3 |
| 3.07609 | down | NM_001035854 | chr11:83217993-83218052 | Ap2b1 |
| 2.4935705 | down | NM_207523 | chr11:77995005-77994946 | Rpl23a |
| 2.7269452 | down | NM_009499 | chr7:19842914-19842855 | Vasp |
| 4.5299254 | down | NM_023503 | chr8:48753301-48753242 | Ing2 |
| 3.7202506 | down |  | chrX:071253437-071253496 |  |
| 3.2453498 | down | NM_172474 | chr3:154240222-154240163 | Tyw3 |
| 3.0353004 | down | NM_172493 | chrX:127000133-127000192 | Diap2 |
| 2.2453622 | down | NM_026032 | chr8:26912646-26912705 | Lsm1 |
| 15.0422899 | down | AK139221 | chr9:31138009-31138068 | Prdm10 |
| 9.6943247 | down | NM_031494 | chrX:70603891-70603950 | Zfp275 |
| 4.3962274 | down | NM_147006 | chr11:73627771-73627712 | Olfr392 |
| 5.9992149 | down | NM_009303 | chr15:79941040-79941099 | Syngr1 |
| 2.2702977 | down | NM_001025384 | chrX:70382654-70382596 | DXBay18 |
| 2.5409377 | down | NM_173756 | chr12:85872287-85872346 | Lin52 |
| 2.1495192 | down | NM_175277 | chr6:83308249-83308308 | Bola3 |
| 2.1389035 | down | NM_021716 | chr2:63816027-63815968 | Fign |
| 3.4933793 | down | NM_026861 | chr14:122419871-122419930 | Ubac2 |
| 5.1596207 | down | NM_001199177 | chr16:29654447-29654506 | Opa1 |
| 2.2360924 | down | NM_199062 | chr10:81366683-81366624 | Zfp781 |
| 2.4549421 | down | NM_001252055 | chr15:74875554-74875495 | Ly6c1 |
| 5.3060968 | down | NM_023154 | chr7:25393395-25393454 | Ethe1 |
| 2.7194276 | down | NM_016750 | chr3:137529723-137529782 | H2afz |
| 5.2374479 | down | NM_010156 | chr6:3322579-3322520 | Samd9l |
| 2.0939638 | down | NM_025507 | chr12:88791047-88790988 | Snw1 |
| 2.441541 | down | NM_172762 | chr8:129471164-129471105 | Rbm34 |
| 7.6955509 | down | NM_027959 | chr12:17291323-17291382 | Pdia6 |
| 20.9804643 | down | NM_026679 | chr14:26743531-26743472 | Tmem254a |
| 2.201311 | down | NM_145599 | chr8:80120321-80120262 | Tmem184c |
| 3.7348249 | down | NM_011818 | chr6:86641973-86641914 | Gmcl1 |
| 8.6349798 | down | NM_001012723 | chr2:164460461-164460402 | Wfdc16 |
| 3.9187187 | down | NM_025839 | chr16:4940856-4940915 | Nudt16l1 |
| 3.9877855 | down | NM_028883 | chr8:122284595-122284536 | Tldc1 |
| 2.1272359 | down | BC020359 | chr18:38624758-38624817 | Ndfip1 |
| 2.7021111 | down | NM_026678 | chr2:126922489-126922548 | Blvra |
| 2.0048629 | down | NM_133930 | chr6:113442957-113443016 | Creld1 |
| 2.3150714 | down | NM_024467 | chr8:97850201-97850142 | Zfp319 |
| 2.7835925 | down | AK039092 | chr16:17548988-17548929 | 4930451C15Rik |
| 4.5101512 | down | NM_180600 | chr9:55054080-55054139 | Ube2q2 |
| 2.101125 | down | NM_053075 | chr5:24309128-24309069 | Rheb |
| 2.8523005 | down | NM_011392 | chr13:55514826-55514885 | Slc34a1 |
| 3.3531407 | down | NM_172253 | chr12:34123518-34123577 | Twistnb |
| 12.5179291 | down | NM_145078 | chr4:146986112-146986053 | 2610305D13Rik |
| 6.2424917 | down |  | chr10:071261986-071261927 |  |
| 4.2259081 | down | NM_146215 | chr8:112748013-112748072 | Cmtr2 |
| 3.359686 | down | NM_183194 | chr15:63689366-63689307 | Gsdmc3 |
| 9.952176 | down | NM_001013377 | chr4:43509077-43509018 | Arhgef39 |
| 3.4601592 | down | NM_145450 | chr12:114054174-114054233 | BC022687 |
| 2.6543117 | down | NM_001285785 | chr10:126766930-126766989 | Arhgap9 |
| 3.1079804 | down | NM_178778 | chr2:38921969-38921910 | Scai |
| 3.2717121 | down | NM_021430 | chr5:124943497-124943438 | Rilpl1 |
| 5.2293876 | down | NM_145625 | chr15:101926483-101926542 | Eif4b |
| 3.59888 | down | NM_008056 | chr15:38869107-38869166 | Fzd6 |
| 3.1420737 | down | NM_029788 | chr11:86312391-86312450 | Rnft1 |
| 2.5585333 | down | NM_001033228 | chr13:115758419-115756765 | Itga1 |
| 36.6734605 | down | NM_010941 | chrX:70202803-70202862 | Nsdhl |
| 8.5345513 | down | NM_172742 | chr7:71484723-71484782 | Mtmr10 |
| 48.8077791 | down | NM_153794 | chr18:68426509-68426450 | Fam210a |
| 2.2203375 | down |  | chr1:075633315-075633374 |  |
| 2.7876009 | down | NM_001198933 | chr9:86475136-86475077 | Me1 |
| 2.624719 | down | NM_026740 | chr11:78285379-78285438 | Slc46a1 |
| 16.5811539 | down | NM_008194 | chrX:82956449-82956390 | Gyk |
| 3.0678969 | down | NM_145548 | chr4:95723288-95712305 | Cyp2j13 |
| 4.9118857 | down | NM_019922 | chr9:114284518-114284459 | Crtap |
| 5.3859476 | down | NM_001033222 | chr19:59373902-59373843 | Pdzd8 |
| 3.4047916 | down | NM_026111 | chr7:19726300-19726241 | Qpctl |
| 2.7090913 | down | NM_019537 | chr16:96201703-96201644 | Psmg1 |
| 2.5918568 | down | NM_026395 | chr4:154448600-154448541 | Rer1 |
| 2.2418889 | down |  | chr9_random:000367776-000367717 | |
| 2.4760137 | down | NM_016982 | chr16:16868618-16868559 | Vpreb1 |
| 6.1735773 | down | NM_178882 | chr1:95748625-95748684 | D2hgdh |
| 2.2258301 | down | NM_133354 | chr11:115384482-115384423 | Sumo2 |
| 2.8348569 | down | NM_001160262 | chr1:169021039-169021098 | Fam78b |
| 3.7198607 | down | NM_144875 | chr1:133769161-133769220 | Rab7l1 |
| 2.2557033 | down | NM_009829 | chr6:127098755-127098696 | Ccnd2 |
| 10.9017097 | down | NM_029522 | chr3:108482281-108482222 | Gpsm2 |
| 2.7352373 | down | NM_013536 | chr6:124655192-124655064 | Emg1 |
| 19.8866174 | down | NM_008537 | chr15:10925139-10925198 | Amacr |
| 3.0354975 | down | NM_019998 | chr4:47482813-47482754 | Alg2 |
| 2.7521937 | down | NM_012005 | chrX:12322895-12321919 | Med14 |
| 2.4022337 | down | NM_001110242 | chr11:119961670-119961729 | 1810043H04Rik |
| 8.0330803 | down | NM_172597 | chr14:45755001-45754942 | Txndc16 |
| 2.4983545 | down | NM_145494 | chr18:73929835-73929776 | Me2 |
| 2.7624112 | down | NM_025275 | chr11:109299319-109299378 | Amz2 |
| 3.7914771 | down | AK013682 | chrX:157239436-157239377 | 2900054C01Rik |
| 9.2359125 | down | NM_028019 | chr11:80012536-80012595 | Rnf135 |
| 8.8810066 | down | NM_008017 | chr4:52501012-52501071 | Smc2 |
| 4.8989209 | down | NM_033616 | chr8_random:111631-111572 | Csprs |
| 3.8921888 | down | NM_198127 | chr1:60537669-60537728 | Abi2 |
| 5.5613025 | down | NM_026708 | chr11:77993064-77993123 | Tlcd1 |
| 2.6520831 | down |  | chr7:050209085-050209026 |  |
| 3.9741541 | down | NM_133755 | chr7:147185200-147185141 | Tubgcp2 |
| 30.540365 | down | NM_022014 | chr11:121301407-121301466 | Fn3k |
| 2.7511866 | down | NM_001267695 | chr3:95360245-95360304 | Ctss |
| 18.3319341 | down | NM_025335 | chr13:90246784-90246843 | Tmem167 |
| 3.5707843 | down | NM_028863 | chr12:86115608-86115667 | Isca2 |
| 2.3100467 | down | NM_172528 | chr9:77278998-77278939 | Lrrc1 |
| 6.7210703 | down | NM_010219 | chr6:128380587-128380528 | Fkbp4 |
| 2.3230165 | down | NM_001033430 | chr6:39091931-39091872 | Jhdm1d |
| 2.0834378 | down | NM_026740 | chr11:78285251-78285310 | Slc46a1 |
| 2.3843632 | down | NM_023538 | chr6:40346656-40346715 | Agk |
| 5.5450297 | down |  | chr13:025674320-025674261 |  |
| 2.2283502 | down | NM_178845 | chr12:41042457-41042398 | Zfp277 |
| 2.0307195 | down | NM_023119 | chr4:149622928-149622987 | Eno1 |
| 3.3761289 | down | NM_009725 | chr3:105745841-105745782 | Atp5f1 |
| 3.242029 | down | NM_133906 | chr5:138548978-138549037 | Zkscan1 |
| 6.2660223 | down | NM_198161 | chrX:132424180-132424239 | Bhlhb9 |
| 2.0615279 | down | NM_011020 | chr3:40594227-40594286 | Hspa4l |
| 2.5122717 | down | NM_001145452 | chr17:80769661-80769720 | Arhgef33 |
| 2.1981647 | down | NM_025360 | chr9:89594110-89594051 | Tmed3 |
| 7.0838418 | down | NM_181325 | chr8:23487931-23487872 | Slc25a15 |
| 2.2355463 | down | AK042594 | chr2:129023056-129022997 | Gm4430 |
| 3.9007377 | down | NM_145615 | chr9:55309487-55309428 | Etfa |
| 3.6755955 | down | NM_023113 | chr11:73119110-73119051 | Aspa |
| 8.6075222 | down | NM_175283 | chr13:69712435-69712376 | Srd5a1 |
| 2.1376698 | down | NM_207523 | chr11:77995065-77995006 | Rpl23a |
| 5.383829 | down | NM_133231 | chr3:54607303-54607244 | Rfxap |
| 5.3272057 | down | NM_153594 | chr2:181591961-181592020 | Pcmtd2 |
| 2.1936639 | down | NM_001142441 | chr8:98349850-98349909 | Gm10094 |
| 2.7715269 | down | NM_020586 | chr9:24912869-24912810 | Herpud2 |
| 4.0710346 | down | NM_001199274 | chr11:40493028-40492969 | Mat2b |
| 2.450425 | down | NM_011353 | chr13:100884007-100884066 | Serf1 |
| 2.8569187 | down | NM_026274 | chr8:97174121-97174180 | Rspry1 |
| 9.5683191 | down | NM_133649 | chr2:112202989-112203048 | Slc12a6 |
| 3.7061776 | down | NM_011739 | chr12:21404163-21404104 | Ywhaq |
| 5.7688966 | down | NM_001081356 | chrX:69065454-69065513 | Vma21 |
| 3.5167883 | down | NM_001039555 | chr19:39763479-39763420 | Cyp2c68 |
| 2.6516273 | down | NM_144784 | chr9:53389927-53389868 | Acat1 |
| 3.7298187 | down |  | chr11:104432045-104431986 |  |
| 2.4367107 | down | NM_009272 | chr4:147968254-147968313 | Srm |
| 2.6136093 | down |  | chr11:050891528-050891587 |  |
| 2.813544 | down | NM_001005423 | chr1:72206107-72206048 | Mreg |
| 4.8444578 | down | NM_016807 | chr4:6308184-6308243 | Sdcbp |
| 2.9951827 | down | NM_152809 | chr18:54114992-54115051 | Csnk1g3 |
| 13.6110768 | down | NM_024263 | chr4:155217688-155217747 | Mxra8 |
| 5.645659 | down |  | chr7:034791458-034791517 |  |
| 4.4287974 | down | NM_145925 | chr10:77061127-77061186 | Pttg1ip |
| 6.7149949 | down | NM_030554 | chr18:70143544-70143485 | Rab27b |
| 2.3236971 | down | NM_175277 | chr6:83308327-83308386 | Bola3 |
| 2.0169677 | down | NM_025794 | chr3:79407865-79407806 | Etfdh |
| 2.7716675 | down | NM_001105245 | chrX:130122415-130122356 | Pcdh19 |
| 13.4765686 | down | NM_001081068 | chr16:87377840-87377781 | Ltn1 |
| 7.7739186 | down | NM_009748 | chr6:4027238-4027179 | Bet1 |
| 10.0458205 | down | NM_173866 | chr8:88050856-88050915 | Gpt2 |
| 8.2141597 | down | NM_025935 | chr13:43248504-43248445 | Tbc1d7 |
| 25.3545085 | down | NM_028836 | chr3:146128348-146128407 | Ctbs |
| 2.8673997 | down | NM_009881 | chr13:35965223-35965282 | Cdyl |
| 4.6918582 | down | NM_027007 | chr18:24122872-24122931 | Zfp397 |
| 2.7548657 | down | NM_013477 | chr8:108048837-108048778 | Atp6v0d1 |
| 2.9421125 | down | NM_144953 | chr1:52982475-52982416 | 1700019D03Rik |
| 3.2274199 | down | NM_177214 | chr2:127064236-127064295 | Snrnp200 |
| 51.0296167 | down | NM_028696 | chr1:51527376-51527317 | Nabp1 |
| 3.6879804 | down | NM_029963 | chr2:127426599-127427149 | Mrps5 |
| 3.8585067 | down | NM_007481 | chr12:70474488-70474547 | Arf6 |
| 4.1287166 | down | XR_035099 | chr15:75052403-75052462 |  |
| 2.3435268 | down | NM_015772 | chr14:52931053-52930994 | Sall2 |
| 4.8104518 | down | NM_001252508 | chr12:106731169-106731228 | D430019H16Rik |
| 3.8297255 | down | NM_013541 | chr19:4037365-4037306 | Gstp1 |
| 4.5138566 | down | NM_001082975 | chr14:56516504-56516445 | Sdr39u1 |
| 2.9376754 | down | NM_026602 | chr3:102982683-102982742 | Bcas2 |
| 2.2448219 | down | AK018453 | chr1:43150589-43150648 |  |
| 3.8923272 | down | BC103784 |  | Gm7120 |
| 3.6610551 | down |  | chr6:102887406-102887347 |  |
| 2.2924972 | down | NM_134154 | chr19:5885329-5885388 | Slc25a45 |
| 4.9084016 | down | NM_019870 | chrX:71163242-71163183 | Naa10 |
| 3.2291411 | down | NM_007502 | chr9:96233375-96233316 | Atp1b3 |
| 3.6313216 | down | NM_146041 | chr13:31911594-31911535 | Gmds |
| 7.6807117 | down | NM_008822 | chr10:19580696-19580638 | Pex7 |
[truncated: 475,054 more chars]
